# Excess hydrogen sulfide and polysulfides production underlies a schizophrenia pathophysiology

**DOI:** 10.15252/emmm.201910695

**Published:** 2019-10-28

**Authors:** Masayuki Ide, Tetsuo Ohnishi, Manabu Toyoshima, Shabeesh Balan, Motoko Maekawa, Chie Shimamoto‐Mitsuyama, Yoshimi Iwayama, Hisako Ohba, Akiko Watanabe, Takashi Ishii, Norihiro Shibuya, Yuka Kimura, Yasuko Hisano, Yui Murata, Tomonori Hara, Momo Morikawa, Kenji Hashimoto, Yayoi Nozaki, Tomoko Toyota, Yuina Wada, Yosuke Tanaka, Tadafumi Kato, Akinori Nishi, Shigeyoshi Fujisawa, Hideyuki Okano, Masanari Itokawa, Nobutaka Hirokawa, Yasuto Kunii, Akiyoshi Kakita, Hirooki Yabe, Kazuya Iwamoto, Kohji Meno, Takuya Katagiri, Brian Dean, Kazuhiko Uchida, Hideo Kimura, Takeo Yoshikawa

**Affiliations:** ^1^ Laboratory of Molecular Psychiatry RIKEN Center for Brain Science Wako Saitama Japan; ^2^ Department of Psychiatry Division of Clinical Medicine Faculty of Medicine University of Tsukuba Tsukuba Ibaraki Japan; ^3^ Support Unit for Bio‐Material Analysis, Research Division RIKEN Center for Brain Science Wako Saitama Japan; ^4^ Research& Development Department MCBI Inc Tsukuba Ibaraki Japan; ^5^ Department of Pharmacology Sanyo‐Onoda City University Sanyo‐Onoda Yamaguchi Japan; ^6^ Department of Molecular Pharmacology National Institute of Neuroscience National Center of Neurology and Psychiatry Kodaira, Tokyo Japan; ^7^ Department of Molecular Brain Science Graduate School of Medical Sciences Kumamoto University Kumamoto Japan; ^8^ Department of Organ Anatomy Tohoku University Graduate School of Medicine Sendai Miyagi Japan; ^9^ Department of Cell Biology and Anatomy Graduate School of Medicine The University of Tokyo Tokyo Japan; ^10^ Division of Clinical Neuroscience Chiba University Center for Forensic Mental Health Chiba Japan; ^11^ Graduate School of Humanities and Sciences Ochanomizu University Tokyo Japan; ^12^ Laboratory for Molecular Dynamics of Mental Disorders RIKEN Center for Brain Science Wako Saitama Japan; ^13^ Department of Pharmacology Kurume University School of Medicine Kurume Fukuoka Japan; ^14^ Laboratory for Systems Neurophysiology RIKEN Center for Brain Science Wako Saitama Japan; ^15^ Department of Physiology Keio University School of Medicine Tokyo Japan; ^16^ Center for Medical Cooperation Tokyo Metropolitan Institute of Medical Science Tokyo Japan; ^17^ Department of Neuropsychiatry School of Medicine Fukushima Medical University Fukushima Japan; ^18^ Department of Psychiatry Aizu Medical Center Fukushima Medical University Aizuwakamatsu Fukushima Japan; ^19^ Department of Pathology Brain Research Institute Niigata University Niigata Japan; ^20^ Department of Pharmacy Faculty of Pharmacy Iryo Sosei University Iwaki Fukushima Japan; ^21^ The Florey Institute of Neuroscience and Mental Health Howard Florey Laboratories The University of Melbourne Parkville Vic. Australia; ^22^ The Centre for Mental Health Swinburne University Hawthorn Vic. Australia; ^23^ Department of Molecular Oncology Division of Biomedical Science Faculty of Medicine University of Tsukuba Tsukuba Ibaraki Japan

**Keywords:** energy metabolism, epigenetics, hydrogen sulfide and polysulfides, prepulse inhibition, proteomics, Chromatin, Epigenetics, Genomics & Functional Genomics, Neuroscience

## Abstract

Mice with the C3H background show greater behavioral propensity for schizophrenia, including lower prepulse inhibition (PPI), than C57BL/6 (B6) mice. To characterize as‐yet‐unknown pathophysiologies of schizophrenia, we undertook proteomics analysis of the brain in these strains, and detected elevated levels of Mpst, a hydrogen sulfide (H_2_S)/polysulfide‐producing enzyme, and greater sulfide deposition in C3H than B6 mice. *Mpst*‐deficient mice exhibited improved PPI with reduced storage sulfide levels, while *Mpst*‐transgenic (Tg) mice showed deteriorated PPI, suggesting that “sulfide stress” may be linked to PPI impairment. Analysis of human samples demonstrated that the H_2_S/polysulfides production system is upregulated in schizophrenia. Mechanistically, the *Mpst‐*Tg brain revealed dampened energy metabolism, while maternal immune activation model mice showed upregulation of genes for H_2_S/polysulfides production along with typical antioxidative genes, partly via epigenetic modifications. These results suggest that inflammatory/oxidative insults in early brain development result in upregulated H_2_S/polysulfides production as an antioxidative response, which in turn cause deficits in bioenergetic processes. Collectively, this study presents a novel aspect of the neurodevelopmental theory for schizophrenia, unraveling a role of excess H_2_S/polysulfides production.

## Graphical abstract

This study proposes a novel concept that excess hydrogen sulfide production (sulfide stress) underlies a schizophrenia pathophysiology in the realm of neurodevelopmental hypothesis of the disease. Targeting the metabolic pathway of hydrogen sulfide provides a novel therapeutic approach.

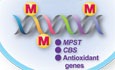

## Introduction

Schizophrenia is a severe mental illness featuring three major symptomatic domains: positive symptoms (hallucinations, delusions, etc.), negative symptoms (affective flattening, avolition, etc.), and cognitive deficits (disorganized thought, etc.) (American Psychiatric Association, 2013). This illness exhibits a life‐time prevalence of approximately one percent worldwide. Repeated relapses of psychotic symptoms often lead to a deterioration of brain function, and eventually to end‐stage illness in some cases, characterized by persistent symptoms and profound functional disabilities (Lewis & Lieberman, 2000). Though causal mechanisms are elusive, accumulating lines of evidence have shown abnormalities in early neurodevelopment processes, stemmed from genetic aberrations and environmental factors, such as maternal immune activation, for the etiopathogenesis of schizophrenia (neurodevelopmental hypothesis) (Knuesel *et al*, 2014; Estes & McAllister, 2016; Birnbaum & Weinberger, 2017). And for symptomatic treatments, drugs targeting dopaminergic systems are predominantly used (Murray *et al*, 2017). However, because currently available therapeutics has limitations in terms of efficacies and adverse effects, a new paradigm is needed for the development of novel drugs.

Here, we hypothesize that examination of the traits and associated molecular underpinnings in inbred mouse strains could potentially identify as‐yet‐unknown pathophysiologies of schizophrenia. Pursuing this hypothesis, we have already reported that across 4 strains of mice, C57BL/6N (B6) mice exhibited the highest prepulse inhibition (PPI) scores while C3H/HeN (C3H) the lowest (Watanabe *et al*, 2007). PPI is the normal suppression of a startle response when a low‐intensity stimulus immediately precedes an unexpected stronger startling stimulus. As a reproducible phenotypic marker, impaired PPI reflects sensorimotor gating deficits, and is typically regarded as an endophenotype for schizophrenia (Braff *et al*, 2001; Roussos *et al*, 2016).

To explore the molecular signature underlying the behavioral differences between B6 and C3H, we performed proteomic analyses, using 2D‐DIGE (two‐dimensional difference gel electrophoresis) and MALDI‐TOF MS (matrix‐assisted laser desorption/ionization time‐of‐flight mass spectrometry). This screening step revealed an increase in the levels of a hydrogen sulfide (H_2_S)‐ and polysulfides‐ (H_2_S/polysulfides)‐producing enzyme, Mpst (3‐mercaptopyruvate sulfurtransferase; also known as 3MST) (Appendix Fig S1), in C3H mice compared to B6 animals. Then, we comprehensively assessed the biological possibility of the novel theory of “excessive H_2_S/polysulfides production” in schizophrenia, and pursued the mechanism underlying the functional consequence and causative origin of this phenomenon.

## Results

### Proteomic analyses of brain and splenic lymphocytes from B6 and C3H mice identified Mpst

**Figure 1:**
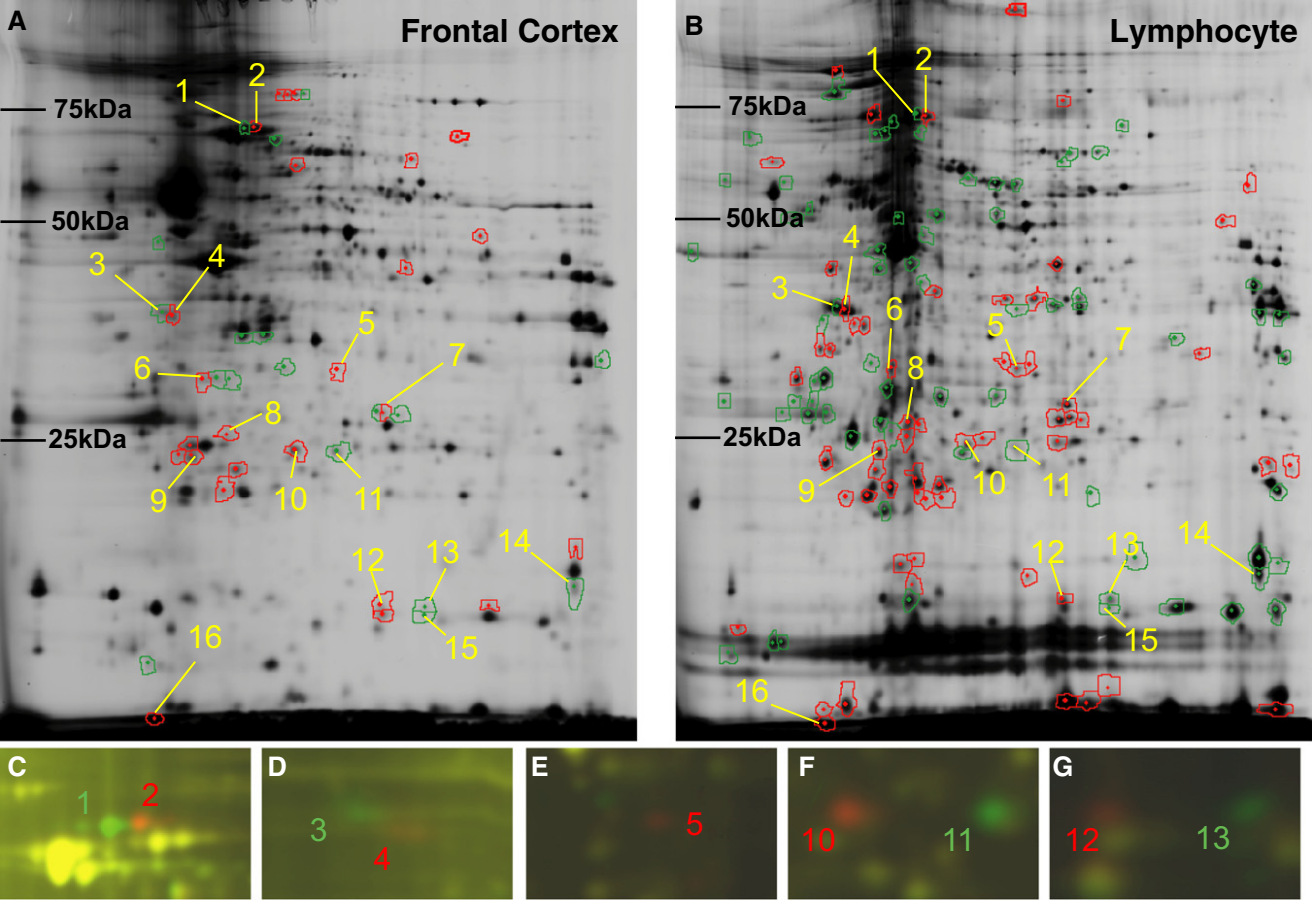
Differential proteomic analysis of brain and lymphocyte tissues from B6 and C3H mice using 2D‐DIGE A, BResults of 2D‐DIGE on frontal cortex tissue (A) and lymphocytes (B). Green and red rings denote spots showing significantly increased expression in B6 and C3H mice, respectively.C–GMerged images of Cy3 (green) and Cy5 (red): green and red spots represent significantly increased expression of the corresponding protein isoforms in B6 and C3H mice, respectively. Nine (spot nos. 1, 2, 3, 4, 5, 10, 11, 12 and 13) out of the 16 spots were successfully identified as mortalin (Hspa9) (C), nucleophosmin (Npm1) (D), mercaptopyruvate sulfurtransferase (Mpst) (E), peroxiredoxin 6 (Prdx6) (F) and nucleoside diphosphate kinase B (Nme2) (G).Data information: Significance for differential expression between B6 and C3H was defined as *P* value of < 0.05 (unpaired two‐tailed *t*‐test) and fold change > 1.2. Yellow numbers indicate the 16 spots that showed consistent alterations between brain and lymphocyte samples from the two mouse strains.Source data are available online for this figure.

We performed proteomic analyses using brain and lymphocyte preparations from B6 and C3H mice to detect a homologous biomarker in the peripheral blood of schizophrenia patients. As shown in Fig 1A and B, and Appendix Tables S1 and S2, of the 1,093 spots identified from the brain using 2D‐DIGE, 43 showed significant differences in expression between B6 and C3H animals, with our criteria of a fold change > 1.2 and *P* < 0.05. Of the 1,400 spots detected from the lymphocyte preparations, 131 showed significant differences in expression between B6 and C3H mice. Sixteen of these differentially expressed protein spots showed consistent change trends between the tissues from the two mouse strains (Appendix Table S2).

**Figure 2:**
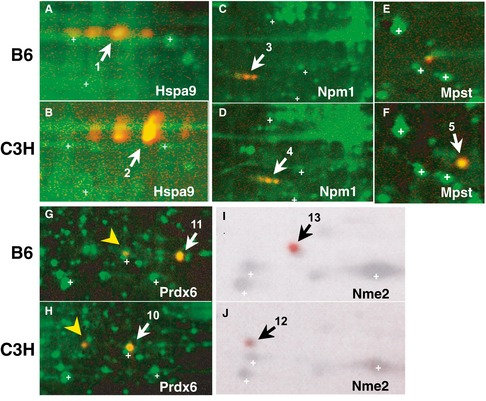
Identified proteins visualized by 2D Western blotting A–JWhole protein extracts from brain tissue of B6 (A, E, G, I). Whole protein extracts from brain tissue of C3H (B, F, H, J). Whole protein extracts from lymphocytes of B6 (C) and C3H (D). Npm3 expression levels were low in the brain (C, D). Hspa9 (mortalin) (A, B), Npm1 (nucleophosmin) (C, D), Mpst (mercaptopyruvate sulfurtransferase) (Mpst) (E, F), Prdx6 (peroxiredoxin 6) (G, H) and Nme2 (nucleoside diphosphate kinase B) (I, J) were detected by 2D Western blotting using the corresponding antibodies and chemiluminescence (red). The chemiluminescent signal of Nme2 was visualized by the LAS 3000 chemiluminescence image analyzer and the other signals were visualized by a Typhoon 9400.Data information: White crosses (+) indicate landmark spots. Spot numbers (indicated by arrows) correspond to the spot numbers in Fig 1. Yellow arrowheads (G, H) indicate the overoxidized form of Prdx6.

We successfully identified the molecular entities of nine of these protein spots, by peptide mass fingerprinting (PMF), and found five different proteins: heat shock 70 kDa protein 9 (mortalin, Hspa9), nucleophosmin/nucleoplasmin (Npm1), Mpst, peroxiredoxin 6 (Prdx6), and nucleoside diphosphate kinase B (Nme2) (Appendix Tables S3 and S4). The proteins Hspa9, Npm1, Prdx6, and Nme2 appeared as paired spots at different isoelectric points (pI) (Fig 1C, D, F and G). Sequencing of the genes encoding these proteins revealed that each gene harbored single‐nucleotide polymorphisms (SNPs) or insertion/deletion polymorphisms, which altered the amino acid sequences between the two mouse strains (Appendix Table S5 and Appendix Fig S2). The theoretical pI and molecular mass values calculated based on DNA sequences and possible posttranslational modifications were consistent with the observed spot profiles of these proteins in 2D gels, and were confirmed by 2D Western blotting (Fig 2A–J). By using 2D‐DIGE, Mpst appeared as a single spot that was expressed in only C3H mice (Fig 1E). Two‐dimensional Western blotting confirmed this spot for C3H animals (Fig 2F) and detected a faint spot for B6 mice at a different pI from the spot observed for C3H mice (Fig 2E). Sequencing of the *Mpst* gene revealed a Asp102Gly polymorphism (Appendix Table S5 and Appendix Fig S2), which can explain the differential mobility in the 2D gel between the two mouse strains. The Mpst protein catalyzes the transfer of a sulfur ion from 3‐mercaptopyruvate to cyanide or other thiol compounds (Szabo, 2007; Kimura, 2015; Wallace & Wang, 2015), and this reaction produces H_2_S/polysulfides and detoxifies cyanide (Appendix Fig S1). The Asp102Gly polymorphism is predicted to have little effect (“benign”) by the PolyPhen‐2 algorithm (http://genetics.bwh.harvard.edu/pph2/). Indeed, the functional assay conducted by preparing Asp102 and Gly102 Mpst constructs showed no significant differences in enzymatic activity between the variants (Appendix Fig S3 and Appendix Table S6).

**Figure EV1:**
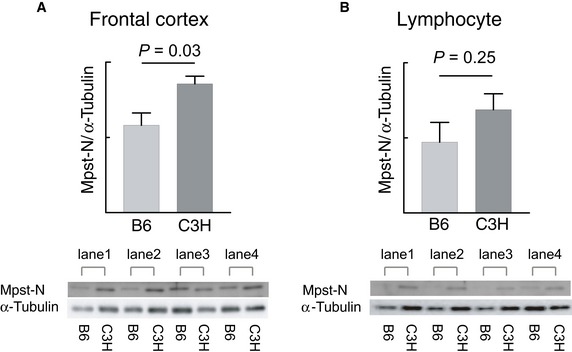
Expression of Mpst examined using anti‐N‐terminal Mpst antibodies A, BMpst protein levels in the brain and lymphocytes from B6 (*n* = 4) and C3H (*n* = 4) mice were quantified by standard Western blotting with anti‐N‐terminal Mpst antibodies. The expression levels of Mpst were normalized using α‐tubulin. Bar graphs show the mean expression levels of Mpst in brain (A) and lymphocyte (B) tissues.Data information: *P* values were calculated by using unpaired two‐tailed *t*‐test. The values represent the mean ± SD.

**Figure 3:**
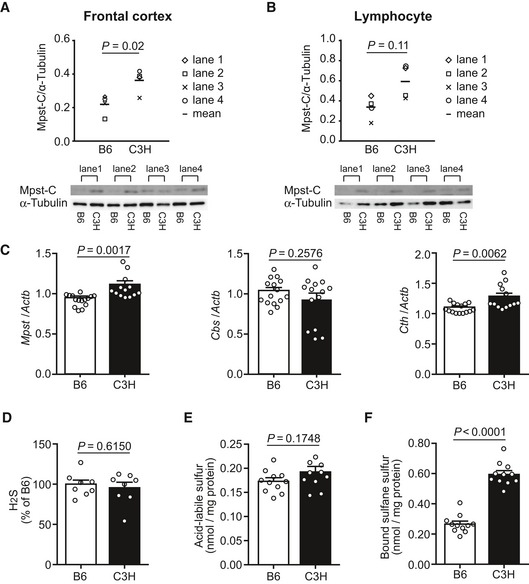
Expression of Mpst/*Mpst* and genes encoding the other H_2_S‐producing enzymes and profile of H_2_S metabolic states in mice A, BMpst protein levels in the brain and lymphocytes from B6 and C3H mice were quantified by standard Western blotting with anti‐C‐terminal Mpst antibodies (for anti‐N‐terminal Mpst antibodies, see Fig EV1). The expression levels of Mpst were normalized using α‐tubulin. Bar graphs show the mean expression levels of Mpst in brain (A) and lymphocyte (B) tissues.CTranscript expression levels of genes encoding three H_2_S‐producing enzymes in the frontal cortex of B6 (*n* = 15) and C3H mice (*n* = 14). The values represent the mean ± SEM.DH_2_S content in the frontal cortex of B6 (*n* = 8) and C3H mice (*n* = 8). The values are relative to those of B6 and represent mean ± SEM.ELevels of labile sulfur in the frontal cortex of B6 (*n* = 10) and C3H mice (*n* = 10). The values are relative to those of B6 and represent mean ± SEM.FLevels of bound sulfane sulfur in the frontal cortex of B6 (*n* = 10) and C3H mice (*n* = 10). The values are relative to those of B6 and represent mean ± SEM.Data information: *P* values were calculated using unpaired two‐tailed *t*‐test.Source data are available online for this figure.

The Mpst spot was the only protein to show differential expression, exhibiting lower expression in B6 mice than in C3H mice. The protein expression levels for Mpst were confirmed by standard Western blot analyses of B6 and C3H mice using both brains and splenic lymphocytes: significantly higher expression of Mpst was observed in the frontal cortex of the C3H mouse brain than in that of the B6 brain using both anti‐Mpst N‐terminus (Mpst‐N, *P* = 0.03, Fig EV1A) and anti‐Mpst C‐terminus (Mpst‐C, *P* = 0.02, Fig 3A) antibodies, though only a marginally increased expression of Mpst was observed in C3H mice in splenic lymphocytes (Mpst‐N, *P* = 0.25; Mpst‐C, *P* = 0.11) (Figs 3B and EV1B). Because of the nonsignificant differences in protein expression levels in lymphocytes between the two strains, we hereafter focused on mainly brain tissues.

### Sulfide content in the brain was higher in C3H than in B6 mice

Next, we examined whether upregulation of Mpst caused elevated deposition of the free form of sulfur, i.e., H_2_S, and/or stored sulfur, i.e., acid‐labile sulfur and bound sulfane sulfur, which includes H_2_S_n_ (*n* = 2: persulfide, *n* > 2: polysulfides) and persulfurated‐cysteine, ‐glutathione (GSH), and ‐proteins (Appendix Fig S4) (Kimura, 2015). H_2_S_2_ and H_2_S_3_ were recently shown to be generated from 3‐mercaptopyruvate by Mpst and to be present in the brain (Kimura *et al*, 2013, 2015, 2017; Koike *et al*, 2017; Nagahara *et al*, 2018). Acid‐labile sulfur is mainly found in an iron–sulfur complex attached to enzymes belonging to the respiratory chain including aconitase (Ishigami *et al*, 2009) (Appendix Fig S4). This complex releases H_2_S under acidic conditions, but not under physiological conditions. Bound sulfane sulfur releases H_2_S under reducing conditions (Appendix Fig S4) (Ishigami *et al*, 2009). As shown in Fig 3D and E, the H_2_S and acid‐labile sulfur content was unchanged between the two mouse strains, but the bound sulfane sulfur content was markedly elevated in the C3H brain compared to the B6 brain (Fig 3F), suggesting an increase in the levels of H_2_S_n_ and per‐ and poly‐sulfurated molecules. The endogenous H_2_S produced by enzymes may be readily oxidized to H_2_S_n_ and incorporated into bound sulfane sulfur (Shibuya *et al*, 2009; Kimura *et al*, 2015).

### *Mpst* deficiency or overexpression, and external sulfides affect mouse behaviors

**Figure 4:**
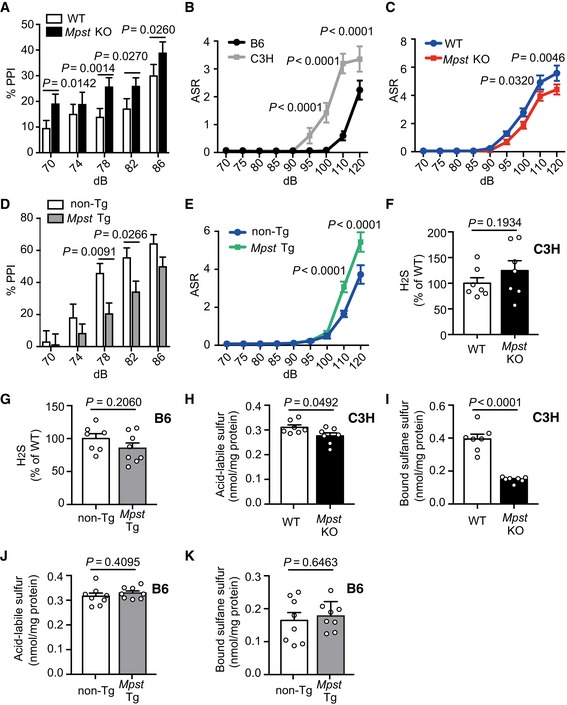
Mouse behaviors and sulfide deposition in *Mpst* knockout (KO) and transgenic (Tg) mice APrepulse inhibition (PPI) levels (%) of C3H wild‐type littermates (*n* = 17) and *Mpst*‐KO C3H (*n* = 17) mice. The *X*‐axis shows prepulse levels. The pulse sound level was 115 dB.BAcoustic startle response (ASR) levels of inbred B6 (*n* = 12) and C3H (*n* = 12) mice. The unit is arbitrary. The *X*‐axis shows pulse sound levels.CASR levels of C3H wild‐type littermates (*n* = 17) and *Mpst*‐KO C3H mice (*n* = 17). The unit is arbitrary. The *X*‐axis shows pulse sound levels.DPPI levels of B6 wild‐type littermates (*n* = 17) and *Mpst*‐Tg B6 mice (*n* = 19). The *X*‐axis shows prepulse levels. The pulse sound level was 115 dB.EASR levels of B6 wild‐type littermates (*n* = 17) and *Mpst*‐Tg B6 mice (*n* = 19). The unit is arbitrary. The *X*‐axis shows pulse sound levels.FH_2_S content in the frontal cortex of C3H wild‐type littermates (*n* = 7) and *Mpst*‐KO C3H mice (*n* = 7).GH_2_S content in the frontal cortex of B6 wild‐type littermates (*n* = 7) and *Mpst*‐Tg B6 mice (*n* = 7).H, ILevels of acid‐labile sulfur (H) and bound sulfane sulfur (I) in the frontal cortex of C3H wild‐type littermates (*n* = 7) and *Mpst*‐KO C3H mice (*n* = 7).J, KLevels of acid‐labile sulfur (J) and bound sulfane sulfur (K) in the frontal cortex of B6 wild‐type littermates (*n* = 7) and *Mpst*‐Tg B6 mice (*n* = 7).Data information: The values represent the mean ± SEM. *P* values were calculated using Sidak's multiple comparisons test (A–E) or unpaired two‐tailed *t*‐test (F–K).

Since Mpst was observed to be overexpressed in C3H when compared to that in B6, it is important to determine whether Mpst plays a role in the distinct PPI levels between B6 and C3H. To assess the role of Mpst, we generated *Mpst* knockout (KO) mice in the C3H background (Appendix Fig S5), and *Mpst‐*transgenic (Tg) mice in the B6 background (Appendix Fig S6). The *Mpst‐*KO mice showed elevated PPI at a prepulse level of 78 dB compared to their wild‐type (WT) littermates (Fig 4A). Interestingly, C3H inbred mice exhibited an enhanced acoustic startle response (ASR) compared to B6 (Fig 4B), which has been previously reported in schizophrenic model mice (Egashira *et al*, 2005; Duncan *et al*, 2006). *Mpst‐*KO mice in the C3H background showed diminished ASR relative to their WT littermates (Fig 4C). In contrast, the *Mpst‐*Tg mice exhibited decreased PPI at prepulse levels of 78 dB and 82 dB (Fig 4D) and magnified ASR compared to the non‐Tg littermates (Fig 4E). These results indicate that upregulation of the *Mpst* causes schizophrenia‐related impaired PPI and exaggerated ASR.

Regarding the sulfide levels in the brain, H_2_S levels were unaltered by the change in copy number of *Mpst* (Fig 4F and G), and the levels of acid‐labile sulfur were slightly decreased in the *Mps*t‐KO mice compared to the WT littermates (Fig 4H). Notably, the *Mpst‐*KO mice showed a dramatic decrease in bound sulfane sulfur levels compared to the WT mice (Fig 4I). These results indicate that when the level of Mpst is decreased, sulfide deposition in the brain is reduced. The *Mpst‐*Tg mice did not show any differences in the levels of both free and stored forms of sulfur (Fig 4G, J and K), probably because the magnitude of transgene expression was not sufficiently large (Appendix Fig S6) for the detection of distinct levels of sulfides in our assay system.

**Figure EV2:**
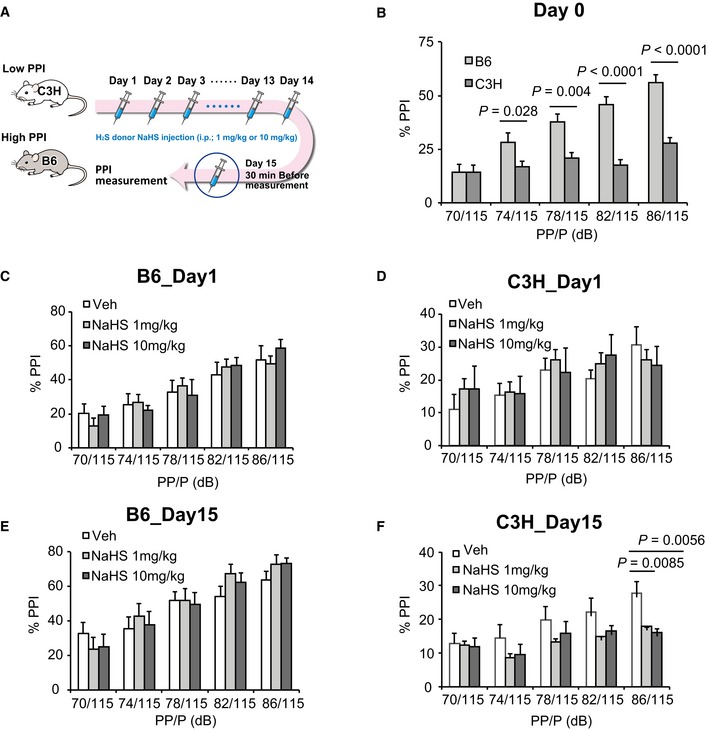
Effects of chronic NaHS administration on PPI (prepulse inhibition) ASchematic illustration of the experimental design.BPPI (%) at 5 different prepulse levels between untreated B6 (*n* = 12) and C3H (*n* = 12) mice. *X*‐axis shows prepulse (PP) levels. Pulse (P) sound was 115 dB.C, DPPI levels of B6 (C) and C3H (D) mice after the first administration of vehicle (Veh: PBS) and two different doses of NaHS. NaHS was intraperitoneally injected once a day, and PPI was measured 30 min after the 1^st^ injection. Each dose group contained *n* = 12 animals.E, FPPI levels of B6 (E) and C3H (F) mice after the 15^th^ administration of vehicle (Veh: PBS) and two different doses of NaHS. NaHS was intraperitoneally injected once a day, and PPI was measured 30 min after the 15^th^ injection. Each dose group contained *n* = 12 animals.Data information: *P* values were calculated using Tukey's *post hoc* test after one‐way ANOVA. All data are shown as the mean ± SEM.

Next, we examined whether external administration of the H_2_S‐producing agent, NaHS, can elicit an impaired PPI. Chronic injections of NaHS decreased the PPI of C3H mice, although that of B6 mice was unchanged (Fig EV2). B6 mice may be relatively resistant to excess H_2_S and polysulfides (also see Fig 4K).

### Expression of the H_2_S synthesis system is upregulated in schizophrenia

**Figure EV3:**
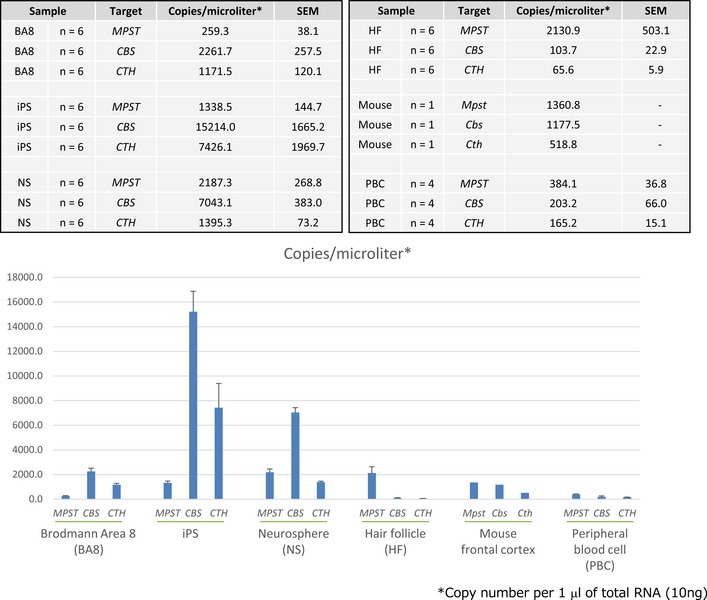
Absolute expression levels of genes for H_2_S‐synthesizing enzymes in human and mouse tissues Transcript expression levels were measured by digital PCR. Samples of BA8, iPSC‐derived NS, HF, and PBC were from human.

**Figure 5:**
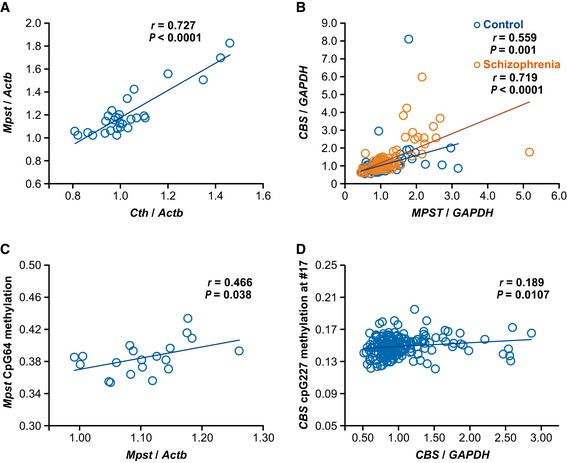
Correlation between expression levels of genes encoding H_2_S‐producing enzymes and between DNA methylation levels and gene expression levels Correlation between relative expression levels of *Mpst* and *Cth* in B6 (*n* = 15) and C3H (*n* = 14) mice.Correlation between relative expression levels of *MPST* and *CBS* in postmortem brain samples (control, *n* = 93; schizophrenia *n* = 95). For demographic data, see Appendix Table S7.Correlation between DNA methylation levels and relative expression levels of *Mpst* in B6 (*n* = 10) and C3H (*n* = 10) mice. Also see Appendix Fig S14.Correlation between DNA methylation levels and relative expression levels of *CBS* in postmortem brain samples from both control subjects and subjects with schizophrenia (*n* = 181). Also see Appendix Fig S17, and Appendix Table S7 for demographic data.Data information: Statistical evaluations were performed using Spearman's rank correlation test.

The results thus far demonstrated that upregulation of Mpst and concomitant accumulation of sulfides in the brain possibly causes the impairment of PPI, a representative biological trait of schizophrenia. There are two other well‐known enzymes, namely, Cbs/CBS (cystathionine‐beta‐synthase) and Cth/CTH (cystathionine gamma‐lyase), that are also involved in the production of H_2_S (Appendix Fig S1) (Szabo, 2007; Kimura, 2015; Wallace & Wang, 2015). Real‐time quantitative PCR (RT–qPCR) analyses revealed that the levels of the *Mpst* and *Cth* mRNAs increased in C3H mouse brains (Fig 3C). The absolute expression levels of the three genes in the mouse brain measured by digital RT–PCR showed the trend *Mpst* ~ *Cbs* > *Cth* (Fig EV3). Therefore, the higher sulfide levels in C3H than in B6 brains mainly stemmed from differential *Mpst* expressions between the two strains. Interestingly, the expression levels of the differentially expressed *Mpst* and *Cth* were positively correlated with each other (Fig 5A), indicating concerted operation of the H_2_S‐producing system.

**Figure 6:**
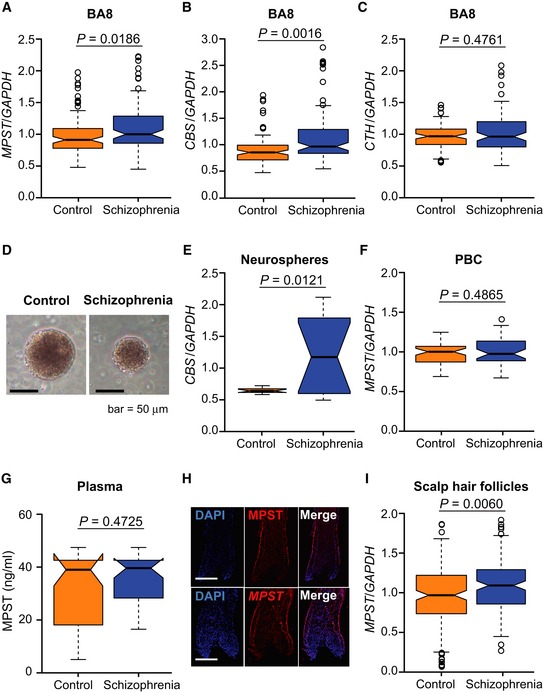
H_2_S‐producing genes/proteins in human samples A–CExpression of *MPST* (A), *CBS* (B), and *CTH* (C) in postmortem brain samples from controls (*n* = 93) and schizophrenia patients (*n* = 95) (1^st^ set). For demographic data, see Appendix Table S7.DNeurospheres from a control (left panel) and a schizophrenia patient (right panel). Scale bar, 50 μm.ERelative expression of *CBS* in neurosphere samples from controls (*n* = 12 cell lines) and schizophrenia patients (*n* = 12 cell lines).FRelative expression of *MPST* in peripheral blood cell (PBC) samples from controls (*n* = 56) and schizophrenia patients (*n* = 44). For demographic data, see Appendix Table S9.GProtein expression levels of MPST in plasma samples from controls (*n* = 56) and schizophrenia patients (*n* = 44). For demographic data, see Appendix Table S9.HExpression patterns of MPST/*MPST* in scalp hair follicles. The upper panels show MPST protein expression, and the lower panels show *MPST* mRNA expression. Nuclei were stained with DAPI (4′,6‐diamidino‐2‐phenylindole, dihydrochloride). Scale bars, 150 μm.IRelative expression of *MPST* and *CBS* in scalp hair follicle samples from controls (*n* = 166) and schizophrenia patients (*n* = 149). For demographic data, see Appendix Table S10.Data information: The boxplot graphs summarize statistical measures (median, the 75^th^ and 25^th^ percentiles, and minimum and maximum data values) of the distributions of relative target gene expression levels (using *GAPDH* as an internal control). BA8, Brodmann area 8 of postmortem brains. *P* values were calculated using two‐tailed Mann–Whitney *U* test.

In human postmortem brains [Brodmann area (BA) 8, frontal cortex] (1^st^ sample set) (Appendix Table S7), RT–qPCR analyses revealed increased expressions of *MPST* and *CBS* in subjects with schizophrenia (Fig 6A and B), with no change in *CTH* mRNA levels (Fig 6C). The absolute expression levels of the three genes in the human brain followed the trend *CBS* >* CTH* > *MPST* (Fig EV3), suggesting a relatively strong role of CBS in the regulation of the total sulfide levels in the human brain. The expression levels of differentially expressed *CBS* and *MPST* were well correlated with each other in both the control and schizophrenia brain samples (Fig 5B). As in the case of C3H mice, H_2_S/polysulfides production is thought to be upregulated by coordinated leveraging of multiple genes for H_2_S/polysulfides synthesis in the schizophrenia brain samples.

**Figure 7:**
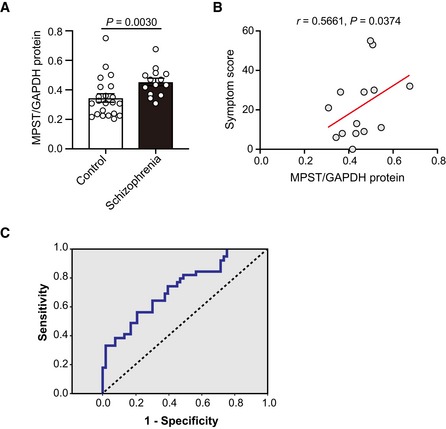
Assessments of MPST in postmortem brains, and its association with symptom severity score and *MPST* in hair follicle samples as a biomarker Protein expression levels of MPST in postmortem brain samples from controls (*n* = 22) and schizophrenia patients (*n* = 14) (2^nd^ set). Also see Appendix Table S8 for demographic data.Correlation between relative expression levels of MPST in postmortem brain samples from schizophrenia (*n* = 14) and symptom severity score rated by using the Diagnostic Instrument for Brain Studies (DIBS). Also see Appendix Table S8 for demographic data.Receiver Operating Characteristic (ROC) curve of *MPST*/*GAPDH* levels in the hair follicle sample set, relative to schizophrenia occurrence. Schizophrenia patients (*n* = 149) are the same to those used in Fig 6I. Regarding the discrimination efficiency of schizophrenia, the sensitivity and specificity were 73.6 and 47.2%, respectively (area under the curve = 0.602).Data information: The values represent the mean ± SEM (A). *P* value was calculated using two‐tailed Mann–Whitney *U* test (A). Correlation analysis was performed using Spearman's rank correlation test (B).Source data are available online for this figure.

To verify the idea of upregulated H_2_S/polysulfides production state in schizophrenia, we examined a different postmortem brain sample set (BA17) (2^nd^ sample set) (Ohnishi *et al*, 2019) (Appendix Table S8) and evaluated phenotypic features of schizophrenia that are associated with elevated H_2_S production system. In this sample set, the MPST protein expression levels were higher in the schizophrenia than in the control groups (Fig 7A). Interestingly, the MPST levels in schizophrenia were positively correlated with symptom severity scores (a sum of positive symptom score, negative symptom score, and general score) at 3 months prior to the death rated by using the Diagnostic Instrument for Brain Studies (DIBS) (Ohnishi *et al*, 2019) (Fig 7B). The results suggest that patients with schizophrenia under “sulfide stress” manifest relatively severe psychotic symptoms. Here it was difficult to precisely measure H_2_S/polysulfides levels in human postmortem brains, because for an accurate measurement, samples should have been quickly removed and frozen immediately after death.

We also examined reprogrammed neuronal lineage cells from patients with schizophrenia. Neurospheres are composed of free‐floating clusters of neural stem or progenitor cells, differentiated from induced pluripotent stem cells (iPSCs) (Maekawa *et al*, 2015; Toyoshima *et al*, 2016). iPSCs‐derived neurospheres from schizophrenia patients carrying 22q11.2 micro‐deletions (Fig 6D) (Bundo *et al*, 2014; Maekawa *et al*, 2015; Toyoshima *et al*, 2016) showed a significant increase in *CBS* mRNA compared to those derived from control subjects (Fig 6E). Absolute expression levels of *CBS* are much higher in early developmental cells, such as iPSCs and iPSCs‐derived neurospheres, than in terminally differentiated cells (postmortem brain cells, hair follicles, peripheral blood cells (PBCs), and mouse brain) (Fig EV3). The results indicate that the H_2_S production system might be already elevated from the early neurodevelopmental stage in schizophrenia, which is associated with “neurodevelopmental theory” (Rapoport *et al*, 2012; Birnbaum & Weinberger, 2017).

We next addressed whether H_2_S/polysulfides generation could be a biomarker of schizophrenia. The absolute transcript expression levels of the three genes for H_2_S/polysulfides production in PBCs were all very low, but *MPST* exhibited the highest expression levels (Fig EV3). However, the *MPST* levels in PBCs exhibited no significant changes between subjects with schizophrenia and control (Fig 6F, Appendix Table S9), and plasma MPST protein levels were also unchanged between the two groups (Fig 6G and Appendix Table S9). These results seem to correspond to the mouse data that in mouse lymphocytes Mpst expression levels were not different between B6 and C3H, and suggest that it would be difficult to identify a biomarker from peripheral blood. The large proportion of Mpst protein in blood sample exists in red blood cells (RBCs) (Włodek & Ostrowski, 1982). Because RBCs do not have nuclei, the levels of Mpst in plasma may not be affected by differential gene expression. Then, we examined hair follicles as a source of biomarker genes (*n* = 166 for control and *n* = 149 for schizophrenia) (Maekawa *et al*, 2015). The *MPST* was the only substantially expressed transcript among the three genes for H_*2*_S synthesis in this mini‐organ (Figs 6H and EV3) (Maekawa *et al*, 2019), and *MPST* mRNA expression was increased in subjects with schizophrenia (Fig 6I, Appendix Table S10). None of the confounding factors, such as age at examination, BMI (body mass index), anti‐psychotic drug dose, age of onset of schizophrenia, duration of the illness, and smoking, were significantly correlated with *MPST* mRNA expression in scalp hair follicles (Appendix Fig S7). Receiver operating characteristics (ROC) curve analysis determined an optimal cut‐off level of 0.876 based on the maximum Youden index. With this cut‐off level for the *MPST/GAPDH* mRNA ratio, the sensitivity and specificity were 73.6 and 47.2%, respectively (area under the curve = 0.602) (Fig 7C). The results suggest that the subset of schizophrenia with “sulfide stress” pathophysiology could be identified using this surrogate marker.

### Excess H_2_S/polysulfides production elicits dampened expression of genes for energy metabolism, and impairs mitochondrial energy metabolism and diminishes spine density

**Figure 8:**
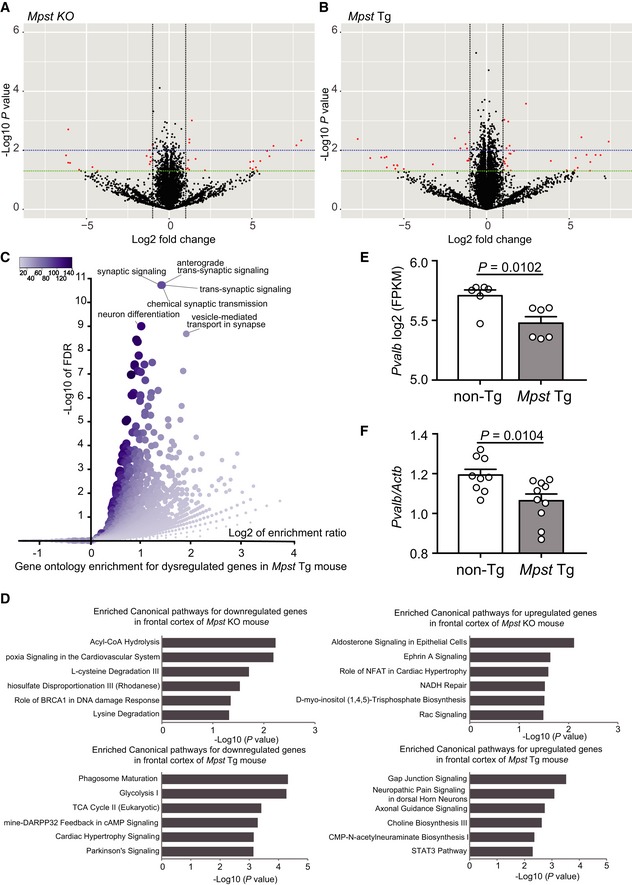
RNA‐seq and RT–PCR analyses of *Mpst*‐KO and Tg mice A, BVolcano plot of RNA‐seq analysis shows differentially expressed genes in the frontal cortex between *Mpst‐*KO mice (A) and *Mpst‐*Tg mice (B) in comparison to their wild‐type littermates; *n* = 6 in each group. Green and blue dashed lines indicate *P* value thresholds of 0.05 and 0.01, respectively. Top hits among the differentially expressed genes (*P* < 0.05 and absolute fold change > 2) are highlighted as red in the plot.CGene ontology enrichment analysis (biological process) of dysregulated genes in *Mpst‐*Tg mice visualized using a volcano plot for the enriched terms.DDifferentially expressed genes in *Mpst‐*KO mice (upper panels) and *Mpst‐*Tg mice (lower panels) (*P* < 0.05) in comparison to their WT or non‐Tg littermates were analyzed for the enriched canonical pathways.E, FGene expression of *Pvalb* in non‐Tg and *Mpst‐*Tg mice examined by RNA‐seq (*n* = 6 in each group) (E) and by qRT–PCR analysis (*n* = 10 in each group, but an outlier was excluded in WT) (F).Data information: The values represent the mean ± SEM. *P* values were calculated using unpaired two‐tailed *t*‐test (E, F).

To identify potential convergent pathways relevant for functional impairments elicited by excessive H_2_S/polysulfides production in the brain, we performed RNA‐seq analyses using *Mpst‐*KO and Tg mice. Upon disruption of *Mpst* expression in the frontal cortex, a total of 480 genes were significantly dysregulated (239 upregulated and 241 downregulated) in the KO mice compared to the WT littermates (*P* < 0.05) (Fig 8A and Appendix Table S11). Gene ontology analysis revealed that the dysregulated genes were mainly enriched in biological processes involved in mRNA splicing and processing (Appendix Table S12). Transcriptomic analysis of the frontal cortex revealed significant dysregulation of 1,136 genes (519 upregulated and 617 downregulated) in the *Mpst‐*Tg mice compared to the non‐Tg littermates (*P* < 0.05) (Fig 8B, Appendix Table S13). Interestingly, the dysregulated genes in the *Mpst‐*Tg mice were significantly enriched in the glutamatergic synaptic transmission and synaptic signaling‐related ontology terms, along with the cellular metabolic processes (Fig 8C and Appendix Table S12). The roles of synaptic dysregulation and glutamatergic signaling impairments in the pathogenesis of schizophrenia are well‐known (Uno & Coyle, 2019). Interestingly, we have also previously showed that deficits in *Fabp7*, a critical gene for regulating PPI, affect neurogenesis, NMDA signaling (Watanabe *et al*, 2007), and glial cell integrity (Ebrahimi *et al*, 2016), thus suggesting that genetic network for the PPI endophenotype overlaps with that of neurodevelopment and synaptic properties. In addition, Ingenuity Pathway Analysis™ demonstrated significant enrichment of molecular pathways, e.g., glycolysis I, TCA (tricarboxylic acid) cycle II, and dopamine‐DARPP32 feedback in cAMP signaling, for the downregulated genes (*P* < 0.05) (Fig 8D and Appendix Table S14). This evidence is consistent with energy metabolism dysfunction (Zuccoli *et al*, 2017; Sullivan *et al*, 2019) and the involvement of deficits in DARP32 systems in the pathogenesis of schizophrenia (Kunii *et al*, 2014; Wang *et al*, 2017; Zuccoli *et al*, 2017).

We specifically examined the expression levels of *Pvalb*, because impairments of parvalbumin (Pvalb, PV)‐containing inhibitory GABA (γ‐aminobutyric acid) neurons in the dorsolateral prefrontal cortex is a well‐recognized finding in schizophrenia pathophysiology (Lewis *et al*, 2005), and PV‐positive GABA neurons are energy‐demanding because of high‐frequency firing (Tremblay *et al*, 2016). The expression levels of *Pvalb* were lower in the *Mpst‐*Tg than in the non‐Tg mice in RNA‐seq analysis (Fig 8E) and in qRT–PCR analysis (Fig 8F), suggesting that deficits in the bioenergetic pathways could lead to functional impairments in PV‐positive interneurons.

**Figure 9:**
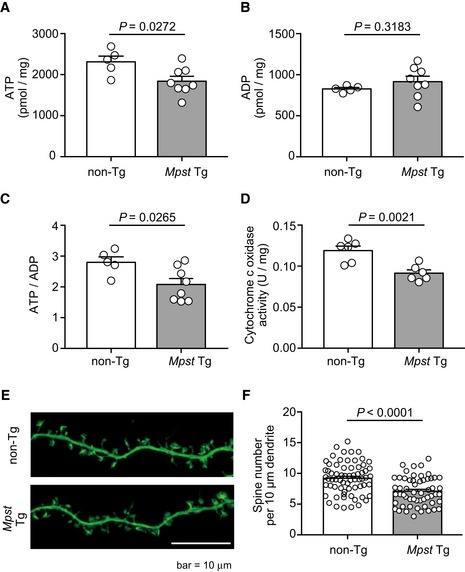
Energy metabolism and spine analyses of *Mpst‐*Tg mice A–CConcentrations of ATP and ADP, and ATP‐to‐ADP ratios in non‐Tg (*n* = 5) and *Mpst*‐Tg (*n* = 8) mice (both age of 6 weeks).DCytochrome c oxidase activity in non‐Tg (*n* = 6) and Tg (*n* = 6) mice (both age of 6 weeks).E, FFluorescence images of dendritic spines of dissociated hippocampal neurons (E16.5) of non‐Tg and *Mpst‐*Tg mice (E) and their quantification (F). *N* = 50–70 images from 10 to 15 cells. Scale bar, 10 μm.Data information: The values represent the mean ± SEM. *P* values were calculated using unpaired two‐tailed *t*‐test.

Then we analyzed the concentrations of ATP (adenosine triphosphate) and ADP (adenosine diphosphate) and the ATP‐to‐ADP ratio (Tantama & Yellen, 2014) as energy metabolism indices (Fig 9A–C). In the frontal cortex of *Mpst‐*Tg mice, the levels of ATP and ATP‐to‐ADP ratio were lower than in the non‐Tg mice (Fig 9A and C). To confirm the energetic dysregulation of *Mpst‐*Tg mice, we analyzed their mitochondrial activity *in vivo*. We focused on cytochrome c oxidase (complex IV), a component of mitochondrial respiratory chain, because excessive H_2_S is known to inhibit cytochrome c oxidase in a non‐competitive manner (Goubern *et al*, 2007; Cooper & Brown, 2008; Hancock & Whiteman, 2016). The enzymatic activity of cytochrome c oxidase in mitochondria isolated from the *Mpst‐*Tg mice was significantly lower than the non‐Tg littermates (Fig 9D).

To further examine the effect of dampened energy metabolism on neural functions, we analyzed the density of dendritic spines using primary culture of hippocampal neurons. Hippocampus is a critical region involved in the neural circuit for the regulation of PPI (Swerdlow *et al*, 2016). The spine density in dissociated hippocampal neurons was found to be diminished in the *Mpst‐*Tg mice (Fig 9E and F) compared to that of non‐Tg littermates, which is consistent with the result of a human postmortem study (Rosoklija *et al*, 2000).

As an additional possibility, we assessed protein palmitoylation. Specific palmitoyltransferases mediate the transfer of palmitoyl groups to specific cysteine residues on a variety of proteins, modulating the intracellular localization of the target proteins. Intriguingly, Pinner *et al* reported that the levels of protein *S*‐palmitoylation in most of the proteins tested were significantly reduced in postmortem brains from schizophrenia patients (Pinner *et al*, 2016). We tested whether an elevated sulfide stress condition might inhibit the protein *S*‐palmitoylation reaction, because the condition results in enhanced modification of cysteine residues (protein‐Cys‐SnH) (Kimura *et al*, 2015, 2017), thereby altering the reactivity of cysteine residues. However, we did not detect any alteration in the levels of protein *S*‐palmitoylation analyzed by means of the modified ABE (acyl‐biotin exchange) method (Forrester *et al*, 2011) in brains from *Mpst‐*Tg or KO mice (Appendix Figs S8–S11).

### DNA methylation levels are correlated with the expression levels of genes for H_2_S synthesis

There were no copy number variations for *Mpst* in the B6 and C3H genomes that can explain the differential expression (Appendix Table 15). By sequencing analysis of the 5′‐upstream region of *Mpst* (~2,600 bp), we detected distinct guanine (G) nucleotide stretches between the B6 (G_10_) and C3H genomes (G_12_) (Appendix Fig S12), which, however, does not seem to explain the differential gene expression. We tested the genetic association of *MPST* and *CBS* with schizophrenia by analyzing SNPs in more than 2,000 cases and a comparable number of controls (Bangel *et al*, 2015; Balan *et al*, 2017), but detected no association signals (Appendix Table 16). These results suggest that the differential expression of *Mpst*/*MPST* and *CBS* in mouse strains/disease brains is not due to inherent genetic variations. Therefore, we examined the epigenetic mechanism underlying the changes in expression.

We examined the DNA methylation status of the relevant genes. There are two CpG islands at the *Mpst* locus in mice, one in the promoter region (“CpG28”) and the other around exon 2 (“CpG64”) (Appendix Fig S13). In “CpG64”, the CpG_31, CpG_42.43.44 (examined by probing the interval #1), and CpG_15 (examined by probing the interval #7) sites were highly methylated in the C3H genome compared to the B6 genome (Appendix Fig S14). The mean methylation levels of the three sites were positively correlated with the expression levels of *Mpst* (Fig 5C).

For the human genes, the *MPST* locus has two CpG islands, as observed in mice (Appendix Fig S15). There were multiple differentially methylated cytosine nucleotides between schizophrenia and control postmortem brain samples, but methylation levels at those sites did not show any significant correlation with the expression levels of *MPST*. In the *CBS* genomic region, there is one CpG island in the intron 1 interval (Appendix Fig S16). In the postmortem brain samples, the average methylation levels in the 3′ portion of “CpG227” (examined by probing the interval #17) were higher in the schizophrenia samples than in the controls (Appendix Fig S17) and exhibited a positive correlation with the expression levels of *CBS* (Fig 5D). In neurosphere samples, the 3′ portion of “CpG227” (examined by probing the interval #17) from schizophrenia with 22q11.2 deletion also showed higher methylation levels than that from controls (Appendix Fig S18).

These results suggest that least elevated expression levels of *Mpst* in C3H brains and *CBS* in the schizophrenia postmortem brain samples and iPSCs‐derived neurospheres could be elicited by altered DNA methylation levels in the CpG islands in the target gene regions.

### Inflammatory/oxidative stress in the early brain developmental stage results in upregulated H_2_S/polysulfides production

**Figure 10:**
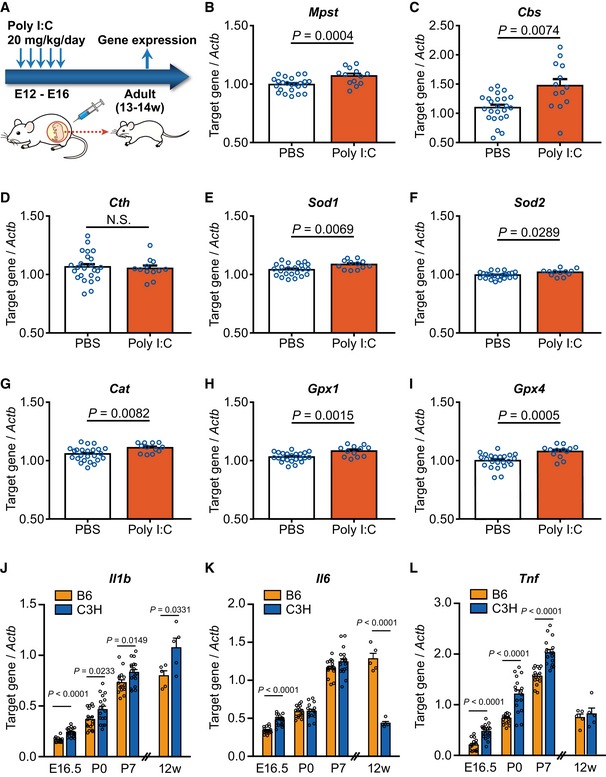
Gene expression in the maternal immune activation mouse model and in multiple developmental stages ASchematic illustration of the experimental design of maternal immune activation mouse. B6 mice were used, and gene expression was examined in the frontal cortex.B–ITarget gene expression levels were relative to that of *Actb*. PBS‐injected mice, *n* = 25; poly‐I:C‐injected mice, *n* = 13.J–LComparison of expression levels of typical inflammatory genes *Il1b* (J), *Il6* (K), and *Tnf* (L) in four developmental points of brains between B6 and C3H mice. “12W” samples were analyzed separately from the others. Whole cortex for E16.5 (*n* = 15–20), P0 (*n* = 17), and P7 (*n* = 17–18), and frontal cortex for 12W (*n* = 5) were used for analysis. E16.5, embryonic day 16.5; P0, at birth; P7, postnatal day 7; 12W, 12 weeks of age.Data information: The values represent the mean ± SEM. *P* values were calculated using unpaired two‐tailed *t*‐test.

H_2_S plays a protective role against oxidative and inflammatory stresses (Kwak *et al*, 2003; Niu *et al*, 2015). Oxidative stress depletes GSH and cysteine, which in turn enhances the activity of H_2_S/polysulfides‐producing enzymes to stimulate the synthesis of H_2_S, cysteine, and GSH, leading to the protection of cells from oxidative stress (Appendix Fig S19) (Kimura, 2010; Niu *et al*, 2015). H_2_S also attenuates the phosphorylation of mitogen‐activated protein kinases as well as the activation of NF‐κB and caspase‐3 and suppresses Bcl‐2 expression under inflammatory conditions (Tripatara *et al*, 2008). Based on the above, when oxidative insults occur in an early neurodevelopmental stage, the expression of both typical antioxidant and H_2_S/polysulfides synthesis genes could be programmed to be maintained at higher levels via epigenetic mechanisms. To examine this hypothesis, we used a mouse model of maternal polyriboinosinic‐polyribocytidilic acid (poly‐I:C) injection (maternal immune activation model) (Meyer & Feldon, 2012; Giovanoli *et al*, 2013; Bundo *et al*, 2014), which is known to perturb early neural development via inflammatory/oxidative insults. Pregnant mice (B6) received five intraperitoneal injections of poly‐I:C during embryonic days 12–16 (E12–E16), and gene expression in the cortex was analyzed at 13–14 weeks of age (Fig 10A). In this protocol, a “schizophrenia endophenotype of increased L1 copy number” was observed (Bundo *et al*, 2014). Interestingly, the expression levels of *Mpst* and *Cbs* were elevated in the adult brain of this model, albeit *Cth* expression was unchanged (Fig 10B–D).

**Figure EV4:**
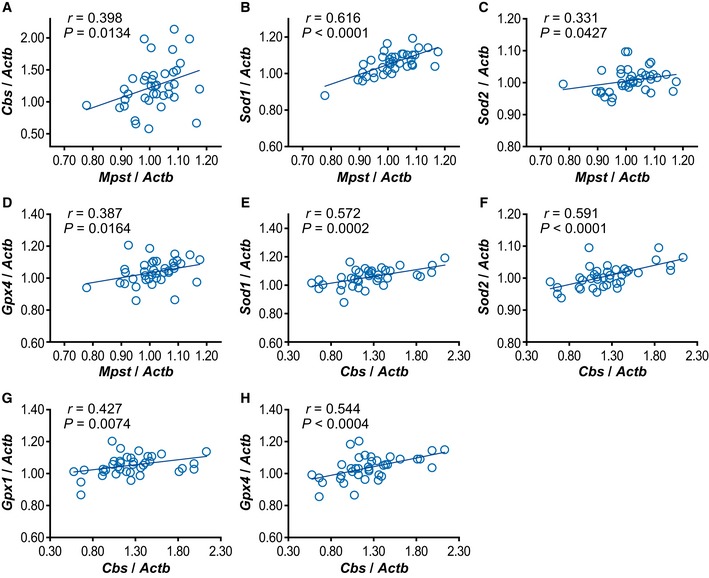
Correlation between expression levels of *Mpst* and *Cbs* (A) and between *Mpst*/*Cbs* and antioxidant genes (B–H) in poly‐I:C administered mice A–HCorrelation was analyzed using frontal cortex of 38 mice. Each gene expression level was normalized to that of *Actb*.Data information: Statistical evaluations were performed using Spearman's rank correlation test.

As representative antioxidant genes, we examined the expression of *Sod1* and *Sod2* (encoding superoxide dismutase), *Cat* (encoding catalase), and *Gpx1* and Gpx*4* (encoding glutathione peroxidase) (Smaga *et al*, 2015). The expression levels of all these genes were significantly upregulated in the poly‐I:C administered mice compared to the controls (Fig 10E–I), although the fold changes were small (for poly‐I:C‐to‐control, *Sod1* = 1.043, *Sod2* = 1.024, *Cat* = 1.051, *Gpx*1 = 1.050, *Gpx4* = 1.079), suggesting small effect sizes of individual expressional changes. The relative expression levels of the two genes encoding H_2_S/polysulfides synthesis were compared in the following pairwise comparisons: *Mpst* versus *Cbs* (Fig EV4A), *Mpst* versus individual antioxidant genes (Fig EV4B–D), and *Cbs* versus individual antioxidant genes (Fig EV4E–H). Except for *Mpst* versus *Gpx1*, all the pairwise comparisons exhibited positive correlation.

Conforming to the above “inflammatory/oxidative stress in the early brain developmental stage” theory, inflammatory genes *Il1b*,* Il6,* and *Tnf* were upregulated in C3H brain compared to B6 at some point(s) during E (embryonic day) 16. 5 to P (postnatal day) 7 without downregulation in any periods, though downregulation of *Il6* was seen at an adult stage (12 weeks old) (Fig 10J–L). These developmental events could lead to an elevated H_2_S/polysulfides production state in C3H mice compared to B6.

**Figure EV5:**
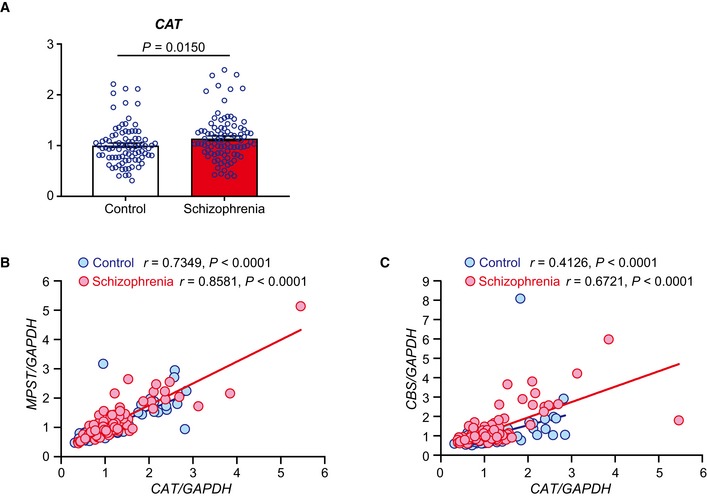
Analysis of antioxidant gene *CAT* and genes for H_2_S‐synthesizing enzymes in human postmortem brain AExpression levels of *CAT* were measured in the frontal cortex (BA8) from control (*n* = 93) and schizophrenia (*n* = 95) (see Appendix Table S7). The values represent the mean ± SEM.B, CCorrelation between expression levels of *CAT* and *MPST*/*CBS*. The same samples as analyzed in (A) were used. Each gene expression level was normalized to that of *GAPDH*.Data information: Statistical evaluations were performed using Mann–Whitney *U* test (A) and Spearman's rank correlation test (B, C).

In human brains, *CAT* was upregulated in schizophrenia samples compared to the controls (Fig EV5A), and the expression level of this gene was positively correlated with that of *MPST* and *CBS* in both the schizophrenia and control samples (Fig EV5B and C). The *IL1B*/*Il1b*,* IL6*/*Il6,* or *TNF*/*Tnf* was not significantly upregulated in the schizophrenia postmortem brain sample (Toyoshima *et al*, 2016) and in the poly‐I:C model mice (Appendix Fig S20). These results support our hypothesis that upregulated H_2_S/polysulfides production in adulthood can stem from oxidative/inflammatory insults in the neurodevelopmental stage, occurring in a concerted manner with the elevation of antioxidant gene expression and via an epigenetic mechanism.

## Discussion

Prior studies have repeatedly reported physiologically necessary/beneficial and neuroprotective roles of H_2_S/polysulfides as gasotransmitters or neuromodulators (Szabo, 2007; Kimura, 2015; Wallace & Wang, 2015), including those in Huntington's disease (Paul *et al*, 2014). In contrast, in this study, we started by analyzing the molecular underpinnings of the sensorimotor gating function against acoustic stimuli in mice and, as a consequence, provide the first evidence that excess H_2_S/polysulfides production could underlie the pathophysiology of, at least, a subset of schizophrenia. Although we revealed the elevated sulfide stress in schizophrenia pathophysiology, in the light of Mpst from our proteomics study, upregulation of a combination of the three genes encoding H_2_S/polysulfides‐synthesizing enzymes, namely, *Mpst* (*MPST*), *Cbs* (*CBS*), and *Cth* (*CTH*), was observed in mouse models and human schizophrenia samples. Regarding such combination, we speculate that depending on the tissue‐specific variability in the expression levels of these genes, a gene network including predominantly expressed one in the relevant tissue (see Fig EV3) may leverage maximum control of H_2_S/polysulfides production. Hydrogen sulfide is a highly bioactive agent in organisms, thus even small changes in its production levels should have serious consequences in life. Therefore, we believe that even small changes detected in this study can be biologically meaningful. We further demonstrated that altered expression of these genes could be, at least in part, primed by changes in genomic DNA methylation markers, although their direct link remains to be proved. We demonstrated that such epigenetic changes can be traced back to inflammatory/oxidative insults in the early brain developmental stage by analyzing the poly‐I:C model, which is consistent with, and a novel aspect of, the “neurodevelopmental theory” of schizophrenia pathogenesis. We reason that upregulation of the H_2_S/polysulfides synthesis genes and representative antioxidant genes can be orchestrated against an oxidative event in a complementary manner because H_2_S/polysulfides can mitigate oxidative stress (Kimura, 2010; Niu *et al*, 2015; Wallace & Wang, 2015). Notably, endogenous H_2_S also enhances the activities of catalase and superoxide dismutase in bacteria (Shatalin *et al*, 2011).

To determine the functional consequence of excess H_2_S/polysulfides production under disease conditions, we performed RNA‐seq analysis using *Mpst‐*KO and Tg mice and detected the DARPP32 and energy metabolism (glycolysis and TCA cycle) systems among the top canonical pathways, dysregulation of which has been documented in schizophrenia (Kunii *et al*, 2014; Wang *et al*, 2017; Zuccoli *et al*, 2017). In particular, several lines of investigation have suggested a reduced capacity of the TCA cycle in schizophrenia (Bubber *et al*, 2011; Paredes *et al*, 2014). Concordant with those reports, our *Mpst‐*Tg mice showed reduced ATP concentration and decreased ATP‐to‐ADP ratio, and deficits in cytochrome c oxidase (complex IV) activity, compared to the non‐Tg animals. These dampened bioenergetic processes may have led to decreased *Pvalb* expression, a functional marker of PV‐positive interneurons, and diminished spine density. The brain is deemed to be susceptible to H_2_S, because in the brain, the activity of SQOR (sulfide quinone oxidoreductase), a H_2_S‐metabolising enzyme, was lower than in peripheral tissues (Linden *et al*, 2012). The detailed mechanistic links between these issues, including *in vivo* stoichiometry of H_2_S/H_2_S_n_ on the suppression of complex IV activity (Szabo *et al*, 2014; Modis *et al*, 2016), should be clarified in future.

There are several cases in which excess H_2_S production results in cognitive impairments. The *CBS* gene is encoded on chromosome 21 (21q22.3), which is associated with trisomy in Down syndrome (DS). Overexpression of CBS may cause developmental abnormalities in cognition in children with DS (Ichinohe *et al*, 2005). Barbaux *et al* (2000) reported that the *CBS* 844ins68 allele was significantly underrepresented in children with high IQs. The *CBS* 844ins68 allele carries a 68‐bp insertion consisting of a duplicated intron 7/exon 8 boundary (Sebastio *et al*, 1995) and exhibits enhanced activity compared to the WT allele (Barbaux *et al*, 2000). Recent evidence has showed that increased copy number of *Cbs* elicits memory‐related cognitive deficits in DS mouse models, by perturbing synaptic activity, possibly through H_2_S signaling (Marechal *et al*, 2019). These results suggest that excess H_2_S induced by enhanced CBS/Cbs activity may contribute to at least some aspect(s) of cognitive impairments manifested in schizophrenia (Rajagopal *et al*, 2014). Similarly, ethylmalonic encephalopathy (EE; OMIM #602473), an autosomal recessive mitochondrial disease associated with progressive neurological failure, is caused by mutations in *ETHE1* (Burlina *et al*, 1991; Mineri *et al*, 2008). *ETHE1* encodes a ubiquitous mitochondrial sulfur dioxygenase (SDO) (Tiranti *et al*, 2009) that eliminates H_2_S (Hildebrandt & Grieshaber, 2008).

Because H_2_S is distributed systemically, some somatic diseases are also affected by H_2_S. Ulcerative colitis is an inflammatory bowel disease, and H_2_S overproduction contributes to the etiology of this disease (Roediger *et al*, 1997). On the other hand, H_2_S is present in joints and acts as a proinflammatory mediator (Muniraj *et al*, 2017); therefore, rheumatic diseases might be associated with decreased production of H_2_S. Comorbidity has been reported between ulcerative colitis and schizophrenia (Cucino & Sonnenberg, 2001), whereas rheumatoid arthritis occurs at a relatively low frequency in schizophrenia patients (Leucht *et al*, 2007).

In schizophrenia, biomarkers are of cardinal importance for early intervention and improved prognoses (Morrison *et al*, 2012; Fusar‐Poli *et al*, 2013). Biomarkers can be also used to categorize patients and determine optimal therapeutics. Our postmortem study suggested a positive correlation between symptomatic severity and “sulfide stress”, and in living patients the present results highlight the potential benefit of examining *MPST* expression levels in scalp hair follicles. The use of scalp hair follicles is advantageous because of the convenience and noninvasiveness of sampling (Maekawa *et al*, 2015).

Meanwhile, enhanced inflammatory conditions in schizophrenia are repeatedly reported, in particular around onset and in active phase, which may be state‐dependent (temporal) (Smaga *et al*, 2015; Koga *et al*, 2016; Watkins & Andrews, 2016). Excess H_2_S/polysulfides production may be a more basic and persistent phenomenon like, “continuous bass”, in the realm of “reductive response” which has not been explicitly addressed in the previous studies. The precise contribution of these two biological reactions to disease process remains elusive.

In summary, although H_2_S/polysulfides are necessary for maintenance of normal cellular functions, an excessive amount of H_2_S/polysulfides may impair brain functions; we propose to name this phenomenon “sulfide stress”, which could be a novel form of “reductive stress” (Perez‐Torres *et al*, 2017). One of the potential origins of “sulfide stress” could be oxidative/inflammatory insults in neurodevelopment and is epigenetic priming as a mechanism, and its consequence could include damped energy metabolism. Elucidation of the precise role of “sulfide stress” in schizophrenia and other mental disorders will facilitate the establishment of a novel paradigm for drug development.

## Materials and Methods

### Experimental animals

For the proteomics study, B6 (C57BL6/NCrj) and C3H (C3H/HeNCrj) mice were purchased from Japan SLC (Shizuoka, Japan). For the other studies, B6 (C57BL/6NCrl) and C3H (C3H/HeNCrl) mouse strains were obtained from Japan's Charles River Laboratories (Kanagawa, Japan). The change in substrains was due to the introduction of the International Genetic Standardization Program (https://www.crj.co.jp/cms/cmsrs/pdf/company/rm_rm_r_igs.pdf) after the proteomics study. The animals were bred in our facilities for 2 weeks before the experiments, housed by strain in groups of four, using standard cages in a temperature‐ and humidity‐controlled room with a 12‐h light/dark cycle (lights on at 0800). The animals had free access to standard laboratory chow and tap water. The experimental procedures conformed to NIH guidelines for animal welfare and were approved by the Animal Ethics Committees of all the relevant institutes. The mouse studies contained no randomization procedures for group allocation and were conducted in an open‐label format. Only male animals were used for all the experiments except for the data for Fig 10J–L, samples for E16.5, P0, and P7.

### Sample preparation for proteomics analysis

Eight‐week‐old mice were decapitated, and the bilateral prefrontal cortex (AP > +2 mm from the bregma) of each brain was dissected and frozen immediately. The spleen was also dissected, rinsed in ice‐cold phosphate‐buffered saline, homogenized on a stainless steel fine mesh net, and filtered using Eagle's MEM supplemented with 10% FBS. The filtered cells were centrifuged, and the supernatants were removed. Red blood cells were ruptured in an NH_4_Cl solution and washed with Eagle's MEM, and then, residual lymphocytes were collected. All lymphocyte and brain samples were stored at −80°C until protein preparation. Tissues were homogenized and sonicated in ice‐cold lysis buffer (20 mM Tris, 7 M urea, 2 M thiourea, 4% (w/v) CHAPS, 10 mM DTT, 1 mM EDTA) containing protease and a phosphatase inhibitor cocktail (P8340, P2850, P5726; Sigma, St Louis, MO, USA). After centrifugation of the lysate (1.7 × 104 *g*), the supernatant was recovered and assayed for protein concentration using the Bradford method.

### 2D‐DIGE analysis of brain and splenic lymphocyte samples from mice: labeling

The experimental design for the 2D‐DIGE analysis in this study is shown in Appendix Table S1. Mixtures from each sample (100 mg of protein) derived from brain or lymphocytic tissue were pooled as an internal standard. A 100‐μg aliquot of protein from each sample was labeled with Cy3 for B6 mice, Cy5 for C3H mice, and Cy2 for the internal standard and left on ice for 30 min in the dark. The labeling reaction was quenched with 2 μl of 10 mM lysine. Each pair of labeled B6‐ and C3H‐derived samples was then combined with the labeled internal standard and added to an equal volume of 2× sample buffer [9 M urea, 4% (w/v) CHAPS, 2.4% (v/v) DeStreak reagent (GE Healthcare, Chicago, IL, USA), 1% (v/v) IPG buffer 3–10 (GE Healthcare)] and diluted with rehydration buffer [9 M urea, 2% CHAPS, 1.2% (v/v) DeStreak reagent, 0.5% (v/v) IPG buffer 3–10], to a final volume of 250 μl, prior to loading onto the 2D gel.

### 2D gel electrophoresis

One Immobiline DryStrip [pH 3–10, 13 cm (GE Healthcare)] was passively rehydrated with each combined sample for more than 10 h at room temperature. For the first dimension of electrophoresis, the rehydrated strips were focused using Ettan IPGphor II (GE Healthcare) for a total of 82,000 Vh. The strips were then reduced and alkylated according to the manufacturer's protocol. For the second dimension of gel electrophoresis, the strips were loaded onto 12% polyacrylamide gels in a Hoefer SE 600 Chroma system, and proteins were resolved at 15 mA/gel for 7 min and then at 30 mA/gel for the duration of the run.

### Data analysis for 2D gel electrophoresis

Separated gels were scanned using a Typhoon 9400 digital scanner (GE Healthcare), and images were analyzed by differential analysis software (DeCyder, version 5.0, GE Healthcare). Spots were recognized using a Differential In‐gel Analysis (DIA) module, with the estimated number of spots for each co‐detection procedure set to 5,000 and filtered using default settings. Intergel matching and statistical analyses were performed using the Biological Variable Analysis (BVA) module.

### Protein identification: SYPRO ruby and silver staining

Three‐hundred‐microgram aliquots of protein from each tissue sample were diluted in rehydration buffer to a final volume of 250 μl and separated by 2D gel electrophoresis as described above. The gels were then stained with SYPRO Ruby Protein Gel Stain (Molecular Probes) according to the manufacturer's protocol. The resulting fluorescent images were scanned. Protein spots were excised and destained with 25 mM (NH_4_)HCO_3_/50% (v/v) acetonitrile (ACN), followed by an additional wash in 100% ACN using Xcise (Shimadzu), and then dried to enable in‐gel digestion.

For large‐scale analysis, 500 μg of protein was focused using a 24‐cm Immobiline DryStrip pH 3–7 (GE Healthcare) for a total of 160,000 Vh. In the second dimension, the run was performed using an Ettan DALT Six (GE Healthcare). Gel spots were stained with a Plus One Silver Stain Kit according to the manufacturer's instructions and excised with an Ettan Spot Picker (GE Healthcare). Gels were then destained in buffer [15 mM potassium hexacyanoferrate (III), 50 mM sodium thiosulfate], washed in 100% ACN, and dried.

### In‐gel digestion and MALDI‐TOF mass spectrometry (MS)

In‐gel digestion was performed using 3 μg/ml trypsin in 50 mM (NH_4_)HCO_3_ with 0.1% (w/v) N‐octylglucoside at 37°C for 16 h. Digested peptides were extracted with 5% (v/v) formic acid/50% ACN, dried, diluted in 0.1% (v/v) trifluoroacetic acid (TFA), and then desalted and concentrated in a PerfectPure C‐18 tip (Eppendorf). Peptides were eluted onto a MALDI target plate with matrix solution (5 mg/ml α‐cyano‐4‐hydroxycinnamic acid (CHCA), 0.1% TFA, 50% ACN, 500 pmol of bradykinin, 400 pmol of ACTH) and dried at room temperature. The peptide masses were then determined by MALDI‐TOF MS. Mass spectra were obtained using an AXIMA CFR plus (Shimadzu, Kyoto, Japan) in positive ion reflectron mode, and the mass axis was calibrated using bradykinin and ACTH. Raw data were processed using Kompact (ver. 2.4, Shimadzu), and the peak list was generated with default parameters as follows: minimum mass: 600, maximum mass: 3,500, minimum isotopes: 2, maximum intensity variation: 50, overlapping distributions: on, and minimum peak percent: 30. The identified proteins were verified by manual comparisons of the computer‐generated fragment ion series of the predicted peptide with experimental MS data. The mass spectrum for each spot was analyzed by PMF using MASCOT™ software (ver. 2.2.1, Matrix Science) (http://www.matrixscience.com) and used for PMF analysis against the UniProt or NCBI nonredundant protein databases. Database searching was performed using the following settings: (i) *Mus musculus*, (ii) trypsin digestion with up to 1 missed cleavage, (iii) ± 0.5 Da error, (iv) cysteine carbamidomethylation as a fixed modification, and methionine oxidation as a variable modification. After excluding unmatched peptides to avoid contaminations of overlapping spots, identities were assigned when score values were returned with *P* value < 0.05 for a false positive and were confirmed by manual inspection of the spectra. Each protein was confirmed with 2D Western blot analyses described below.

### 1D and 2D Western blot analyses: antibodies

We generated rabbit polyclonal antibodies against the N‐terminal (DASWYLPKLGRDARREF) and C‐terminal (YMRAQPEHIISEGRGKT) regions of mouse Mpst [designated as anti‐Mpst #1 and anti‐Mpst #2, respectively]. For 1D Western blot analyses, the anti‐Mpst #1 and #2 and anti‐α‐tubulin (T5168, Sigma) antibodies were diluted in 1:100, 1:1,000, and 1:20,000, respectively. The anti‐Mpst #1 (dilution 1:500) and commercially sourced antibodies, which included anti‐GRP 75 (Sigma; dilution 1:2,000) for Hspa9 and anti‐NPM1 (ProteinTech Group, Rosemont, IL, USA; dilution 1:1,000) for Npm1, were used in 2D Western blot analyses of both frontal cortex and lymphocytes. Anti‐peroxiredoxin‐6 (Abcam, Cambridge, England) for Prdx6 was diluted in 1:1,000 (frontal cortex) and 1:500 (lymphocytes), and anti‐NME2 (Abgent, San Diego, CA, USA) for Nme2 was diluted in 1:100 (frontal cortex) and 1:50 (lymphocyte) (expression levels of Prdx6 and Nme2 in lymphocytes were low).

### Western blotting

For 2D Western blotting, 300 μg of protein was separated by 2D gel electrophoresis using a 13‐cm Immobiline DryStrip pH 3–10 or pH 4–7 (GE Healthcare) using the same procedure as described above, except for the rehydration buffer [9 M urea, 2% CHAPS, 0.28% (w/v) DTT, 0.5% (v/v) IPG buffer 4–7] in the pH 4–7 strip. Proteins were transferred to PVDF membranes by semidry blotting, and the membranes were stained with Deep Purple (GE Healthcare) according to the manufacturer's protocol. Fluorescent signals were scanned with Typhoon 9400, after which the membranes were soaked in transfer buffer prior to antibody binding. Membranes were incubated in blocking solution containing 5% skimmed milk in TBS with 0.05% Tween 20 (TBS‐T) for 1 h and then incubated overnight at 4°C with primary antibodies in blocking solution. The membranes were then washed in TBS‐T and probed with horseradish peroxidase (HRP)‐conjugated anti‐rabbit or anti‐mouse IgG antibodies (Sigma). Signals were detected using Western Blotting Luminol Reagent (Santa Cruz Biotechnology, Dallas, Texas, USA). The fluorescent signals on the 2D Western blots were detected using a Typhoon 9400. Chemiluminescent signals were visualized using a Typhoon 9400 or LAS‐3000 (Fuji Film, Tokyo, Japan). Fluorescent and chemiluminescent images were merged using FluorSep software (version 2.2, GE Healthcare). For standard 1D Western blotting, 20‐μg protein samples were loaded onto 12% SDS–polyacrylamide gels and separated by electrophoresis. Proteins were transferred to PVDF membranes by semidry blotting, and the membranes were incubated overnight at 4°C with primary antibodies in blocking solution. The membranes were then washed in TBS‐T and probed with horseradish peroxidase (HRP)‐conjugated anti‐rabbit (Sigma, cat no. 12‐348) or anti‐mouse IgG antibodies (Sigma, cat no. 12‐349).

### DNA sequencing and database searching

Genomic DNA samples were extracted using a standard phenol/chloroform method from mouse tails and amplified using fluorescently labeled forward and reverse primers and ExTaq polymerase (TaKaRa Bio, Shiga, Japan). Sequencing was performed using the BigDye Terminator Cycle Sequencing FS Ready Reaction Kit ver. 3.1 (Applied Biosystems, Foster City, CA, USA) and an ABI PRISM 3730 genetic analyzer (Applied Biosystems). Genomic sequences and SNPs were examined using the UCSC Genome Bioinformatics online database (http://genome.ucsc.edu/cgi-bin/hgGateway: version Dec. 2011).

### Measurement of H_2_S, acid‐labile and bound sulfur

After 10‐ to 12‐week‐old mice were decapitated, brains were quickly removed and sagittally divided into two groups. Using one hemisphere, H_2_S levels were measured according to a previously reported method (Kimura *et al*, 2015). Using the other hemisphere, acid‐labile and bound sulfur levels were measured according to methods described elsewhere (Ishigami *et al*, 2009; Shibuya *et al*, 2009).

### Generation of *Mpst* knockout (KO) mice

We generated *Mpst*‐deficient mice by genome editing using the CRISPR/Cas9 nickase in the genetic background of the C3H strain (Appendix Fig S6). First, the sgRNAs [*Mpst*‐upstream (target sequence: 5′‐GGAGAGACAAGCCTGCATTCAGG‐3′) at intron 1 and *Mpst*‐downstream (target sequence: 5′‐GTGGGTGGCGGAGGCTCTGAAGG‐3′) at exon 2, where the underlined bases indicate the PAM sequences] were synthesized *in vitro* (T7 gRNA Smart Nuclease Synthesis Kit, System Biosciences, Mountain View, CA, USA) following the manufacturer's instructions. C3H zygotes were microinjected with the cocktail [5 ng/ml Cas9 nickase mRNA (System Biosciences) and 5 ng/ml each of the two sgRNAs]. The injected zygotes were transplanted into the uteri of pseudopregnant dams, and the targeted region of the *Mpst* gene from the resultant pups, which were obtained by cesarean section, was examined by direct sequencing of PCR products amplified from the template DNAs extracted from the tail with primer set A (forward: 5′‐ CTGTTTTGCAGCCCCGCAATCT‐3′, reverse: 5′‐TAGGTACCAGGACGCGTCCAGTAA‐3′). Mutated alleles of the promising founders were further analyzed by sequencing of the PCR products that were subcloned into pCR2.0 (Invitrogen, Grand Island, NY, USA). When the selected founder (#461) carrying a 4‐bp deletion downstream of the start codon in exon 2 of *Mpst* reached sexual maturity, *in vitro* fertilization was performed with C3H strain‐derived oocytes to obtain heterozygous mice harboring the mutated allele. The heterozygous males and females, at sexual maturity, were intercrossed to produce homozygotes and control littermates. Routine genotyping of the mice was conducted on an ABI 3130xI genetic analyzer (Life Technologies, Carlsbad, CA, USA) followed by genomic PCR using primer set A, which produced 152‐ and 156‐bp fragments from the deleted allele and unedited allele, respectively. To confirm loss of the Mpst protein in homozygotes, the brain tissues of the animals were subjected to Western blot analysis with an anti‐Mpst antibody (Nagahara *et al*, 1998).

### Generation of *Mpst‐*transgenic (Tg) mice

*Mpst‐*Tg mice were generated as described elsewhere (Ohnishi *et al*, 2010) with minor modifications, using the pCAGGS backbone harboring the CAG promoter (Niwa *et al*, 1991) (Appendix Fig S7) in the genetic background of B6. The HA‐tagged *Mpst* ORF derived from B6 was cloned into the *Xho*I site of pCAGGS, and the resultant plasmid was microinjected into B6 zygotes after linearization with *Sca* I. The injected zygotes were transplanted into the uteri of pseudopregnant ICR dams. As a primary screening, genomic DNAs from the tail of the pups were subjected to genomic PCR to identify animals with the transgene using the three primer pairs covering different parts of the construct (pair A: *pCAGGS_FW2*: 5′‐TAACCATGTTCATGCCTTCT‐3′ and *Mpst_Rv1*: 5′‐TTTACTGAGCCAGGGATGT‐3′, pair B: *Mpst_Fw1*: 5′‐ATCTACGACGACAGTGACCA‐3′and *β‐globinA_Rv1*: 5′‐GTCGAGGGATCTCCATAAGA‐3′, and pair C: *CAG_Fw4*: 5′‐ACCTGGGTCGACATTGATTA‐3′ and *CAG_Rv2*: 5′‐AACATGGTTAGCAGAGGCTC‐3′). As a secondary screening, proteins extracted from the tail were subjected to SDS–PAGE and Western blotting with anti‐*Mpst* and anti‐HA antibodies to identify HA‐Mpst‐positive animals. Here, notably, the CAG promoter is known to be ubiquitously active among various tissues, including the tail and brain, enabling us to expect high‐level expression in the brains of animals that exhibited transgene expression in the tail. Since founder #737 showed the highest expression of the transgene among the candidates, the offspring animals of this founder were generated. Transgene expression was examined by Western blot analysis.

To identify the integration site of the transgene, next‐generation sequencing of genomic DNA obtained from the tail was performed by TaKaRa Bio. The raw sequence data were analyzed using multiple tools, including BWA (http://biobwa.sourceforge.net/), Picard (http://picard.sourceforge.net/), BEDTools (http://code.google.com/p/bedtools/), BreakDancer (http://breakdancer.sourceforge.net/), and PinDel (http://trac.nbic.nl/pindel/). Routine genotyping of mice after identification of the integration site was conducted by multiplex PCR using a primer set (*Chr_BP1‐F*: 5′‐GATGGGCCACACTCTTAGAGCCTCTG‐3′, *Insert_BP1‐R*: 5′‐AGCGCAGAAGTGGTCCTGCAACTT‐3′ and Del_BP1‐R: 5′‐GGAGTTTGTTTGGGTCTTTTGTTGCTTG‐3′) that produced 503‐ and 411‐bp fragments from the Tg‐integrated and normal alleles, respectively.

### Behavioral analysis

PPI scores and ASR against acoustic stimuli in mice were measured according to previously published methods (Shimamoto *et al*, 2014). Sodium hydrosulfide (NaHS) was purchased from Nacalai Tesque (Kyoto, Japan). This compound was dissolved in saline and administered to a mouse intraperitoneally at a dose of 1 mg/kg or 10 mg/kg body weight once a day. Control mice were administered saline.

### Real‐time quantitative RT–PCR and digital RT–PCR

Single‐stranded cDNA for each total RNA sample was synthesized using SuperScript III RT (Invitrogen), oligo(dT), and random hexamers (Maekawa *et al*, 2015). Real‐time quantitative PCR analysis was conducted using an ABI7900HT Fast real‐time PCR system (Applied Biosystems). The TaqMan probes used were TaqMan™ Gene Expression Assay products (Applied Biosystems). All real‐time quantitative PCR data were captured using SDS v2.4 (Applied Biosystems). The ratio of the relative concentration of the target molecule to that of the *GAPDH* gene (target molecule/*GAPDH* gene) was calculated. All reactions were performed in triplicate based on a standard curve method. Outliers (more or less than mean ± 2SD) were excluded.

Absolute quantitative analysis of mRNAs was conducted using the QuantStudio™ 3D digital PCR system (ThermoFisher, Waltham, MA, USA). The TaqMan probes used were TaqMan™ Gene Expression Assay products (Applied Biosystems). All absolute quantitative data were captured using QuantStudio™ 3D AnalysisSuite™ software (ThermoFisher).

### Human samples

A set of RNA samples derived from BA8 was obtained from the Victorian Brain Bank Network (http://www.mhri.edu.au/brain-bank) (1^st^ set). Demographic data of the BA8 samples are described in Appendix Table S7. Another set of postmortem brain tissues (BA17) from schizophrenia and age‐matched controls were obtained from the Postmortem Brain Bank of Fukushima for Psychiatric Research and Brain Research Institute, Niigata University, Japan (in total *n* = 22 for schizophrenia and *n* = 14 for control) (2^nd^ set) (Ohnishi *et al*, 2019) (Appendix Table S8).

Characteristics of the human iPS cells and neurosphere samples analyzed are described elsewhere (Bundo *et al*, 2014; Maekawa *et al*, 2015; Toyoshima *et al*, 2016). Peripheral whole‐blood samples were collected, and total RNA was extracted using the RiboPure™‐Blood Kit (Ambion, Grand Island, NY, USA). MPST levels in plasma were measured using a human ELISA kit for 3‐mercaptopyryvate sulfurtransferase (MST) (Wuhan USCN Business, Hubei, China). Subjects consisted of 44 subjects with schizophrenia and 56 control subjects, all from the Tokyo area of Japan. Diagnoses were made in accordance with the DSM‐IV criteria. Demographic data are shown in Appendix Table S8.

Preparation of scalp hair follicle samples and the method used for RNA extraction from those samples are described elsewhere (Maekawa *et al*, 2015). We collected 10–12 hair follicles from an individual, with RNA yield per one hair follicle being 58.09 ± 27.61 ng (mean ± SD). We did not amplify RNA but pre‐amplified cDNA of all the target genes before quantification, using TaqMan PreAmp Master Mix (ThermoFisher). Demographic data are shown in Appendix Table S9.

The human studies, including the use of iPS cells, conformed to the principles set out in the WMA Declaration of Helsinki and the NIH Belmont Report and were approved by the Ethics Committees of all relevant institutes, and all the participants provided written informed consent to participate in the study.

### Immunohistochemistry and *in situ* hybridization of MPST/*MPST* in hair follicles

Analysis of MPST protein expression in scalp hair follicle samples was performed as previously reported (Maekawa *et al*, 2015, 2019). Primary polyclonal rabbit antibody was used for MPST detection (Santa Cruz Biotechnology (Dallas, TX, USA); dilution 1:100). The secondary antibodies used were Alexa Fluor 488‐ or 594‐labeled goat anti‐rabbit IgG or goat anti‐guinea pig IgG (Life Technologies; dilution, 1:400).

Sequences for *in situ* probes were amplified by PCR using the appropriate primers and cloned into pGEM‐T (Promega, Fitchburg, WI, USA). Digoxigenin (DIG)‐labeled RNA probes were prepared with a template plasmid and the DIG RNA Labeling Kit (Roche, Basel, Switzerland). After drying, the sections were fixed for 10 min in 4% paraformaldehyde in PBS at room temperature. The sections were rinsed with PBS and incubated with 0.5 μg/ml proteinase K (in 10 mM Tris–HCl, pH 7.4, 1 mM EDTA) for 5 min at 37°C. Fixing was repeated with 4% paraformaldehyde in PBS for 10 min, followed by rinsing with PBS. Probes were diluted (1:200) with hybridization buffer [5× SSC, 50% formamide, 1% (v/v) SDS, 50 μg/ml heparin, 50 μg/ml tRNA], and 300 μl of each sample was applied to a slide. After 16 h of incubation at 65°C, the sections were washed, first with MABT buffer [100 mM maleic acid, pH 7.5, 150 mM NaCl and 0.3% (v/v) Tween 20] twice for 5 min, then with washing buffer [1× SSC, 50% formamide, 0.1% (v/v) Tween 20] twice for 30 min at 65°C, and finally with MABT buffer twice for 5 min. Then, the slides were incubated in PBS containing 3% H_2_O_2_ for 30 min and washed and blocked with blocking reagent (Roche) for 1 h. After blocking, the slides were incubated with HRP‐conjugated anti‐DIG antibody (1:500; Roche) in blocking reagent for 16 h at 4°C. The samples were washed three times (5 min each) with MABT buffer. Signals were visualized by incubating the sample with Tyramide‐Alexa Fluor 488 (1:200) for 10 min using a TSA kit (Life Technologies). After three rinses with PBS, coverslips were placed on the slides for examination. Fluorescence signals were detected using an FV1000 confocal laser‐scanning microscope (Olympus, Tokyo, Japan).

### Western blot analysis of MPST in postmortem brain samples

Western blot analysis was performed as per our previous report (Ohnishi *et al*, 2019). The protein (40 μg/lane) was analyzed in Western blot with anti‐MPST (Nagahara *et al*, 2013; dilution 1:3,000) and anti‐GAPDH (Santa Cruz Biotechnology, sc‐20357; dilution 1:5,000) antibodies. The chemiluminescent intensities for the signal of MPST were normalized to those of GAPDH.

### Receiver operating characteristic (ROC) curve analysis

The equations for calculation of sensitivity and specificity were “sensitivity = number of true positives/(number of true positives + number of false negatives)” and “specificity = number of true negatives/(number of false positives + number of true negatives)” with the criterion that relative expression levels of *MPST* mRNA above a cut‐off value were deemed as positive for schizophrenia.

We used the Mann–Whitney *U* test (two‐tailed) to detect significant changes in the expression levels of each gene. Correlations of confounding factors for *MPST* gene expression were calculated by Spearman's rank correlation test.

### RNA‐seq analysis

Transcriptome analysis in the frontal cortical brain region was performed by RNA‐seq in (a) *Mpst‐*KO mice (*n* = 6) versus WT controls (*n* = 6) and (b) *Mpst‐*Tg (*n* = 6) versus littermate (non‐Tg) controls (*n* = 6). Total RNA from the frontal cortical brain region was extracted (miRNeasy Mini Kit, Qiagen, Hilden, Germany), and the quantity and quality of RNA were estimated (2200 TapeStation system, Agilent, Santa Clara, CA, USA). Samples with RNA integrity number (RIN) ≥ 8.7 were further used for library preparation with 200 ng of total RNA according to the manufacturer's instructions for the TruSeq Stranded mRNA Sample Prep Kit (Illumina, San Diego, CA, USA). Briefly, poly‐A‐containing mRNA was purified using poly‐T oligo‐attached magnetic beads and then heat fragmented and reverse transcribed into first‐strand cDNA using reverse transcriptase and random primers. Second‐strand cDNA synthesis was performed by incorporating dUTP followed by addition of a single “A” nucleotide at the 3′ ends of the blunt fragments to prevent ligation of double‐stranded cDNA. After adapter ligation (including multiplexing barcodes), the cDNA fragments were enriched by PCR (15 cycles) to create the final cDNA library. The quality, size distribution, and quantity of the cDNA libraries were assessed (2100 Bioanalyzer, Agilent), and the libraries were further sequenced in 100‐bp paired‐end read format on the HiSeq 2500 platform (Illumina).

The RNA‐seq data for the individual samples were demultiplexed using the unique index adapters. The quality of the sequence reads was evaluated by FastQC (http://wwwbioinformaticsbabrahamacuk/projects/fastqc), and the reads were trimmed for adapter sequences and low‐quality bases using the FASTX tool kit (http://hannonlabcshledu/fastx_toolkit/indexhtml). The reads were further mapped to the mouse reference genome (GRCm38/mm10, http://hgdownload.soe.ucsc.edu/goldenpath/mm10/chromosomes/) using TopHat with default parameters (v.2.0.14) (Trapnell *et al*, 2012), utilizing the aligner Bowtie2 (v.2.2.5) (Langmead *et al*, 2009). The expression levels were quantified using Cufflinks (v.2.2.1) (Trapnell *et al*, 2012) based on the read mapping and calculated as fragments per kilobase of transcript per million mapped reads (FPKM), corresponding to the UCSC gene annotations for mm10 (http://hgdownload.soe.ucsc.edu/goldenpath/mm10/database/refFlat.txt.gz). To test the statistical significance for differential expression among the comparison groups, Student's *t*‐test on log‐transformed FPKM values (log_2_ FPKM) was applied. *P* values < 0.05 were considered statistically significant, and these reads were further analyzed for gene ontology enrichment and pathway analysis. Visualization of differentially expressed genes using volcano plots was performed in R (https://www.r-project.org). Differentially expressed genes were tested for gene ontology enrichment and pathway analysis. Gene ontology enrichment analysis was performed in the PANTHER Overrepresentation Test (http://pantherdb.org/webservices/go/overrep.jsp, annotation version and release date: GO Ontology database, released on 2018‐06‐01). Reported *P* values were corrected for multiple testing using the FDR method. Pathway enrichment was performed using Ingenuity Pathway Analysis (IPA) (Qiagen, content version: 36601845, release date: 2017‐06‐22). The statistical significance of the enriched canonical signaling pathways was calculated using Fischer's exact test. *P* values < 0.05 were considered statistically significant.

### Analysis of metabolites

To minimize postmortem degradation, we subjected mice to high‐energy focused beam microwave irradiation (5 kW, 0.94 s; Muromachi Kikai, Tokyo, Japan) without anesthesia and then analyzed the extracted brains (Sugiura *et al*, 2014). A report provided by the American Veterinary Medical Association Recommendations described that microwave irradiation is a humane method for euthanizing small laboratory rodents, with the advantages that unconsciousness is achieved in < 100 ms and a complete loss of brain function in < 1 s.

ATP and ADP concentrations were measured using MRM (Multiple Reaction Monitoring) method by the LC‐triple quadrupole mass spectrometer (UHPLC: ACQUITY UPLC, Waters, Milford, Massachusetts, USA; mass spectrometer: TSQ Vantage EMR, ThermoFisher), and using d5‐AMP as an internal standard, at the Support Unit for Bio‐Material Analysis in RIKEN Center for Brain Science, Research Resource Division (RRD). Mouse samples (more or less than mean ± 1.5SD in terms of ATP‐to‐ADP ratio) were excluded (*n* = 1 for non‐Tg group and *n* = 1 for *Mpst* ‐Tg group) for analysis.

### Analysis of mitochondrial cytochrome c oxidase activity

Mitochondrial cytochrome c oxidase activity was measured in non‐Tg (*n* = 6) and *Mpst‐*Tg mice (*n* = 6) (both age of 6 weeks). Fresh mitochondria were isolated from cerebral hemisphere by using Mitochondria Isolation Kit for Tissue and Cultured Cells (Biochain, Newark, CA, USA) and collected pellets were stored at −80°C until use. At the timing of measurement, pellets were re‐suspended in the storage buffer [10 mM HEPES (pH 7.1–7.5), 250 mM sucrose, 1 mM ATP, 80 μΜ ADP, 5 mM sodium succinate, and 2 mM K_2_HPO_4_]. The activity was determined by Cytochrome c Oxidase Assay Kit (Sigma). The assay was performed using Ultrospec 3300 pro (GE Healthcare). Results were represented in units per mg of protein defined by Micro BCA Protein Assay Kit (ThermoFisher).

### Analysis of spines

Primary culture of E16.5 hippocampal neurons from non‐Tg and *Mpst‐*Tg mice were performed as previously described (Morikawa *et al*, 2018). Briefly, dissociated hippocampal neurons were plated on chambered coverslips coated with polyethyleneimine at 1 × 10^5^ cells per well and cultured in a 5% CO_2_ atmosphere at 37°C. Cultured hippocampal neurons at DIV 14–21 were transfected with an EGFP expression vector using the calcium phosphate method for 2 days before the observation. The dendritic spines were observed using a confocal laser‐scanning microscope (LSM780) equipped with an Airyscan module (ZEISS). Data analyses were performed using IMARIS (BITPLANE) software (Oxford Instruments, Abingdon, UK).

### Protein *S*‐palmitoylation assay

Protein *S*‐palmitoylation in the brain was examined by the modified ABE method (Forrester *et al*, 2011) with minor modifications. Brain tissues (~20 mg wet weight) were lysed in 250 μl of lysis buffer (50 mM Tris–HCl (pH 7.5), 150 mM NaCl, 5 mM EDTA, 4% SDS, 2 mM PMSF) by sonication. The lysates were diluted with 250 μl of Triton buffer (50 mM NaCl (pH 7.5), 150 mM NaCl, 5 mM EDTA, 0.2% Triton X‐100, 2 mM PMSF) and then centrifuged for 10 min at 18,000 *g* and 15°C. The supernatants (~1.1 mg of protein) were treated with 20 mM TCEP [Tris(2‐carboxyethyl)phosphine, FUJIFILM Wako Pure Chemical Co., Osaka, Japan] for 30 min at room temperature in a reaction volume of 1 ml to cleave disulfide bonds. Next, the mixtures were treated with 40 mM NEM (N‐ethyl maleimide) for 2.5 h to block free thiol groups. For detection of “global” *S*‐palmitoylation, one‐tenth of the mixture was stored as “input” for Western blot analysis with HRP‐labeled streptavidin (GE Healthcare) under non‐reducing conditions followed by non‐reducing SDS–PAGE. For detection of palmitoylation of specific proteins, the remaining mixture after the ABE reaction was extracted with 4 ml of methanol, 1 ml of chloroform, and 3 ml of water, and the upper phase obtained after centrifugation at 5,800 *g* for 10 min was removed. The pellet was rinsed twice with 10 ml of methanol. The pellets were completely dissolved in 550 μl of 4% SDS buffer (4% SDS, 50 mM Tris–HCl (pH 7.5), 5 mM EDTA) by sonication. To a 250‐μl aliquot, 750 μl of HA(+) buffer [1 M hydroxyamine (pH 7.5), 150 mM NaCl, 0.2% Triton X‐100, 1 mM biotin‐HPDP (*N*‐[6‐(biotinamide)hexyl]‐3′‐(2′‐pyridyldithio)propionamide; ThermoFisher), and 1 mM PMSF] or HA(−) buffer (1 M Tris–HCl (pH 7.5), 150 mM NaCl, 0.2% Triton X‐100, 1 mM biotin‐HPDP, and 1 mM PMSF). After incubation for an hour, the mixture was extracted with 4 ml of methanol, 1 ml of chloroform, and 3 ml of water, and the upper phase obtained after centrifugation at 5,800 *g* for 10 min was removed. The pellet was rinsed twice with 10 ml of methanol. The pellet was dried and then dissolved in 170 μl of 2% SDS buffer [2% SDS, 50 mM Tris–HCl (pH 7.5), 150 mM NaCl, 5 mM EDTA, and 1 mM PMSF] by sonication. After diluting with 10 volumes of 0.2% Triton buffer (0.2% Triton X‐100, 50 mM Tris–HCl (pH 7.5), 5 mM EDTA), the biotinylated proteins were captured with NeurAvidin agarose beads (ThermoFisher). The complex was rinsed with wash buffer (0.2% Triton buffer supplemented with 0.1% SDS) three times. Biotinylated proteins were released with 1.5× SDS sample buffer by boiling for 10 min and subjected to Western blot analysis with an anti‐PSD95 (Cell Signaling, Danvers, MA, USA; cat. no. 3450), anti‐Ras (Cell Signaling, cat. no. 3965), anti‐Cdc42 (Cell Signaling, cat. no. 2466), or anti‐MBP (Cell signaling, cat. no. 78896) antibody.

### Genetic association study

The genetic association of *MPST* and *CBS* with schizophrenia was evaluated by analyzing SNPs in 2,011 cases and 2,170 controls (Bangel *et al*, 2015; Balan *et al*, 2017). *MPST* was examined using 4 tag SNPs, and *CBS* was assessed with 7 tag SNPs and two promoter SNPs (rs1788484 and rs2850144) (Appendix Table S15). The promoter SNPs are described in the Tohoku Medical Megabank Organization of Tohoku University (ToMMo) database of the Japanese population (https://ijgvd.megabank.tohoku.ac.jp) (Nagasaki *et al*, 2015). The SNPs were genotyped using TaqMan SNP genotyping assays (Applied Biosystems) following the manufacturer's instructions.

### DNA methylation analysis

DNA methylation was performed using EpiTYPER on the MassARRAY System platform (Agena Bioscience, San Diego, CA, USA) following the manufacturer's instructions. The sequence information of the probes used for DNA methylation analysis and information on the lengths of PCR products is shown in Appendix Table S17.

### Poly‐I:C model

Pregnant B6 mice received five consecutive intraperitoneal injections of poly‐I:C (2 mg/ml, Sigma) dissolved in PBS (20 mg/kg) or an equivalent volume of PBS at embryonic days 12, 13, 14, 15, and 16. At adulthood (13–14 weeks old), brains were dissected from pups for analysis.

## Author contributions

Conception and design: MId, KU, TY; mass spectrometry analysis: TI, KM, TKata, KU; biochemical analysis of sulfides: NS, YKi, HK; experiments using animals and human samples (generation of model animals, behavioral analysis, genetic analysis, biochemical analysis, metabolic analysis, spine analysis, etc.): TO, MT, MMa, C‐SM, YI, HOh, AW, YH, YM, TH, MMo, KH, YN, YW, YT, TKato, AN, SF, NH, KI; iPS cell study: MT, YH, HOk; collection and management of postmortem brain samples: YKu, AK, HY, BD; collection and evaluation of other clinical samples: KH, TT, MIt; analysis and interpretation of data (e.g., statistical analysis, biostatistics, computational analysis): MId, TO, MT, SB, YI, AW, TH, HK, TY; writing, review, and/or revision of the manuscript: MId, TO, SB, BD, HK, TY; study supervision: TY.

## Conflict of interest

The authors declare that they have no conflict of interest.

**The paper explained**

**Problem**

Schizophrenia is a severe mental illness, and susceptibility to schizophrenia is given by multiple genetic variants and environmental factors, the latter particularly being subtle insults including oxidative/inflammatory stress during the early brain development (termed the “neurodevelopmental hypothesis”). However, a precise mechanism for non‐genetic effects remains largely elusive.

**Results**

We focused on the differential integrity of prepulse inhibition (PPI), a representative endophenotype of schizophrenia, between different inbred mouse strains. Our proteomics analysis and examination of gene‐manipulated mice revealed that elevated levels of Mpst, a hydrogen sulfide (H_2_S)/polysulfides‐producing enzyme, led to impaired PPI with increased sulfide deposition. Analysis of human samples demonstrated that the H_2_S/polysulfides production system is indeed upregulated in schizophrenia (“sulfide stress”). Mechanistically, *Mpst* overexpression dampened energy metabolism, while maternal immune activation model mice showed upregulation of genes for H_2_S/polysulfides production, partly via epigenetic modifications. These results suggest that inflammatory/oxidative insults in early brain development result in upregulated H_2_S/polysulfides production as an antioxidative response, which in turn cause deficits in bioenergetic processes.

**Impact**

This study revealed that an elevated H_2_S/polysulfides‐producing system (“sulfide stress”) underlies the pathophysiology of a subset of schizophrenia, explaining a novel aspect of the “neurodevelopmental hypothesis”, and could provide a novel paradigm for drug development.

## For more information

(i) The novel insertion/deletion polymorphism in the *Npm1* gene and a novel missense polymorphism in the *Nme2* gene in mouse genome, which we have identified in this study, have been deposited in the NCBI database (http://www.ncbi.nlm.nih.gov) and have been assigned the tentative IDs ss410758760 and ss410758759, respectively.

## Supporting information



Review Process File

Appendix

Expanded View Figures PDF

Source Data for Figure 1

Source Data for Figure 3

Source Data for Figure 7

## Data Availability

RNA‐seq data of *Mpst‐*KO and Tg mice are available on NCBI BioProject (https://www.ncbi.nlm.nih.gov/bioproject) accession numbers PRJDB8817 and PRJDB8818, respectively. Proteomics data of mouse brain and lymphocytes are available on PeptideAtlas (http://www.peptideatlas.org) Dataset Identifier PASS01452.

## References

[emmm201910695-bib-0001] American Psychiatric Association (2013) Diagnostic and statistical manual of mental disorders, 5^th^ edn. Arlington, VA: American Psychiatric Publishing

[emmm201910695-bib-0002] Balan S , Yamada K , Iwayama Y , Hashimoto T , Toyota T , Shimamoto C , Maekawa M , Takagai S , Wakuda T , Kameno Y *et al* (2017) Comprehensive association analysis of 27 genes from the GABAergic system in Japanese individuals affected with schizophrenia. Schizophr Res 185: 33–40 28073605 10.1016/j.schres.2017.01.003

[emmm201910695-bib-0003] Bangel FN , Yamada K , Arai M , Iwayama Y , Balan S , Toyota T , Iwata Y , Suzuki K , Kikuchi M , Hashimoto T *et al* (2015) Genetic analysis of the glyoxalase system in schizophrenia. Prog Neuropsychopharmacol Biol Psychiatry 59: 105–110 25645869 10.1016/j.pnpbp.2015.01.014

[emmm201910695-bib-0004] Barbaux S , Plomin R , Whitehead AS (2000) Polymorphisms of genes controlling homocysteine/folate metabolism and cognitive function. NeuroReport 11: 1133–1136 10790895 10.1097/00001756-200004070-00044

[emmm201910695-bib-0005] Birnbaum R , Weinberger DR (2017) Genetic insights into the neurodevelopmental origins of schizophrenia. Nat Rev Neurosci 18: 727–740 29070826 10.1038/nrn.2017.125

[emmm201910695-bib-0006] Braff DL , Geyer MA , Swerdlow NR (2001) Human studies of prepulse inhibition of startle: normal subjects, patient groups, and pharmacological studies. Psychopharmacology 156: 234–258 11549226 10.1007/s002130100810

[emmm201910695-bib-0007] Bubber P , Hartounian V , Gibson GE , Blass JP (2011) Abnormalities in the tricarboxylic acid (TCA) cycle in the brains of schizophrenia patients. Eur Neuropsychopharmacol 21: 254–260 21123035 10.1016/j.euroneuro.2010.10.007 PMC3033969

[emmm201910695-bib-0008] Bundo M , Toyoshima M , Okada Y , Akamatsu W , Ueda J , Nemoto‐Miyauchi T , Sunaga F , Toritsuka M , Ikawa D , Kakita A *et al* (2014) Increased l1 retrotransposition in the neuronal genome in schizophrenia. Neuron 81: 306–313 24389010 10.1016/j.neuron.2013.10.053

[emmm201910695-bib-0009] Burlina A , Zacchello F , Dionisi‐Vici C , Bertini E , Sabetta G , Bennet MJ , Hale DE , Schmidt‐Sommerfeld E , Rinaldo P (1991) New clinical phenotype of branched‐chain acyl‐CoA oxidation defect. Lancet 338: 1522–1523 10.1016/0140-6736(91)92338-3 1683940

[emmm201910695-bib-0010] Cooper CE , Brown GC (2008) The inhibition of mitochondrial cytochrome oxidase by the gases carbon monoxide, nitric oxide, hydrogen cyanide and hydrogen sulfide: chemical mechanism and physiological significance. J Bioenerg Biomembr 40: 533–539 18839291 10.1007/s10863-008-9166-6

[emmm201910695-bib-0011] Cucino C , Sonnenberg A (2001) The comorbid occurrence of other diagnoses in patients with ulcerative colitis and Crohn's disease. Am J Gastroenterol 96: 2107–2112 11467640 10.1111/j.1572-0241.2001.03943.x

[emmm201910695-bib-0012] Duncan GE , Moy SS , Lieberman JA , Koller BH (2006) Effects of haloperidol, clozapine, and quetiapine on sensorimotor gating in a genetic model of reduced NMDA receptor function. Psychopharmacology 184: 190–200 16362405 10.1007/s00213-005-0214-1

[emmm201910695-bib-0013] Ebrahimi M , Yamamoto Y , Sharifi K , Kida H , Kagawa Y , Yasumoto Y , Islam A , Miyazaki H , Shimamoto C , Maekawa M (2016) Astrocyte‐expressed FABP 7 regulates dendritic morphology and excitatory synaptic function of cortical neurons. Glia 64: 48–62 26296243 10.1002/glia.22902

[emmm201910695-bib-0014] Egashira N , Tanoue A , Higashihara F , Fuchigami H , Sano K , Mishima K , Fukue Y , Nagai H , Takano Y , Tsujimoto G *et al* (2005) Disruption of the prepulse inhibition of the startle reflex in vasopressin V1b receptor knockout mice: reversal by antipsychotic drugs. Neuropsychopharmacology 30: 1996–2005 15956991 10.1038/sj.npp.1300784

[emmm201910695-bib-0015] Estes ML , McAllister AK (2016) Maternal immune activation: implications for neuropsychiatric disorders. Science 353: 772–777 27540164 10.1126/science.aag3194 PMC5650490

[emmm201910695-bib-0016] Forrester MT , Hess DT , Thompson JW , Hultman R , Moseley MA , Stamler JS , Casey PJ (2011) Site‐specific analysis of protein S‐acylation by resin‐assisted capture. J Lipid Res 52: 393–398 21044946 10.1194/jlr.D011106 PMC3023561

[emmm201910695-bib-0017] Fusar‐Poli P , Borgwardt S , Bechdolf A , Addington J , Riecher‐Rossler A , Schultze‐Lutter F , Keshavan M , Wood S , Ruhrmann S , Seidman LJ *et al* (2013) The psychosis high‐risk state: a comprehensive state‐of‐the‐art review. JAMA Psychiatry 70: 107–120 23165428 10.1001/jamapsychiatry.2013.269 PMC4356506

[emmm201910695-bib-0018] Giovanoli S , Engler H , Engler A , Richetto J , Voget M , Willi R , Winter C , Riva MA , Mortensen PB , Feldon J *et al* (2013) Stress in puberty unmasks latent neuropathological consequences of prenatal immune activation in mice. Science 339: 1095–1099 23449593 10.1126/science.1228261

[emmm201910695-bib-0019] Goubern M , Andriamihaja M , Nubel T , Blachier F , Bouillaud F (2007) Sulfide, the first inorganic substrate for human cells. FASEB J 21: 1699–1706 17314140 10.1096/fj.06-7407com

[emmm201910695-bib-0020] Hancock JT , Whiteman M (2016) Hydrogen sulfide signaling: interactions with nitric oxide and reactive oxygen species. Ann N Y Acad Sci 1365: 5–14 25782612 10.1111/nyas.12733

[emmm201910695-bib-0021] Hildebrandt TM , Grieshaber MK (2008) Three enzymatic activities catalyze the oxidation of sulfide to thiosulfate in mammalian and invertebrate mitochondria. FEBS J 275: 3352–3361 18494801 10.1111/j.1742-4658.2008.06482.x

[emmm201910695-bib-0022] Ichinohe A , Kanaumi T , Takashima S , Enokido Y , Nagai Y , Kimura H (2005) Cystathionine beta‐synthase is enriched in the brains of Down's patients. Biochem Biophys Res Commun 338: 1547–1550 16274669 10.1016/j.bbrc.2005.10.118

[emmm201910695-bib-0023] Ishigami M , Hiraki K , Umemura K , Ogasawara Y , Ishii K , Kimura H (2009) A source of hydrogen sulfide and a mechanism of its release in the brain. Antioxid Redox Signal 11: 205–214 18754702 10.1089/ars.2008.2132

[emmm201910695-bib-0024] Kimura H (2010) Hydrogen sulfide as a physiological mediator: its function and therapeutic applications. Nihon Yakurigaku Zasshi 136: 335–339 21139284 10.1254/fpj.136.335

[emmm201910695-bib-0025] Kimura Y , Mikami Y , Osumi K , Tsugane M , Oka J , Kimura H (2013) Polysulfides are possible H2S‐derived signaling molecules in rat brain. FASEB J 27: 2451–2457 23413359 10.1096/fj.12-226415

[emmm201910695-bib-0026] Kimura H (2015) Hydrogen sulfide and polysulfides as signaling molecules. Proc Jpn Acad Ser B Phys Biol Sci 91: 131–159 10.2183/pjab.91.131 PMC4568289 25864468

[emmm201910695-bib-0027] Kimura Y , Toyofuku Y , Koike S , Shibuya N , Nagahara N , Lefer D , Ogasawara Y , Kimura H (2015) Identification of H2S3 and H2S produced by 3‐mercaptopyruvate sulfurtransferase in the brain. Sci Rep 5: 14774 26437775 10.1038/srep14774 PMC4594004

[emmm201910695-bib-0028] Kimura Y , Koike S , Shibuya N , Lefer D , Ogasawara Y , Kimura H (2017) 3‐Mercaptopyruvate sulfurtransferase produces potential redox regulators cysteine‐ and glutathione‐persulfide (Cys‐SSH and GSSH) together with signaling molecules H2S2, H2S3 and H2S. Sci Rep 7: 10459 28874874 10.1038/s41598-017-11004-7 PMC5585270

[emmm201910695-bib-0029] Knuesel I , Chicha L , Britschgi M , Schobel SA , Bodmer M , Hellings JA , Toovey S , Prinssen EP (2014) Maternal immune activation and abnormal brain development across CNS disorders. Nat Rev Neurol 10: 643–660 25311587 10.1038/nrneurol.2014.187

[emmm201910695-bib-0030] Koga M , Serritella AV , Sawa A , Sedlak TW (2016) Implications for reactive oxygen species in schizophrenia pathogenesis. Schizophr Res 176: 52–71 26589391 10.1016/j.schres.2015.06.022

[emmm201910695-bib-0031] Koike S , Nishimoto S , Ogasawara Y (2017) Cysteine persulfides and polysulfides produced by exchange reactions with H2S protect SH‐SY5Y cells from methylglyoxal‐induced toxicity through Nrf2 activation. Redox Biol 12: 530–539 28371750 10.1016/j.redox.2017.03.020 PMC5377440

[emmm201910695-bib-0032] Kunii Y , Hyde TM , Ye T , Li C , Kolachana B , Dickinson D , Weinberger DR , Kleinman JE , Lipska BK (2014) Revisiting DARPP‐32 in postmortem human brain: changes in schizophrenia and bipolar disorder and genetic associations with t‐DARPP‐32 expression. Mol Psychiatry 19: 192–199 23295814 10.1038/mp.2012.174

[emmm201910695-bib-0033] Kwak WJ , Kwon GS , Jin I , Kuriyama H , Sohn HY (2003) Involvement of oxidative stress in the regulation of H(2)S production during ultradian metabolic oscillation of *Saccharomyces cerevisiae* . FEMS Microbiol Lett 219: 99–104 12594030 10.1016/S0378-1097(02)01198-9

[emmm201910695-bib-0034] Langmead B , Trapnell C , Pop M , Salzberg SL (2009) Ultrafast and memory‐efficient alignment of short DNA sequences to the human genome. Genome Biol 10: R25 19261174 10.1186/gb-2009-10-3-r25 PMC2690996

[emmm201910695-bib-0035] Leucht S , Burkard T , Henderson J , Maj M , Sartorius N (2007) Physical illness and schizophrenia: a review of the literature. Acta Psychiatr Scand 116: 317–333 17919153 10.1111/j.1600-0447.2007.01095.x

[emmm201910695-bib-0036] Lewis DA , Lieberman JA (2000) Catching up on schizophrenia: natural history and neurobiology. Neuron 28: 325–334 11144342 10.1016/s0896-6273(00)00111-2

[emmm201910695-bib-0037] Lewis DA , Hashimoto T , Volk DW (2005) Cortical inhibitory neurons and schizophrenia. Nat Rev Neurosci 6: 312–324 15803162 10.1038/nrn1648

[emmm201910695-bib-0038] Linden DR , Furne J , Stoltz G , Abdel‐Rehim M , Levitt MD , Szurszewski J (2012) Sulphide quinone reductase contributes to hydrogen sulphide metabolism in murine peripheral tissues but not in the CNS. Br J Pharmacol 165: 2178–2190 21950400 10.1111/j.1476-5381.2011.01681.x PMC3413855

[emmm201910695-bib-0039] Maekawa M , Yamada K , Toyoshima M , Ohnishi T , Iwayama Y , Shimamoto C , Toyota T , Nozaki Y , Balan S , Matsuzaki H *et al* (2015) Utility of scalp hair follicles as a novel source of biomarker genes for psychiatric illnesses. Biol Psychiatry 78: 116–125 25444170 10.1016/j.biopsych.2014.07.025

[emmm201910695-bib-0040] Maekawa M , Ohnishi T , Balan S , Hisano Y , Nozaki Y , Ohba H , Toyoshima M , Shimamoto C , Tabata C , Wada Y *et al* (2019) Thiosulfate promotes hair growth in mouse model. Biosci Biotechnol Biochem 83: 114–122 30200826 10.1080/09168451.2018.1518705

[emmm201910695-bib-0041] Marechal D , Brault V , Leon A , Martin D , Lopes Pereira P , Loaec N , Birling MC , Friocourt G , Blondel M , Herault Y (2019) Cbs overdosage is necessary and sufficient to induce cognitive phenotypes in mouse models of Down syndrome and interacts genetically with Dyrk1a. Hum Mol Genet 28: 1561–1577 30649339 10.1093/hmg/ddy447

[emmm201910695-bib-0042] Meyer U , Feldon J (2012) To poly(I:C) or not to poly(I:C): advancing preclinical schizophrenia research through the use of prenatal immune activation models. Neuropharmacology 62: 1308–1321 21238465 10.1016/j.neuropharm.2011.01.009

[emmm201910695-bib-0043] Mineri R , Rimoldi M , Burlina AB , Koskull S , Perletti C , Heese B , von Dobeln U , Mereghetti P , Di Meo I , Invernizzi F *et al* (2008) Identification of new mutations in the ETHE1 gene in a cohort of 14 patients presenting with ethylmalonic encephalopathy. J Med Genet 45: 473–478 18593870 10.1136/jmg.2008.058271

[emmm201910695-bib-0044] Modis K , Ju Y , Ahmad A , Untereiner AA , Altaany Z , Wu L , Szabo C , Wang R (2016) S‐Sulfhydration of ATP synthase by hydrogen sulfide stimulates mitochondrial bioenergetics. Pharmacol Res 113: 116–124 27553984 10.1016/j.phrs.2016.08.023 PMC5107138

[emmm201910695-bib-0045] Morikawa M , Tanaka Y , Cho HS , Yoshihara M , Hirokawa N (2018) The molecular motor KIF21B mediates synaptic plasticity and fear extinction by terminating Rac1 activation. Cell Rep 23: 3864–3877 29949770 10.1016/j.celrep.2018.05.089

[emmm201910695-bib-0046] Morrison AP , French P , Stewart SL , Birchwood M , Fowler D , Gumley AI , Jones PB , Bentall RP , Lewis SW , Murray GK *et al* (2012) Early detection and intervention evaluation for people at risk of psychosis: multisite randomised controlled trial. BMJ 344: e2233 22491790 10.1136/bmj.e2233 PMC3320714

[emmm201910695-bib-0047] Muniraj N , Stamp LK , Badiei A , Hegde A , Cameron V , Bhatia M (2017) Hydrogen sulfide acts as a pro‐inflammatory mediator in rheumatic disease. Int J Rheum Dis 20: 182–189 25196086 10.1111/1756-185X.12472

[emmm201910695-bib-0048] Murray RM , Bhavsar V , Tripoli G , Howes O (2017) 30 years on: how the neurodevelopmental hypothesis of schizophrenia morphed into the developmental risk factor model of psychosis. Schizophr Bull 43: 1190–1196 28981842 10.1093/schbul/sbx121 PMC5737804

[emmm201910695-bib-0049] Nagahara N , Ito T , Kitamura H , Nishino T (1998) Tissue and subcellular distribution of mercaptopyruvate sulfurtransferase in the rat: confocal laser fluorescence and immunoelectron microscopic studies combined with biochemical analysis. Histochem Cell Biol 110: 243–250 9749958 10.1007/s004180050286

[emmm201910695-bib-0050] Nagahara N , Nagano M , Ito T , Shimamura K , Akimoto T , Suzuki H (2013) Antioxidant enzyme, 3‐mercaptopyruvate sulfurtransferase‐knockout mice exhibit increased anxiety‐like behaviors: a model for human mercaptolactate‐cysteine disulfiduria. Sci Rep 3: 1986 23759691 10.1038/srep01986 PMC3680806

[emmm201910695-bib-0051] Nagahara N , Koike S , Nirasawa T , Kimura H , Ogasawara Y (2018) Alternative pathway of H2S and polysulfides production from sulfurated catalytic‐cysteine of reaction intermediates of 3‐mercaptopyruvate sulfurtransferase. Biochem Biophys Res Commun 496: 648–653 29331374 10.1016/j.bbrc.2018.01.056

[emmm201910695-bib-0052] Nagasaki M , Yasuda J , Katsuoka F , Nariai N , Kojima K , Kawai Y , Yamaguchi‐Kabata Y , Yokozawa J , Danjoh I , Saito S *et al* (2015) Rare variant discovery by deep whole‐genome sequencing of 1,070 Japanese individuals. Nat Commun 6: 8018 26292667 10.1038/ncomms9018 PMC4560751

[emmm201910695-bib-0053] Niu WN , Yadav PK , Adamec J , Banerjee R (2015) S‐glutathionylation enhances human cystathionine beta‐synthase activity under oxidative stress conditions. Antioxid Redox Signal 22: 350–361 24893130 10.1089/ars.2014.5891 PMC4307034

[emmm201910695-bib-0054] Niwa H , Yamamura K , Miyazaki J (1991) Efficient selection for high‐expression transfectants with a novel eukaryotic vector. Gene 108: 193–199 1660837 10.1016/0378-1119(91)90434-d

[emmm201910695-bib-0055] Ohnishi T , Watanabe A , Ohba H , Iwayama Y , Maekawa M , Yoshikawa T (2010) Behavioral analyses of transgenic mice harboring bipolar disorder candidate genes, IMPA1 and IMPA2. Neurosci Res 67: 86–94 20153384 10.1016/j.neures.2010.02.003

[emmm201910695-bib-0056] Ohnishi T , Balan S , Toyoshima M , Maekawa M , Ohba H , Watanabe A , Iwayama Y , Fujita Y , Tan Y , Hisano Y *et al* (2019) Investigation of betaine as a novel psychotherapeutic for schizophrenia. EBioMedicine 45: 432–446 31255657 10.1016/j.ebiom.2019.05.062 PMC6642071

[emmm201910695-bib-0057] Paredes RM , Quinones M , Marballi K , Gao X , Valdez C , Ahuja SS , Velligan D , Walss‐Bass C (2014) Metabolomic profiling of schizophrenia patients at risk for metabolic syndrome. Int J Neuropsychopharmacol 17: 1139–1148 24565079 10.1017/S1461145714000157

[emmm201910695-bib-0058] Paul BD , Sbodio JI , Xu R , Vandiver MS , Cha JY , Snowman AM , Snyder SH (2014) Cystathionine gamma‐lyase deficiency mediates neurodegeneration in Huntington's disease. Nature 509: 96–100 24670645 10.1038/nature13136 PMC4349202

[emmm201910695-bib-0059] Perez‐Torres I , Guarner‐Lans V , Rubio‐Ruiz ME (2017) Reductive stress in inflammation‐associated diseases and the pro‐oxidant effect of antioxidant agents. Int J Mol Sci 18: E2098 28981461 10.3390/ijms18102098 PMC5666780

[emmm201910695-bib-0060] Pinner AL , Tucholski J , Haroutunian V , McCullumsmith RE , Meador‐Woodruff JH (2016) Decreased protein S‐palmitoylation in dorsolateral prefrontal cortex in schizophrenia. Schizophr Res 177: 78–87 26876311 10.1016/j.schres.2016.01.054 PMC4981568

[emmm201910695-bib-0061] Rajagopal L , Massey BW , Huang M , Oyamada Y , Meltzer HY (2014) The novel object recognition test in rodents in relation to cognitive impairment in schizophrenia. Curr Pharm Des 20: 5104–5114 24345269 10.2174/1381612819666131216114240

[emmm201910695-bib-0062] Rapoport JL , Giedd JN , Gogtay N (2012) Neurodevelopmental model of schizophrenia: update 2012. Mol Psychiatry 17: 1228–1238 22488257 10.1038/mp.2012.23 PMC3504171

[emmm201910695-bib-0063] Roediger WE , Moore J , Babidge W (1997) Colonic sulfide in pathogenesis and treatment of ulcerative colitis. Dig Dis Sci 42: 1571–1579 9286219 10.1023/a:1018851723920

[emmm201910695-bib-0064] Rosoklija G , Toomayan G , Ellis SP , Keilp J , Mann JJ , Latov N , Hays AP , Dwork AJ (2000) Structural abnormalities of subicular dendrites in subjects with schizophrenia and mood disorders: preliminary findings. Arch Gen Psychiatry 57: 349–356 10768696 10.1001/archpsyc.57.4.349

[emmm201910695-bib-0065] Roussos P , Giakoumaki SG , Zouraraki C , Fullard JF , Karagiorga VE , Tsapakis EM , Petraki Z , Siever LJ , Lencz T , Malhotra A *et al* (2016) The relationship of common risk variants and polygenic risk for schizophrenia to sensorimotor gating. Biol Psychiatry 79: 988–996 26212897 10.1016/j.biopsych.2015.06.019

[emmm201910695-bib-0066] Sebastio G , Sperandeo MP , Panico M , de Franchis R , Kraus JP , Andria G (1995) The molecular basis of homocystinuria due to cystathionine beta‐synthase deficiency in Italian families, and report of four novel mutations. Am J Hum Genet 56: 1324–1333 7762555 PMC1801112

[emmm201910695-bib-0067] Shatalin K , Shatalina E , Mironov A , Nudler E (2011) H2S: a universal defense against antibiotics in bacteria. Science 334: 986–990 22096201 10.1126/science.1209855

[emmm201910695-bib-0068] Shibuya N , Tanaka M , Yoshida M , Ogasawara Y , Togawa T , Ishii K , Kimura H (2009) 3‐Mercaptopyruvate sulfurtransferase produces hydrogen sulfide and bound sulfane sulfur in the brain. Antioxid Redox Signal 11: 703–714 18855522 10.1089/ars.2008.2253

[emmm201910695-bib-0069] Shimamoto C , Ohnishi T , Maekawa M , Watanabe A , Ohba H , Arai R , Iwayama Y , Hisano Y , Toyota T , Toyoshima M *et al* (2014) Functional characterization of FABP3, 5 and 7 gene variants identified in schizophrenia and autism spectrum disorder and mouse behavioral studies. Hum Mol Genet 23: 6495–6511 25027319 10.1093/hmg/ddu369 PMC4240203

[emmm201910695-bib-0070] Smaga I , Niedzielska E , Gawlik M , Moniczewski A , Krzek J , Przegalinski E , Pera J , Filip M (2015) Oxidative stress as an etiological factor and a potential treatment target of psychiatric disorders. Part 2. Depression, anxiety, schizophrenia and autism. Pharmacol Rep 67: 569–580 25933971 10.1016/j.pharep.2014.12.015

[emmm201910695-bib-0071] Sugiura Y , Honda K , Kajimura M , Suematsu M (2014) Visualization and quantification of cerebral metabolic fluxes of glucose in awake mice. Proteomics 14: 829–838 23970501 10.1002/pmic.201300047

[emmm201910695-bib-0072] Sullivan CR , Koene RH , Hasselfeld K , O'Donovan SM , Ramsey A , McCullumsmith RE (2019) Neuron‐specific deficits of bioenergetic processes in the dorsolateral prefrontal cortex in schizophrenia. Mol Psychiatry 24: 1319–1328 29497148 10.1038/s41380-018-0035-3 PMC6119539

[emmm201910695-bib-0073] Swerdlow NR , Braff DL , Geyer MA (2016) Sensorimotor gating of the startle reflex: what we said 25 years ago, what has happened since then, and what comes next. J Psychopharmacol 30: 1072–1081 27539931 10.1177/0269881116661075 PMC6036900

[emmm201910695-bib-0074] Szabo C (2007) Hydrogen sulphide and its therapeutic potential. Nat Rev Drug Discov 6: 917–935 17948022 10.1038/nrd2425

[emmm201910695-bib-0075] Szabo C , Ransy C , Modis K , Andriamihaja M , Murghes B , Coletta C , Olah G , Yanagi K , Bouillaud F (2014) Regulation of mitochondrial bioenergetic function by hydrogen sulfide. Part I. Biochemical and physiological mechanisms. Br J Pharmacol 171: 2099–2122 23991830 10.1111/bph.12369 PMC3976625

[emmm201910695-bib-0076] Tantama M , Yellen G (2014) Imaging changes in the cytosolic ATP‐to‐ADP ratio. Methods Enzymol 547: 355–371 25416365 10.1016/B978-0-12-801415-8.00017-5 PMC4323350

[emmm201910695-bib-0077] Tiranti V , Viscomi C , Hildebrandt T , Di Meo I , Mineri R , Tiveron C , Levitt MD , Prelle A , Fagiolari G , Rimoldi M *et al* (2009) Loss of ETHE1, a mitochondrial dioxygenase, causes fatal sulfide toxicity in ethylmalonic encephalopathy. Nat Med 15: 200–205 19136963 10.1038/nm.1907

[emmm201910695-bib-0078] Toyoshima M , Akamatsu W , Okada Y , Ohnishi T , Balan S , Hisano Y , Iwayama Y , Toyota T , Matsumoto T , Itasaka N *et al* (2016) Analysis of induced pluripotent stem cells carrying 22q11.2 deletion. Transl Psychiatry 6: e934 27801899 10.1038/tp.2016.206 PMC5314118

[emmm201910695-bib-0079] Trapnell C , Roberts A , Goff L , Pertea G , Kim D , Kelley DR , Pimentel H , Salzberg SL , Rinn JL , Pachter L (2012) Differential gene and transcript expression analysis of RNA‐seq experiments with TopHat and Cufflinks. Nat Protoc 7: 562–578 22383036 10.1038/nprot.2012.016 PMC3334321

[emmm201910695-bib-0080] Tremblay R , Lee S , Rudy B (2016) GABAergic interneurons in the neocortex: from cellular properties to circuits. Neuron 91: 260–292 27477017 10.1016/j.neuron.2016.06.033 PMC4980915

[emmm201910695-bib-0081] Tripatara P , Patel NS , Collino M , Gallicchio M , Kieswich J , Castiglia S , Benetti E , Stewart KN , Brown PA , Yaqoob MM *et al* (2008) Generation of endogenous hydrogen sulfide by cystathionine gamma‐lyase limits renal ischemia/reperfusion injury and dysfunction. Lab Invest 88: 1038–1048 18679378 10.1038/labinvest.2008.73

[emmm201910695-bib-0082] Uno Y , Coyle JT (2019) Glutamate hypothesis in schizophrenia. Psychiatry Clin Neurosci 73: 204–215 30666759 10.1111/pcn.12823

[emmm201910695-bib-0083] Wallace JL , Wang R (2015) Hydrogen sulfide‐based therapeutics: exploiting a unique but ubiquitous gasotransmitter. Nat Rev Drug Discov 14: 329–345 25849904 10.1038/nrd4433

[emmm201910695-bib-0084] Wang H , Farhan M , Xu J , Lazarovici P , Zheng W (2017) The involvement of DARPP‐32 in the pathophysiology of schizophrenia. Oncotarget 8: 53791–53803 28881851 10.18632/oncotarget.17339 PMC5581150

[emmm201910695-bib-0085] Watanabe A , Toyota T , Owada Y , Hayashi T , Iwayama Y , Matsumata M , Ishitsuka Y , Nakaya A , Maekawa M , Ohnishi T *et al* (2007) Fabp7 maps to a quantitative trait locus for a schizophrenia endophenotype. PLoS Biol 5: e297 18001149 10.1371/journal.pbio.0050297 PMC2071943

[emmm201910695-bib-0086] Watkins CC , Andrews SR (2016) Clinical studies of neuroinflammatory mechanisms in schizophrenia. Schizophr Res 176: 14–22 26235751 10.1016/j.schres.2015.07.018

[emmm201910695-bib-0087] Włodek L , Ostrowski WS (1982) 3‐Mercaptopyruvate sulphurtransferase from rat erythrocytes. Acta Biochim Pol 29: 121–133 6960622

[emmm201910695-bib-0088] Zuccoli GS , Saia‐Cereda VM , Nascimento JM , Martins‐de‐Souza D (2017) The energy metabolism dysfunction in psychiatric disorders postmortem brains: focus on proteomic evidence. Front Neurosci 11: 493 28936160 10.3389/fnins.2017.00493 PMC5594406

