# Supplementary material for: Excess hydrogen sulfide and polysulfides production underlies a schizophrenia pathophysiology
Source: EMBO Mol Med. 2019 Oct 28;11(12):e10695. doi: 10.15252/emmm.201910695 (PMC6895609; doi:10.15252/emmm.201910695)
Supplement: Supplementary file 2 — Appendix [file EMMM-11-e10695-s001.pdf]

Excess hydrogen sulfide and polysulfides production underlies a schizophrenia pathophysiology

Masayuki Ide [1,2,†], Tetsuo Ohnishi[1,†], Manabu Toyoshima[1], Shabeesh Balan[1], Motoko Maekawa[1], Chie Shimamoto-Mitsuyama[1], Yoshimi Iwayama[1,3], Hisako Ohba[1], Akiko Watanabe,[1] Takashi Ishii[4], Norihiro Shibuya[5,6], Yuka Kimura[5,6], Yasuko Hisano[1], Yui Murata[7], Tomonori Hara[1,8], Momo Morikawa[9], Kenji Hashimoto[10], Yayoi Nozaki[1], Tomoko Toyota[1], Yuina Wada[1,11], Yosuke Tanaka[9], Tadafumi Kato[12], Shigeyoshi Fujisawa[13], Hideyuki Okano[14], Masanari Itokawa[15], Nobutaka Hirokawa[9], Yasuto Kunii[16,17], Akiyoshi Kakita[18], Hirooki Yabe[16], Kazuya Iwamoto[6], Kohji Meno[3], Takuya Katagiri[19], Brian Dean[20,21], Kazuhiko Uchida[22], Hideo Kimura[4,5] & Takeo Yoshikawa[1]*

[1] Laboratory of Molecular Psychiatry, RIKEN Center for Brain Science, Wako 351-0198, Japan

[2] Department of Psychiatry, Division of Clinical Medicine, Faculty of Medicine, University of Tsukuba, Tsukuba 305-8575, Japan

[3] Support Unit for Bio-Material Analysis, Research Division, RIKEN Center for Brain Science, Wako 351-0198, Japan

[4] Research & Development Department, MCBI Inc, Tsukuba 305-0031, Japan

[5] Department of Pharmacology, Sanyo-Onoda City University, Sanyo-Onoda 756-0884, Japan

[6] Department of Molecular Pharmacology, National Institute of Neuroscience, National Center of Neurology and Psychiatry (NCNP), Kodaira 187-8502, Japan

[7] Department of Molecular Brain Science, Graduate School of Medical Sciences, Kumamoto University, Kumamoto 860-0811, Japan

[8] Department of Organ Anatomy, Tohoku University Graduate School of Medicine, Sendai 980-8575, Japan

[9] Department of Cell Biology and Anatomy, Graduate School of Medicine, The University of Tokyo, Tokyo 113-0033, Japan.

[10] Division of Clinical Neuroscience, Chiba University Center for Forensic Mental Health, Chiba 260-8670, Japan

[11] Ochanomizu University, Graduate School of Humanities and Sciences, Tokyo 112-8610, Japan

[12] Laboratory for Molecular Dynamics of Mental Disorders, RIKEN Brain Science Institute, Wako 351-0198, Japan

[13] Laboratory for Systems Neurophysiology, RIKEN Center for Brain Science, Wako 351-0198, Japan

[14] Department of Physiology, Keio University School of Medicine, Tokyo 160-0016, Japan

[15] Center for Medical Cooperation, Tokyo Metropolitan Institute of Medical Science, Tokyo 156-8506, Japan

[16] Department of Neuropsychiatry, School of Medicine, Fukushima Medical University, Fukushima 960-1247, Japan

[17] Department of Psychiatry, Aizu Medical Center, School of Medicine, Fukushima Medical University, Aizuwakamatsu 969-3492, Japan

[18] Department of Pathology, Brain Research Institute, Niigata University 951-8122, Niigata, Japan

[19] Department of Pharmacy, Faculty of Pharmacy, Iryo Sosei University, Iwaki 970-8551, Japan

[20] The Florey Institute of Neuroscience and Mental Health, Howard Florey Laboratories, The University of Melbourne, Parkville, Victoria 3052, Australia

[21] The Centre for Mental Health, Swinburne University, Hawthorn, Victoria 3122, Australia

[22] Department of Molecular Oncology, Division of Biomedical Science, Faculty of Medicine, University of Tsukuba, Tsukuba 305-8575, Japan

[†] These authors contributed equally to this work.

*Corresponding author:
Takeo Yoshikawa, MD, PhD
Laboratory for Molecular Psychiatry
RIKEN Center for Brain Science
2-1 Hirosawa, Wako-city, Saitama 351-0198, Japan
Tel: +81(Japan)-48-467-5968; Fax: +81(Japan)-48-467-7462
E-mail: takeo.yoshikawa@riken.jp

Appendix Figures:

Appendix Figure S1: Biosynthesis of hydrogen sulfide, polysulfide and bound sulfane sulfur species

Appendix Figure S2: The structures of genes corresponding to proteins identified by proteomics

Appendix Figure S3: Purification of recombinant MPST proteins

Appendix Figure S4: Production of hydrogen sulfide from acid-labile sulfur and bound sulfane sulfur

Appendix Figure S5: Generation of *Mpst* KO mice

Appendix Figure S6: Generation of *Mpst* transgenic mice

Appendix Figure S7: No confounding factors affect the expression level of the *MPST* transcripts in postmortem brain samples

Appendix Figure S8: Global protein S-palmitoylation was not affected by a genetic ablation of the *Mpst* gene

Appendix Figure S9: S-palmitoylation on specific proteins was not affected by a genetic ablation of the *Mpst* gene

Appendix Figure S10: Global protein S-palmitoylation was not affected by overexpression of the Mpst protein

Appendix Figure S11: S-palmitoylation on specific proteins was not affected by overexpression of the Mpst protein

Appendix Figure S12: Three variants of the mouse *Mpst* gene and polymorphic region between the two inbred strains B6 and C3H

Appendix Figure S13: Two CpG islands at the mouse *Mpst* locus

Appendix Figure S14: Comparison of the methylation levels between B6 and C3H

Appendix Figure S15: Two CpG islands at the human *MPST* locus

Appendix Figure S16: CpG island at the human *CBS* locus

Appendix Figure S17: The methylation levels of the *CBS* locus in the postmortem brain from schizophrenia patients

Appendix Figure S18: The methylation levels of the *CBS* locus in neurosphere differentiated from schizophrenia patient-specific iPSC

Appendix Figure S19: Oxidative stress and hydrogen sulfide

Appendix Figure S20: No upregulation of typical inflammatory gene expression in offspring from the poly – I: C-treated dams

Appendix tables:

Appendix Table S1. Experimental design for 2D-DIGE

Appendix Table S2. Differentially expressed proteins between B6 and C3H mice analyzed by 2D-DIGE

Appendix Table S3.List of identified proteins showing similar alteration

Appendix Table S4. Identification of proteins showing consistent increases and decreases between brain and lymphocytes derived from B6 and C3H mice by 2D-DIGE

Appendix Table S5. Genetic variations detected in the coding regions of proteins expressed differentially between B6 and C3H mice.

Appendix Table S6. Enzymatic activity of Mpst from B6 and C3H mouse brains

Appendix Table S7. Demographic characteristics of postmortem brains (1st set).

Appendix Table S8. Demographic characteristics of postmortem brains (2nd set)

Appendix Table S9.Demographic characteristics of peripheral blood sample set for *MPST* / MPST expression analyses

Appendix Table S10. Demographic characteristics of hair follicle sample set.

Appendix Table S11. Differentially expressed genes from the frontal cortex of *Mpst* KO mouse

Appendix Table S12. Gene ontology enrichment analysis of differentially expressed genes in the frontal cortex of *Mpst* KO and *Mpst* Tg mouse strains

Appendix Table S13. Differentially expressed genes from the frontal cortex of *Mpst* Tg mouse

Appendix Table S14. Canonical pathways enriched for dysregulated genes in the frontal cortex of *Mpst* KO and *Mpst* Tg mice

Appendix Table S15. Results of genomic quantitative PCR for *Mpst*

Appendix Table S16. Genetic association analyses of *MPST* and *CBS* with schizophrenia

Appendix Table S17. Primer sequences used for DNA methylation analysis and PCR product length

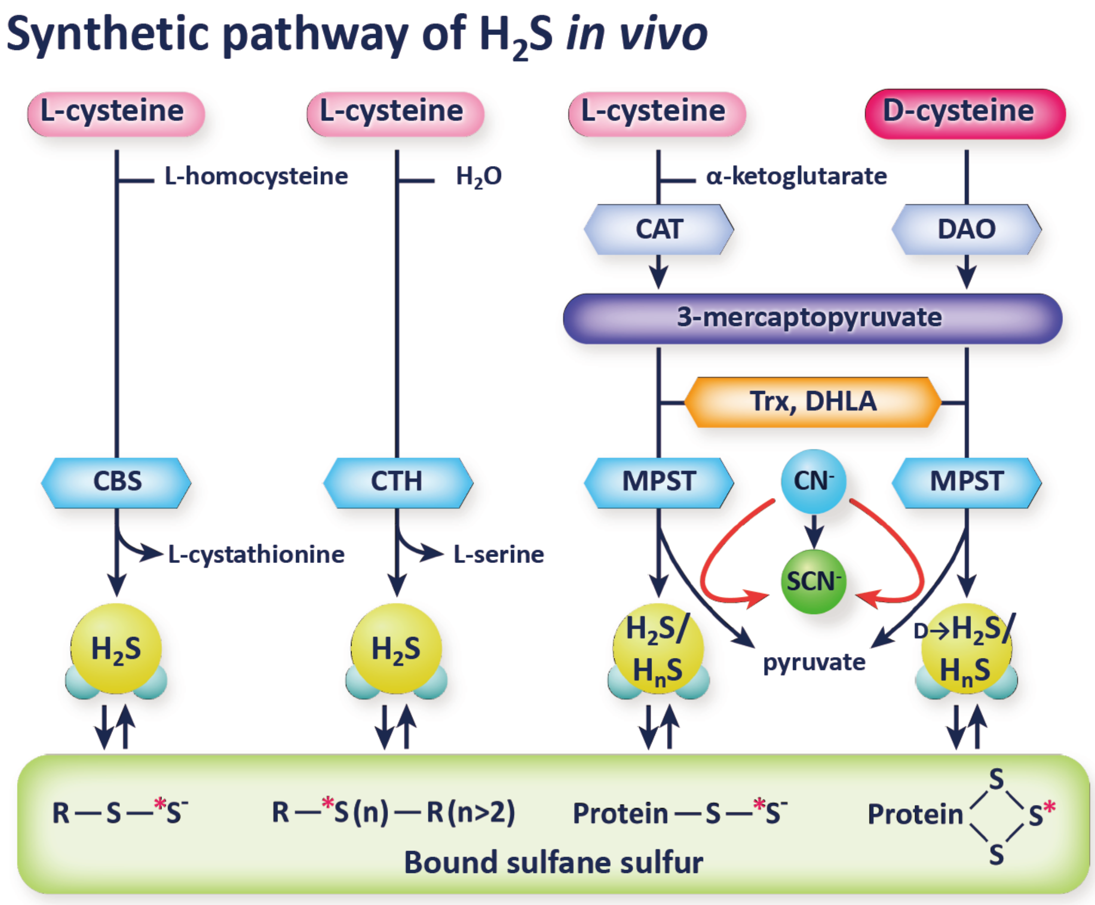

Appendix Figure S1. Biosynthesis of hydrogen sulfide, polysulfide and bound sulfane sulfur species. The three enzymes CBS, CTH and MPST mediate generation of hydrogen sulfide and bound sulfane sulfure *in vivo*. Asterisks indicate sulfane sulfur atoms. CBS; cystathionine β-synthase, CTH; cystathionine γ-lyase, MPST; 3-mercaptopruvate sulfurtransferase, Trx; thioredoxine, DHLA; dihydrolipoic acid.

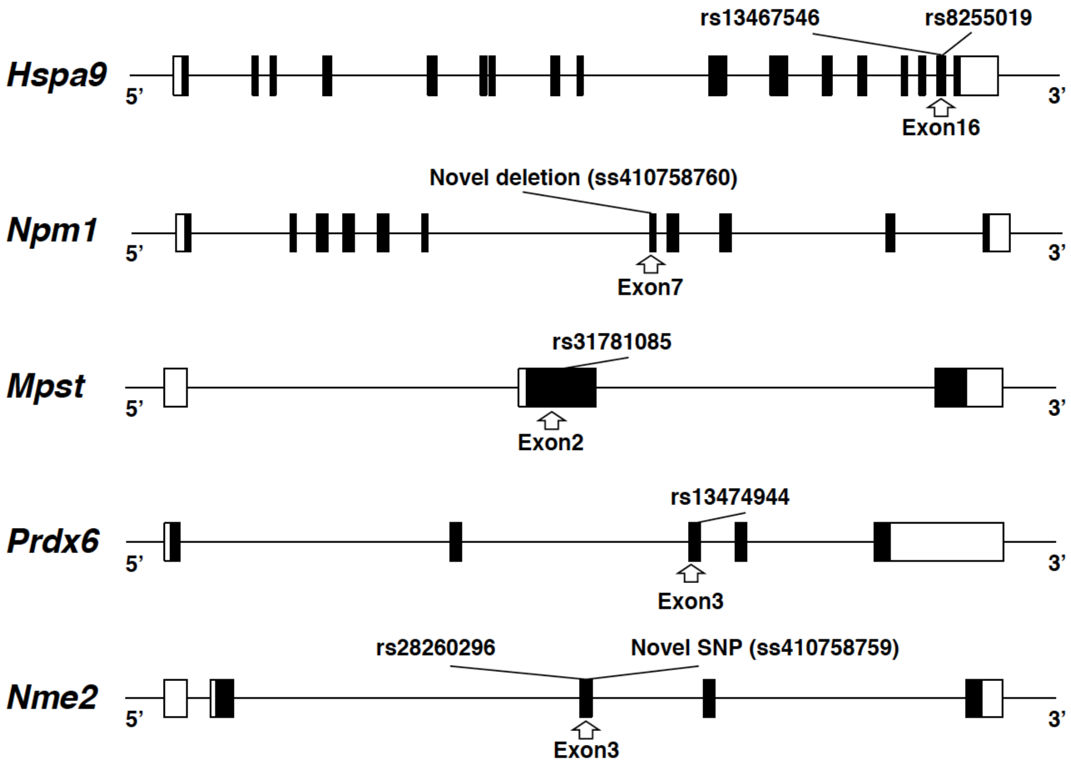

Appendix Figure S2. The structures of genes corresponding to proteins identified by proteomics. Sites found to be polymorphic between B6 and C3H are also indicated. See the text for the details.

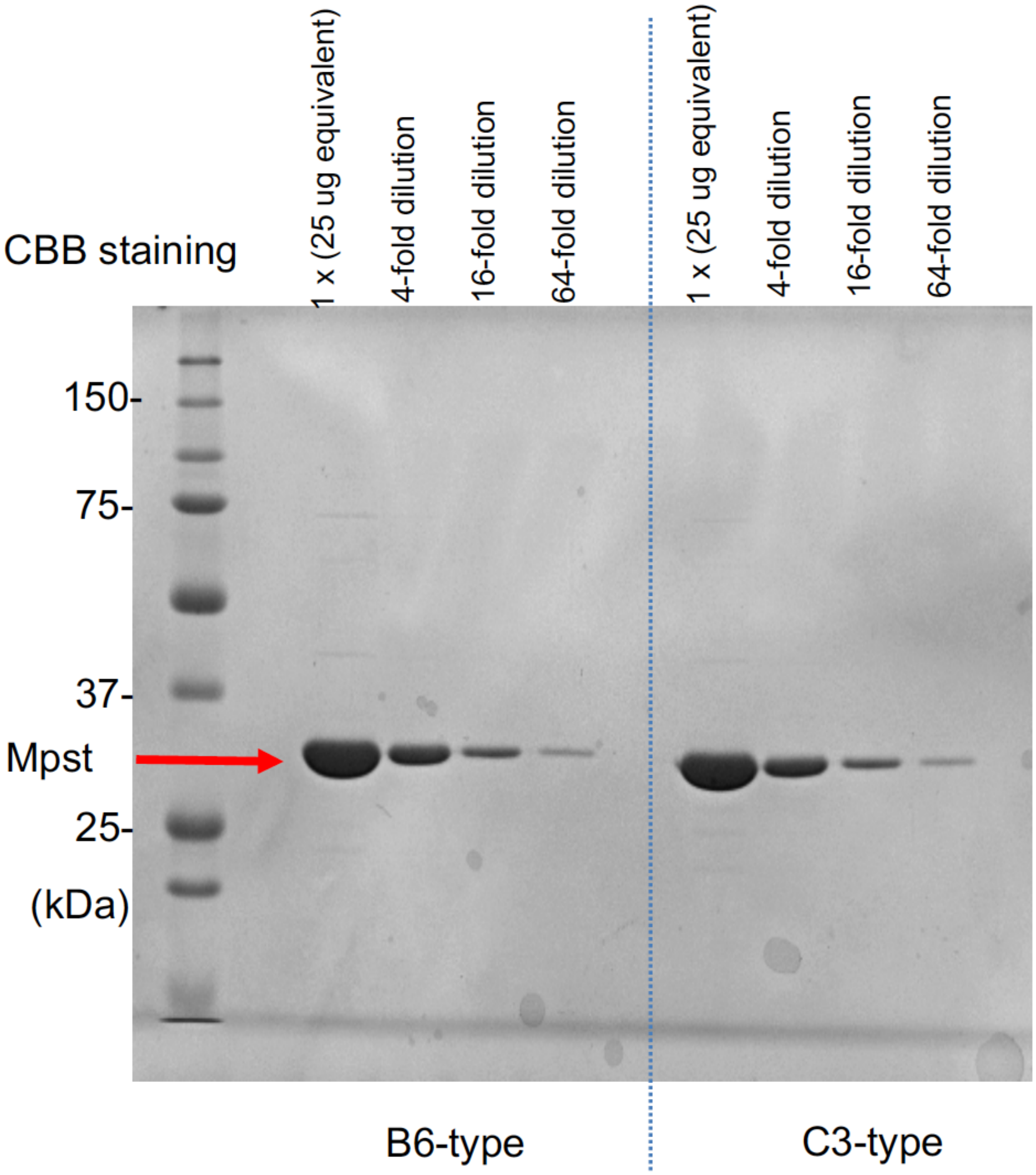

Appendix Figure S3. Purification of recombinant MPST proteins. B6-and C3-type MPST proteins (indicated by an arrow) produced in *E. coli* were purified, and their 4-fold serial dilution series were subjected to SDS-PAGE followed by CBB (Coomassie Brilliant Blue) staining.

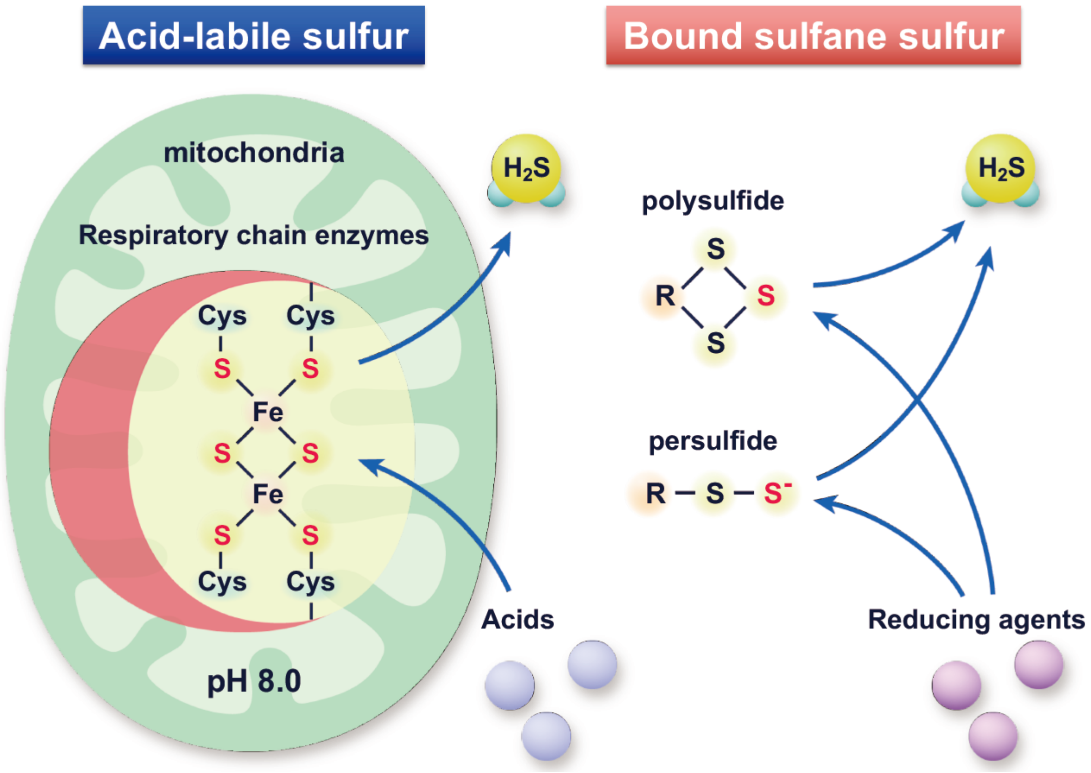

Appendix Figure S4. Production of hydrogen sulfide from acid-labile sulfur and bound sulfane sulfur. Hydrogen sulfide is released under the acidic condition from acid-labile sulfur species in the heme moiety of cytochrome c and other molecules. Bound sulfane sulfur such as polysulfide and persulfide also produce hydrogen sulfide in the presence of reducing agents.

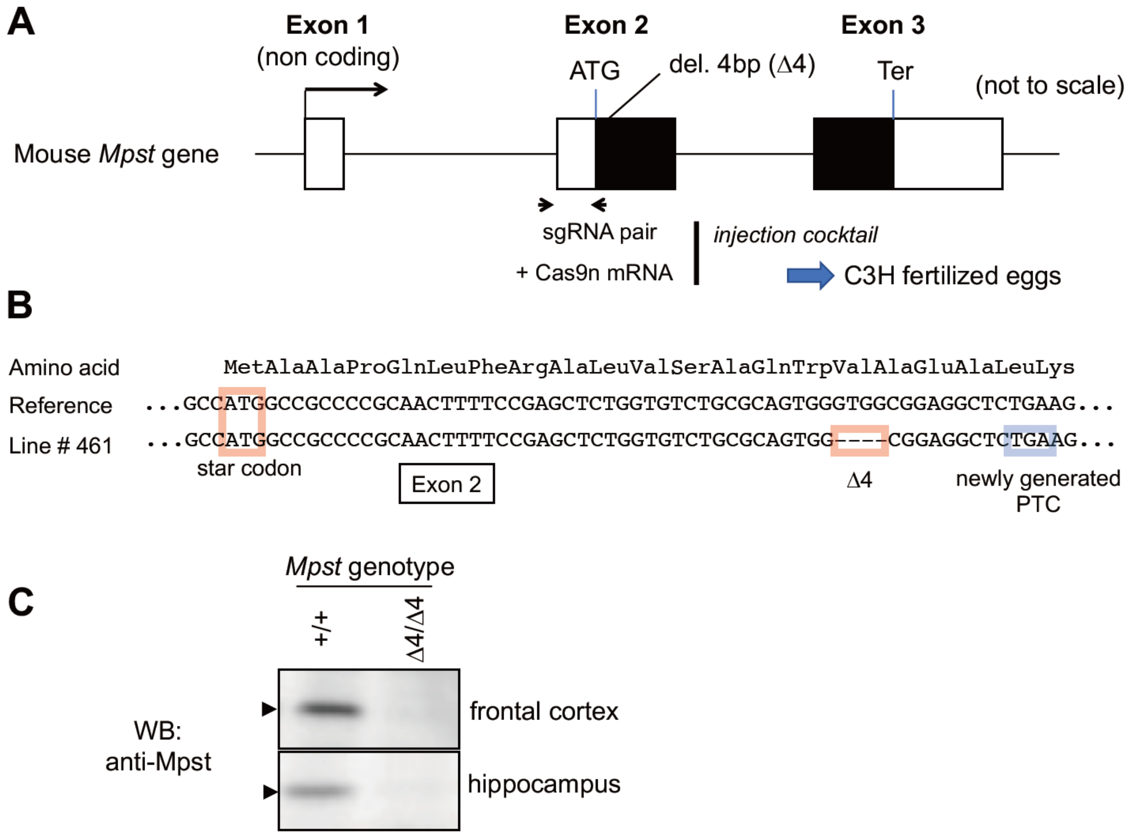

Appendix Figure S5. Generation of *Mpst* KO mice. (**A**) The CRISPR-Cas9n methodology was used to produce *Mpst* KO allele in mice. The upstream sgRNA was designed around the transcription start site of the gene, and the downstream one around the translation start site. The pair of the two sgRNA was microinjected into fertilized eggs of C3H mice with Cas9n mRNA. (**B**) Four bases (boxed in orange) in the first coding exon (exon 2) are deleted in the founder line #461, generating a novel premature termination codon (PTC, boxed in light blue) downstream of the deletion. The start codon is also boxed in orange. (**C**) Western blot analysis confirmed that homozygote for the deletion (Δ4/Δ4) lacks the Mpst protein in the frontal cortex or hippocampus.

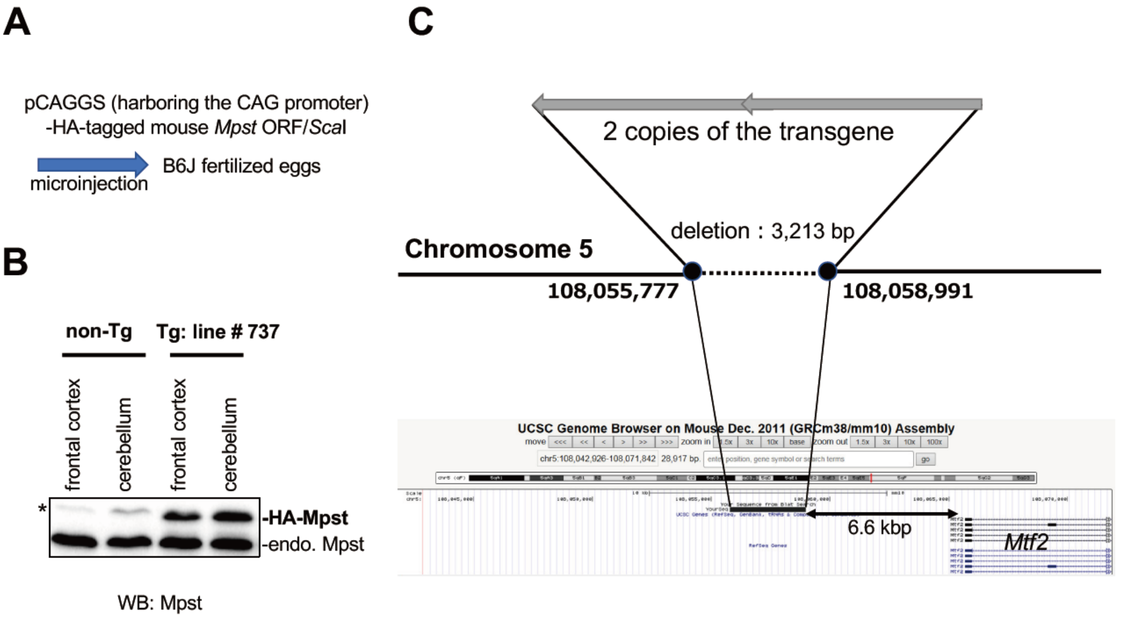

Appendix Figure S6. Generation of *Mpst* transgenic mice. (A) The strategy to generate *Mpst*-Tg mouse is shown. HA-tagged Mpst was placed under the control of the CAG promoter. **(B)** Expression of the transgene was confirmed by western blotting with anti-Mpst antibody in the frontal cortex and cerebellum. Note that the upper and lower bands correspond to the transgene-derived HA-Mpst and endogenous (endo.) Mpst proteins, respectively. The asterisk indicates the position for a non-specific band. **(C)** NGS analysis identified the integration site of the transgene at chromosome 5. Two copies of the transgene were replaced with a ~3.2 kbp region of chromosome 5.

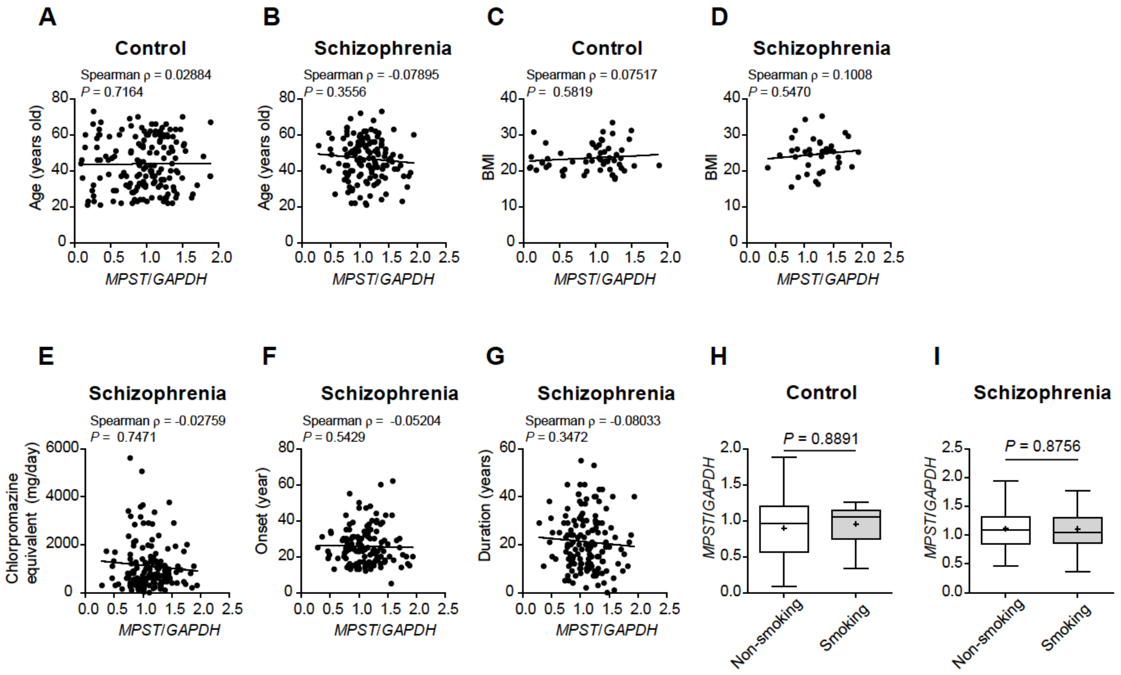

Appendix Figure S7. No confounding factors affect the expression level of the *MPST* transcripts in postmortem brain samples. (A, B) No correlation was found between the *MPST* expression and age in control **(A)** or schizophrenia **(B)** subjects. **(C, D)** No correlation was found between the *MPST* expression and BMI in control **(C)** or schizophrenia **(D)** subjects. **(E-G)** No correlation was found between the *MPST* expression and the amounts of antipsychotics taken (chlorpromazine equivalent) **(E)**, age of onset **(F)** or disease duration **(G)** in schizophrenic patients. **(H, I)** Smoking did not affect the levels of the *MPST* expression in control **(H)** or schizophrenia **(I)** subjects.

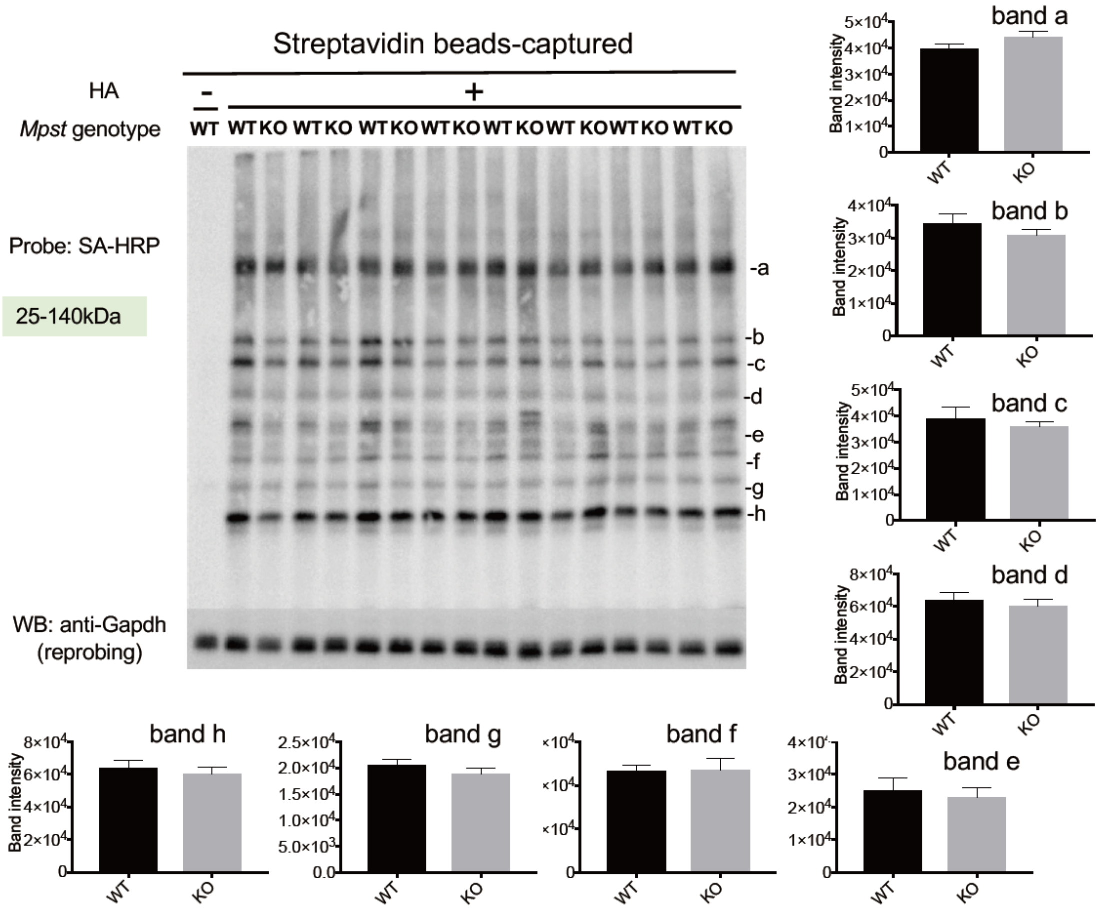

Appendix Figure S8. Global protein S-palmitoylation was not affected by a genetic ablation of the *Mpst* gene. Global protein S-palmitoylation was tested by the modified ABE method. After the treatment with (+) or without (-) hydroxylamine (HA), the proteins captured with streptoavidin beads were subjected to SDS-PAGE. Biotinylated proteins transferred onto a PVDF membrane were detected with HRP-labeled streptavidin and chemiluminescent substrate, producing discrete bands a-h. The sample before beads capturing (input) was also analyzed. The membrane was reprobed with anti-Gapdh antibody. The band intensities were compared between WT and *Mpst* KO animals. The data are shown as means ± SEM (n = 8 for each genotype) in the bar graphs.

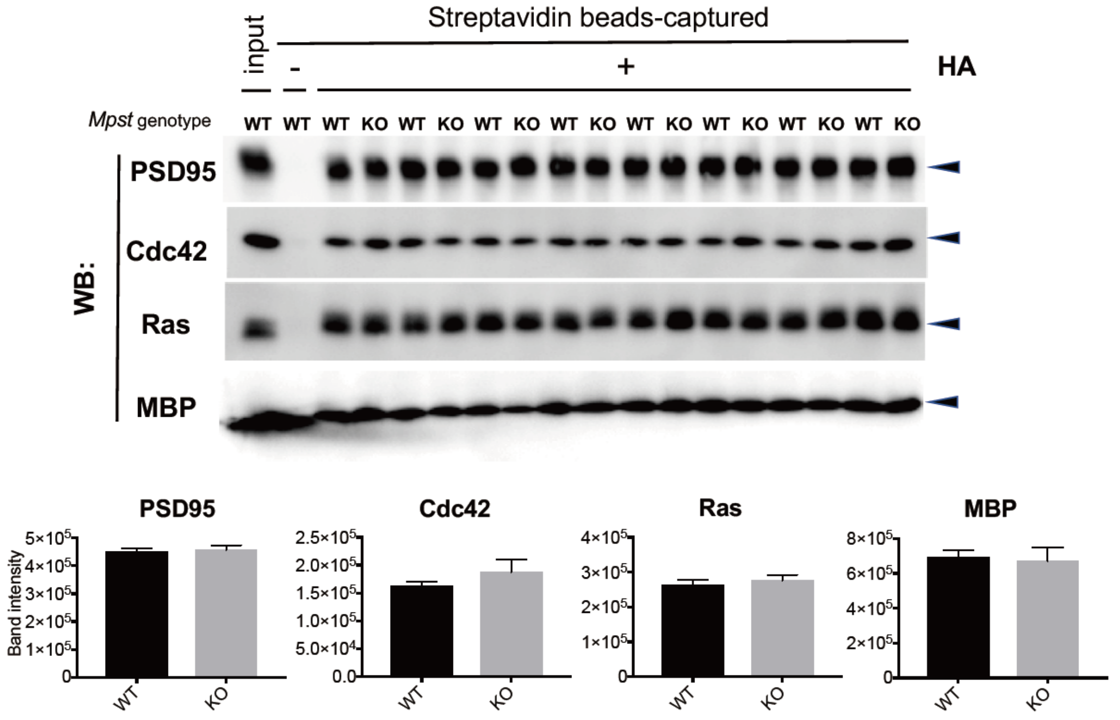

Appendix Figure S9. S-palmitoylation on specific proteins was not affected by a genetic ablation of the *Mpst* gene. A part of the proteins captured by the streptavidin beads (**Appendix Figure S8)** was subjected to conventional western blotting with antibodies against PSD95, Cdc42, Ras and MBP. The data were shown as means ± SEM (n = 8 for each genotype) in the bar graphs.

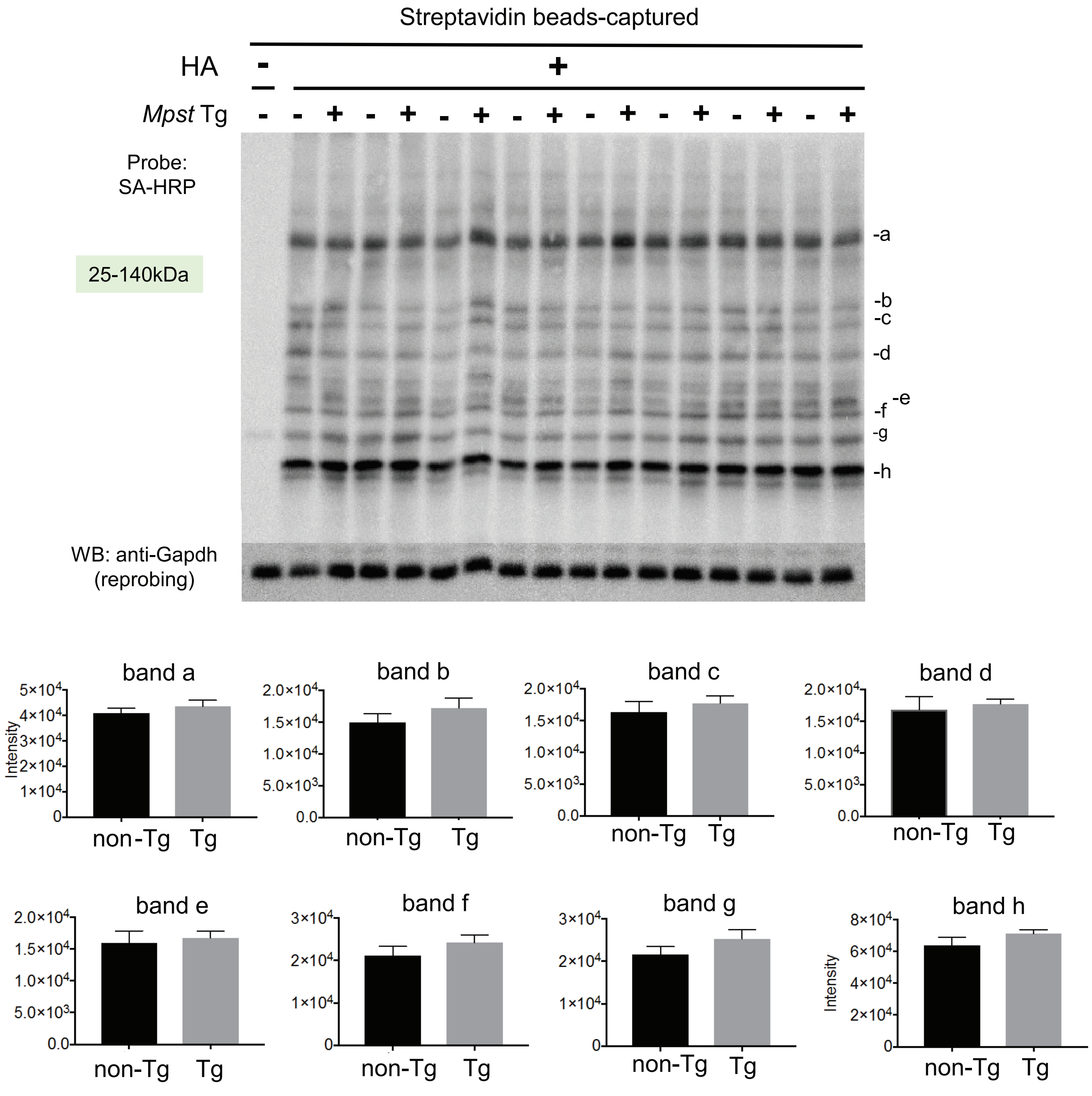

Appendix Figure S10. Global protein S-palmitoylation was not affected by overexpression of the Mpst protein. The experiment was done as in Appendix Figure S8.

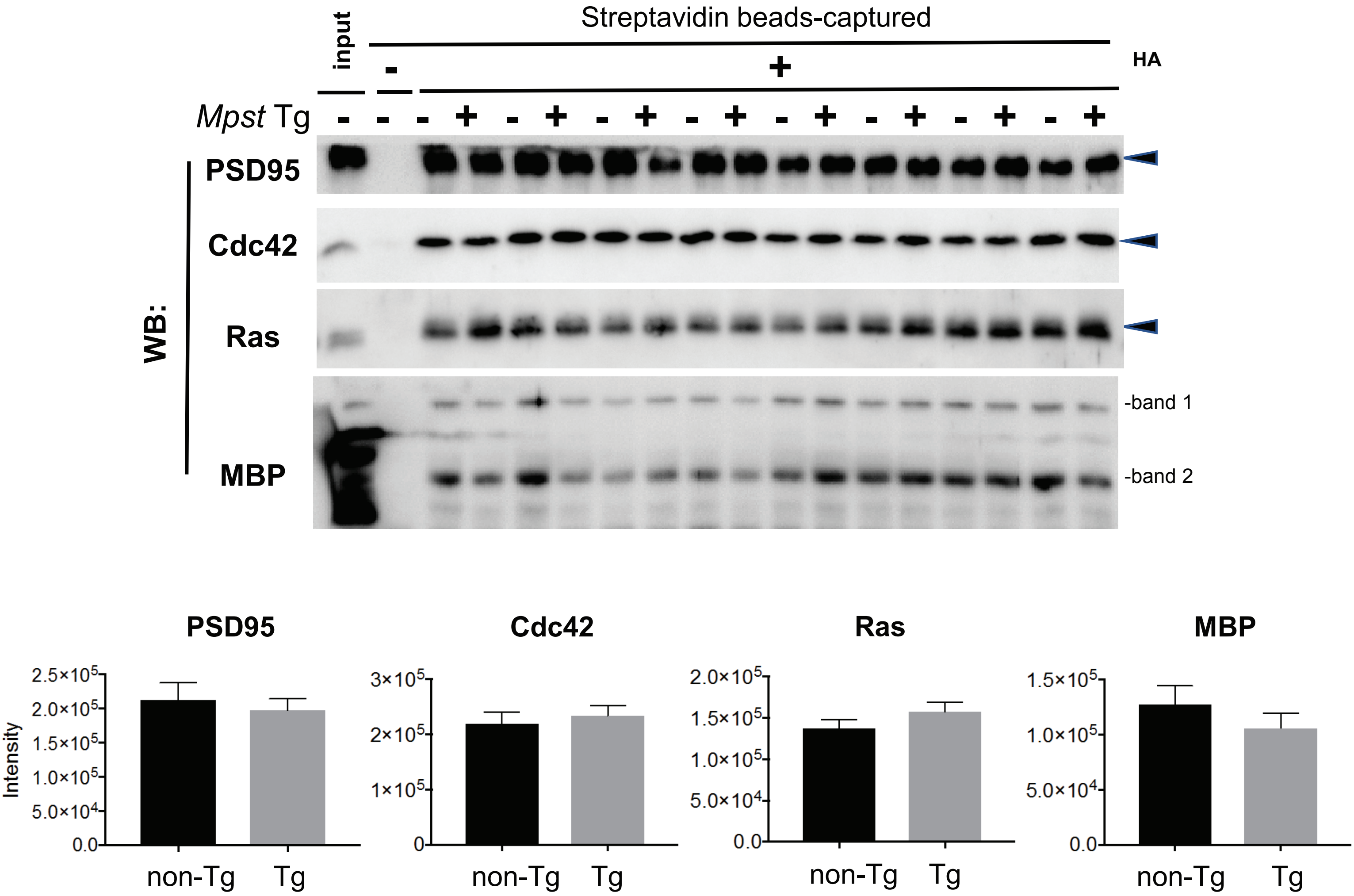

Appendix Figure S11. S-palmitoylation on specific proteins was not affected by overexpression of the Mpst protein. The experiment was done as in Appendix Figure S9.

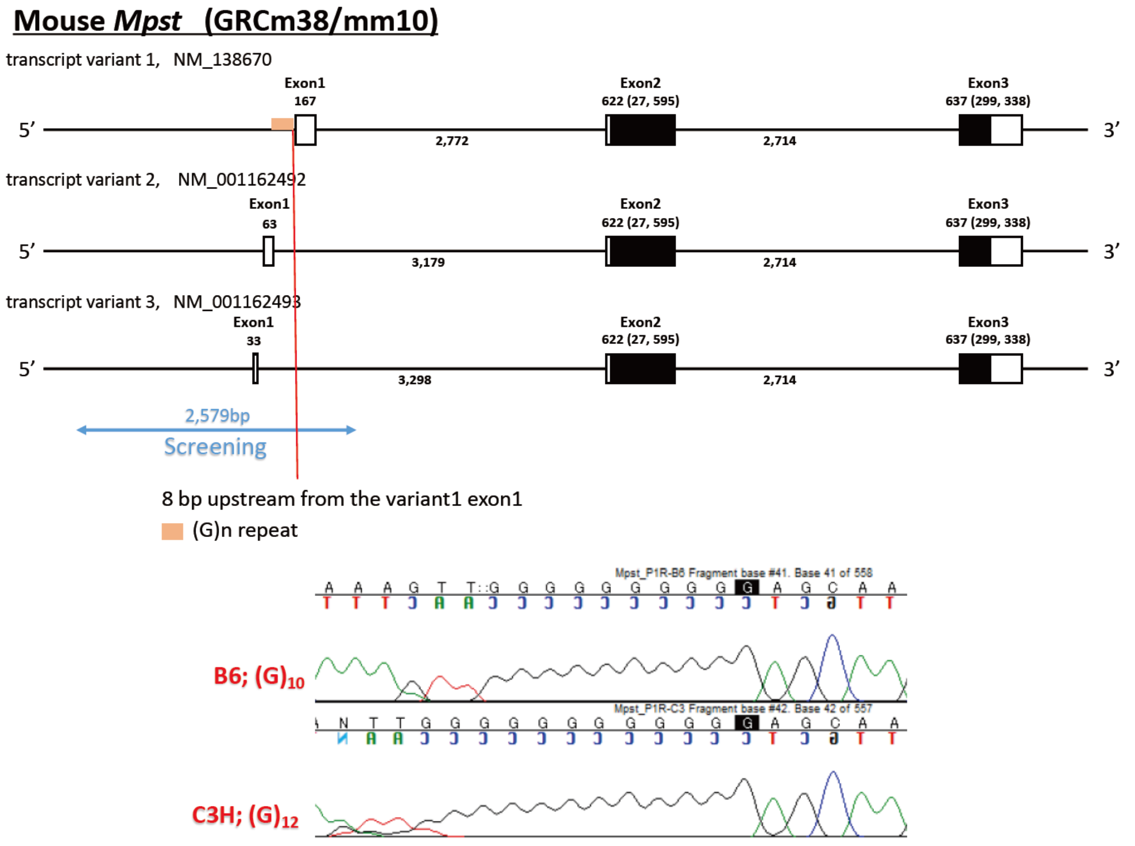

Appendix Figure S12. Three variants of the mouse *Mpst* gene and polymorphic region between the two inbred strains B6 and C3H. The 2.5-kb spanning three independent promoters and corresponding non-coding exon 1 regions was sequenced. The upstream region of exon1 of the variant 1 was found polymorphic.

Mouse *Mpst*

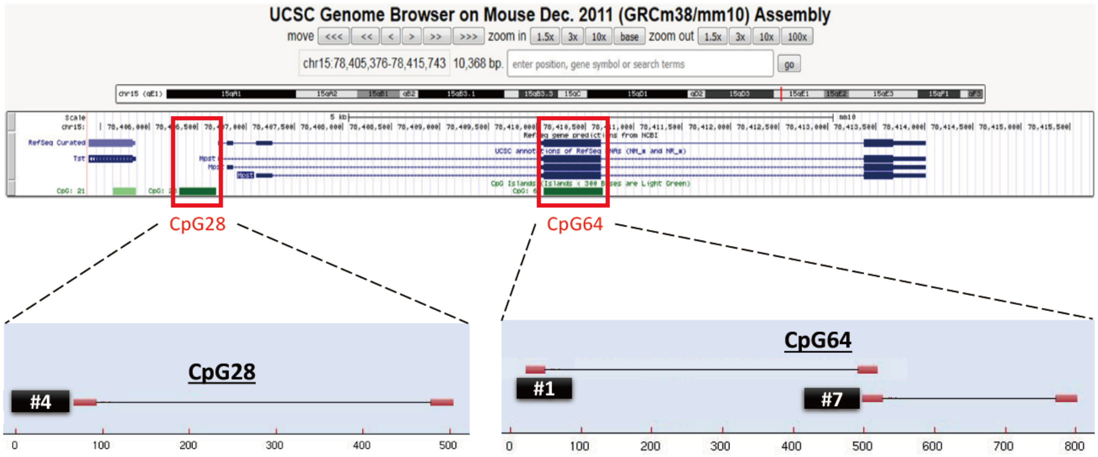

Appendix Figure S13. Two CpG islands at the mouse *Mpst* locus. The mouse *Mpst* locus harbors two CpG islands, CpG28 and CpG64. The positions for the probing intervals are indicated.

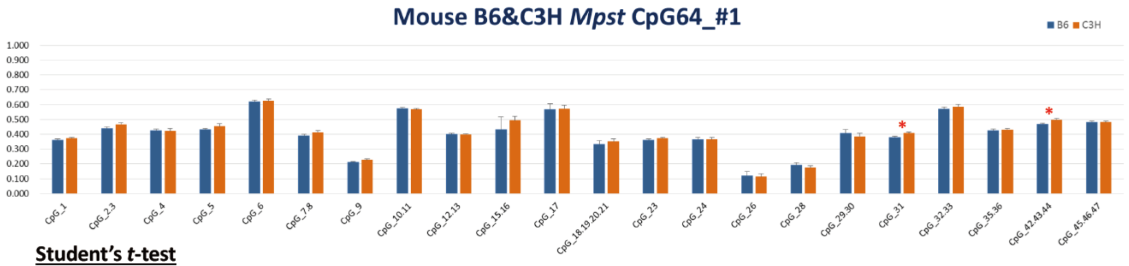

Student's *t*-test

	CpG_1	CpG_2.3	CpG_4	CpG_5	CpG_6	CpG_7.8	CpG_9	CpG_10.11	CpG_12.13	CpG_15.16	CpG_17	CpG_18.19.20.21	CpG_23
P value	0.2249<br>ns	0.1259<br>ns	0.9151<br>ns	0.2324<br>ns	0.8955<br>ns	0.1049<br>ns	0.0522<br>ns	0.6773<br>ns	0.9488<br>ns	0.4361<br>ns	0.8852<br>ns	0.5841<br>ns	0.2249<br>ns

	CpG_24	CpG_26	CpG_28	CpG_29.30	CpG_31	CpG_32.33	CpG_35.36	CpG_42.43.44	CpG_45.46.47
P value	0.9815<br>ns	0.8559<br>ns	0.4383<br>ns	0.5292<br>ns	0.0165<br>*	0.4122<br>ns	0.6289<br>ns	0.0158<br>*	0.9788<br>ns

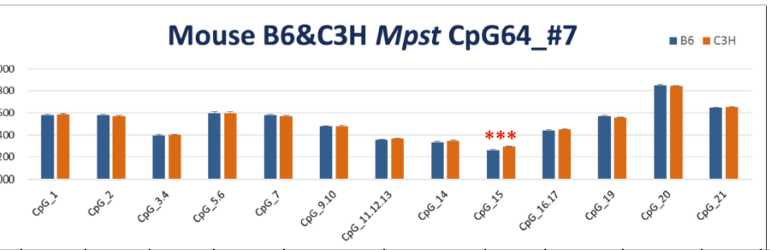

Student's *t*-test

	CpG_1	CpG_2	CpG_3.4	CpG_5.6	CpG_7	CpG_9.10	CpG_11.12.13	CpG_14	CpG_15	CpG_16.17	CpG_19	CpG_20	CpG_21
P value	0.6752<br>ns	0.2722<br>ns	0.6653<br>ns	0.9119<br>ns	0.2722<br>ns	0.7837<br>ns	0.1095<br>ns	0.1053<br>ns	0.0002<br>***	0.3449<br>ns	0.0928<br>ns	0.4439<br>ns	0.468<br>ns

Appendix Figure S14. Comparison of the methylation levels between B6 and C3H. Brains from B6 shows significantly lower methylation levels than those of C3H in CpG_31 and CpG_42.43.44 in the probing interval 1 (top) and CpG_15 in the probing interval 7 (bottom). Data are indicated as mean ± SEM. Student *t*-test, *: $P < 0.05$, ***: $P < 0.001$

Human *MPST*

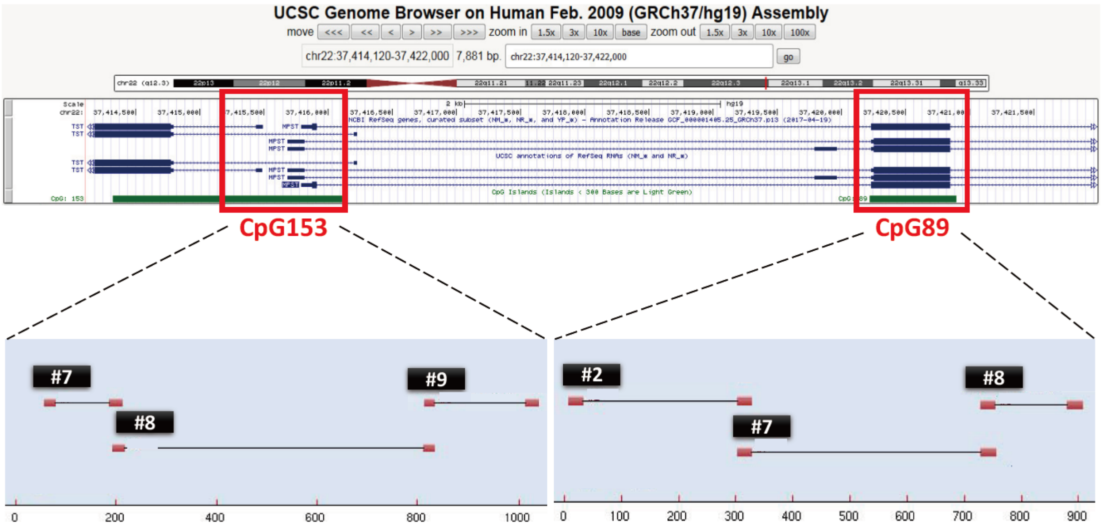

Appendix Figure S15. Two CpG islands at the human *MPST* locus. The human *MPST* locus harbors two CpG islands, CpG153 and CpG89. The positions for the probing intervals are indicated.

Human *CBS*

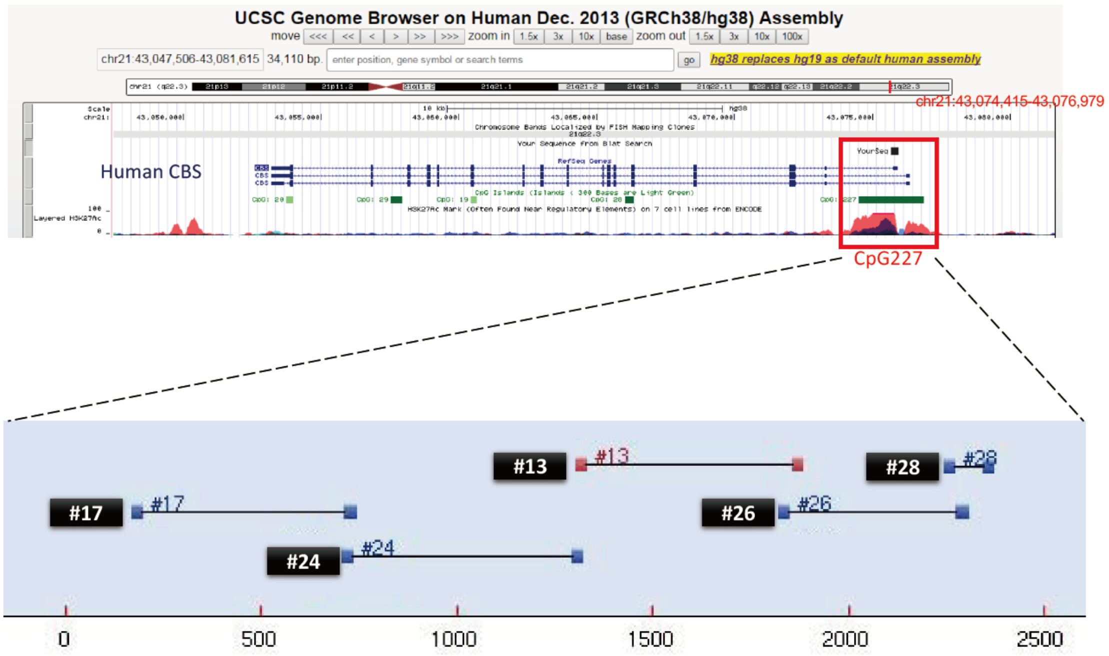

Appendix Figure S16. CpG island at the human *CBS* locus. The human *CBS* locus harbors a CpG island, CpG227. The positions for the probing intervals are indicated.

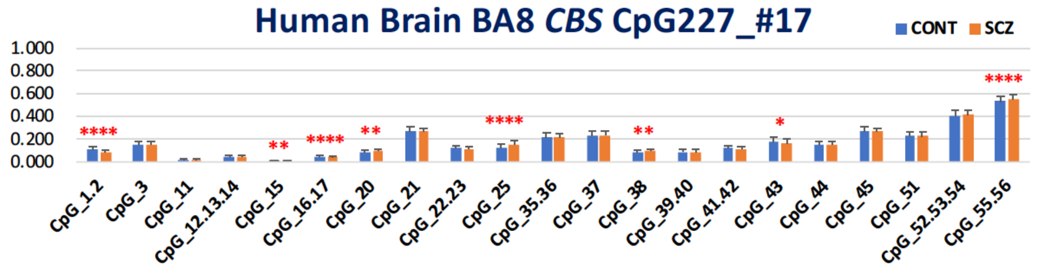

Mann Whitney *U* test

	CpG_1.2	CpG_3	CpG_11	CpG_12.13.14	CpG_15	CpG_16.17	CpG_20	CpG_21	CpG_22.23	CpG_25	CpG_35.36	CpG_37	CpG_38
P value	< 0.0001 ****	0.5844 ns	0.465 ns	0.7418 ns	0.002 **	< 0.0001 ****	0.0013 **	0.8247 ns	0.1912 ns	< 0.0001 ****	0.3546 ns	0.1483 ns	0.0013 **

	CpG_39.40	CpG_41.42	CpG_43	CpG_44	CpG_45	CpG_51	CpG_52.53.54	CpG_55.56
P value	0.6416 ns	0.1912 ns	0.0203 *	0.5844 ns	0.8247 ns	0.7325 ns	0.2131 ns	< 0.0001 ****

Appendix Figure S17. The methylation levels of the *CBS* locus in the postmortem brain from schizophrenia patients. Schizophrenia shows dysregulated methylation levels at the probing interval #17 of CpG227. Data are indicated as mean ± SEM. Student *t*-test, *: $P < 0.05$, **: $P < 0.01$, ***: $P < 0.001$, ****: $P < 0.0001$.

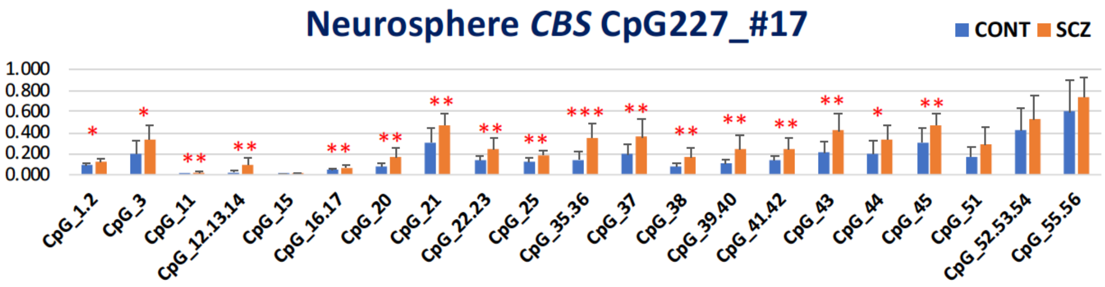

Student's *t*-test

	CpG_1.2	CpG_3	CpG_11	CpG_12.13.14	CpG_15	CpG_16.17	CpG_20	CpG_21	CpG_22.23	CpG_25	CpG_35.36
P value	0.0143<br>*	0.0168<br>*	0.0045<br>**	0.0011<br>**	0.0746<br>ns	0.0075<br>**	0.0043<br>**	0.005<br>**	0.0038<br>**	0.0012<br>**	0.0002<br>***

	CpG_37	CpG_38	CpG_39.40	CpG_41.42	CpG_43	CpG_44	CpG_45	CpG_51	CpG_52.53.54	CpG_55.56
P value	0.0098<br>**	0.0043<br>**	0.0044<br>**	0.0038<br>**	0.0017<br>**	0.0168<br>*	0.005<br>**	0.0565<br>ns	0.2704<br>ns	0.1915<br>ns

Appendix Figure S18. The methylation levels of the *CBS* locus in neurosphere differentiated from schizophrenia patient-specific iPSC. Schizophrenia showed higher methylation levels than control at the probing interval #17 of CpG227. Data are indicated as mean ± SEM. Student *t*-test, *: $P < 0.05$, **: $P < 0.01$, ***: $P < 0.001$, ****: $P < 0.0001$.

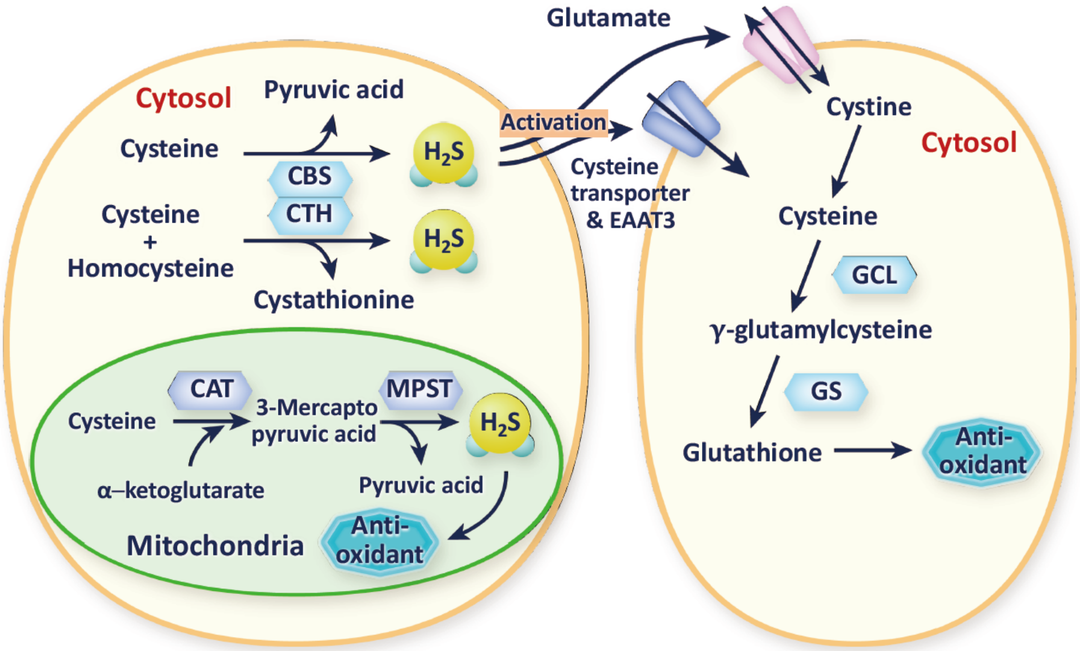

Appendix Figure S19. Oxidative stress and hydrogen sulfide. Cellular glutathione (GSH) is consumed to protect cells from oxidative stress, in turn activating synthesis of hydrogen sulfide and GSH. CBS; cystathionine β-synthase, CTH; cystathionine γ-lyase, MPST; 3-mercaptopruvate sulfurtransferase, CAT; cysteine aminotransferase, GCL; glutamate-cysteine ligase, GS; glutathione synthetase.

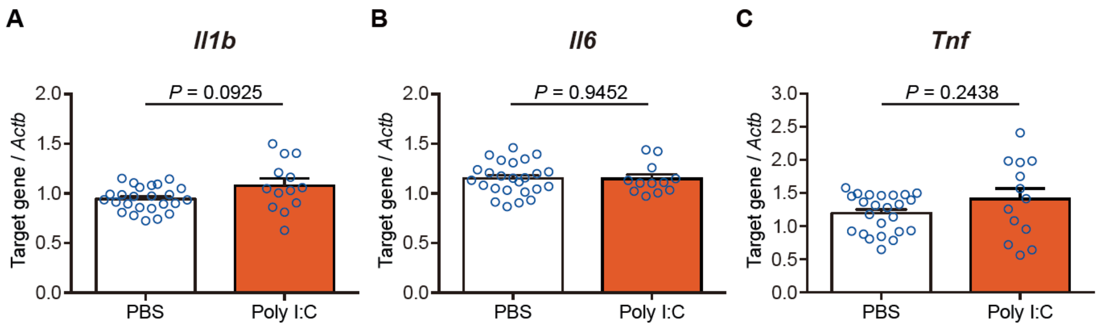

Appendix Figure S20. No upregulation of typical inflammatory gene expression in offspring from the poly-I:C-treated dams at adult stage. The expressions of *Il1b*, *Il6* and *Tnf* in the brain were examined at adult stage. The expression levels of the three genes were normalized to that of *Actb* encoding β actin. Data are indicated as mean ± SEM. *P* value was calculated using unpaired two-tailed *t*-test.

Appendix Table S1. Experimental design for 2D-DIGE.

Gel No.	Cy3		Cy5		Cy2	
	Sample ID	Tissue	Sample ID	Tissue	Sample ID	Tissue
1	B6-1	FC [a]	C3H-1	FC	IS [b]	FC
2	B6-2	FC	C3H-2	FC	IS	FC
3	B6-3	FC	C3H-3	FC	IS	FC
4	B6-4	FC	C3H-4	FC	IS	FC
5	B6-1	Lym [c]	C3H-1	Lym	IS	Lym
6	B6-2	Lym	C3H-2	Lym	IS	Lym
7	B6-3	Lym	C3H-3	Lym	IS	Lym
8	B6-4	Lym	C3H-4	Lym	IS	Lym

[a] FC, Frontal cortex of brain

[b] IS, Internal standard, a mixture of all samples in each tissue

[c] Lym, Spleen-derived lymphocytes

Appendix Table S2. **Differentially expressed proteins between B6 and C3H mice analyzed by 2D-DIGE.**

Tissue	Differentially expressed protein spots [a]			Total number of spots
	B6 > C3H	B6 < C3H	Total	
Brain	18	25	43	1,093
Lymphocytes	72	59	131	1,400
Brain & Lymphocytes	6	10	16	not applicable

[a] B6 > C3 and B6 < C3 signify higher and lower intensities of spots in B6 compared with C3H mice, respectively. Significance was determined by $P < 0.05$ and a fold change > 1.2.

Appendix Table S3. List of identified proteins showing similar alteration.

Spot No.	Protein Identification	Acc. No.	Theo. pI /mass	Tissue	Matched peptides	Sequence coverage	MASCOT score	Peptides						
								Start - End	Observed Mr (expt) [c]	Mr (expt)	Mr (calc)	Delta	Miss	Sequence
1	Heat shock 70 kDa protein 9	A48127	5.81/73701	FC [a]	6	12%	72	188 - 202	1694.95	1693.94	1693.84	0.10	0	NAVITVPAYFNDSQR
								266 - 284	2056.03	2055.02	2054.95	0.07	0	STNGDTFLGGEDFDQALLR
								349 - 360	1333.79	1332.78	1332.73	0.05	0	AQFEGIVTDLIK
								378 - 391	1462.78	1461.77	1461.75	0.02	0	SDIGEVILVGGMTR
								395 - 405	1290.76	1289.75	1289.67	0.08	0	VQQTVQDLFGR
								499 - 513	1593.05	1592.04	1591.94	0.1	0	LLGQFTLIGIPPAPR
				Lym [b]	7	14%	85	160 - 173	1569.87	1568.86	1568.83	0.04	0	LYSPSQIGAFVLMK Oxidation (M)
								188 - 202	1694.94	1693.93	1693.84	0.09	0	NAVITVPAYFNDSQR
								266 - 284	2056.02	2055.01	2054.95	0.06	0	STNGDTFLGGEDFDQALLR
								349 - 360	1333.79	1332.78	1332.73	0.05	0	AQFEGIVTDLIK
								378 - 391	1462.76	1461.75	1461.75	0.00	0	SDIGEVILVGGMTR Oxidation (M)
								395 - 405	1290.78	1289.77	1289.67	0.10	0	VQQTVQDLFGR
								499 - 513	1593.03	1592.02	1591.94	0.08	0	LLGQFTLIGIPPAPR
2	Heat shock 70 kDa protein 9	A48127		FC	6	10%	74	174 – 187	1592.59	1591.58	1591.77	-0.18	1	MKETAENYLGHTAK
								176 – 187	1333.46	1332.45	1332.63	-0.18	0	ETAENYLGHTAK

								188 – 202	1694.43	1693.42	1693.84	-0.42	0	NAVITVPAYFNDSQR
								266 – 284	2056.25	2055.24	2054.95	0.29	0	STNGDTFLGGEDFDQALLR
								378 – 391	1462.42	1461.41	1461.75	-0.34	0	SDIGEVILVGGMTR Oxidation (M)
								395 – 405	1290.5	1289.49	1289.67	-0.18	0	VQQTVQDLFGR
				Lym	6	12%	72	188 - 202	1694.97	1693.96	1693.84	0.12	0	NAVITVPAYFNDSQR
								266 - 284	2056	2054.99	2054.95	0.04	0	STNGDTFLGGEDFDQALLR
								349 - 360	1333.82	1332.81	1332.73	0.08	0	AQFEGIVTDLIK
								378 - 391	1462.86	1461.85	1461.75	0.10	0	SDIGEVILVGGMTR Oxidation (M)
								395 - 405	1290.79	1289.78	1289.67	0.11	0	VQQTVQDLFGR
								499 - 513	1593.03	1592.02	1591.94	0.08	0	LLGQFTLIGIPPAPR
3	Nucleophosmin	Q5SQB0	4.62/32739	FC		-	-							
				Lym	6	25%	68	33 - 45	1568.84	1567.83	1567.72	0.11	0	VDNDENEHQLSLR
								55 - 73	2161.02	2160.01	2160	0.01	0	DELHIVEAEAMNYEGSPIK Oxidation (M)
								81 - 101	2227.23	2226.22	2226.21	0.01	0	MSVQPTVSLGGFEITPPVVLR
								81 - 101	2243.24	2242.23	2242.2	0.03	0	MSVQPTVSLGGFEITPPVVLR Oxidation (M)
								248 - 261	1819.99	1818.98	1818.84	0.15	0	MTDQEAIQDLWQWR
								248 - 261	1835.94	1834.93	1834.83	0.10	0	MTDQEAIQDLWQWR

														Oxidation (M)
4	Nucleophosmin	Q5SQB0	4.62/32739	FC		-	-							
				Lym	7	27%	85	28 - 45	2259.26	2258.25	2258.04	0.22	1	DYHFKVDNDENEHQLSLR
								33 - 45	1568.92	1567.91	1567.72	0.19	0	VDNDENEHQLSLR
								55 - 73	2161.02	2160.01	2160	0.01	0	DELHIVEAEAMNYEGSPIK<br>Oxidation (M)
								81 - 101	2227.23	2226.22	2226.21	0.01	0	MSVQPTVSLGGFEITPPVVLR
								81 - 101	2243.24	2242.23	2242.2	0.03	0	MSVQPTVSLGGFEITPPVVLR<br>Oxidation (M)
								248 - 261	1819.95	1818.94	1818.84	0.11	0	MTDQEAIQDLWQWR
								248 - 261	1835.9	1834.89	1834.83	0.06	0	MTDQEAIQDLWQWR<br>Oxidation (M)
5	3-mercaptopyruvate<br>sulfurtransferase<br>(EC 2.8.1.2)	Q99J99	6.12/33984	FC	4	19%	64	8 – 20	1453.92	1452.91	1452.81	0.10	0	ALVSAQWVAEALK
								52 – 63	1358.83	1357.82	1357.68	0.14	0	HIPGAAFFDIDR
								146 – 163	2058.13	2057.12	2056.99	0.14	0	SHSEPAEFSAQLDPSFIK
								266 – 281	1988.98	1987.97	1987.88	0.10	0	SDVPVYDGSWVEWYMR
				Lym		-	-							
10	Peroxiredoxin 6<br>(EC 1.11.1.9)	O08709	5.72/24838	FC	5	22%	84	1－21	2142.15	2141.14	2141.08	0.07	0	PGGLLLGDEAPNFEANTTIGR
								97 - 105	1057.63	1056.62	1056.59	0.04	0	LPFPIIDDK

								132 - 140	1021.67	1020.66	1020.56	0.10	0	VVFIFGPDK
								132 - 141	1149.72	1148.71	1148.66	0.05	1	VVFIFGPDKK
								144 - 154	1191.8	1190.79	1190.67	0.13	0	K.LSILYPATTGR
				Lym	5	18%	81	41 – 52	1395.77	1394.79	1394.65	0.11	0	DFTPVCTTELGR
								97 – 105	1057.63	1056.62	1056.59	0.04	0	LPFPIIDDK
								132—140	1021.68	1020.67	1020.56	0.11	0	VVFIFGPDK
								132 – 141	1149.76	1148.75	1148.66	0.09	1	VVFIFGPDKK
								144 – 154	1191.69	1190.68	1190.67	0.02	0	LSILYPATTGR
11	Peroxiredoxin 6 (EC 1.11.1.9)	O08709	5.72/24838	FC	5	20%	83	97 – 105	1057.44	1056.43	1056.59	-0.15	0	LPFPIIDDK
								106 – 121	1755.5	1754.49	1754.96	-0.47	1	GRDLAILLGMLDPVEK Oxidation (M)
								108 – 121	1542.51	1541.5	1541.84	-0.33	0	DLAILLGMLDPVEK Oxidation (M)
								132 – 141	1149.52	1148.51	1148.66	-0.15	1	VVFIFGPDKK
								144 – 154	1191.53	1190.52	1190.67	-0.14	0	LSILYPATTGR
				Lym	3	8%	48	97 – 105	1057.59	1056.58	1056.59	-0.00	0	LPFPIIDDK
								132 – 140	1021.59	1020.58	1020.56	0.02	0	VVFIFGPDK
								132 – 141	1149.65	1148.64	1148.66	-0.02	1	VVFIFGPDKK
12	Nucleoside diphosphate kinase B (EC 2.7.4.6)	S29241	6.97/17466	FC	4	32%	72	1 – 18	2059.11	2058.1	2058.1	-0.00	1	MANLERTFIAIKPDGVQR
								7 – 18	1344.85	1343.84	1343.76	0.09	0	TFIAIKPDGVQR
								89 – 105	1802	1800.99	1800.9	0.09	0	VMLGETNPADSKPGTIR

													Oxidation (M)
								129 – 143	1960.07	1959.06	1959.01	0.05	0	EIHLWFKPEELIDYK
				Lym	3	21%	54	1 – 18	2059.13	2058.12	2058.1	0.02	1	MANLERTFIAIKPDGVQR
								7 – 18	1344.85	1343.84	1343.76	0.09	0	TFIAIKPDGVQR
								129 – 143	1960.07	1959.06	1959.01	0.05	0	EIHLWFKPEELIDYK
13	Nucleoside diphosphate kinase B (EC 2.7.4.6)	S29241	6.97/17466	FC	3	24%	52	7 – 18	1344.81	1343.83	1343.76	0.08	0	TFIAIKPDGVQR
								57 – 66	1175.74	1174.73	1174.65	0.08	0	DRPFFPGLVK
								129 – 143	1960.15	1959.14	1959.01	0.13	0	EIHLWFKPEELIDYK
				Lym	3	24%	52	7 – 18	1344.81	1343.8	1343.76	0.05	0	TFIAIKPDGVQR
								57 – 66	1175.73	1174.72	1174.65	0.07	0	DRPFFPGLVK
								129 – 143	1960.11	1959.1	1959.01	0.09	0	EIHLWFKPEELIDYK

[a] FC, Frontal cortex of brain

[b] Lym, Spleen-derived lymphocytes

[c] Mr, Molecular Mass

Appendix Table S4. Identification of proteins showing consistent increases and decreases between brain and lymphocytes derived from B6 and C3H mice by 2D-DIGE.

Spot No.	Protein name	Synonyms	Accession [a]	Theoretical pI /mass [b]	Tissue [c]	Fold Change [d]	%Seq /No. peptides [e]	MASCOT score [f]	Western blot [g]
1	Heat shock 70kDa protein 9 (mortalin)	Mortalin<br>GRP 75<br>Heat shock 70 kDa protein 9<br>PBP74<br>p66 MOT	P38646 (A48127 [h])	5.81 / 73701	FC	-2.61	12% / 6	72	Confirmed 4 spots
					Lym	-3.43	14% / 7	85	
2	Heat shock 70kDa protein 9 (mortalin)				FC	2.91	10% / 6	74	
					Lym	3.01	12% / 6	72	
3	Nucleophosmin (nucleolar phosphoprotein B23, numatrin)	NPM<br>Nucleolar phosphoprotein B23<br>Numatrin<br>Nucleolar protein NO38	Q61937 (Q5SQB0 [h])	4.62 / 32739	FC	-1.42	-	-	Confirmed 3 spots
					Lym	-3.14	22% / 6	66	
4	Nucleophosmin (nucleolar phosphoprotein B23, numatrin)				FC	2.75	-	-	
					Lym	6.4	24% / 7	83	
5	Mercaptopyruvate sulfurtransferase	MST<br>EC 2.8.1.2	Q99J99	6.12 / 33984	FC	5.62	19% / 4	64	Confirmed 1 spot
					Lym	1.59	-	-	
10	Peroxiredoxin 6	EC 1.11.1.15<br>Antioxidant protein 2<br>1-Cys peroxiredoxin<br>Acidic calcium- independent phospholipase A2	O08709	5.72 / 24838	FC	7.33	22% / 5	84	Confirmed 2 spots
					Lym	4.84	18% / 4	65	
11	Peroxiredoxin 6				FC	-4.2	20% / 5	83	
					Lym	-2.88	8% / 3	48	
12	NME/NM23 nucleoside diphosphate kinase 2	NDK B<br>EC 2.7.4.6<br>NDP kinase B<br>nm23-H2<br>C-myc purine-binding transcription factor PUF	Q01768 (S29241 [h])	6.97 / 17466	FC	4.32	32% / 4	72	Confirmed 1 spot
					Lym	2.92	21% / 3	54	
13	NME/NM23 nucleoside diphosphate kinase 2				FC	-3.81	24% / 3	52	
					Lym	-3.96	24% / 3	52	

[a] UniProt accession

[b] Theoretical pI and mass were calculated according to the sequence

[c] FC, Frontal cortex of brain; Lym, lymphocytes

[d] Minus denotes down-regulation in C3H compared to B6 mice

[e] %Seq and No. peptides denotes percentage of sequence coverage and the number of peptides identified by PMF, respectively.

[f] Protein Identification from peptide mass spectra were performed using MASCOT

[g] Confirmed signifies spots in 2D-DIGE that were identical or included in the spots detected by western blotting. Indicated number of spot is the number of spots, showing identical molecular mass and different pI values, detected in western blotting

[h] MASCOT referred to unreviewed sequences

Appendix Table S5. Genetic variations detected in the coding regions of proteins expressed differentially between B6 and C3H mice.

Protein	Gene	dbSNP	SNP position [a]	Variation		Number of Phosphates	pI [b]		Molecular mass [b]	
				B6	C3H		B6	C3H	B6	C3H
Mortalin	*Hspa9*	rs13467546	1852 bp	ATG (Met)	GTG (Val)	0	5.81	5.91	73,460	73,529
						1	5.72	5.81	73,538	73,607
		rs8255019	1870 bp	GGA (Gly)	CGA (Arg)	2	5.63	5.71	73,616	73,684
						3	5.56	5.63	73,694	73,762
Nucleophosmin	*Npm1*	ss410758760	526-537 bp	GATGATGATGAT (AspAspAspAsp)	deletion	0	4.62	4.73	32,568	32,111
						1	4.59	4.69	32,646	32,189
						2	4.56	4.66	32,724	32,267
						3	4.53	4.62	32,802	32,345
MST	*Mpst*	rs31781085	305 bp	GAC (Asp102)	GGC (Gly102)	-	6.00	6.11	33,081	33,023
Peroxiredoxin-6	*Prdx6*	rs13474944	371 bp	GCT (Ala)	GAT (Asp)	-	5.98	5.71	24,827	24,870
NDK B	*Nme2*	rs28260296	168 bp	AAA (Lys)	AAC (Asn)	-	6.97	6.52	17,368	17,385
		ss410758759	217 bp	GTG (Val)	ATG (Met)					

[a] UCSC Genome Bioinformatics (http://genome.ucsc.edu/cgi-bin/hgGateway) version Feb. 2006

[b] Theoretical pI and molecular mass are calculated according to the amino acid sequence of each protein by using Scansite (http://scansite.mit.edu/calc_mw_pi.html)

Appendix Table S6. Enzymatic activity of Mpst from B6 and C3H mouse brains.

	**Km (mM) (mean ± SEM)**	***P* value**	**Vmax (μmole/min x mg protein) (mean ± SEM)**	***P* value**
B6	3.397 ± 0.309	0.678	7.182 ± 1.461	0.861
C3H	3.817 ± 0.862		7.714 ± 2.380	

P value was calculated by Student's *t*-test (two-tailed)

Appendix Table S7. Demographic characteristics of postmortem brains (1st set).

	BA8		*P* value
	**Control**	**Schizophrenia**	
N	93	95	
Age at death (mean ± SD)	47.89 ± 16.62	45.92 ± 17.37	0.4281 [a]
Sex (female/male)	20 / 73	25 / 70	0.4396 [b]
Postmortem interval (hours)	41.78	41.71	0.9725 [a]
Brain tissue pH (mean ± SD)	6.33 ± 0.21	6.26 ± 0.25	0.0392 [a]

[a] Evaluated by Mann-Whitney *U* test (two-tailed)

[b] Evaluated by Fisher's exact test

Appendix Table S8. Demographic characteristics of postmortem brains (2nd set).

	BA17		*P* value
	**Control**	**Schizophrenia**	
N	22	14	
Age at death (mean ± SD)	63.05 ± 16.81	65.79 ± 12.07	0.9936 [a]
Sex (female/male)	8 / 14	5 / 9	0.9685 [b]
Postmortem interval (hours)	7.65	13.40	0.0055 [a]
Brain tissue pH (mean ± SD)	6.33 ± 0.35	6.46 ± 0.40	0.5395 [a]

[a] Evaluated by Mann-Whitney *U* test (two-tailed)

[b] Evaluated by Fisher's exact test

Appendix Table S9. Demographic characteristics of peripheral blood sample set for *MPST* / MPST expression analyses.

	Control	Schizophrenia	*P* value
N	56	44	
Sex (female/male)	26 / 30	21 / 23	1.00 [a]
Age (mean ± SD)	46.8 ± 13.3	49.2 ± 2.0	0.41[b]

[a] Evaluated by Fisher's exact test

[b] Evaluated by Mann-Whitney's *U* test (two-tailed)

Appendix Table S10. Demographic characteristics of hair follicle sample set.

	**Control**	**Schizophrenia**	***P* value**
N	166	149	
Sex (female/male)	105 / 61	67 / 82	0.0011[a]
Age (mean ± SD)	43.72 ± 14.02	46.58 ± 11.67	0.0515[b]

[a] Evaluated by Fisher's exact test

[b] Evaluated by Mann-Whitney's *U* test (two-tailed)

Appendix Table S11. Differentially expressed genes from the frontal cortex of *Mpst* KO mouse.

Refseq_ID	Gene	Log_2 fold change	*P* value	Regulation
NM_001285913	*Cdh8*	-6.23587353	0.014846654	Mpst_KO_downregulated
NM_001302258	*Pmp22*	-6.105512243	0.026291167	Mpst_KO_downregulated
NM_153089	*Ppp1r16b*	-6.102283612	0.001963833	Mpst_KO_downregulated
NM_177942	*Ece2*	-5.992666442	0.026775943	Mpst_KO_downregulated
NM_001159641	*Tasp1*	-5.442536439	0.045575681	Mpst_KO_downregulated
NM_001130184	*Jade1*	-4.639623752	0.037046995	Mpst_KO_downregulated
NM_001177937	*Eda*	-4.321802399	0.049442503	Mpst_KO_downregulated
NR_073122	*Ube2w*	-1.362218267	0.015523558	Mpst_KO_downregulated
NM_001276443	*Gpr155*	-1.177181124	0.029453946	Mpst_KO_downregulated
NM_001164402	*Ptpro*	-1.173565019	0.009829531	Mpst_KO_downregulated
NM_144863	*Wdr36*	-1.133053829	0.024179137	Mpst_KO_downregulated
NM_001271390	*Ankra2*	-1.120801588	0.017103603	Mpst_KO_downregulated
NM_001286382	*Vkorc1l1*	-1.013175544	0.021788154	Mpst_KO_downregulated
NM_009021	*Rai1*	-1.003560686	0.006630548	Mpst_KO_downregulated
NR_040592	*1700030L20Rik*	-0.992903115	0.01340516	Mpst_KO_downregulated
NM_001164818	*Aar2*	-0.981498949	0.045486891	Mpst_KO_downregulated
NM_001290381	*Tfpt*	-0.980082104	0.006232284	Mpst_KO_downregulated
NM_001166503	*Slc39a11*	-0.968001398	0.021339603	Mpst_KO_downregulated
NM_001162493	*Mpst*	-0.957182679	0.000482652	Mpst_KO_downregulated
NM_026531	*Aen*	-0.928076105	0.008497835	Mpst_KO_downregulated
NR_033133	*Plscr3*	-0.848688738	0.022435396	Mpst_KO_downregulated
NM_001293791	*Ints10*	-0.838876516	0.005428147	Mpst_KO_downregulated
NM_001177464	*Zfp516*	-0.814571084	0.034663206	Mpst_KO_downregulated
NM_009771	*Btrc*	-0.791687273	0.033366242	Mpst_KO_downregulated
NM_001001565	*Chpf*	-0.771705576	0.025289854	Mpst_KO_downregulated
NM_001001177	*BC051142*	-0.770299625	0.008583959	Mpst_KO_downregulated
NM_145502	*Erlin1*	-0.758629853	0.025125398	Mpst_KO_downregulated
NM_001085417	*Zfp467*	-0.755212502	0.004085676	Mpst_KO_downregulated
NM_001290646	*Lhx2*	-0.734263501	0.007293286	Mpst_KO_downregulated
NM_001291120	*Epb4.1l1*	-0.711761529	0.039330815	Mpst_KO_downregulated
NM_001285455	*Mapt*	-0.698793497	0.032504362	Mpst_KO_downregulated
NM_021402	*Ube2j2*	-0.687852525	0.011627977	Mpst_KO_downregulated
NM_001290759	*Tbc1d9b*	-0.686859086	0.047629404	Mpst_KO_downregulated
NM_001109748	*Tomm40*	-0.654256076	0.022642086	Mpst_KO_downregulated
NR_027887	*9430008C03Rik*	-0.637885147	0.047377227	Mpst_KO_downregulated
NM_175411	*Fam118b*	-0.627265302	0.041810445	Mpst_KO_downregulated
NM_177592	*Tmem164*	-0.610255872	0.010807102	Mpst_KO_downregulated
NM_001159717	*Sept2*	-0.60899063	0.012974108	Mpst_KO_downregulated
NM_172781	*Klhl4*	-0.607654397	0.028538314	Mpst_KO_downregulated
NM_001252522	*Picalm*	-0.602385072	0.025739071	Mpst_KO_downregulated
NM_001159537	*Adcy3*	-0.569844121	7.69497E-05	Mpst_KO_downregulated
NM_001042613	*Sepp1*	-0.556715013	0.026151476	Mpst_KO_downregulated
NR_028094	*Rabggtb*	-0.542969103	0.01357672	Mpst_KO_downregulated
NM_001145958	*Crocc*	-0.540718719	0.005230673	Mpst_KO_downregulated
NR_034030	*Lins*	-0.530655117	0.038326974	Mpst_KO_downregulated
NM_010135	*Enah*	-0.522406525	0.004745659	Mpst_KO_downregulated
NM_001164793	*Polr2m*	-0.520998789	0.021607327	Mpst_KO_downregulated
NM_025482	*Tpd52l2*	-0.520629766	0.044386335	Mpst_KO_downregulated
NM_001190718	*Dcaf12l1*	-0.519220739	0.041296047	Mpst_KO_downregulated
NM_008006	*Fgf2*	-0.504643013	0.021896091	Mpst_KO_downregulated
NM_001243192	*Serpinb6a*	-0.492700083	0.04462934	Mpst_KO_downregulated
NM_001177629	*Grb10*	-0.484762708	0.020656967	Mpst_KO_downregulated
NM_001113545	*Lima1*	-0.47809486	0.02437217	Mpst_KO_downregulated
NM_030017	*Rdh12*	-0.476251096	0.032369638	Mpst_KO_downregulated
NM_001164210	*Sptssb*	-0.455980547	0.01040194	Mpst_KO_downregulated
NM_001135019	*Zfp266*	-0.455430332	0.041127765	Mpst_KO_downregulated
NM_001193309	*Morc4*	-0.453968123	0.019745648	Mpst_KO_downregulated

NR_028364	*AI450353*	-0.441123243	0.015359092	Mpst_KO_downregulated
NM_010160	*Celf2*	-0.437520424	0.022036306	Mpst_KO_downregulated
NM_009614	*Adam15*	-0.434161104	0.034528361	Mpst_KO_downregulated
NM_145841	*Sgcz*	-0.430360252	0.018260172	Mpst_KO_downregulated
NM_001201389	*Gm11128*	-0.427298741	0.022202865	Mpst_KO_downregulated
NM_026563	*Sdccag3*	-0.423154666	0.047799568	Mpst_KO_downregulated
NM_018887	*Cyp39a1*	-0.421058978	0.039841216	Mpst_KO_downregulated
NM_001242944	*Gm14306*	-0.419671136	0.00224154	Mpst_KO_downregulated
NM_177025	*Cobll1*	-0.415225995	0.033525279	Mpst_KO_downregulated
NM_001286986	*Dram2*	-0.409485571	0.046709736	Mpst_KO_downregulated
NM_001168516	*Zdhhc24*	-0.403223036	0.030945381	Mpst_KO_downregulated
NM_024464	*Pigx*	-0.399626329	0.029431399	Mpst_KO_downregulated
NM_001286468	*Arhgap24*	-0.398083403	0.041776124	Mpst_KO_downregulated
NR_103516	*Ndufa7*	-0.390041618	0.020352604	Mpst_KO_downregulated
NM_001159610	*Lrrc57*	-0.387085361	0.005125789	Mpst_KO_downregulated
NM_011665	*Ube2i*	-0.386684985	0.044724301	Mpst_KO_downregulated
NM_153787	*Bclaf1*	-0.384267593	0.014822288	Mpst_KO_downregulated
NR_033121	*Gm10409*	-0.372236304	0.040792895	Mpst_KO_downregulated
NM_172485	*Thsd7b*	-0.369527317	0.046749974	Mpst_KO_downregulated
NR_033121	*Gm10409*	-0.368911	0.042190381	Mpst_KO_downregulated
NM_001163579	*Lrrcc1*	-0.368139183	0.024334664	Mpst_KO_downregulated
NM_001190385	*Caly*	-0.367647337	0.039875471	Mpst_KO_downregulated
NM_176839	*Abcc5*	-0.3608101	0.040074896	Mpst_KO_downregulated
NM_177786	*Depdc5*	-0.356018236	0.00689278	Mpst_KO_downregulated
NR_033198	*2700046G09Rik*	-0.355362997	0.007595826	Mpst_KO_downregulated
NM_001290734	*Osbpl6*	-0.351549636	0.040349095	Mpst_KO_downregulated
NM_001270842	*Gm3558*	-0.333004397	0.030668194	Mpst_KO_downregulated
NR_045297	*Gm17644*	-0.327078574	0.045876274	Mpst_KO_downregulated
NM_001033148	*1700029J07Rik*	-0.325335147	0.009462913	Mpst_KO_downregulated
NM_001166360	*6030458C11Rik*	-0.314385605	0.017396037	Mpst_KO_downregulated
NM_170671	*Mycbpap*	-0.311594642	0.025115027	Mpst_KO_downregulated
NM_001163473	*1810010H24Rik*	-0.307962095	0.044218927	Mpst_KO_downregulated
NM_001082412	*Slc25a53*	-0.304838389	0.0423498	Mpst_KO_downregulated
NM_173187	*2310035C23Rik*	-0.303350163	0.032227654	Mpst_KO_downregulated
NM_001302443	*Cdc37l1*	-0.297180229	0.043541264	Mpst_KO_downregulated
NM_001256065	*Gm5141*	-0.296751606	0.001658411	Mpst_KO_downregulated
NM_013649	*Ryk*	-0.295909516	0.036671804	Mpst_KO_downregulated
NM_001171801	*Triqk*	-0.294216765	0.035938819	Mpst_KO_downregulated
NM_001285427	*Cacnb4*	-0.29331462	0.035090145	Mpst_KO_downregulated
NM_011972	*Poli*	-0.288374617	0.025606733	Mpst_KO_downregulated
NM_144792	*Sgms1*	-0.286165509	0.029220924	Mpst_KO_downregulated
NM_001163025	*Mon2*	-0.282617202	0.040683824	Mpst_KO_downregulated
NM_001114679	*9930111J21Rik1*	-0.269418064	0.006200964	Mpst_KO_downregulated
NM_134074	*Dock9*	-0.266261871	0.007677532	Mpst_KO_downregulated
NR_073587	*Tysnd1*	-0.258745256	0.026634559	Mpst_KO_downregulated
NM_172600	*Tmem260*	-0.25866209	0.008187218	Mpst_KO_downregulated
NR_037988	*Gm16701*	-0.257503403	0.02509985	Mpst_KO_downregulated
NM_001038696	*Rnpc3*	-0.256127248	0.036172066	Mpst_KO_downregulated
NM_172126	*Adam1a*	-0.25390598	0.042386615	Mpst_KO_downregulated
NM_001285827	*Ppil3*	-0.250201833	0.035581637	Mpst_KO_downregulated
NM_001163522	*Emcn*	-0.250150419	0.001095816	Mpst_KO_downregulated
NM_183185	*Zfp300*	-0.248133355	0.041938807	Mpst_KO_downregulated
NM_001093775	*Myt1l*	-0.24466743	0.010145872	Mpst_KO_downregulated
NM_026507	*Zwilch*	-0.243210107	0.026360227	Mpst_KO_downregulated
NM_152801	*Arhgef6*	-0.241669848	0.02737191	Mpst_KO_downregulated
NM_178164	*Ptbp3*	-0.236806431	0.023613513	Mpst_KO_downregulated
NM_019829	*Stx5a*	-0.236093819	0.009582653	Mpst_KO_downregulated
NM_001164676	*Zfp229*	-0.235434462	0.034292206	Mpst_KO_downregulated
NM_001033123	*Gm14288*	-0.231812079	0.014475971	Mpst_KO_downregulated
NM_001001333	*Hexdc*	-0.23124103	0.023977971	Mpst_KO_downregulated
NM_026041	*Rrp15*	-0.229935376	0.032536158	Mpst_KO_downregulated
NM_001048204	*Zfp455*	-0.227833946	0.03651193	Mpst_KO_downregulated

NM_023873	*Cep70*	-0.225246758	0.009804271	Mpst_KO_downregulated
NM_001033123	*Gm14288*	-0.221154965	0.021165518	Mpst_KO_downregulated
NM_025465	*Tma16*	-0.220748084	0.005647554	Mpst_KO_downregulated
NR_015505	*Firre*	-0.220041827	0.044193512	Mpst_KO_downregulated
NM_001205371	*Casc4*	-0.21974807	0.038289415	Mpst_KO_downregulated
NM_001284504	*Cask*	-0.218611723	0.045972619	Mpst_KO_downregulated
NM_017368	*Celf1*	-0.218428959	0.012692375	Mpst_KO_downregulated
NM_175193	*Golim4*	-0.216055966	0.041698004	Mpst_KO_downregulated
NM_030715	*Polh*	-0.214793575	0.015772793	Mpst_KO_downregulated
NM_001159290	*Bcl11a*	-0.21106365	0.015556406	Mpst_KO_downregulated
NM_133240	*Acot8*	-0.21039478	0.041769726	Mpst_KO_downregulated
NM_053263	*Hnrnpa3*	-0.207730692	0.027511711	Mpst_KO_downregulated
NM_008921	*Prim1*	-0.206398303	0.047317316	Mpst_KO_downregulated
NM_001164254	*Tpm1*	-0.201216111	0.046702924	Mpst_KO_downregulated
NM_177429	*Ofd1*	-0.198419366	0.048912534	Mpst_KO_downregulated
NM_001174074	*Celf4*	-0.197967834	0.028028356	Mpst_KO_downregulated
NM_001081009	*Parp8*	-0.196875592	0.013033261	Mpst_KO_downregulated
NM_001256380	*Prdm2*	-0.196176376	0.024153253	Mpst_KO_downregulated
NM_001033123	*Gm14288*	-0.188686979	0.020511325	Mpst_KO_downregulated
NM_026467	*Rps27l*	-0.188171982	0.016639667	Mpst_KO_downregulated
NM_178337	*Tbce*	-0.187974846	0.015139522	Mpst_KO_downregulated
NM_010933	*Nppc*	-0.187290694	0.04632846	Mpst_KO_downregulated
NM_007616	*Cav1*	-0.185553928	0.046270899	Mpst_KO_downregulated
NM_001045525	*Cyb5d1*	-0.183061149	0.049611441	Mpst_KO_downregulated
NM_173022	*BC048403*	-0.176713591	0.026816439	Mpst_KO_downregulated
NR_030708	*6820431F20Rik*	-0.174340481	0.016715898	Mpst_KO_downregulated
NM_025716	*Spryd4*	-0.174044531	0.048916111	Mpst_KO_downregulated
NM_001290741	*Cnot6*	-0.172631269	0.034572	Mpst_KO_downregulated
NM_019864	*Atr*	-0.172389604	0.009559364	Mpst_KO_downregulated
NM_175277	*Bola3*	-0.171274667	0.046284542	Mpst_KO_downregulated
NM_001290414	*Fgf13*	-0.16977547	0.047300181	Mpst_KO_downregulated
NM_001284373	*Atf2*	-0.166616005	0.024718468	Mpst_KO_downregulated
NM_145618	*Ice2*	-0.166291129	0.017107267	Mpst_KO_downregulated
NM_028479	*Mrgbp*	-0.165589819	0.035217685	Mpst_KO_downregulated
NM_001243908	*Zfp383*	-0.165093033	0.025421922	Mpst_KO_downregulated
NM_178643	*C230052I12Rik*	-0.162241421	0.027688055	Mpst_KO_downregulated
NM_145960	*Mtrf1*	-0.161470817	0.023847837	Mpst_KO_downregulated
NM_022986	*Irak1bp1*	-0.159557531	0.00852654	Mpst_KO_downregulated
NM_173406	*Jazf1*	-0.157652625	0.002049917	Mpst_KO_downregulated
NM_013851	*Abca8b*	-0.156682996	0.044288179	Mpst_KO_downregulated
NM_025380	*Eef1e1*	-0.155773609	0.011183076	Mpst_KO_downregulated
NM_001081252	*Uggt2*	-0.152676162	0.017439817	Mpst_KO_downregulated
NM_001160222	*Pum2*	-0.152325734	0.00959215	Mpst_KO_downregulated
NM_019653	*Wsb1*	-0.152049509	0.018981255	Mpst_KO_downregulated
NM_175313	*Eogt*	-0.144385513	0.047197553	Mpst_KO_downregulated
NM_001142963	*Gm10778*	-0.142388809	0.020657994	Mpst_KO_downregulated
NM_026810	*Mlh1*	-0.140941408	0.035161526	Mpst_KO_downregulated
NM_008861	*Pkd2*	-0.140848551	0.038025044	Mpst_KO_downregulated
NM_145607	*Ttc13*	-0.140596036	0.020132303	Mpst_KO_downregulated
NM_001285901	*Eri3*	-0.140512762	0.036494654	Mpst_KO_downregulated
NM_001110017	*Dzip3*	-0.139860574	0.043456893	Mpst_KO_downregulated
NM_178411	*Zfp334*	-0.139593458	0.014716434	Mpst_KO_downregulated
NM_133786	*Smc4*	-0.139433007	0.014708963	Mpst_KO_downregulated
NM_026331	*Slc25a37*	-0.138532187	0.02736555	Mpst_KO_downregulated
NM_175175	*Plekhf2*	-0.138287223	0.035192832	Mpst_KO_downregulated
NM_021540	*Rnf130*	-0.137943545	0.026245889	Mpst_KO_downregulated
NM_027869	*Pnpt1*	-0.136782789	0.001172854	Mpst_KO_downregulated
NM_133691	*Puf60*	-0.136047018	0.043862697	Mpst_KO_downregulated
NM_145125	*Brwd1*	-0.13564318	0.003690863	Mpst_KO_downregulated
NM_153552	*Thoc1*	-0.13460342	0.012963656	Mpst_KO_downregulated
NM_001199485	*4931406C07Rik*	-0.13283695	0.021787796	Mpst_KO_downregulated
NM_173760	*Ppip5k2*	-0.130526531	0.015161102	Mpst_KO_downregulated

NM_173765	*Aasdh*	-0.130201815	0.044368895	Mpst_KO_downregulated
NM_026604	*Fam135a*	-0.130110923	0.036322907	Mpst_KO_downregulated
NM_001252209	*Kcnj16*	-0.126957104	0.026391106	Mpst_KO_downregulated
NM_134071	*Ankrd32*	-0.126006614	0.037241081	Mpst_KO_downregulated
NM_010067	*Trdmt1*	-0.125874793	0.016333455	Mpst_KO_downregulated
NM_024270	*Stard3nl*	-0.125782653	0.010218188	Mpst_KO_downregulated
NM_009383	*Tial1*	-0.125544551	0.02937188	Mpst_KO_downregulated
NM_011419	*Kdm5d*	-0.125499925	0.007184957	Mpst_KO_downregulated
NM_172713	*Sdad1*	-0.125016677	0.046455804	Mpst_KO_downregulated
NM_008577	*Slc3a2*	-0.124681526	0.02298314	Mpst_KO_downregulated
NM_033476	*Tfcp2*	-0.124013499	0.026491301	Mpst_KO_downregulated
NM_175552	*Wdr3*	-0.123208555	0.042534818	Mpst_KO_downregulated
NM_001167988	*Taf9b*	-0.12202086	0.029495348	Mpst_KO_downregulated
NM_001007575	*Zfp58*	-0.119812324	0.041834966	Mpst_KO_downregulated
NR_033535	*Gm10845*	-0.119047526	0.039050876	Mpst_KO_downregulated
NM_029344	*Acyp2*	-0.11856191	0.037201488	Mpst_KO_downregulated
NM_144925	*Tnrc6a*	-0.117183459	0.046885785	Mpst_KO_downregulated
NM_001162945	*Mtx3*	-0.116675911	0.013682705	Mpst_KO_downregulated
NM_025748	*Adat2*	-0.114738626	0.045939113	Mpst_KO_downregulated
NM_175090	*Slc31a1*	-0.112145508	0.042682666	Mpst_KO_downregulated
NM_012061	*Cadps*	-0.11097458	0.029152134	Mpst_KO_downregulated
NM_173444	*Nbeal1*	-0.110192054	0.031920796	Mpst_KO_downregulated
NM_013784	*Pign*	-0.106145692	0.046973421	Mpst_KO_downregulated
NM_138756	*Slc25a36*	-0.105543764	0.022920203	Mpst_KO_downregulated
NM_178625	*Tmem209*	-0.103623388	0.018326773	Mpst_KO_downregulated
NM_028315	*Dis3*	-0.103506871	0.025804873	Mpst_KO_downregulated
NM_011247	*Rbbp6*	-0.102954617	0.031109605	Mpst_KO_downregulated
NM_026343	*Stx17*	-0.102461439	0.024525451	Mpst_KO_downregulated
NM_026499	*Srsf6*	-0.100313122	0.010608554	Mpst_KO_downregulated
NM_172807	*Ppwd1*	-0.096900588	0.034667344	Mpst_KO_downregulated
NM_175480	*Zfp612*	-0.091135679	0.046671474	Mpst_KO_downregulated
NM_016799	*Srrm1*	-0.090231489	0.040978535	Mpst_KO_downregulated
NM_001081022	*D430042O09Rik*	-0.089466888	0.020971566	Mpst_KO_downregulated
NM_172671	*Lgr4*	-0.089287703	0.045202272	Mpst_KO_downregulated
NM_145968	*Tagap*	-0.088973771	0.042458267	Mpst_KO_downregulated
NM_026609	*Leprotl1*	-0.087426735	0.002710459	Mpst_KO_downregulated
NM_028248	*Tmem87b*	-0.08739308	0.04630087	Mpst_KO_downregulated
NM_027421	*Ints2*	-0.084987288	0.045986733	Mpst_KO_downregulated
NM_008971	*Twf1*	-0.084669426	0.039864058	Mpst_KO_downregulated
NM_029658	*Fam101b*	-0.084185372	0.007797617	Mpst_KO_downregulated
NM_009461	*Ubr1*	-0.08071835	0.029405357	Mpst_KO_downregulated
NM_027264	*Zfp715*	-0.078788706	0.015580897	Mpst_KO_downregulated
NM_198033	*Setx*	-0.078103636	0.034557314	Mpst_KO_downregulated
NM_027530	*Rufy3*	-0.077434346	0.040801935	Mpst_KO_downregulated
NM_009360	*Tfam*	-0.076993977	0.016902505	Mpst_KO_downregulated
NM_028731	*Esyt2*	-0.075687661	0.015010662	Mpst_KO_downregulated
NM_011358	*Srsf2*	-0.075665193	0.033668585	Mpst_KO_downregulated
NM_011890	*Sgcb*	-0.070010119	0.009875574	Mpst_KO_downregulated
NM_177139	*Lypd6*	-0.070001536	0.04325906	Mpst_KO_downregulated
NM_008917	*Ppt1*	-0.065967048	0.040356733	Mpst_KO_downregulated
NM_001164101	*Add3*	-0.065904627	0.026990325	Mpst_KO_downregulated
NM_010832	*Msl3*	-0.065805587	0.029839382	Mpst_KO_downregulated
NM_133679	*Cryzl1*	-0.064271777	0.044048967	Mpst_KO_downregulated
NM_027472	*5730455P16Rik*	-0.063920244	0.038993531	Mpst_KO_downregulated
NM_001045536	*Zzef1*	-0.058059865	0.044714452	Mpst_KO_downregulated
NM_011946	*Map3k2*	-0.055432289	0.044758201	Mpst_KO_downregulated
NM_172585	*Larp4b*	-0.052590255	0.049348847	Mpst_KO_downregulated
NM_178880	*Son*	-0.049776844	0.041758831	Mpst_KO_downregulated
NM_018872	*Tmem131*	-0.049565841	0.021331754	Mpst_KO_downregulated
NM_011714	*Baz1b*	-0.039389459	0.009723668	Mpst_KO_downregulated
NM_027315	*Ube2q1*	0.034544346	0.046595465	Mpst_KO_upregulated
NM_009005	*Rab7*	0.048040143	0.040512798	Mpst_KO_upregulated

NM_028860	*Mtmr3*	0.053583175	0.033142308	Mpst_KO_upregulated
NM_001013380	*Dync1li2*	0.054525638	0.020897409	Mpst_KO_upregulated
NM_001080932	*Foxk2*	0.061479516	0.03601024	Mpst_KO_upregulated
NM_019634	*Tspan7*	0.062827297	0.011286511	Mpst_KO_upregulated
NM_011416	*Smarca2*	0.06488185	0.040498054	Mpst_KO_upregulated
NM_177267	*Dcaf5*	0.065756697	0.041933293	Mpst_KO_upregulated
NM_033526	*Ubqln4*	0.067836942	0.035540869	Mpst_KO_upregulated
NM_144518	*2900011O08Rik*	0.068265975	0.04900675	Mpst_KO_upregulated
NM_175930	*Rapgef5*	0.069342218	0.027136216	Mpst_KO_upregulated
NM_010357	*Gsta4*	0.070187651	0.032950598	Mpst_KO_upregulated
NM_013681	*Syn2*	0.070906899	0.010036049	Mpst_KO_upregulated
NM_009142	*Cx3cl1*	0.07191687	0.047634909	Mpst_KO_upregulated
NM_001102455	*Aplp2*	0.073742847	0.046296384	Mpst_KO_upregulated
NM_001080930	*Atxn1l*	0.073908652	0.018448602	Mpst_KO_upregulated
NM_001033463	*Tatdn2*	0.075544184	0.027980132	Mpst_KO_upregulated
NM_008395	*Itch*	0.07618967	0.040081874	Mpst_KO_upregulated
NM_174998	*Hpcal4*	0.07737377	0.036365676	Mpst_KO_upregulated
NM_026519	*Emc4*	0.078174741	0.025075754	Mpst_KO_upregulated
NM_175164	*Arhgap26*	0.079345986	0.029328233	Mpst_KO_upregulated
NM_010885	*Ndufa2*	0.0815374	0.039350795	Mpst_KO_upregulated
NM_001040106	*Aak1*	0.084061294	0.032615978	Mpst_KO_upregulated
NM_170755	*Fam134a*	0.084927145	0.04084514	Mpst_KO_upregulated
NM_009136	*Scrg1*	0.087322712	0.020822555	Mpst_KO_upregulated
NM_001163425	*Myeov2*	0.088729878	0.045813459	Mpst_KO_upregulated
NM_021539	*Wsb2*	0.090241597	0.043011187	Mpst_KO_upregulated
NM_134091	*Sgsm3*	0.095407612	0.024775525	Mpst_KO_upregulated
NM_033583	*Pcdhgc5*	0.097865379	0.029542529	Mpst_KO_upregulated
NM_133665	*Mef2d*	0.100652022	0.030221325	Mpst_KO_upregulated
NM_153134	*Irgq*	0.102342607	0.0160296	Mpst_KO_upregulated
NM_021393	*Nelfb*	0.103002813	0.045950326	Mpst_KO_upregulated
NM_001146174	*Rangap1*	0.103472416	0.011391914	Mpst_KO_upregulated
NM_007805	*Cyb561*	0.103758381	0.03735946	Mpst_KO_upregulated
NM_008948	*Psmc3*	0.103762084	0.04375182	Mpst_KO_upregulated
NM_026615	*Urm1*	0.104422901	0.010457626	Mpst_KO_upregulated
NM_183315	*Ctxn1*	0.105960558	0.022592669	Mpst_KO_upregulated
NM_175402	*Rbm15b*	0.105972409	0.042899217	Mpst_KO_upregulated
NM_025544	*Mrps15*	0.109902573	0.04526553	Mpst_KO_upregulated
NM_029219	*Rnf19b*	0.110342199	0.037772845	Mpst_KO_upregulated
NM_080559	*Sh3bgrl3*	0.110451761	0.006067902	Mpst_KO_upregulated
NM_012014	*Gprin1*	0.111623305	0.04784855	Mpst_KO_upregulated
NM_019661	*Ykt6*	0.111998349	0.016647058	Mpst_KO_upregulated
NM_053177	*Mcoln1*	0.113404596	0.033885075	Mpst_KO_upregulated
NM_025808	*Lztr1*	0.115809293	0.036752544	Mpst_KO_upregulated
NM_019392	*Tyro3*	0.116598311	0.048841079	Mpst_KO_upregulated
NM_011431	*Eftud2*	0.117989704	0.02471878	Mpst_KO_upregulated
NM_025500	*Mrpl37*	0.119804632	0.030649017	Mpst_KO_upregulated
NM_001081114	*Clip3*	0.120397121	0.022070494	Mpst_KO_upregulated
NM_001271487	*Rtn3*	0.12062268	0.033839539	Mpst_KO_upregulated
NM_144534	*Tmem38a*	0.121202363	0.030418364	Mpst_KO_upregulated
NM_001042451	*Snca*	0.122557246	0.04630279	Mpst_KO_upregulated
NM_148946	*Slc8a2*	0.124643352	0.016784527	Mpst_KO_upregulated
NM_023900	*Plekhj1*	0.127625926	0.032003864	Mpst_KO_upregulated
NM_028762	*Rbm19*	0.128295202	0.021476098	Mpst_KO_upregulated
NR_027372	*Arhgap1*	0.129317022	0.038481053	Mpst_KO_upregulated
NM_138601	*D10Jhu81e*	0.131567262	0.029674995	Mpst_KO_upregulated
NM_001109975	*Synpo*	0.131751963	0.040834735	Mpst_KO_upregulated
NM_001024717	*Gal3st3*	0.1321672	0.01433959	Mpst_KO_upregulated
NM_025421	*Acyp1*	0.134651863	0.04718569	Mpst_KO_upregulated
NM_001252639	*Tbc1d7*	0.135004962	0.029633528	Mpst_KO_upregulated
NM_009164	*Sh3bp1*	0.135474081	0.027513833	Mpst_KO_upregulated
NR_045176	*2810468N07Rik*	0.135592017	0.04870288	Mpst_KO_upregulated
NM_007698	*Chrm1*	0.137778834	0.020835641	Mpst_KO_upregulated

NM_011846	*Mmp17*	0.138811991	0.013729962	Mpst_KO_upregulated
NM_013622	*Oprd1*	0.141536599	0.026673036	Mpst_KO_upregulated
NM_172668	*Lrp4*	0.141914834	0.035894922	Mpst_KO_upregulated
NM_175154	*Galk2*	0.142864434	0.036722795	Mpst_KO_upregulated
NM_001081227	*6330403A02Rik*	0.143226705	0.012408208	Mpst_KO_upregulated
NM_024461	*1810037I17Rik*	0.144255104	0.010460654	Mpst_KO_upregulated
NM_001252332	*Sept8*	0.144876407	0.043292757	Mpst_KO_upregulated
NM_001001566	*Chpf*	0.145624147	0.020577689	Mpst_KO_upregulated
NM_011228	*Rab33a*	0.146482598	0.036971615	Mpst_KO_upregulated
NM_029609	*Lhpp*	0.148592872	0.037442937	Mpst_KO_upregulated
NM_001134383	*Iqsec1*	0.152275106	0.03838609	Mpst_KO_upregulated
NM_001037758	*Btrc*	0.152449665	0.0259189	Mpst_KO_upregulated
NM_009000	*Rab24*	0.153078383	0.013834317	Mpst_KO_upregulated
NM_207225	*Hdac4*	0.153086063	0.032423333	Mpst_KO_upregulated
NM_019800	*Acp6*	0.153541019	0.038161764	Mpst_KO_upregulated
NM_170777	*Elof1*	0.153657246	0.033852158	Mpst_KO_upregulated
NM_153136	*Nudt18*	0.153905744	0.042543742	Mpst_KO_upregulated
NM_001085374	*Mcc*	0.154875898	0.04221112	Mpst_KO_upregulated
NM_012026	*Arhgef28*	0.155338539	0.017920488	Mpst_KO_upregulated
NM_026768	*Mrps18a*	0.156266844	0.037482125	Mpst_KO_upregulated
NM_026512	*Bphl*	0.15726149	0.015146313	Mpst_KO_upregulated
NM_001081456	*Plcd4*	0.157686005	0.034071842	Mpst_KO_upregulated
NM_008027	*Flot1*	0.15845309	0.003527817	Mpst_KO_upregulated
NM_029614	*Prss23*	0.162023291	0.048234751	Mpst_KO_upregulated
NM_199197	*Rbfa*	0.162524105	0.013042923	Mpst_KO_upregulated
NM_172630	*Mppe1*	0.162771178	0.030259871	Mpst_KO_upregulated
NM_001159640	*Tasp1*	0.162793139	0.046078797	Mpst_KO_upregulated
NM_144852	*Slc7a4*	0.163483211	0.008836858	Mpst_KO_upregulated
NM_001282127	*Brpf1*	0.16692971	0.03234568	Mpst_KO_upregulated
NM_026337	*Sltm*	0.167820749	0.039302982	Mpst_KO_upregulated
NM_027106	*Avpi1*	0.168634661	0.047860056	Mpst_KO_upregulated
NM_028364	*Puf60*	0.169910458	0.041924032	Mpst_KO_upregulated
NM_001173483	*Rlbp1*	0.172081531	0.031649939	Mpst_KO_upregulated
NM_001034013	*Asic2*	0.174957214	0.047794891	Mpst_KO_upregulated
NM_007656	*Cd82*	0.177118952	0.047696503	Mpst_KO_upregulated
NM_172758	*Slc38a7*	0.178100079	0.029356723	Mpst_KO_upregulated
NM_015773	*Spag6*	0.179212886	0.012250364	Mpst_KO_upregulated
NM_025870	*Swsap1*	0.179370654	0.015846202	Mpst_KO_upregulated
NM_010698	*Ldb2*	0.179542924	0.048440035	Mpst_KO_upregulated
NM_010108	*Efna3*	0.180129572	0.027220359	Mpst_KO_upregulated
NM_001167799	*Stx5a*	0.182078763	0.024146233	Mpst_KO_upregulated
NM_138654	*Sugct*	0.182824391	0.016691969	Mpst_KO_upregulated
NM_001170567	*Depdc5*	0.184246059	0.029763219	Mpst_KO_upregulated
NM_028454	*Tm7sf2*	0.185913004	0.034425348	Mpst_KO_upregulated
NM_001164671	*Dnaja1*	0.186275626	0.036682956	Mpst_KO_upregulated
NM_145450	*BC022687*	0.189327823	0.02785268	Mpst_KO_upregulated
NM_029761	*Dok5*	0.190011229	0.048794985	Mpst_KO_upregulated
NM_001077495	*Pik3r1*	0.191838029	0.002827016	Mpst_KO_upregulated
NM_008812	*Padi2*	0.200916256	0.027280851	Mpst_KO_upregulated
NM_026345	*Mansc1*	0.204467109	0.036837115	Mpst_KO_upregulated
NM_181318	*Rasgef1b*	0.207150071	0.047017449	Mpst_KO_upregulated
NM_144849	*Ankrd54*	0.207531287	0.033761012	Mpst_KO_upregulated
NM_001033204	*Rpusd3*	0.217811083	0.005406374	Mpst_KO_upregulated
NM_018855	*Gas8*	0.231716197	0.007553074	Mpst_KO_upregulated
NM_001029933	*Zfp114*	0.233186448	0.024800215	Mpst_KO_upregulated
NR_033326	*Gm5089*	0.237337688	0.044693813	Mpst_KO_upregulated
NM_016762	*Matn2*	0.238364887	0.009489848	Mpst_KO_upregulated
NM_001025296	*Dffa*	0.238878346	0.04343749	Mpst_KO_upregulated
NM_001083320	*Magi1*	0.239010574	0.023083876	Mpst_KO_upregulated
NM_001171035	*Tmbim6*	0.240938977	0.046231938	Mpst_KO_upregulated
NM_010187	*Fcgr2b*	0.242641859	0.026696696	Mpst_KO_upregulated
NM_029602	*Znrd1as*	0.246832168	0.006983127	Mpst_KO_upregulated

NM_001166430	*Hnrnpf*	0.247127109	0.029186132	Mpst_KO_upregulated
NM_001289685	*Iqsec3*	0.249295789	0.038745499	Mpst_KO_upregulated
NM_029628	*Ppp1r3g*	0.254364679	0.013846455	Mpst_KO_upregulated
NM_023240	*Eef1d*	0.258665072	0.047607417	Mpst_KO_upregulated
NM_053122	*Immp2l*	0.262702753	0.029843856	Mpst_KO_upregulated
NM_001291052	*Gpcpd1*	0.26394704	0.008778419	Mpst_KO_upregulated
NM_025649	*Mad2l1bp*	0.26599702	0.013133248	Mpst_KO_upregulated
NM_172546	*Cnksr3*	0.269339546	0.009739513	Mpst_KO_upregulated
NM_001145950	*5730409E04Rik*	0.271080035	0.00732188	Mpst_KO_upregulated
NM_001111288	*Sco2*	0.273767057	0.045597933	Mpst_KO_upregulated
NM_001143776	*Fam13c*	0.274249702	0.048370245	Mpst_KO_upregulated
NM_001170982	*Hnrnpc*	0.275825043	0.037794224	Mpst_KO_upregulated
NM_001170866	*Ncald*	0.278097568	0.041860403	Mpst_KO_upregulated
NM_145954	*Aldh16a1*	0.285610247	0.024789649	Mpst_KO_upregulated
NM_198299	*Enkd1*	0.285918399	0.011513729	Mpst_KO_upregulated
NM_172872	*Kank4*	0.286980778	0.027820315	Mpst_KO_upregulated
NM_172815	*Rspo2*	0.293157622	0.046176489	Mpst_KO_upregulated
NM_001303610	*Hus1*	0.301690326	0.038068849	Mpst_KO_upregulated
NM_001146212	*Mrps10*	0.30305218	0.03063755	Mpst_KO_upregulated
NM_001110148	*Mgat1*	0.303552682	0.048771992	Mpst_KO_upregulated
NM_001166635	*Mid1ip1*	0.30586617	0.013312395	Mpst_KO_upregulated
NM_175530	*Fbxo46*	0.30661666	0.011253494	Mpst_KO_upregulated
NM_001170561	*Sbf1*	0.307543646	0.019001898	Mpst_KO_upregulated
NM_001302336	*Mrpl55*	0.312107507	0.010965715	Mpst_KO_upregulated
NM_024251	*Aplf*	0.312452751	0.033228803	Mpst_KO_upregulated
NR_027844	*Pex11b*	0.316879332	0.041143998	Mpst_KO_upregulated
NM_001199247	*Kat5*	0.318624052	0.049822354	Mpst_KO_upregulated
NM_025791	*Tmem223*	0.326899672	0.003603333	Mpst_KO_upregulated
NM_023524	*Tfpt*	0.333459536	0.019129973	Mpst_KO_upregulated
NM_133978	*Cmtm7*	0.338548976	0.024021066	Mpst_KO_upregulated
NM_027434	*Rprd1b*	0.343966609	0.041366889	Mpst_KO_upregulated
NM_026995	*Carkd*	0.351740471	0.015562617	Mpst_KO_upregulated
NM_001112731	*C030039L03Rik*	0.353535054	0.048524653	Mpst_KO_upregulated
NM_010438	*Hk1*	0.358242602	0.046501743	Mpst_KO_upregulated
NM_001033209	*Xylb*	0.359292032	0.008112324	Mpst_KO_upregulated
NM_001256195	*Eif4g3*	0.362257782	0.011014513	Mpst_KO_upregulated
NM_001282993	*Cobl*	0.364413522	0.001266959	Mpst_KO_upregulated
NM_026270	*Akt1s1*	0.365787919	0.048716579	Mpst_KO_upregulated
NM_001286498	*Tex264*	0.369348062	0.027749047	Mpst_KO_upregulated
NM_198214	*Snph*	0.369541061	0.042273394	Mpst_KO_upregulated
NM_028770	*Krt80*	0.376762543	0.043103971	Mpst_KO_upregulated
NM_027351	*Ppil3*	0.379790933	0.047264688	Mpst_KO_upregulated
NM_001161796	*Gucy1b3*	0.399335544	0.017543995	Mpst_KO_upregulated
NM_001252460	*Cyfip2*	0.405283211	0.008089743	Mpst_KO_upregulated
NM_001166388	*Ptp4a3*	0.418192455	0.005877309	Mpst_KO_upregulated
NM_001290457	*Relb*	0.420436979	0.031826632	Mpst_KO_upregulated
NM_010724	*Psmb8*	0.42375119	0.023099094	Mpst_KO_upregulated
NR_040577	*1700123M08Rik*	0.431430994	0.006165611	Mpst_KO_upregulated
NM_001290477	*Klhl4*	0.43532163	0.036488342	Mpst_KO_upregulated
NM_019583	*Il17rb*	0.454346847	0.018909936	Mpst_KO_upregulated
NR_026924	*1700020N18Rik*	0.454605137	0.045017496	Mpst_KO_upregulated
NM_001164413	*Cnot4*	0.461483483	0.022757924	Mpst_KO_upregulated
NM_001277992	*Stk16*	0.465922326	0.046654198	Mpst_KO_upregulated
NM_008546	*Mfap2*	0.476253827	0.046332481	Mpst_KO_upregulated
NM_020616	*Akip1*	0.485775345	0.041885854	Mpst_KO_upregulated
NM_001199685	*Taok3*	0.491072495	0.027143718	Mpst_KO_upregulated
NR_033185	*Rcbtb2*	0.507031569	0.03429913	Mpst_KO_upregulated
NM_001160219	*Pum2*	0.515210489	0.019875633	Mpst_KO_upregulated
NM_001290375	*Camk1d*	0.516886997	0.048522584	Mpst_KO_upregulated
NM_001145780	*Use1*	0.51957533	0.038583623	Mpst_KO_upregulated
NM_010839	*Mtcp1*	0.526800151	0.041405362	Mpst_KO_upregulated
NM_146024	*Ankrd40*	0.536653699	0.040187586	Mpst_KO_upregulated

NM_001164359	*Erlin1*	0.542241455	0.0361156	Mpst_KO_upregulated
NM_001080386	*Clta*	0.550343063	0.019790704	Mpst_KO_upregulated
NM_001170867	*Ncald*	0.556788137	0.037880661	Mpst_KO_upregulated
NR_102282	*Cops5*	0.557548983	0.015910014	Mpst_KO_upregulated
NM_029341	*Capsl*	0.580985156	0.008088	Mpst_KO_upregulated
NM_001040131	*Eif4g2*	0.581162505	0.0452219	Mpst_KO_upregulated
NM_001270495	*Tmem254b*	0.582643037	0.016837485	Mpst_KO_upregulated
NM_001302383	*March8*	0.600223473	0.007055224	Mpst_KO_upregulated
NM_007681	*Cenpa*	0.607267035	0.023535562	Mpst_KO_upregulated
NM_033474	*Arvcf*	0.615265405	0.030696155	Mpst_KO_upregulated
NM_025657	*Lrrc57*	0.620258248	0.017851632	Mpst_KO_upregulated
NM_001270495	*Tmem254b*	0.622310888	0.01480553	Mpst_KO_upregulated
NM_146042	*Rnf144b*	0.631033676	0.036367551	Mpst_KO_upregulated
NM_011359	*Sftpc*	0.647191256	0.025927819	Mpst_KO_upregulated
NM_001191025	*Dync1i1*	0.67798386	0.012671719	Mpst_KO_upregulated
NM_001270495	*Tmem254b*	0.679384499	0.013291164	Mpst_KO_upregulated
NM_001146293	*Celf4*	0.681815561	0.024749004	Mpst_KO_upregulated
NM_027216	*Slc39a11*	0.687115478	0.039637545	Mpst_KO_upregulated
NM_031186	*Ndst3*	0.689266488	0.038678642	Mpst_KO_upregulated
NM_001008791	*Whrn*	0.710802367	0.027805526	Mpst_KO_upregulated
NM_001161629	*Efcab6*	0.71182	0.03457082	Mpst_KO_upregulated
NM_001284328	*Syncrip*	0.723963207	0.023284527	Mpst_KO_upregulated
NM_001293647	*Pip5k1c*	0.740389216	0.0018895	Mpst_KO_upregulated
NM_001291747	*Xlr*	0.741097995	0.040768277	Mpst_KO_upregulated
NM_172434	*Celf3*	0.750693912	0.01885894	Mpst_KO_upregulated
NM_053155	*Clmn*	0.81444415	0.041873744	Mpst_KO_upregulated
NM_144832	*BC017643*	0.819296718	0.01753166	Mpst_KO_upregulated
NM_001301858	*Uba3*	0.838030226	0.044421317	Mpst_KO_upregulated
NM_138312	*Fam172a*	0.887491229	0.027489413	Mpst_KO_upregulated
NM_001277280	*Ank1*	0.890758191	0.012282248	Mpst_KO_upregulated
NM_001167991	*Hook2*	1.030048524	0.043305179	Mpst_KO_upregulated
NM_001290795	*Wdr45*	1.124346844	0.040650237	Mpst_KO_upregulated
NM_001159394	*Nfkbiz*	1.158792014	0.02145787	Mpst_KO_upregulated
NM_001199360	*Tmem164*	1.214007141	0.04557474	Mpst_KO_upregulated
NM_008054	*Fyn*	1.220781995	0.005491825	Mpst_KO_upregulated
NM_172963	*Mtcl1*	1.229522345	0.004251622	Mpst_KO_upregulated
NM_001289785	*Cryab*	1.372382099	0.000975047	Mpst_KO_upregulated
NM_001289436	*Ccdc50*	1.600926578	0.019575966	Mpst_KO_upregulated
NM_001039387	*Nsmf*	2.161510292	0.047581279	Mpst_KO_upregulated
NM_199238	*Sema6d*	4.900578903	0.024718602	Mpst_KO_upregulated
NM_178371	*Slc9a8*	4.903544557	0.039949626	Mpst_KO_upregulated
NM_001177784	*Dis3l*	5.057352872	0.04358277	Mpst_KO_upregulated
NM_001281926	*Zmynd8*	5.086358627	0.023541935	Mpst_KO_upregulated
NM_183033	*Zfp516*	5.24749189	0.037842616	Mpst_KO_upregulated
NM_172445	*Wdr37*	5.304436844	0.047958372	Mpst_KO_upregulated
NM_201385	*Plec*	5.511575508	0.023120016	Mpst_KO_upregulated
NM_001177938	*Eda*	5.926834551	0.010535103	Mpst_KO_upregulated
NM_001281829	*Phyhd1*	6.066692948	0.015855977	Mpst_KO_upregulated
NM_001166648	*Zfp280c*	6.311140896	0.007110142	Mpst_KO_upregulated
NM_008330	*Ifi47*	7.69799315	0.006786125	Mpst_KO_upregulated
NM_016885	*Emcn*	7.994088303	0.004623172	Mpst_KO_upregulated

Appendix Table S12. Gene ontology enrichment analysis of differentially expressed genes in the frontal cortex of *Mpst* KO and *Mpst* Tg mouse strains.

Group	PANTHER GO-Slim Biological Process	Fold Enrichment	Raw *P*-value	FDR
***Mpst* KO dysregulated genes**	regulation of alternative mRNA splicing, via spliceosome (GO:0000381)	8.99	2.87E-05	1.28E-02
	alternative mRNA splicing, via spliceosome (GO:0000380)	8.99	2.87E-05	1.02E-02
	mRNA processing (GO:0006397)	3.47	9.64E-05	2.15E-02
	RNA processing (GO:0006396)	3.01	2.44E-04	4.83E-02
	**PANTHER GO-Slim Molecular Function**	**Fold Enrichment**	**Raw *P*-value**	**FDR**
	mRNA binding (GO:0003729)	3.58	4.21E-04	3.59E-02
	RNA binding (GO:0003723)	2.34	1.96E-04	2.01E-02
	**PANTHER GO-Slim Cellular Component**	**Fold Enrichment**	**Raw *P*-value**	**FDR**
	nucleus (GO:0005634)	1.72	4.51E-04	4.04E-02
	intracellular membrane-bounded organelle (GO:0043231)	1.51	2.61E-04	5.86E-02
	membrane-bounded organelle (GO:0043227)	1.5	2.69E-04	4.01E-02

Group	PANTHER GO-Slim Biological Process	Fold Enrichment	Raw *P*-value	FDR
***Mpst* Tg dysregulated genes**	nucleotide phosphorylation (GO:0046939)	5.46	7.09E-04	2.94E-02
	synaptic transmission, glutamatergic (GO:0035249)	5.1	4.38E-04	2.05E-02
	chemical synaptic transmission (GO:0007268)	2.24	2.37E-04	1.32E-02
	anterograde trans-synaptic signaling (GO:0098916)	2.24	2.37E-04	1.28E-02
	trans-synaptic signaling (GO:0099537)	2.23	2.48E-04	1.30E-02
	synaptic signaling (GO:0099536)	2.23	2.48E-04	1.26E-02
	intracellular transport (GO:0046907)	1.73	1.12E-04	9.55E-03
	establishment of localization in cell (GO:0051649)	1.71	1.01E-04	8.98E-03
	nucleobase-containing compound metabolic process (GO:0006139)	1.7	4.89E-04	2.24E-02
	intracellular protein transport (GO:0006886)	1.68	1.06E-03	4.20E-02
	cellular aromatic compound metabolic process (GO:0006725)	1.65	8.62E-04	3.49E-02
	transport (GO:0006810)	1.51	1.57E-04	9.65E-03
	cellular metabolic process (GO:0044237)	1.47	1.53E-04	9.73E-03
	establishment of localization (GO:0051234)	1.46	6.30E-05	7.02E-03
	localization (GO:0051179)	1.42	8.48E-05	8.90E-03
	regulation of cellular process (GO:0050794)	1.32	6.58E-04	2.79E-02
	biological regulation (GO:0065007)	1.22	1.11E-03	4.31E-02
	metabolic process (GO:0008152)	1.21	5.75E-04	2.57E-02
	**PANTHER GO-Slim Molecular Function**	**Fold Enrichment**	**Raw *P*-value**	**FDR**
	glutamate receptor activity (GO:0008066)	4.11	1.50E-03	4.26E-02
	glutamate binding (GO:0016595)	4.11	1.50E-03	4.03E-02
	hydro-lyase activity (GO:0016836)	4.03	8.69E-04	3.70E-02
	carbon-oxygen lyase activity (GO:0016835)	4.03	8.69E-04	3.42E-02
	carboxylic acid binding (GO:0031406)	3.09	1.72E-03	4.19E-02
	neurotransmitter binding (GO:0042165)	2.82	1.44E-03	4.89E-02
	ligand-gated ion channel activity (GO:0015276)	2.76	1.69E-03	4.33E-02
	calcium ion binding (GO:0005509)	2.43	2.34E-04	1.33E-02
	ion binding (GO:0043167)	2.13	1.69E-06	8.64E-04
	heterocyclic compound binding (GO:1901363)	2.11	1.03E-03	3.77E-02
	transferase activity, transferring phosphorus-containing groups (GO:0016772)	2.09	8.58E-04	3.99E-02

	cation binding (GO:0043169)	2.02	1.34E-04	8.59E-03
	RNA binding (GO:0003723)	1.71	1.44E-03	4.61E-02
	transferase activity (GO:0016740)	1.55	1.45E-03	4.36E-02
	catalytic activity (GO:0003824)	1.28	1.88E-05	1.60E-03
	binding (GO:0005488)	1.2	2.49E-04	1.27E-02
	**PANTHER GO-Slim Cellular Component**	**Fold Enrichment**	**Raw P-value**	**FDR**
	cell body (GO:0044297)	4.51	4.27E-04	1.74E-02
	neuronal cell body (GO:0043025)	4.11	1.50E-03	3.95E-02
	transferase complex, transferring phosphorus-containing groups (GO:0061695)	2.94	1.02E-03	3.25E-02
	neuron projection (GO:0043005)	2.5	4.49E-05	2.87E-03
	synapse (GO:0045202)	2.46	4.20E-04	1.88E-02
	synapse part (GO:0044456)	2.45	6.07E-04	2.27E-02
	cell projection (GO:0042995)	2.05	2.89E-04	1.44E-02
	plasma membrane bounded cell projection (GO:0120025)	1.98	9.86E-04	3.40E-02
	plasma membrane region (GO:0098590)	1.94	8.94E-05	5.01E-03
	intracellular part (GO:0044424)	1.29	1.80E-05	1.61E-03
	cell (GO:0005623)	1.23	1.76E-05	1.97E-03
	cell part (GO:0044464)	1.22	2.74E-05	2.05E-03

Appendix Table S13. Differentially expressed genes from the frontal cortex of *Mpst* Tg mouse.

Refseq_ID	Gene	Log_2 fold change	*P* value	Regulation
NM_001252439	*D16Ertd472e*	-7.031826953	0.018302488	Mpst_Tg_Downregulation
NM_018812	*Pias3*	-6.365427871	0.017477454	Mpst_Tg_Downregulation
NM_001162479	*Stra6*	-6.163856332	0.017809574	Mpst_Tg_Downregulation
NM_030265	*Kcnip4*	-6.048734301	0.016593018	Mpst_Tg_Downregulation
NM_001289596	*Slc25a40*	-6.032358301	0.038210421	Mpst_Tg_Downregulation
NM_015804	*Atp11a*	-5.610338577	0.032011218	Mpst_Tg_Downregulation
NM_001281926	*Zmynd8*	-5.515705025	0.042294458	Mpst_Tg_Downregulation
NM_001035243	*Trpm3*	-5.400935045	0.031823909	Mpst_Tg_Downregulation
NM_172587	*Cdc14b*	-5.383538868	0.033081934	Mpst_Tg_Downregulation
NM_001204281	*Hdac7*	-5.003319105	0.04304314	Mpst_Tg_Downregulation
NM_178701	*Lrrc8d*	-3.246011957	0.029913852	Mpst_Tg_Downregulation
NM_001286546	*Ccpg1*	-1.912573008	0.003888251	Mpst_Tg_Downregulation
NM_001038700	*Fnbp1*	-1.580472758	0.008564262	Mpst_Tg_Downregulation
NM_028811	*Elp3*	-1.267563726	0.006154797	Mpst_Tg_Downregulation
NM_011523	*Synj2*	-1.180682326	0.008612549	Mpst_Tg_Downregulation
NM_001098237	*Zbtb3*	-1.156420222	0.022477822	Mpst_Tg_Downregulation
NR_045869	*Gm9866*	-1.087972438	0.022241967	Mpst_Tg_Downregulation
NR_028280	*Prr18*	-1.045744612	0.002471894	Mpst_Tg_Downregulation
NM_009122	*Satb1*	-1.043387998	0.018988682	Mpst_Tg_Downregulation
NM_028027	*Arhgef25*	-0.955094645	0.011897939	Mpst_Tg_Downregulation
NM_001302506	*Tmub2*	-0.952876451	0.020816054	Mpst_Tg_Downregulation
NM_144731	*Galnt7*	-0.916041368	0.019674067	Mpst_Tg_Downregulation
NR_040260	*2210408F21Rik*	-0.906243493	0.007698294	Mpst_Tg_Downregulation
NM_001177655	*Nsmf*	-0.903036424	0.016355997	Mpst_Tg_Downregulation
NM_016865	*Htatip2*	-0.900103625	0.012727248	Mpst_Tg_Downregulation
NM_001164099	*Add3*	-0.875292542	0.037441826	Mpst_Tg_Downregulation
NM_001282044	*Schip1*	-0.867869272	0.01627605	Mpst_Tg_Downregulation
NM_001024857	*Ttbk2*	-0.838802869	0.009304209	Mpst_Tg_Downregulation
NM_001291190	*Ssh2*	-0.835503572	0.014512986	Mpst_Tg_Downregulation
NM_001277970	*Itga6*	-0.816646245	0.014296822	Mpst_Tg_Downregulation
NM_001177605	*Ak4*	-0.782833233	0.0248379	Mpst_Tg_Downregulation
NM_001139511	*Raly*	-0.776538057	0.005883337	Mpst_Tg_Downregulation
NM_020276	*Nsmf*	-0.762911615	0.03183584	Mpst_Tg_Downregulation
NM_001029836	*Npnt*	-0.756175603	0.009134095	Mpst_Tg_Downregulation
NM_001141933	*Nkain4*	-0.712509379	0.038609624	Mpst_Tg_Downregulation
NM_001281929	*Gria3*	-0.698373851	0.027050789	Mpst_Tg_Downregulation
NM_001033356	*Ntn5*	-0.684122292	0.02363356	Mpst_Tg_Downregulation
NM_027100	*Rwdd2a*	-0.679561024	0.019880286	Mpst_Tg_Downregulation
NM_001010941	*Gpr12*	-0.669760524	0.020356059	Mpst_Tg_Downregulation
NM_145375	*Tm6sf1*	-0.661581489	0.030195165	Mpst_Tg_Downregulation
NR_028056	*Rit1*	-0.642863934	0.026559598	Mpst_Tg_Downregulation
NM_009393	*Tnnc1*	-0.627869213	5.01735E-06	Mpst_Tg_Downregulation
NM_001098230	*Pdp1*	-0.59149559	0.008194007	Mpst_Tg_Downregulation
NM_153597	*Trpt1*	-0.589414762	0.033278693	Mpst_Tg_Downregulation
NM_001161822	*Rgs17*	-0.580156143	0.009822152	Mpst_Tg_Downregulation
NM_001302095	*Gfra2*	-0.576795117	0.01609594	Mpst_Tg_Downregulation
NM_020256	*Zbtb33*	-0.576608782	0.030439631	Mpst_Tg_Downregulation
NM_145215	*Abhd11*	-0.565158055	0.030563545	Mpst_Tg_Downregulation
NM_053078	*Nrep*	-0.563619394	0.005405412	Mpst_Tg_Downregulation
NM_001271430	*Cd82*	-0.56126336	0.040525815	Mpst_Tg_Downregulation
NM_001302085	*Uros*	-0.535099605	0.042525149	Mpst_Tg_Downregulation
NM_001302336	*Mrpl55*	-0.53247534	0.038910179	Mpst_Tg_Downregulation
NM_001163569	*Kif9*	-0.531012672	0.046265418	Mpst_Tg_Downregulation
NM_001286600	*Clasp2*	-0.517712537	0.006480956	Mpst_Tg_Downregulation
NM_001276482	*Dag1*	-0.509248109	0.029636919	Mpst_Tg_Downregulation
NR_040257	*2210408F21Rik*	-0.508328498	0.00629517	Mpst_Tg_Downregulation

NM_001286362	*Tcf25*	-0.506744444	0.014867442	Mpst_Tg_Downregulation
NR_045868	*Gm9866*	-0.505418984	0.000438511	Mpst_Tg_Downregulation
NM_001163764	*Tcf19*	-0.502024373	0.008960471	Mpst_Tg_Downregulation
NM_207161	*Dnph1*	-0.497740428	0.02923159	Mpst_Tg_Downregulation
NM_001134459	*Prickle2*	-0.488455164	0.038965165	Mpst_Tg_Downregulation
NM_134066	*Akr1c18*	-0.486525397	0.048474939	Mpst_Tg_Downregulation
NM_026518	*Rnf146*	-0.480071681	0.035031525	Mpst_Tg_Downregulation
NM_001271759	*Adra1a*	-0.479161564	0.035901563	Mpst_Tg_Downregulation
NM_011444	*Sox5*	-0.47210266	0.016856431	Mpst_Tg_Downregulation
NM_178929	*Kazald1*	-0.466850218	0.00650838	Mpst_Tg_Downregulation
NM_001130408	*Arf1*	-0.452985867	0.013441759	Mpst_Tg_Downregulation
NM_153060	*Spns2*	-0.448687986	0.045569909	Mpst_Tg_Downregulation
NM_033564	*Mpv17l*	-0.447539918	0.01601173	Mpst_Tg_Downregulation
NM_178779	*Rnf152*	-0.445536239	0.039456927	Mpst_Tg_Downregulation
NM_027476	*Zdhhc24*	-0.432106788	0.034895912	Mpst_Tg_Downregulation
NM_178643	*C230052I12Rik*	-0.431331278	0.032797363	Mpst_Tg_Downregulation
NM_001113554	*Nudcd1*	-0.4210442	0.038383161	Mpst_Tg_Downregulation
NM_138303	*Yipf2*	-0.419845022	0.014396469	Mpst_Tg_Downregulation
NM_001252055	*Ly6c1*	-0.41656549	0.025121889	Mpst_Tg_Downregulation
NM_001288612	*Atg9a*	-0.413906374	0.00762262	Mpst_Tg_Downregulation
NM_025426	*Med7*	-0.404101371	0.0344107	Mpst_Tg_Downregulation
NM_022009	*Flii*	-0.396201465	0.006412254	Mpst_Tg_Downregulation
NM_001161722	*Tfeb*	-0.390132322	0.021606065	Mpst_Tg_Downregulation
NM_001302078	*Fkbp1a*	-0.379853621	0.00352545	Mpst_Tg_Downregulation
NM_178256	*Reps2*	-0.37837863	0.006818853	Mpst_Tg_Downregulation
NM_001291076	*Snph*	-0.376765193	0.042441743	Mpst_Tg_Downregulation
NM_001113564	*Serbp1*	-0.374199128	0.002096617	Mpst_Tg_Downregulation
NM_007394	*Acvr1*	-0.37385336	0.014040017	Mpst_Tg_Downregulation
NM_001130185	*Jade1*	-0.367105394	0.047529572	Mpst_Tg_Downregulation
NM_001302779	*Iffo1*	-0.361620619	0.042586621	Mpst_Tg_Downregulation
NM_080433	*Fezf2*	-0.360523363	0.03690322	Mpst_Tg_Downregulation
NM_010131	*Emx1*	-0.360174285	0.018276947	Mpst_Tg_Downregulation
NM_028897	*Mei1*	-0.359418355	0.028460731	Mpst_Tg_Downregulation
NM_001025584	*Kcnj6*	-0.358415642	0.049766464	Mpst_Tg_Downregulation
NM_001002011	*Lmna*	-0.357564604	0.009565045	Mpst_Tg_Downregulation
NM_011344	*Sel1l*	-0.357475156	0.004848597	Mpst_Tg_Downregulation
NM_001271548	*Gm715*	-0.356829099	0.01162675	Mpst_Tg_Downregulation
NM_013838	*Trpc6*	-0.350923163	0.047238906	Mpst_Tg_Downregulation
NM_011878	*Tiam2*	-0.34920452	0.04035477	Mpst_Tg_Downregulation
NM_152947	*Ovol2*	-0.347751415	0.028120707	Mpst_Tg_Downregulation
NM_001110211	*Anxa6*	-0.347116018	0.02406202	Mpst_Tg_Downregulation
NM_001043354	*Rorb*	-0.346968636	0.037021455	Mpst_Tg_Downregulation
NM_031161	*Cck*	-0.34474743	0.000715399	Mpst_Tg_Downregulation
NM_026675	*Nudt22*	-0.344314573	0.026485846	Mpst_Tg_Downregulation
NM_013613	*Nr4a2*	-0.342040879	0.034676894	Mpst_Tg_Downregulation
NM_001190386	*Caly*	-0.341122201	0.016478521	Mpst_Tg_Downregulation
NM_198408	*Crhbp*	-0.339550655	0.001611807	Mpst_Tg_Downregulation
NM_001289710	*Sh3glb2*	-0.338815223	0.037539231	Mpst_Tg_Downregulation
NM_008939	*Prss12*	-0.337598271	0.017831976	Mpst_Tg_Downregulation
NM_001286785	*Magi1*	-0.334028672	0.030615981	Mpst_Tg_Downregulation
NR_027832	*1700020I14Rik*	-0.322449797	0.015741706	Mpst_Tg_Downregulation
NM_001177720	*Madd*	-0.32022901	0.001232782	Mpst_Tg_Downregulation
NM_030234	*Wdr76*	-0.320052831	0.012136729	Mpst_Tg_Downregulation
NM_001102437	*Acbd5*	-0.320005864	0.001345871	Mpst_Tg_Downregulation
NM_134094	*Ncald*	-0.319990895	0.012923177	Mpst_Tg_Downregulation
NM_001198911	*Arhgef2*	-0.317717912	0.023366915	Mpst_Tg_Downregulation
NM_001166251	*Mgll*	-0.315141002	0.003060661	Mpst_Tg_Downregulation
NM_008627	*Meis3*	-0.313712542	0.037975767	Mpst_Tg_Downregulation
NM_153820	*Arhgap15*	-0.311754089	0.018012501	Mpst_Tg_Downregulation
NM_001285999	*Plekhg5*	-0.311272461	0.028914784	Mpst_Tg_Downregulation
NR_045778	*C730002L08Rik*	-0.309869615	0.043518325	Mpst_Tg_Downregulation
NM_001160256	*Smg7*	-0.309799283	0.020020975	Mpst_Tg_Downregulation

NM_032397	*Kcnn1*	-0.309053838	0.007796019	Mpst_Tg_Downregulation
NM_138663	*Pcdha12*	-0.308810389	0.017436088	Mpst_Tg_Downregulation
NM_001252105	*Cadps2*	-0.308420897	0.027385945	Mpst_Tg_Downregulation
NM_172815	*Rspo2*	-0.308157776	0.04072273	Mpst_Tg_Downregulation
NM_173777	*Olfm2*	-0.3054474	0.004201641	Mpst_Tg_Downregulation
NM_010074	*Dpp4*	-0.30493765	0.042335129	Mpst_Tg_Downregulation
NM_175443	*Etnk2*	-0.302096257	0.021156009	Mpst_Tg_Downregulation
NM_001081981	*Nfix*	-0.300216923	0.030389161	Mpst_Tg_Downregulation
NM_024215	*Zfp593*	-0.299430289	0.010269961	Mpst_Tg_Downregulation
NM_198602	*Cux1*	-0.297531272	0.021891879	Mpst_Tg_Downregulation
NM_177910	*Gmppb*	-0.295442361	0.043543385	Mpst_Tg_Downregulation
NM_010858	*Myl4*	-0.295144127	0.0325284	Mpst_Tg_Downregulation
NM_001081960	*Clasp2*	-0.29382056	0.00377518	Mpst_Tg_Downregulation
NM_001013012	*Zfp787*	-0.293056845	0.001170435	Mpst_Tg_Downregulation
NM_001177616	*Arpp21*	-0.291590298	0.019734609	Mpst_Tg_Downregulation
NM_001290636	*Dock7*	-0.291527701	0.049154784	Mpst_Tg_Downregulation
NM_001033496	*Zfp213*	-0.290101071	0.041540532	Mpst_Tg_Downregulation
NM_145419	*Hkdc1*	-0.286204289	0.011481822	Mpst_Tg_Downregulation
NM_030226	*9130019O22Rik*	-0.286161139	0.020798274	Mpst_Tg_Downregulation
NM_029353	*Malsu1*	-0.285013411	0.026150596	Mpst_Tg_Downregulation
NM_001113181	*Gria4*	-0.279817294	0.014191688	Mpst_Tg_Downregulation
NM_001145820	*Gpd2*	-0.277462686	0.035142799	Mpst_Tg_Downregulation
NM_011698	*Lin7b*	-0.275292323	0.022144537	Mpst_Tg_Downregulation
NM_001252491	*Osbpl1a*	-0.274586397	0.035413162	Mpst_Tg_Downregulation
NR_027966	*4930429B21Rik*	-0.269522375	0.018415239	Mpst_Tg_Downregulation
NM_172812	*Htr2a*	-0.26809855	0.035374472	Mpst_Tg_Downregulation
NM_198001	*1110008P14Rik*	-0.265516686	0.000231156	Mpst_Tg_Downregulation
NM_001253692	*Elmod3*	-0.265389758	0.012693958	Mpst_Tg_Downregulation
NM_001282127	*Brpf1*	-0.262430439	0.043058284	Mpst_Tg_Downregulation
NM_010710	*Lhx2*	-0.256327959	0.023233239	Mpst_Tg_Downregulation
NR_024325	*9130024F11Rik*	-0.249481381	0.046509301	Mpst_Tg_Downregulation
NM_198651	*4430402I18Rik*	-0.24940281	0.023892985	Mpst_Tg_Downregulation
NM_053162	*Mrpl34*	-0.249117573	0.012040907	Mpst_Tg_Downregulation
NM_001146351	*Ephb6*	-0.248348589	0.010086169	Mpst_Tg_Downregulation
NM_153104	*Phospho1*	-0.247575683	0.039974256	Mpst_Tg_Downregulation
NM_030689	*Nptxr*	-0.245125977	0.037769038	Mpst_Tg_Downregulation
NM_170673	*Cpne9*	-0.243231588	0.014112189	Mpst_Tg_Downregulation
NM_001253716	*Msra*	-0.236987059	0.014379715	Mpst_Tg_Downregulation
NM_172822	*Pigz*	-0.236148626	0.025146961	Mpst_Tg_Downregulation
NM_175029	*Atg4c*	-0.235433589	0.047892247	Mpst_Tg_Downregulation
NM_027435	*Atad2*	-0.234817402	0.010270805	Mpst_Tg_Downregulation
NM_145942	*Hmgcs1*	-0.232915039	0.03211182	Mpst_Tg_Downregulation
NM_139146	*Satb2*	-0.231174887	0.013403942	Mpst_Tg_Downregulation
NM_026895	*Tatdn3*	-0.230866717	0.044971352	Mpst_Tg_Downregulation
NM_013645	*Pvalb*	-0.230196488	0.010160759	Mpst_Tg_Downregulation
NM_001304965	*Pou6f1*	-0.228448973	0.01391358	Mpst_Tg_Downregulation
NM_001128181	*Dlgap1*	-0.226933564	0.002878886	Mpst_Tg_Downregulation
NM_178399	*3110035E14Rik*	-0.226327706	0.009798875	Mpst_Tg_Downregulation
NM_007634	*Ccnf*	-0.225391242	0.021846193	Mpst_Tg_Downregulation
NM_001081381	*Elp6*	-0.225211101	0.010391621	Mpst_Tg_Downregulation
NM_207204	*Ninl*	-0.222644772	0.012184955	Mpst_Tg_Downregulation
NM_001160353	*Grm2*	-0.220576684	0.024291177	Mpst_Tg_Downregulation
NM_001037707	*Zfp27*	-0.220071832	0.048735735	Mpst_Tg_Downregulation
NM_145362	*Alg1*	-0.219448763	0.030704528	Mpst_Tg_Downregulation
NM_015802	*Dlc1*	-0.219120211	0.029619989	Mpst_Tg_Downregulation
NM_022982	*Rtn4r*	-0.21851466	0.021827507	Mpst_Tg_Downregulation
NM_001160211	*Cnih3*	-0.218146892	0.001459087	Mpst_Tg_Downregulation
NM_138583	*Tango2*	-0.215545177	0.001480443	Mpst_Tg_Downregulation
NM_001170537	*Mef2c*	-0.215346008	0.020745517	Mpst_Tg_Downregulation
NM_053190	*S1pr5*	-0.213773324	0.019536033	Mpst_Tg_Downregulation
NM_027123	*Fastkd3*	-0.2137532	0.009845157	Mpst_Tg_Downregulation
NM_025992	*Herc6*	-0.213610836	0.044355736	Mpst_Tg_Downregulation

NM_010212	*Fhl2*	-0.213403858	0.004140378	Mpst_Tg_Downregulation
NM_001099288	*Arhgap44*	-0.210068295	0.028969906	Mpst_Tg_Downregulation
NM_033269	*Chrm3*	-0.209089077	0.028986794	Mpst_Tg_Downregulation
NM_001100449	*Taf4b*	-0.205368623	0.017064344	Mpst_Tg_Downregulation
NM_001303015	*Ap3s1*	-0.205289057	0.031212082	Mpst_Tg_Downregulation
NM_001163516	*Pex5l*	-0.204664297	0.025926663	Mpst_Tg_Downregulation
NM_001160403	*Il1rapl1*	-0.204106904	0.045888871	Mpst_Tg_Downregulation
NM_001293631	*Slc37a4*	-0.202738764	0.006424152	Mpst_Tg_Downregulation
NM_001293685	*Vti1a*	-0.201873291	0.004871537	Mpst_Tg_Downregulation
NM_001130166	*Oxr1*	-0.20092596	0.02597524	Mpst_Tg_Downregulation
NM_001109753	*Sv2b*	-0.20090798	0.019489084	Mpst_Tg_Downregulation
NM_001037906	*Nell1*	-0.200256873	0.029221504	Mpst_Tg_Downregulation
NM_026021	*Zmynd19*	-0.199589551	0.019277268	Mpst_Tg_Downregulation
NM_001170694	*Rcbtb2*	-0.199497344	0.048203603	Mpst_Tg_Downregulation
NM_146028	*Stac2*	-0.199010617	0.008531653	Mpst_Tg_Downregulation
NM_008537	*Amacr*	-0.197781918	0.020485397	Mpst_Tg_Downregulation
NM_001146294	*Celf4*	-0.196461682	0.035473278	Mpst_Tg_Downregulation
NM_010895	*Neurod2*	-0.196394102	0.012687097	Mpst_Tg_Downregulation
NM_010173	*Faah*	-0.195986345	0.003983721	Mpst_Tg_Downregulation
NM_026345	*Mansc1*	-0.195903304	0.015169137	Mpst_Tg_Downregulation
NM_001033341	*Zfp407*	-0.195437928	0.005947567	Mpst_Tg_Downregulation
NM_001025388	*Eno1b*	-0.195314778	0.040557167	Mpst_Tg_Downregulation
NM_199024	*Nol4*	-0.195212819	0.004978701	Mpst_Tg_Downregulation
NM_010250	*Gabra1*	-0.195105799	0.025943181	Mpst_Tg_Downregulation
NM_001289658	*Agbl3*	-0.195046646	0.039814737	Mpst_Tg_Downregulation
NM_026878	*Rasl11b*	-0.195009631	0.043628677	Mpst_Tg_Downregulation
NM_001193305	*Mical2*	-0.191987864	0.036964614	Mpst_Tg_Downregulation
NM_028243	*Prcp*	-0.191536944	0.011631229	Mpst_Tg_Downregulation
NM_001177630	*Etl4*	-0.191403289	0.025059745	Mpst_Tg_Downregulation
NM_001081141	*Gabbr2*	-0.189657532	0.012750146	Mpst_Tg_Downregulation
NM_019942	*Sept6*	-0.189322389	0.015991753	Mpst_Tg_Downregulation
NM_029530	*Lamp5*	-0.189184084	0.003779955	Mpst_Tg_Downregulation
NM_001198968	*Itsn2*	-0.188233909	0.017853556	Mpst_Tg_Downregulation
NM_001177480	*Gm14391*	-0.188103408	0.04195279	Mpst_Tg_Downregulation
NM_001164294	*Cuedc2*	-0.187712424	0.046440533	Mpst_Tg_Downregulation
NM_027519	*Medag*	-0.186880534	0.011111501	Mpst_Tg_Downregulation
NM_028023	*Cdca4*	-0.184951554	0.005297096	Mpst_Tg_Downregulation
NM_001170800	*Ipcef1*	-0.184796662	0.039317265	Mpst_Tg_Downregulation
NM_053161	*Mrpl27*	-0.184629054	0.000434453	Mpst_Tg_Downregulation
NM_026790	*Ifi27*	-0.184336868	0.033049359	Mpst_Tg_Downregulation
NM_172885	*Tmem132d*	-0.184070205	0.047234969	Mpst_Tg_Downregulation
NM_019833	*Fam69b*	-0.18315225	0.028343453	Mpst_Tg_Downregulation
NM_133804	*Tmem132a*	-0.182607164	0.012456302	Mpst_Tg_Downregulation
NM_013546	*Hebp1*	-0.182358341	0.049076591	Mpst_Tg_Downregulation
NM_138956	*Rassf3*	-0.181812503	0.04440877	Mpst_Tg_Downregulation
NM_021452	*Kcnmb4*	-0.181662352	0.035397117	Mpst_Tg_Downregulation
NM_001012330	*Zbtb18*	-0.180394423	0.00812291	Mpst_Tg_Downregulation
NM_146244	*Rps6kl1*	-0.180235941	0.008489085	Mpst_Tg_Downregulation
NM_177640	*D030056L22Rik*	-0.18021049	0.002368656	Mpst_Tg_Downregulation
NM_015789	*Dkkl1*	-0.179335802	0.043825078	Mpst_Tg_Downregulation
NM_001166065	*Gcnt4*	-0.179322827	0.034925721	Mpst_Tg_Downregulation
NM_153529	*Nrn1*	-0.179215674	0.017691458	Mpst_Tg_Downregulation
NM_001081567	*Mapk10*	-0.179054693	0.001602144	Mpst_Tg_Downregulation
NM_145128	*Mgat5*	-0.178003741	0.022999787	Mpst_Tg_Downregulation
NM_198122	*Arid4b*	-0.177881682	0.02617739	Mpst_Tg_Downregulation
NM_001038010	*Kat2a*	-0.177846128	0.048588673	Mpst_Tg_Downregulation
NM_009904	*Clgn*	-0.177252879	0.026768581	Mpst_Tg_Downregulation
NM_008426	*Kcnj3*	-0.176792863	0.026523148	Mpst_Tg_Downregulation
NM_001042451	*Snca*	-0.176691461	0.019738716	Mpst_Tg_Downregulation
NM_001040087	*Sytl2*	-0.176384122	0.024570274	Mpst_Tg_Downregulation
NM_008384	*Inpp1*	-0.174971244	0.045312032	Mpst_Tg_Downregulation
NR_040411	*Gm11549*	-0.174694673	0.037846183	Mpst_Tg_Downregulation

NM_021877	*Hr*	-0.17469352	0.043659759	Mpst_Tg_Downregulation
NM_207220	*Gpr137*	-0.174599402	0.038930626	Mpst_Tg_Downregulation
NM_001114660	*Scfd2*	-0.17426944	0.039405686	Mpst_Tg_Downregulation
NM_016744	*Pde1a*	-0.174044255	0.031482511	Mpst_Tg_Downregulation
NM_028009	*Rpusd1*	-0.172952523	0.014533495	Mpst_Tg_Downregulation
NM_001163399	*Elavl4*	-0.172923048	0.048929928	Mpst_Tg_Downregulation
NM_175398	*Fam212b*	-0.172512524	0.009951728	Mpst_Tg_Downregulation
NM_001033908	*Med22*	-0.17196504	0.026360639	Mpst_Tg_Downregulation
NM_172701	*Oscp1*	-0.171146642	0.038096014	Mpst_Tg_Downregulation
NM_001164585	*Dnajc6*	-0.170999664	0.006668574	Mpst_Tg_Downregulation
NM_001159774	*Ikbkb*	-0.170377177	0.020217167	Mpst_Tg_Downregulation
NM_001276321	*Cyhr1*	-0.170352214	0.033857604	Mpst_Tg_Downregulation
NM_172829	*St6gal2*	-0.170111374	0.04180218	Mpst_Tg_Downregulation
NM_026255	*Slc25a26*	-0.169452423	0.047825606	Mpst_Tg_Downregulation
NM_026520	*Fam122a*	-0.169437726	0.015084016	Mpst_Tg_Downregulation
NM_177868	*Fhad1*	-0.169421392	0.047402348	Mpst_Tg_Downregulation
NM_001040026	*Sco1*	-0.168957601	0.005587215	Mpst_Tg_Downregulation
NM_011660	*Txn1*	-0.168837461	0.007199621	Mpst_Tg_Downregulation
NM_010699	*Ldha*	-0.168298677	0.018402606	Mpst_Tg_Downregulation
NM_026746	*Nsmce2*	-0.167726702	0.020792067	Mpst_Tg_Downregulation
NM_130858	*Nxph3*	-0.167174563	0.040665053	Mpst_Tg_Downregulation
NM_001024955	*Pik3r1*	-0.166843027	0.019081195	Mpst_Tg_Downregulation
NM_008826	*Pfkl*	-0.166120497	0.004411708	Mpst_Tg_Downregulation
NM_001252342	*Syt1*	-0.166070464	0.016305309	Mpst_Tg_Downregulation
NM_010316	*Gng3*	-0.165345236	0.009382054	Mpst_Tg_Downregulation
NM_025910	*Mina*	-0.164960839	0.039615175	Mpst_Tg_Downregulation
NM_001025309	*Pja2*	-0.164605902	0.037812748	Mpst_Tg_Downregulation
NM_172710	*Sel1l3*	-0.164506023	0.003921728	Mpst_Tg_Downregulation
NM_008202	*Slc39a7*	-0.164489489	0.025722043	Mpst_Tg_Downregulation
NM_027193	*Dph5*	-0.164351037	0.021268228	Mpst_Tg_Downregulation
NM_133803	*Dpp3*	-0.1640381	0.009673991	Mpst_Tg_Downregulation
NM_023456	*Npy*	-0.163367689	0.017220864	Mpst_Tg_Downregulation
NM_026998	*Snx6*	-0.163123475	0.024076896	Mpst_Tg_Downregulation
NM_134072	*Akr1c14*	-0.162377981	0.033521011	Mpst_Tg_Downregulation
NM_080428	*Fbxw7*	-0.162221594	0.013101816	Mpst_Tg_Downregulation
NM_026631	*Nhp2*	-0.161703991	0.039727693	Mpst_Tg_Downregulation
NM_008704	*Nme1*	-0.161346046	0.010343613	Mpst_Tg_Downregulation
NM_021322	*Wdr4*	-0.161161465	0.027117326	Mpst_Tg_Downregulation
NM_033074	*Tars*	-0.161150216	0.004655541	Mpst_Tg_Downregulation
NM_199447	*Rrp12*	-0.160944325	0.047314583	Mpst_Tg_Downregulation
NM_153405	*Rbm45*	-0.160002872	0.034829803	Mpst_Tg_Downregulation
NM_001039710	*Coq10b*	-0.159666213	0.030157439	Mpst_Tg_Downregulation
NM_001276763	*Brsk2*	-0.159449976	0.040682413	Mpst_Tg_Downregulation
NM_008429	*Kcnj9*	-0.158468494	0.016738032	Mpst_Tg_Downregulation
NM_001081007	*Zfp382*	-0.158263229	0.044065665	Mpst_Tg_Downregulation
NM_010191	*Fdft1*	-0.158244771	0.020225146	Mpst_Tg_Downregulation
NM_008983	*Ptprk*	-0.158123557	0.048557507	Mpst_Tg_Downregulation
NM_001289490	*Oard1*	-0.158107099	0.020515797	Mpst_Tg_Downregulation
NM_172631	*Ldlrad4*	-0.157421514	0.01520506	Mpst_Tg_Downregulation
NM_001015046	*Rap1gap2*	-0.157201995	0.001174058	Mpst_Tg_Downregulation
NM_178936	*Tmem56*	-0.155922572	0.047619084	Mpst_Tg_Downregulation
NM_181074	*Lingo1*	-0.15553936	0.040859693	Mpst_Tg_Downregulation
NM_011207	*Ptpn3*	-0.155475919	0.013969413	Mpst_Tg_Downregulation
NM_015730	*Chrna4*	-0.155470258	0.01356078	Mpst_Tg_Downregulation
NM_015807	*Nt5c*	-0.155174881	0.033260178	Mpst_Tg_Downregulation
NM_028705	*Herc3*	-0.154613863	0.039233691	Mpst_Tg_Downregulation
NM_001081035	*Nav3*	-0.154054563	0.025071263	Mpst_Tg_Downregulation
NM_053179	*Nans*	-0.153735509	0.04388278	Mpst_Tg_Downregulation
NM_023500	*Xk*	-0.153337982	0.023749936	Mpst_Tg_Downregulation
NM_009133	*Stmn3*	-0.153242949	0.00706875	Mpst_Tg_Downregulation
NM_008255	*Hmgcr*	-0.152965683	0.009750254	Mpst_Tg_Downregulation
NM_133939	*Lsm8*	-0.152898102	0.025687565	Mpst_Tg_Downregulation

NM_001301737	*Dnm1*	-0.15197546	0.041354497	Mpst_Tg_Downregulation
NM_013464	*Ahr*	-0.151703526	0.025665233	Mpst_Tg_Downregulation
NM_001289653	*Nell2*	-0.151380568	0.004417285	Mpst_Tg_Downregulation
NM_001164268	*Kalrn*	-0.150485594	0.017607892	Mpst_Tg_Downregulation
NM_026662	*Prps2*	-0.150389819	0.0487736	Mpst_Tg_Downregulation
NM_008730	*Nptx1*	-0.149806524	0.017660626	Mpst_Tg_Downregulation
NM_026406	*Rnf115*	-0.149582158	0.007685946	Mpst_Tg_Downregulation
NM_008792	*Pcsk2*	-0.149173287	0.005197477	Mpst_Tg_Downregulation
NM_001034115	*Shank1*	-0.14875022	0.048938457	Mpst_Tg_Downregulation
NM_008310	*Htr1f*	-0.148343114	0.025763399	Mpst_Tg_Downregulation
NM_170756	*Spata2*	-0.148169101	0.012500857	Mpst_Tg_Downregulation
NM_022029	*Nrgn*	-0.147849739	0.010026709	Mpst_Tg_Downregulation
NM_010158	*Khdrbs3*	-0.14780173	0.012801427	Mpst_Tg_Downregulation
NM_183147	*Sprn*	-0.147539393	0.015024978	Mpst_Tg_Downregulation
NM_133972	*Armc6*	-0.147158326	0.027606478	Mpst_Tg_Downregulation
NM_001007574	*A830010M20Rik*	-0.147127136	0.04484315	Mpst_Tg_Downregulation
NM_026618	*Coa3*	-0.146880717	0.019662965	Mpst_Tg_Downregulation
NM_023051	*Clstn1*	-0.146838375	0.039616297	Mpst_Tg_Downregulation
NM_001033305	*Ndufb6*	-0.146284471	0.038103691	Mpst_Tg_Downregulation
NM_172687	*Coq3*	-0.146079424	0.044697769	Mpst_Tg_Downregulation
NM_144900	*Atp1a1*	-0.145852125	0.025367619	Mpst_Tg_Downregulation
NM_175357	*Crbn*	-0.144785772	0.044213663	Mpst_Tg_Downregulation
NM_178939	*Pdrg1*	-0.144382447	0.048316435	Mpst_Tg_Downregulation
NM_007936	*Epha4*	-0.14428816	0.005986371	Mpst_Tg_Downregulation
NM_025994	*Efhd2*	-0.143457149	0.023761895	Mpst_Tg_Downregulation
NM_001083945	*Rsph3b*	-0.143443851	0.005885052	Mpst_Tg_Downregulation
NM_177003	*9630033F20Rik*	-0.143403602	0.022545219	Mpst_Tg_Downregulation
NM_133248	*Glmn*	-0.143344525	0.011504191	Mpst_Tg_Downregulation
NM_175259	*Shisa4*	-0.143163626	0.017674859	Mpst_Tg_Downregulation
NM_011838	*Lynx1*	-0.143138559	0.028254479	Mpst_Tg_Downregulation
NM_001024206	*Trappc1*	-0.143047781	0.018966841	Mpst_Tg_Downregulation
NM_172266	*Lpgat1*	-0.142626426	0.037590142	Mpst_Tg_Downregulation
NM_019824	*Arpc3*	-0.142625082	0.012383154	Mpst_Tg_Downregulation
NM_172610	*Mpped1*	-0.14258034	0.010317998	Mpst_Tg_Downregulation
NM_011293	*Polr2j*	-0.142536787	0.019057237	Mpst_Tg_Downregulation
NM_010305	*Gnai1*	-0.142088893	0.015705773	Mpst_Tg_Downregulation
NM_025347	*Ypel3*	-0.141940825	0.039404586	Mpst_Tg_Downregulation
NM_011428	*Snap25*	-0.141847655	0.007544207	Mpst_Tg_Downregulation
NM_001081022	*D430042O09Rik*	-0.141787712	0.00678262	Mpst_Tg_Downregulation
NM_011035	*Pak1*	-0.141586891	0.017314445	Mpst_Tg_Downregulation
NM_011967	*Psma5*	-0.14156806	0.01047383	Mpst_Tg_Downregulation
NM_023526	*Nkiras1*	-0.140815389	0.005149024	Mpst_Tg_Downregulation
NM_019816	*Aatf*	-0.140166832	0.033956393	Mpst_Tg_Downregulation
NM_001290750	*Rnf130*	-0.139451763	0.022925281	Mpst_Tg_Downregulation
NM_182993	*Slc17a7*	-0.138754447	0.007750293	Mpst_Tg_Downregulation
NM_133778	*Fam131a*	-0.138369625	0.00737619	Mpst_Tg_Downregulation
NM_026360	*Ddx47*	-0.137940043	0.005100824	Mpst_Tg_Downregulation
NM_011352	*Sema7a*	-0.137888694	0.026414916	Mpst_Tg_Downregulation
NM_153128	*Klhl12*	-0.137753344	0.033161054	Mpst_Tg_Downregulation
NM_146168	*Vopp1*	-0.137353754	0.002118628	Mpst_Tg_Downregulation
NM_001285813	*Dtna*	-0.13693105	0.04802815	Mpst_Tg_Downregulation
NM_001199227	*Osbpl5*	-0.136729546	0.033604098	Mpst_Tg_Downregulation
NM_024220	*Ndufc2*	-0.136651619	0.040459432	Mpst_Tg_Downregulation
NM_019535	*Sh3gl2*	-0.136555561	0.006451727	Mpst_Tg_Downregulation
NM_178920	*Mal2*	-0.136354413	0.009607727	Mpst_Tg_Downregulation
NM_010298	*Glrb*	-0.135728718	0.047755238	Mpst_Tg_Downregulation
NM_176930	*Nrcam*	-0.135559258	0.009633633	Mpst_Tg_Downregulation
NM_177893	*Fan1*	-0.13542429	0.023074709	Mpst_Tg_Downregulation
NM_201357	*Tssc1*	-0.135415643	0.016090809	Mpst_Tg_Downregulation
NM_180678	*Gars*	-0.135030855	0.004129292	Mpst_Tg_Downregulation
NM_001164256	*Tpm1*	-0.134732711	0.038252753	Mpst_Tg_Downregulation
NM_026638	*Cdipt*	-0.13350217	0.029697272	Mpst_Tg_Downregulation

NM_134010	*Nup107*	-0.133071593	0.023707868	Mpst_Tg_Downregulation
NM_008828	*Pgk1*	-0.133059228	0.02541593	Mpst_Tg_Downregulation
NM_001195004	*Kat7*	-0.132784249	0.020570323	Mpst_Tg_Downregulation
NM_008687	*Nfib*	-0.132782556	0.020667112	Mpst_Tg_Downregulation
NM_009898	*Coro1a*	-0.13272506	0.049561318	Mpst_Tg_Downregulation
NM_016801	*Stx1a*	-0.132361998	0.034745903	Mpst_Tg_Downregulation
NM_025417	*Commd4*	-0.131735455	0.017417116	Mpst_Tg_Downregulation
NM_009218	*Sstr3*	-0.131718066	0.018696279	Mpst_Tg_Downregulation
NM_134091	*Sgsm3*	-0.131541925	0.042637158	Mpst_Tg_Downregulation
NM_146130	*Hnrnpa3*	-0.131501284	0.043022631	Mpst_Tg_Downregulation
NM_009193	*Slbp*	-0.131047551	0.037815145	Mpst_Tg_Downregulation
NM_181850	*Grm3*	-0.130982825	0.017428364	Mpst_Tg_Downregulation
NM_153534	*Adcy2*	-0.129917486	0.003175972	Mpst_Tg_Downregulation
NM_177718	*Mb21d2*	-0.129911953	0.022220065	Mpst_Tg_Downregulation
NM_207237	*Man1c1*	-0.129881636	0.03683878	Mpst_Tg_Downregulation
NM_001142965	*Frrs1l*	-0.129835891	0.044558136	Mpst_Tg_Downregulation
NM_027412	*Ttc9c*	-0.129626118	0.007428812	Mpst_Tg_Downregulation
NM_009415	*Tpi1*	-0.129270597	0.027637679	Mpst_Tg_Downregulation
NM_026303	*Alkbh8*	-0.129107308	0.040875799	Mpst_Tg_Downregulation
NM_133700	*Btbd10*	-0.129096262	0.049656722	Mpst_Tg_Downregulation
NM_146165	*Aimp2*	-0.128971394	0.043942178	Mpst_Tg_Downregulation
NM_172621	*Clic5*	-0.128871744	0.031768923	Mpst_Tg_Downregulation
NM_021287	*Sptbn2*	-0.128684813	0.017485269	Mpst_Tg_Downregulation
NM_011876	*Twf2*	-0.12858156	0.009445991	Mpst_Tg_Downregulation
NM_173738	*Zfp940*	-0.128410073	0.034981462	Mpst_Tg_Downregulation
NM_001289826	*Clock*	-0.128247597	0.039594576	Mpst_Tg_Downregulation
NM_012021	*Prdx5*	-0.127466896	0.043383614	Mpst_Tg_Downregulation
NM_139154	*Rab40c*	-0.12708663	0.04678123	Mpst_Tg_Downregulation
NM_207625	*Acsl4*	-0.126608614	0.049500685	Mpst_Tg_Downregulation
NM_011510	*Abcc8*	-0.126434647	0.047334937	Mpst_Tg_Downregulation
NM_175494	*Zfp367*	-0.125898811	0.029385072	Mpst_Tg_Downregulation
NM_008323	*Idh3g*	-0.125688754	0.002218788	Mpst_Tg_Downregulation
NM_181517	*Ipo7*	-0.125486275	0.03384173	Mpst_Tg_Downregulation
NM_001081304	*Atf6*	-0.125332063	0.017262446	Mpst_Tg_Downregulation
NM_025358	*Ndufa9*	-0.124277605	0.009340883	Mpst_Tg_Downregulation
NM_019578	*Extl1*	-0.12388593	0.031115345	Mpst_Tg_Downregulation
NM_133783	*Ptges2*	-0.123756731	0.032935666	Mpst_Tg_Downregulation
NM_009897	*Ckmt1*	-0.123567818	0.045805325	Mpst_Tg_Downregulation
NM_009447	*Tuba4a*	-0.123328639	0.034735476	Mpst_Tg_Downregulation
NM_025494	*Atp6v1c1*	-0.12329249	0.02918633	Mpst_Tg_Downregulation
NM_025403	*Nop10*	-0.123138725	0.022698617	Mpst_Tg_Downregulation
NM_022992	*Arl6ip5*	-0.123031612	0.010457441	Mpst_Tg_Downregulation
NM_030004	*Cryl1*	-0.123002207	0.03524027	Mpst_Tg_Downregulation
NM_030252	*Smim12*	-0.122954602	0.029416558	Mpst_Tg_Downregulation
NM_026330	*Nsmce1*	-0.122566413	0.023769388	Mpst_Tg_Downregulation
NM_024260	*Ccdc132*	-0.122531769	0.032641256	Mpst_Tg_Downregulation
NM_009729	*Atp6v0c*	-0.122400391	0.045484085	Mpst_Tg_Downregulation
NM_019580	*Gde1*	-0.122209408	0.002372635	Mpst_Tg_Downregulation
NM_022653	*Thop1*	-0.122006373	0.030871444	Mpst_Tg_Downregulation
NM_026519	*Emc4*	-0.121617887	0.00847282	Mpst_Tg_Downregulation
NM_001081277	*Ak5*	-0.120779371	0.044027846	Mpst_Tg_Downregulation
NM_173430	*Fkrp*	-0.120708385	0.045476001	Mpst_Tg_Downregulation
NM_008923	*Prkar1b*	-0.120619674	0.011961177	Mpst_Tg_Downregulation
NM_145371	*Eif2b1*	-0.120559976	0.041459327	Mpst_Tg_Downregulation
NM_001135100	*Il34*	-0.119690105	0.035202004	Mpst_Tg_Downregulation
NM_133999	*Fig4*	-0.119477046	0.028184634	Mpst_Tg_Downregulation
NM_144838	*Sgtb*	-0.119409188	0.031156876	Mpst_Tg_Downregulation
NM_183146	*A530054K11Rik*	-0.119328746	0.010300083	Mpst_Tg_Downregulation
NM_028142	*Nsun4*	-0.119235643	0.048610493	Mpst_Tg_Downregulation
NM_172656	*Stradb*	-0.11921804	0.01191933	Mpst_Tg_Downregulation
NM_001243769	*Cdc42*	-0.119211844	0.038655859	Mpst_Tg_Downregulation
NM_027439	*Atp6ap2*	-0.118966981	0.004475117	Mpst_Tg_Downregulation

NM_007804	*Cux2*	-0.118832595	0.029567165	Mpst_Tg_Downregulation
NM_028292	*Ppme1*	-0.118563881	0.017543067	Mpst_Tg_Downregulation
NM_001290810	*Mrto4*	-0.118548243	0.049035296	Mpst_Tg_Downregulation
NM_007508	*Atp6v1a*	-0.118272495	0.041627464	Mpst_Tg_Downregulation
NM_025882	*Pole4*	-0.118052762	0.045573418	Mpst_Tg_Downregulation
NM_023328	*Agtpbp1*	-0.117063954	0.009109281	Mpst_Tg_Downregulation
NM_026502	*1110004E09Rik*	-0.116868237	0.024175057	Mpst_Tg_Downregulation
NM_134137	*Lars*	-0.116052023	0.021121359	Mpst_Tg_Downregulation
NM_026430	*Uxs1*	-0.115634676	0.029821338	Mpst_Tg_Downregulation
NM_172290	*Ntm*	-0.115171518	0.029612195	Mpst_Tg_Downregulation
NM_001109749	*Cntn4*	-0.115155501	0.022705013	Mpst_Tg_Downregulation
NM_009622	*Adcy1*	-0.11476662	0.041812537	Mpst_Tg_Downregulation
NM_012055	*Asns*	-0.114372355	0.001484293	Mpst_Tg_Downregulation
NM_026329	*Polr2g*	-0.114242986	0.011702807	Mpst_Tg_Downregulation
NM_001026212	*Ensa*	-0.114239925	0.021362604	Mpst_Tg_Downregulation
NM_007981	*Acsl1*	-0.114138016	0.032257126	Mpst_Tg_Downregulation
NM_001112705	*Tlk2*	-0.113494574	0.036174464	Mpst_Tg_Downregulation
NM_001142950	*Nars*	-0.112773821	0.037881064	Mpst_Tg_Downregulation
NM_182839	*Tppp*	-0.112713326	0.012135633	Mpst_Tg_Downregulation
NM_001024474	*Diras2*	-0.11214925	0.017938057	Mpst_Tg_Downregulation
NM_025959	*Psmc6*	-0.111943356	0.037281535	Mpst_Tg_Downregulation
NM_130884	*Idh3b*	-0.111824714	0.006755186	Mpst_Tg_Downregulation
NM_019584	*Becn1*	-0.111571766	0.000193774	Mpst_Tg_Downregulation
NM_175752	*Chn1*	-0.111419701	0.011986213	Mpst_Tg_Downregulation
NM_024475	*Ublcp1*	-0.111130616	0.029975434	Mpst_Tg_Downregulation
NM_008618	*Mdh1*	-0.110950068	0.034749891	Mpst_Tg_Downregulation
NM_007590	*Calm3*	-0.110769631	0.01882291	Mpst_Tg_Downregulation
NM_019967	*Brinp1*	-0.110715719	0.028545894	Mpst_Tg_Downregulation
NM_019754	*Tagln3*	-0.11025249	0.009465098	Mpst_Tg_Downregulation
NM_018818	*Chm*	-0.110134747	0.036145943	Mpst_Tg_Downregulation
NM_153459	*Dusp7*	-0.110027716	0.045285898	Mpst_Tg_Downregulation
NM_029505	*Ap3m2*	-0.109889226	0.031842376	Mpst_Tg_Downregulation
NM_183285	*Kctd2*	-0.109882646	0.024932365	Mpst_Tg_Downregulation
NM_199199	*Tmem199*	-0.109831132	0.044735105	Mpst_Tg_Downregulation
NM_177325	*Tsr1*	-0.109683352	0.04094662	Mpst_Tg_Downregulation
NM_018788	*Extl3*	-0.109561997	0.010274474	Mpst_Tg_Downregulation
NM_026272	*Narf*	-0.109557462	0.00164371	Mpst_Tg_Downregulation
NM_027886	*Stk11ip*	-0.108965361	0.020906	Mpst_Tg_Downregulation
NM_011181	*Cyth2*	-0.108911969	0.038152566	Mpst_Tg_Downregulation
NM_007547	*Sirpa*	-0.108891719	0.007087499	Mpst_Tg_Downregulation
NM_026056	*Cap2*	-0.108769063	0.032198457	Mpst_Tg_Downregulation
NM_030254	*Tusc3*	-0.108578659	0.013013161	Mpst_Tg_Downregulation
NM_027375	*Gcc2*	-0.108123203	0.030618013	Mpst_Tg_Downregulation
NR_002890	*Gm12070*	-0.107988724	0.045506586	Mpst_Tg_Downregulation
NM_001110240	*Slc24a2*	-0.1079869	0.049492982	Mpst_Tg_Downregulation
NM_153542	*Lrrc20*	-0.107598912	0.015946817	Mpst_Tg_Downregulation
NM_026735	*Mob1b*	-0.107105123	0.0329543	Mpst_Tg_Downregulation
NM_019811	*Acss2*	-0.107084702	0.010292814	Mpst_Tg_Downregulation
NM_013715	*Cops5*	-0.107031714	0.027552012	Mpst_Tg_Downregulation
NM_001001885	*Tmem151a*	-0.106727925	0.043570415	Mpst_Tg_Downregulation
NM_029920	*Mtus2*	-0.106655796	0.001460254	Mpst_Tg_Downregulation
NM_175561	*Pcnxl2*	-0.106615834	0.003289426	Mpst_Tg_Downregulation
NM_019703	*Pfkp*	-0.106188837	0.035751828	Mpst_Tg_Downregulation
NM_018743	*Agpat6*	-0.106042435	0.019838048	Mpst_Tg_Downregulation
NM_138306	*Dgkz*	-0.10579696	0.028266393	Mpst_Tg_Downregulation
NM_025808	*Lztr1*	-0.105667757	0.021696058	Mpst_Tg_Downregulation
NM_001199676	*Camkk2*	-0.105636925	0.032077306	Mpst_Tg_Downregulation
NM_176928	*Brwd1*	-0.104986574	0.045777508	Mpst_Tg_Downregulation
NM_029665	*Ipo11*	-0.10486486	0.033430209	Mpst_Tg_Downregulation
NM_001081282	*Ibtk*	-0.10461561	0.019988283	Mpst_Tg_Downregulation
NM_028097	*Tmem68*	-0.10451479	0.031850148	Mpst_Tg_Downregulation
NM_023119	*Eno1*	-0.104310956	0.045990549	Mpst_Tg_Downregulation

NM_181415	*Atrnl1*	-0.104272608	0.02511101	Mpst_Tg_Downregulation
NM_019780	*Vps29*	-0.104184709	0.00773795	Mpst_Tg_Downregulation
NM_007589	*Calm2*	-0.104020732	0.036097988	Mpst_Tg_Downregulation
NM_008551	*Mapkapk2*	-0.10400443	0.031622499	Mpst_Tg_Downregulation
NM_133826	*Atp6v1h*	-0.103692848	0.013343954	Mpst_Tg_Downregulation
NM_152810	*Cdc5l*	-0.103464795	0.025007912	Mpst_Tg_Downregulation
NM_001163487	*Pfkm*	-0.103441669	0.0443305	Mpst_Tg_Downregulation
NM_009382	*Thy1*	-0.103217837	0.022222847	Mpst_Tg_Downregulation
NM_025337	*Akr7a5*	-0.103200188	0.039279279	Mpst_Tg_Downregulation
NM_026545	*Psmd8*	-0.103135319	0.030554078	Mpst_Tg_Downregulation
NM_025976	*Bfar*	-0.101985093	0.009938944	Mpst_Tg_Downregulation
NM_011506	*Sucla2*	-0.101880065	0.035722667	Mpst_Tg_Downregulation
NM_025542	*Rtfdc1*	-0.101684377	0.016524413	Mpst_Tg_Downregulation
NM_009157	*Map2k4*	-0.101501633	0.021771812	Mpst_Tg_Downregulation
NM_026737	*Phf5a*	-0.100940577	0.038591348	Mpst_Tg_Downregulation
NM_018811	*Abhd2*	-0.100739336	0.011051709	Mpst_Tg_Downregulation
NM_178628	*Atl1*	-0.100232195	0.038250694	Mpst_Tg_Downregulation
NM_172723	*Adap1*	-0.099897571	0.005510389	Mpst_Tg_Downregulation
NM_146107	*Actr1b*	-0.099811025	0.034603034	Mpst_Tg_Downregulation
NM_178635	*Uvrag*	-0.099779068	0.0428808	Mpst_Tg_Downregulation
NM_007808	*Cycs*	-0.099391566	0.011576816	Mpst_Tg_Downregulation
NM_025418	*Vta1*	-0.098839897	0.024360537	Mpst_Tg_Downregulation
NM_001205361	*Dcun1d1*	-0.09868952	0.013219269	Mpst_Tg_Downregulation
NM_019913	*Txn2*	-0.098111849	0.046209441	Mpst_Tg_Downregulation
NM_001081045	*Kansl1*	-0.097482429	0.011494617	Mpst_Tg_Downregulation
NM_025283	*Mob4*	-0.097338576	0.0215181	Mpst_Tg_Downregulation
NM_018871	*Ywhag*	-0.097248971	0.041654879	Mpst_Tg_Downregulation
NM_019571	*Tspan5*	-0.096597959	0.027057882	Mpst_Tg_Downregulation
NM_026095	*Snrpd3*	-0.096475734	0.025903319	Mpst_Tg_Downregulation
NM_013556	*Hprt*	-0.096245562	0.029120968	Mpst_Tg_Downregulation
NM_019972	*Sort1*	-0.096114152	0.042326544	Mpst_Tg_Downregulation
NM_001014973	*Snx13*	-0.09555263	0.038248497	Mpst_Tg_Downregulation
NM_133722	*Abhd17c*	-0.095103237	0.035744652	Mpst_Tg_Downregulation
NM_028664	*Ankrd45*	-0.094833004	0.010669775	Mpst_Tg_Downregulation
NM_207583	*Brinp2*	-0.09440623	0.049236452	Mpst_Tg_Downregulation
NM_133764	*Atp6v0e2*	-0.094248809	0.013572522	Mpst_Tg_Downregulation
NM_172595	*Arl15*	-0.094071489	0.012777551	Mpst_Tg_Downregulation
NM_007698	*Chrm1*	-0.093142219	0.035688104	Mpst_Tg_Downregulation
NM_134101	*Psmd2*	-0.092949638	0.045839801	Mpst_Tg_Downregulation
NM_178624	*Fbxl2*	-0.092946458	0.016275268	Mpst_Tg_Downregulation
NM_029645	*Gatc*	-0.09283744	0.024684954	Mpst_Tg_Downregulation
NM_144534	*Tmem38a*	-0.092731391	0.037045156	Mpst_Tg_Downregulation
NM_001035228	*St3gal5*	-0.092647335	0.032180899	Mpst_Tg_Downregulation
NM_013812	*Cdk2ap1*	-0.092555848	0.032666006	Mpst_Tg_Downregulation
NM_023579	*Ipo5*	-0.092339456	0.010778227	Mpst_Tg_Downregulation
NM_011738	*Ywhah*	-0.092159199	0.027211958	Mpst_Tg_Downregulation
NM_025987	*Ndufa6*	-0.091745877	0.026229025	Mpst_Tg_Downregulation
NM_007450	*Slc25a4*	-0.091703199	0.022488812	Mpst_Tg_Downregulation
NM_025898	*Napa*	-0.091353901	0.028361444	Mpst_Tg_Downregulation
NM_028207	*Dusp3*	-0.090563736	0.043648587	Mpst_Tg_Downregulation
NM_010687	*Large*	-0.090420523	0.006223962	Mpst_Tg_Downregulation
NM_001004365	*Actr3b*	-0.090111563	0.026755637	Mpst_Tg_Downregulation
NM_054097	*Pip4k2c*	-0.089996721	0.035596689	Mpst_Tg_Downregulation
NM_134040	*Ddx1*	-0.089616604	0.029211159	Mpst_Tg_Downregulation
NM_172920	*Dpy19l1*	-0.089607286	0.048671971	Mpst_Tg_Downregulation
NM_029478	*Vmp1*	-0.089421636	0.029652902	Mpst_Tg_Downregulation
NM_025589	*Rpl36al*	-0.088170993	0.042810646	Mpst_Tg_Downregulation
NM_019668	*Ube2a*	-0.087853057	0.019137353	Mpst_Tg_Downregulation
NM_026157	*Mtpap*	-0.087782222	0.030082536	Mpst_Tg_Downregulation
NM_001098476	*Grsf1*	-0.087521697	0.037476806	Mpst_Tg_Downregulation
NM_001159591	*Csnk1g2*	-0.08751045	0.038300198	Mpst_Tg_Downregulation
NM_026374	*Ilf2*	-0.087269301	0.017534292	Mpst_Tg_Downregulation

NM_001302970	*Ap2m1*	-0.086833961	0.013758707	Mpst_Tg_Downregulation
NM_021526	*Psmd14*	-0.086832984	0.032457237	Mpst_Tg_Downregulation
NM_213614	*Sept5*	-0.086160961	0.003181681	Mpst_Tg_Downregulation
NM_145544	*Rap1gds1*	-0.08572457	0.037196473	Mpst_Tg_Downregulation
NM_026178	*Mmd*	-0.08527577	0.034891393	Mpst_Tg_Downregulation
NM_019742	*Tusc2*	-0.084223596	0.017809536	Mpst_Tg_Downregulation
NM_026620	*Fam98b*	-0.083957679	0.02362829	Mpst_Tg_Downregulation
NM_144518	*2900011O08Rik*	-0.083586744	0.042669691	Mpst_Tg_Downregulation
NM_020569	*Park7*	-0.0830706	0.03197068	Mpst_Tg_Downregulation
NM_023179	*Atp6v1g2*	-0.082672119	0.016790598	Mpst_Tg_Downregulation
NM_153407	*Csrnp2*	-0.082654788	0.016621739	Mpst_Tg_Downregulation
NR_104484	*Erc1*	-0.082599531	0.005780266	Mpst_Tg_Downregulation
NM_175104	*Fam53c*	-0.082590503	0.016135495	Mpst_Tg_Downregulation
NM_201371	*Prmt8*	-0.082273854	0.042776405	Mpst_Tg_Downregulation
NM_018883	*Camkk1*	-0.082053391	0.010470004	Mpst_Tg_Downregulation
NM_133907	*Ube3c*	-0.081929035	0.006588399	Mpst_Tg_Downregulation
NM_026048	*Cinp*	-0.081621107	0.017609861	Mpst_Tg_Downregulation
NM_013562	*Ifrd1*	-0.081566816	0.049941976	Mpst_Tg_Downregulation
NM_013477	*Atp6v0d1*	-0.08129969	0.037531594	Mpst_Tg_Downregulation
NM_133905	*Papd4*	-0.081105705	0.03567825	Mpst_Tg_Downregulation
NM_053076	*Rtn3*	-0.080424978	0.03398971	Mpst_Tg_Downregulation
NM_011188	*Psmc2*	-0.080388505	0.013556679	Mpst_Tg_Downregulation
NM_019961	*Pex3*	-0.078859121	0.036136272	Mpst_Tg_Downregulation
NM_001033213	*Ttc7b*	-0.078410726	0.022369955	Mpst_Tg_Downregulation
NM_001177667	*Sptan1*	-0.078396043	0.023664499	Mpst_Tg_Downregulation
NM_019914	*Mllt11*	-0.077928494	0.001784125	Mpst_Tg_Downregulation
NM_175226	*Rnf139*	-0.077241446	0.013733748	Mpst_Tg_Downregulation
NM_172784	*Lrp11*	-0.076775973	0.008846998	Mpst_Tg_Downregulation
NM_018740	*Elp5*	-0.075923374	0.030242431	Mpst_Tg_Downregulation
NM_020050	*Tmem9b*	-0.075760925	0.01429559	Mpst_Tg_Downregulation
NM_007509	*Atp6v1b2*	-0.075733455	0.043867124	Mpst_Tg_Downregulation
NM_008810	*Pdha1*	-0.075666812	0.048172588	Mpst_Tg_Downregulation
NM_144786	*Ggt7*	-0.074703446	0.047638349	Mpst_Tg_Downregulation
NM_025997	*Fam103a1*	-0.07438395	0.039099878	Mpst_Tg_Downregulation
NM_197987	*Trim37*	-0.073549345	0.025970201	Mpst_Tg_Downregulation
NM_029157	*Sf3a3*	-0.072786907	0.044470341	Mpst_Tg_Downregulation
NM_001007596	*Rtn1*	-0.072335791	0.011830792	Mpst_Tg_Downregulation
NM_021435	*Slc35b4*	-0.071985374	0.031702734	Mpst_Tg_Downregulation
NM_146200	*Eif3c*	-0.071751674	0.035604345	Mpst_Tg_Downregulation
NM_021463	*Prps1*	-0.07099291	0.014761718	Mpst_Tg_Downregulation
NM_173406	*Jazf1*	-0.070397972	0.041951654	Mpst_Tg_Downregulation
NM_013686	*Tcp1*	-0.070205986	0.016781232	Mpst_Tg_Downregulation
NM_020578	*Ehd3*	-0.068208153	0.031155422	Mpst_Tg_Downregulation
NM_027130	*Afg3l2*	-0.066716688	0.039076086	Mpst_Tg_Downregulation
NM_030018	*Tmem50b*	-0.065647068	0.03623429	Mpst_Tg_Downregulation
NM_001029934	*Usp32*	-0.0654345	0.039891817	Mpst_Tg_Downregulation
NM_001081203	*Sbno1*	-0.065292728	0.007186453	Mpst_Tg_Downregulation
NM_027258	*Rnf157*	-0.065114184	0.047512884	Mpst_Tg_Downregulation
NM_009031	*Rbbp7*	-0.064090793	0.023669037	Mpst_Tg_Downregulation
NM_133789	*Strn4*	-0.063827687	0.014877734	Mpst_Tg_Downregulation
NM_023374	*Sdhb*	-0.063554817	0.04832766	Mpst_Tg_Downregulation
NM_025558	*Cyb5b*	-0.063278069	0.012283675	Mpst_Tg_Downregulation
NM_001001181	*BC031181*	-0.061327837	0.036410486	Mpst_Tg_Downregulation
NM_018804	*Syt11*	-0.058176952	0.014705177	Mpst_Tg_Downregulation
NM_028759	*Dcaf6*	-0.058013147	0.045684469	Mpst_Tg_Downregulation
NM_012003	*Cops7a*	-0.053618457	0.028129707	Mpst_Tg_Downregulation
NM_178367	*Dhx33*	-0.053565352	0.021116397	Mpst_Tg_Downregulation
NM_023764	*Tollip*	-0.052003077	0.009498931	Mpst_Tg_Downregulation
NM_020332	*Ank*	-0.049006341	0.045336741	Mpst_Tg_Downregulation
NM_173781	*Rab6b*	-0.048855791	0.038624278	Mpst_Tg_Downregulation
NM_172994	*Ppp2r2c*	-0.046915209	0.046238508	Mpst_Tg_Downregulation
NM_001013380	*Dync1li2*	-0.045821183	0.009406911	Mpst_Tg_Downregulation

NM_173180	*Pmpca*	-0.044934372	0.049000257	Mpst_Tg_Downregulation
NM_001166591	*Eif5a*	7.387618308	0.005075079	Mpst_Tg_upregulation
NM_001163488	*Pfkm*	6.897949734	0.014506251	Mpst_Tg_upregulation
NM_032460	*Gpank1*	6.598013966	0.014335423	Mpst_Tg_upregulation
NM_001290799	*Inpp4a*	6.279024556	0.024938186	Mpst_Tg_upregulation
NM_009732	*Avp*	6.273780295	0.049380838	Mpst_Tg_upregulation
NM_001304774	*Pex26*	6.051214751	0.014183093	Mpst_Tg_upregulation
NM_138670	*Mpst*	5.741195897	0.00362056	Mpst_Tg_upregulation
NR_028297	*Fance*	5.517812722	0.039142146	Mpst_Tg_upregulation
NM_013634	*Med1*	5.395702713	0.018083964	Mpst_Tg_upregulation
NM_133759	*Zbtb3*	5.324239059	0.047403772	Mpst_Tg_upregulation
NM_001256518	*Zfp672*	4.846993934	0.047395517	Mpst_Tg_upregulation
NM_001276485	*Dag1*	3.890312153	0.02286984	Mpst_Tg_upregulation
NM_001162493	*Mpst*	2.390038181	0.000263594	Mpst_Tg_upregulation
NM_001293780	*Chtop*	1.664779482	0.010504483	Mpst_Tg_upregulation
NM_177914	*Dgkk*	1.464243276	0.048439822	Mpst_Tg_upregulation
NM_001042614	*Sepp1*	1.458120113	0.013140222	Mpst_Tg_upregulation
NR_046280	*Zfp672*	1.397928738	0.006991662	Mpst_Tg_upregulation
NM_001142760	*Pcdh15*	1.38448399	0.012854497	Mpst_Tg_upregulation
NM_001167925	*A730017C20Rik*	1.315168087	0.001064471	Mpst_Tg_upregulation
NM_001163270	*Baiap3*	1.267900359	0.033980754	Mpst_Tg_upregulation
NM_001302207	*Flii*	1.20898847	0.00844587	Mpst_Tg_upregulation
NM_001289432	*Cipc*	1.171724134	0.018761239	Mpst_Tg_upregulation
NM_001287058	*Vegfa*	1.110373362	0.028313192	Mpst_Tg_upregulation
NM_013496	*Crabp1*	1.079916709	0.000925227	Mpst_Tg_upregulation
NM_001287800	*Fhl1*	1.068758262	0.024268346	Mpst_Tg_upregulation
NM_001291121	*Epb4.1l1*	1.055073392	0.046050147	Mpst_Tg_upregulation
NM_001289695	*Zfp740*	1.043149782	0.023762694	Mpst_Tg_upregulation
NM_007428	*Agt*	0.994176275	0.017304934	Mpst_Tg_upregulation
NM_001159941	*Kctd10*	0.992524297	0.048002719	Mpst_Tg_upregulation
NM_008316	*Hus1*	0.975106622	0.000995095	Mpst_Tg_upregulation
NR_033144	*4921524J17Rik*	0.971535691	0.028070939	Mpst_Tg_upregulation
NM_175363	*Pphln1*	0.941852264	0.031415234	Mpst_Tg_upregulation
NM_001291238	*Cux1*	0.918689031	0.007379071	Mpst_Tg_upregulation
NM_001034962	*Sorbs1*	0.912009989	0.031879948	Mpst_Tg_upregulation
NM_025312	*Sostdc1*	0.908001672	0.032714356	Mpst_Tg_upregulation
NM_001134461	*Prickle2*	0.880895259	0.010020172	Mpst_Tg_upregulation
NM_013758	*Add3*	0.879950327	0.0208621	Mpst_Tg_upregulation
NM_026082	*Dock7*	0.874812809	0.036486588	Mpst_Tg_upregulation
NM_015749	*Tcn2*	0.862496042	0.025449109	Mpst_Tg_upregulation
NM_021382	*Tacr3*	0.823570365	0.024639904	Mpst_Tg_upregulation
NM_001286541	*Sigmar1*	0.806214553	0.041451751	Mpst_Tg_upregulation
NM_013823	*Kl*	0.803050575	0.019636849	Mpst_Tg_upregulation
NM_053270	*Rims1*	0.765630812	0.006792607	Mpst_Tg_upregulation
NM_013484	*C2*	0.749006642	0.031037923	Mpst_Tg_upregulation
NM_026868	*Abhd13*	0.737236379	0.020408003	Mpst_Tg_upregulation
NM_001122989	*Cdc14b*	0.733142503	0.002711153	Mpst_Tg_upregulation
NM_173052	*Serpinb1b*	0.72735553	0.010714314	Mpst_Tg_upregulation
NM_016862	*Vti1a*	0.719758779	0.01049632	Mpst_Tg_upregulation
NM_026952	*Cdk12*	0.705692574	0.03285241	Mpst_Tg_upregulation
NM_023440	*Tmem86b*	0.699949035	0.004671201	Mpst_Tg_upregulation
NM_001294323	*Zfp2*	0.694446568	0.010842953	Mpst_Tg_upregulation
NM_177981	*Hap1*	0.688415241	0.019065438	Mpst_Tg_upregulation
NM_001025382	*Fam196b*	0.686647266	0.033222659	Mpst_Tg_upregulation
NM_001193659	*Cldn12*	0.678902242	0.04660872	Mpst_Tg_upregulation
NM_031875	*Otof*	0.677000809	0.030801955	Mpst_Tg_upregulation
NM_001276681	*Syt6*	0.672855894	0.035017804	Mpst_Tg_upregulation
NR_125833	*Clasrp*	0.671998313	0.004108149	Mpst_Tg_upregulation
NM_144917	*Elmod3*	0.658132847	0.027044028	Mpst_Tg_upregulation
NR_027853	*Clk1*	0.646661648	0.002928094	Mpst_Tg_upregulation
NM_001291930	*Adora1*	0.645960006	0.015239215	Mpst_Tg_upregulation
NM_001205236	*Arhgap27*	0.638662152	0.016029041	Mpst_Tg_upregulation

NM_009697	*Nr2f2*	0.621934897	0.011010098	Mpst_Tg_upregulation
NR_033218	*Rreb1*	0.621245905	0.026771071	Mpst_Tg_upregulation
NR_002167	*E530001F21Rik*	0.617139707	0.027977218	Mpst_Tg_upregulation
NM_170689	*Ank3*	0.609650023	0.026530981	Mpst_Tg_upregulation
NM_026127	*4833420G17Rik*	0.602788908	0.013299321	Mpst_Tg_upregulation
NM_001170746	*Magi2*	0.602236132	0.009939889	Mpst_Tg_upregulation
NM_016697	*Gpc3*	0.598502516	0.043052935	Mpst_Tg_upregulation
NM_001164187	*Nagk*	0.597140318	0.047692263	Mpst_Tg_upregulation
NM_001161369	*Ss18*	0.594622532	0.046949646	Mpst_Tg_upregulation
NM_026149	*Nudcd1*	0.590828682	0.047033924	Mpst_Tg_upregulation
NM_011569	*Tekt1*	0.589397282	0.042411668	Mpst_Tg_upregulation
NM_133364	*Prima1*	0.588730112	0.046133992	Mpst_Tg_upregulation
NR_038158	*5730405O15Rik*	0.577359471	0.031266558	Mpst_Tg_upregulation
NM_011365	*Itsn2*	0.57415686	0.035813398	Mpst_Tg_upregulation
NR_033133	*Plscr3*	0.571780746	0.008671961	Mpst_Tg_upregulation
NM_001165949	*Pias3*	0.571628034	0.00048612	Mpst_Tg_upregulation
NM_172144	*Ppp2r3a*	0.562748484	0.011964685	Mpst_Tg_upregulation
NM_008731	*Npy2r*	0.540671436	0.00450017	Mpst_Tg_upregulation
NM_001134391	*Apobec1*	0.539171439	0.023546713	Mpst_Tg_upregulation
NM_001286986	*Dram2*	0.53576531	0.017213782	Mpst_Tg_upregulation
NM_008708	*Nmt2*	0.533151743	0.02932796	Mpst_Tg_upregulation
NM_001122766	*Sirt2*	0.529312207	0.026668396	Mpst_Tg_upregulation
NM_207176	*Tes*	0.527799197	0.034341014	Mpst_Tg_upregulation
NM_175344	*Ano6*	0.5275823	0.005519219	Mpst_Tg_upregulation
NM_001289925	*Egr3*	0.525373119	0.024530695	Mpst_Tg_upregulation
NM_001079513	*Zbtb33*	0.515188756	0.007942157	Mpst_Tg_upregulation
NM_029612	*Slamf9*	0.506034794	0.02218719	Mpst_Tg_upregulation
NM_001099275	*Tekt5*	0.504765785	0.019604402	Mpst_Tg_upregulation
NM_007715	*Clock*	0.503122525	0.042761337	Mpst_Tg_upregulation
NM_028325	*Zcchc12*	0.50179745	0.015279963	Mpst_Tg_upregulation
NM_013696	*Trhr*	0.497688851	0.001915495	Mpst_Tg_upregulation
NM_183319	*Xkrx*	0.492132939	0.00680671	Mpst_Tg_upregulation
NM_001081052	*Nhs*	0.488801783	0.04618749	Mpst_Tg_upregulation
NM_026451	*1600012H06Rik*	0.48556293	0.024988686	Mpst_Tg_upregulation
NM_001297596	*Inpp4b*	0.484248784	0.004481094	Mpst_Tg_upregulation
NM_001302834	*Nsun7*	0.482670252	0.004860885	Mpst_Tg_upregulation
NM_198410	*Paqr6*	0.482554906	0.022809177	Mpst_Tg_upregulation
NM_001286602	*Clasp2*	0.480185641	0.013685467	Mpst_Tg_upregulation
NM_001172121	*Rbms3*	0.480166823	0.016324181	Mpst_Tg_upregulation
NM_001033248	*Gm266*	0.478554042	0.045725557	Mpst_Tg_upregulation
NM_019691	*Gria4*	0.473843976	0.034829424	Mpst_Tg_upregulation
NM_001168623	*Znrf1*	0.473258904	0.046748728	Mpst_Tg_upregulation
NM_001285810	*Dtna*	0.472573525	0.01789425	Mpst_Tg_upregulation
NM_172205	*Sbsn*	0.471802203	0.041171612	Mpst_Tg_upregulation
NM_145135	*Rnh1*	0.46996317	0.046752355	Mpst_Tg_upregulation
NM_172930	*Tmem255a*	0.4693485	0.001736401	Mpst_Tg_upregulation
NM_001039676	*Slc39a2*	0.467345974	0.021954212	Mpst_Tg_upregulation
NM_016768	*Pbx3*	0.466643248	0.033806044	Mpst_Tg_upregulation
NM_008312	*Htr2c*	0.460552204	0.007700518	Mpst_Tg_upregulation
NM_001080795	*G3bp2*	0.451875856	0.008513336	Mpst_Tg_upregulation
NM_153140	*Rab11fip3*	0.45080108	0.030855503	Mpst_Tg_upregulation
NM_008097	*Gcdh*	0.447663454	0.044745105	Mpst_Tg_upregulation
NM_026167	*Klhl13*	0.447052643	0.035227973	Mpst_Tg_upregulation
NM_001291349	*Anapc15*	0.445182349	0.018644605	Mpst_Tg_upregulation
NM_001033442	*Gm1604b*	0.444851551	0.03140467	Mpst_Tg_upregulation
NM_001037717	*Slc38a6*	0.443970798	0.004853968	Mpst_Tg_upregulation
NM_001290419	*Snapc4*	0.441130525	0.013335566	Mpst_Tg_upregulation
NM_001159486	*Mcf2l*	0.43896118	0.022797737	Mpst_Tg_upregulation
NM_030700	*Maged2*	0.438046836	0.030945514	Mpst_Tg_upregulation
NM_153118	*Fnbp1l*	0.431062651	0.001530338	Mpst_Tg_upregulation
NM_010404	*Hap1*	0.430690132	0.030804146	Mpst_Tg_upregulation
NR_045399	*Gm15328*	0.429963758	0.03912518	Mpst_Tg_upregulation

NM_001290817	*Sparc*	0.429894308	0.004531893	Mpst_Tg_upregulation
NM_019397	*Egfl6*	0.429295794	0.049350045	Mpst_Tg_upregulation
NM_025771	*Cntnap2*	0.427615311	0.019844676	Mpst_Tg_upregulation
NM_146012	*Ctdsp2*	0.424627443	0.012315511	Mpst_Tg_upregulation
NM_177704	*Sytl5*	0.423627032	0.020970297	Mpst_Tg_upregulation
NM_001102436	*Acbd5*	0.420988179	0.005224411	Mpst_Tg_upregulation
NR_028364	*AI450353*	0.414677455	0.047299439	Mpst_Tg_upregulation
NR_045297	*Gm17644*	0.408993733	0.007513358	Mpst_Tg_upregulation
NM_001040072	*Nynrin*	0.404860363	0.026722494	Mpst_Tg_upregulation
NM_001080127	*Rnps1*	0.403597777	0.033298632	Mpst_Tg_upregulation
NM_001039151	*Cd44*	0.398299964	0.005828315	Mpst_Tg_upregulation
NM_001081243	*Filip1*	0.395837019	0.021167258	Mpst_Tg_upregulation
NM_007696	*Ovgp1*	0.389285465	0.032983544	Mpst_Tg_upregulation
NM_001081153	*Unc13c*	0.384959196	0.006651293	Mpst_Tg_upregulation
NM_023386	*Rtp4*	0.384502342	0.0055665	Mpst_Tg_upregulation
NM_010225	*Foxf2*	0.384416546	0.029575299	Mpst_Tg_upregulation
NM_011978	*Slc27a2*	0.382987193	0.037224058	Mpst_Tg_upregulation
NR_037983	*9330159M07Rik*	0.381307451	0.035419267	Mpst_Tg_upregulation
NM_016672	*Ddc*	0.376424964	0.027695952	Mpst_Tg_upregulation
NM_173016	*Vat1l*	0.371443362	0.019361933	Mpst_Tg_upregulation
NR_029443	*4933407K13Rik*	0.371070587	0.002391546	Mpst_Tg_upregulation
NM_001077266	*Hnrnpd*	0.369839549	0.046394462	Mpst_Tg_upregulation
NM_026724	*Rpl34*	0.367957646	0.03111007	Mpst_Tg_upregulation
NM_001162934	*Ctxn2*	0.367827645	0.005914053	Mpst_Tg_upregulation
NR_102277	*C030029H02Rik*	0.366853512	0.02746743	Mpst_Tg_upregulation
NM_001290288	*Samd15*	0.366369042	0.021045574	Mpst_Tg_upregulation
NM_026855	*Arv1*	0.366018051	0.011568065	Mpst_Tg_upregulation
NM_001081338	*L3mbtl1*	0.365665764	0.007935998	Mpst_Tg_upregulation
NM_030066	*Armcx1*	0.364103505	0.048726045	Mpst_Tg_upregulation
NM_007671	*Cdkn2c*	0.361615616	0.026764243	Mpst_Tg_upregulation
NM_001038610	*Dach1*	0.359599869	0.027328071	Mpst_Tg_upregulation
NM_001271898	*Acox1*	0.359025701	0.004771865	Mpst_Tg_upregulation
NM_170778	*Dpyd*	0.354765883	0.008054454	Mpst_Tg_upregulation
NM_019588	*Plce1*	0.353379929	0.010214396	Mpst_Tg_upregulation
NR_027388	*1700096K18Rik*	0.352159051	0.025137476	Mpst_Tg_upregulation
NM_001291115	*Rbm39*	0.351156484	0.02072752	Mpst_Tg_upregulation
NM_008397	*Itga6*	0.349958257	0.001513489	Mpst_Tg_upregulation
NM_001193271	*Meis1*	0.343503616	0.031739728	Mpst_Tg_upregulation
NM_001115151	*Uspl1*	0.343501852	0.041098927	Mpst_Tg_upregulation
NM_133990	*Il13ra1*	0.342273818	0.001299358	Mpst_Tg_upregulation
NM_178227	*Scn3b*	0.342139512	0.007430974	Mpst_Tg_upregulation
NM_024474	*Col26a1*	0.341828716	0.032385342	Mpst_Tg_upregulation
NR_003633	*Meg3*	0.341116811	0.021363229	Mpst_Tg_upregulation
NM_080466	*Kcnn3*	0.338739717	0.017661979	Mpst_Tg_upregulation
NM_001077709	*Slc39a7*	0.337335398	0.002226705	Mpst_Tg_upregulation
NM_001082962	*Snrpn*	0.332990198	0.005834529	Mpst_Tg_upregulation
NM_172485	*Thsd7b*	0.328180365	0.01658178	Mpst_Tg_upregulation
NM_021416	*Fam184b*	0.326912349	0.020300646	Mpst_Tg_upregulation
NM_175215	*Lysmd4*	0.322326647	0.030568263	Mpst_Tg_upregulation
NM_009145	*Nptn*	0.320034429	0.012031161	Mpst_Tg_upregulation
NM_011307	*Uimc1*	0.319144924	0.015869102	Mpst_Tg_upregulation
NM_198037	*Cachd1*	0.318933553	0.005171233	Mpst_Tg_upregulation
NM_001081263	*Slc44a5*	0.318119005	0.006021245	Mpst_Tg_upregulation
NM_028398	*Phykpl*	0.317904292	0.049287929	Mpst_Tg_upregulation
NM_001042670	*Mterf1b*	0.314823019	0.011991715	Mpst_Tg_upregulation
NM_001289474	*Erbb2ip*	0.314333165	0.012626628	Mpst_Tg_upregulation
NM_145492	*Zfp521*	0.312216973	0.022091046	Mpst_Tg_upregulation
NM_001291282	*Tm6sf1*	0.311499745	0.026645606	Mpst_Tg_upregulation
NM_008553	*Ascl1*	0.309978235	0.039723829	Mpst_Tg_upregulation
NM_001009948	*Nrsn2*	0.308024188	0.02940404	Mpst_Tg_upregulation
NM_009573	*Zic1*	0.306138673	0.038540147	Mpst_Tg_upregulation
NM_013534	*P3h3*	0.305262945	0.022987868	Mpst_Tg_upregulation

NM_172661	*Prrc2b*	0.304231071	0.015850547	Mpst_Tg_upregulation
NM_001256142	*Fsbp*	0.302856984	0.026065384	Mpst_Tg_upregulation
NM_023543	*Chn2*	0.299838432	0.021891263	Mpst_Tg_upregulation
NM_001290465	*Adamts13*	0.299166498	0.045381832	Mpst_Tg_upregulation
NM_010127	*Pou6f1*	0.298931119	0.001860827	Mpst_Tg_upregulation
NM_016701	*Nes*	0.297709199	0.007744428	Mpst_Tg_upregulation
NM_175423	*Orai1*	0.297009157	0.020599081	Mpst_Tg_upregulation
NM_175308	*Mob3c*	0.296765779	0.017137149	Mpst_Tg_upregulation
NM_028966	*Samd4*	0.29463294	0.049175682	Mpst_Tg_upregulation
NR_033324	*BC065397*	0.292141479	0.010963586	Mpst_Tg_upregulation
NM_001293668	*Atp11a*	0.292062775	0.04708436	Mpst_Tg_upregulation
NM_019923	*Itpr2*	0.291318538	0.049921415	Mpst_Tg_upregulation
NM_011797	*Car14*	0.290988641	0.041552319	Mpst_Tg_upregulation
NM_053269	*Rad51c*	0.288424617	0.022229418	Mpst_Tg_upregulation
NM_001271860	*Add2*	0.288133393	0.032111989	Mpst_Tg_upregulation
NM_009384	*Tiam1*	0.287445918	0.015048547	Mpst_Tg_upregulation
NM_175650	*Atp13a5*	0.287320187	0.004243898	Mpst_Tg_upregulation
NM_153197	*Clec4a3*	0.283501064	0.04036594	Mpst_Tg_upregulation
NM_007652	*Cd59a*	0.28096166	0.005597167	Mpst_Tg_upregulation
NM_146257	*Slc29a4*	0.280358995	0.037908494	Mpst_Tg_upregulation
NM_198937	*Hn1l*	0.28016672	0.042934354	Mpst_Tg_upregulation
NM_001001333	*Hexdc*	0.279663769	0.042879343	Mpst_Tg_upregulation
NM_001278534	*Rgag4*	0.276327296	0.015180811	Mpst_Tg_upregulation
NM_147218	*Abca6*	0.27447077	0.026303756	Mpst_Tg_upregulation
NM_175003	*Arhgap44*	0.274184978	0.036547061	Mpst_Tg_upregulation
NM_001081005	*1500012F01Rik*	0.273179703	0.019679059	Mpst_Tg_upregulation
NM_198090	*Hnrnpa3*	0.272900667	0.044660248	Mpst_Tg_upregulation
NM_178598	*Tagln2*	0.270240147	0.021111695	Mpst_Tg_upregulation
NR_037906	*Luc7l*	0.269463392	0.001533966	Mpst_Tg_upregulation
NR_015505	*Firre*	0.268935029	0.006941007	Mpst_Tg_upregulation
NM_172126	*Adam1a*	0.268838756	0.041163853	Mpst_Tg_upregulation
NM_175162	*Stox2*	0.268442547	0.002804948	Mpst_Tg_upregulation
NM_020259	*Hhip*	0.26679888	0.018524638	Mpst_Tg_upregulation
NM_009285	*Stc1*	0.266247302	0.041656694	Mpst_Tg_upregulation
NM_053196	*Sfxn2*	0.266240773	0.036358397	Mpst_Tg_upregulation
NR_015566	*A330023F24Rik*	0.265238054	0.013008732	Mpst_Tg_upregulation
NM_019696	*Cpxm1*	0.263094828	0.020471158	Mpst_Tg_upregulation
NM_001113325	*Gria1*	0.262664736	0.023690586	Mpst_Tg_upregulation
NR_033621	*Olfr856-ps1*	0.26233822	0.034082681	Mpst_Tg_upregulation
NM_028894	*Lonrf3*	0.259114099	0.007725141	Mpst_Tg_upregulation
NM_199475	*Fam63a*	0.258446965	0.028159184	Mpst_Tg_upregulation
NM_008239	*Foxq1*	0.254527876	0.048535186	Mpst_Tg_upregulation
NM_001009947	*Dock11*	0.254407307	0.025406862	Mpst_Tg_upregulation
NM_001082484	*Snx27*	0.254379755	0.003765828	Mpst_Tg_upregulation
NM_175692	*Snhg11*	0.254057637	0.025268357	Mpst_Tg_upregulation
NM_199079	*Ddx17*	0.253595361	0.017100947	Mpst_Tg_upregulation
NM_001081329	*Zkscan2*	0.252948579	0.026696879	Mpst_Tg_upregulation
NM_153545	*Lrrc45*	0.252790369	0.027361125	Mpst_Tg_upregulation
NM_001110506	*Efcab12*	0.25193168	0.031646949	Mpst_Tg_upregulation
NM_001033380	*Itpripl2*	0.251928288	0.035876881	Mpst_Tg_upregulation
NM_153596	*Tmem17*	0.251750875	0.020854242	Mpst_Tg_upregulation
NM_018851	*Samhd1*	0.25078153	0.025860133	Mpst_Tg_upregulation
NM_178673	*Fstl5*	0.248694028	0.042200659	Mpst_Tg_upregulation
NR_028380	*Ftx*	0.248568989	0.013630226	Mpst_Tg_upregulation
NM_021720	*Donson*	0.248492135	0.016360472	Mpst_Tg_upregulation
NM_181413	*Anks1*	0.247265519	0.019977941	Mpst_Tg_upregulation
NM_019832	*Gkap1*	0.247199293	0.043673219	Mpst_Tg_upregulation
NR_036616	*Srsf9*	0.246415714	0.030642423	Mpst_Tg_upregulation
NR_040398	*4930570G19Rik*	0.246243039	0.029466016	Mpst_Tg_upregulation
NR_026990	*Gm5577*	0.244560379	0.045271585	Mpst_Tg_upregulation
NM_011157	*Srgn*	0.242257257	0.047637973	Mpst_Tg_upregulation
NR_033221	*BC031361*	0.241798458	0.037280206	Mpst_Tg_upregulation

NR_027651	*Meg3*	0.24081407	0.023571381	Mpst_Tg_upregulation
NM_172471	*Itih5*	0.239389491	0.034425598	Mpst_Tg_upregulation
NM_001290379	*Ap1s2*	0.239173206	0.049647197	Mpst_Tg_upregulation
NR_033121	*Gm10409*	0.236348427	0.041086173	Mpst_Tg_upregulation
NM_001163728	*4930563E22Rik*	0.235591327	0.043303368	Mpst_Tg_upregulation
NR_033121	*Gm10409*	0.235168465	0.036091452	Mpst_Tg_upregulation
NM_016967	*Olig2*	0.234074395	0.012623005	Mpst_Tg_upregulation
NM_023047	*Dpysl5*	0.233817484	0.007554349	Mpst_Tg_upregulation
NM_001035510	*Zcchc18*	0.233380035	0.012281283	Mpst_Tg_upregulation
NM_178066	*Tmem198b*	0.231622008	0.03348749	Mpst_Tg_upregulation
NM_146057	*Dap*	0.230620517	0.028683312	Mpst_Tg_upregulation
NM_001276279	*Cyld*	0.230300283	0.034077348	Mpst_Tg_upregulation
NM_012018	*Cntrl*	0.228598003	0.040854783	Mpst_Tg_upregulation
NM_022980	*Rcan3*	0.228282613	0.028006206	Mpst_Tg_upregulation
NM_017401	*Polm*	0.228275507	0.03176932	Mpst_Tg_upregulation
NM_001081395	*Amotl1*	0.227780702	0.028591008	Mpst_Tg_upregulation
NM_178774	*Prr18*	0.227322198	0.001516113	Mpst_Tg_upregulation
NM_001110271	*Abhd14a*	0.226954919	0.029532757	Mpst_Tg_upregulation
NM_007616	*Cav1*	0.226946979	0.010960981	Mpst_Tg_upregulation
NM_177074	*Slc38a11*	0.224407612	0.045136805	Mpst_Tg_upregulation
NM_001025381	*Gpr17*	0.224268579	0.013837185	Mpst_Tg_upregulation
NM_027890	*Susd2*	0.223892885	0.014106489	Mpst_Tg_upregulation
NM_007801	*Ctsh*	0.222953081	0.015041648	Mpst_Tg_upregulation
NM_009621	*Adamts1*	0.222363422	0.049717089	Mpst_Tg_upregulation
NM_177185	*Ubn2*	0.222027461	0.012459157	Mpst_Tg_upregulation
NM_133188	*Dazap1*	0.221913651	0.020858163	Mpst_Tg_upregulation
NM_010252	*Gabrg1*	0.220775591	0.034037743	Mpst_Tg_upregulation
NM_001159301	*Lgals9*	0.220757662	0.013056604	Mpst_Tg_upregulation
NM_172530	*She*	0.220149186	0.049408815	Mpst_Tg_upregulation
NM_019427	*Epb4.1l4b*	0.219613215	0.00986834	Mpst_Tg_upregulation
NM_001199352	*Pnck*	0.219407557	0.011489599	Mpst_Tg_upregulation
NM_001081386	*Cdh19*	0.218344137	0.033700527	Mpst_Tg_upregulation
NR_028429	*Thap6*	0.218334601	0.005491868	Mpst_Tg_upregulation
NM_010656	*Sspn*	0.218185116	0.034174881	Mpst_Tg_upregulation
NM_177135	*D830030K20Rik*	0.216735244	0.048805109	Mpst_Tg_upregulation
NM_008832	*Phka1*	0.216715357	0.020434467	Mpst_Tg_upregulation
NM_001293559	*Cox4i1*	0.215927422	0.032669087	Mpst_Tg_upregulation
NM_207655	*Egfr*	0.215891832	0.012021199	Mpst_Tg_upregulation
NM_178281	*Trim39*	0.215690168	0.024888891	Mpst_Tg_upregulation
NM_177710	*Ssh2*	0.213771293	0.004091161	Mpst_Tg_upregulation
NM_153507	*Cpne2*	0.21316154	0.039859903	Mpst_Tg_upregulation
NM_001001806	*Zfp36l2*	0.212622511	0.012270435	Mpst_Tg_upregulation
NM_031184	*Glis2*	0.211965421	0.009563405	Mpst_Tg_upregulation
NM_173012	*Letm2*	0.211873602	0.046272871	Mpst_Tg_upregulation
NM_146065	*Atf7*	0.210769962	0.045760083	Mpst_Tg_upregulation
NM_008830	*Abcb4*	0.210455752	0.035839308	Mpst_Tg_upregulation
NM_008026	*Fli1*	0.208691538	0.011671752	Mpst_Tg_upregulation
NM_001014974	*Ttll4*	0.207961467	0.00762626	Mpst_Tg_upregulation
NM_001159553	*H13*	0.206584923	0.020550744	Mpst_Tg_upregulation
NM_019422	*Elovl1*	0.206261343	0.044071618	Mpst_Tg_upregulation
NM_021390	*Sall1*	0.204937717	0.028736574	Mpst_Tg_upregulation
NM_172833	*Malt1*	0.204288417	0.02270605	Mpst_Tg_upregulation
NM_001099785	*D3Ertd751e*	0.203580084	0.041894324	Mpst_Tg_upregulation
NM_029098	*Lmbr1l*	0.201576844	0.005251497	Mpst_Tg_upregulation
NR_027351	*Trpt1*	0.201042097	0.038798922	Mpst_Tg_upregulation
NM_177364	*Sh3pxd2b*	0.200973008	0.041252064	Mpst_Tg_upregulation
NM_028749	*Npl*	0.200658959	0.011736048	Mpst_Tg_upregulation
NM_008156	*Gpld1*	0.200600124	0.00592344	Mpst_Tg_upregulation
NM_007564	*Zfp36l1*	0.20047138	0.036034498	Mpst_Tg_upregulation
NM_178704	*Dpy19l3*	0.199379044	0.047966241	Mpst_Tg_upregulation
NM_001081049	*Kmt2a*	0.199159476	0.017357553	Mpst_Tg_upregulation
NM_019802	*Ggcx*	0.199102128	0.017138681	Mpst_Tg_upregulation

NM_176837	*Arhgap18*	0.195331124	0.009035804	Mpst_Tg_upregulation
NM_007831	*Dcc*	0.193943566	0.026389056	Mpst_Tg_upregulation
NM_134257	*Rgs3*	0.19389993	0.008234924	Mpst_Tg_upregulation
NM_001198861	*Ctbp1*	0.193605404	0.043907061	Mpst_Tg_upregulation
NM_013864	*Ndrg2*	0.193103694	0.002070217	Mpst_Tg_upregulation
NM_194262	*Arid4b*	0.192922163	0.015243592	Mpst_Tg_upregulation
NM_001164805	*Thsd7a*	0.192821375	0.027573324	Mpst_Tg_upregulation
NM_008043	*Frat1*	0.192393224	0.015322583	Mpst_Tg_upregulation
NM_011976	*Sema4g*	0.191687842	0.02031498	Mpst_Tg_upregulation
NM_175229	*Srrm2*	0.191581589	0.023325164	Mpst_Tg_upregulation
NM_026493	*Cspp1*	0.191430559	0.044423665	Mpst_Tg_upregulation
NM_138590	*Zcchc7*	0.191062569	0.018718043	Mpst_Tg_upregulation
NM_001163184	*Plekhb1*	0.190560618	0.046296404	Mpst_Tg_upregulation
NM_009987	*Cx3cr1*	0.190032669	0.003507687	Mpst_Tg_upregulation
NM_019653	*Wsb1*	0.189876264	0.002356652	Mpst_Tg_upregulation
NM_001015681	*E130308A19Rik*	0.189375761	0.028162763	Mpst_Tg_upregulation
NM_175500	*Gpc5*	0.18880903	0.00069076	Mpst_Tg_upregulation
NM_013822	*Jag1*	0.188512892	0.042435033	Mpst_Tg_upregulation
NR_015483	*2610203C20Rik*	0.188165422	0.013630284	Mpst_Tg_upregulation
NM_001164659	*Trank1*	0.187679336	0.04144003	Mpst_Tg_upregulation
NM_027911	*Raver1*	0.187624841	0.024313897	Mpst_Tg_upregulation
NM_026599	*Cgnl1*	0.187538792	0.046203693	Mpst_Tg_upregulation
NM_001042488	*Dlgap4*	0.186683504	0.033792037	Mpst_Tg_upregulation
NM_026769	*Caly*	0.186581954	0.049652988	Mpst_Tg_upregulation
NM_011159	*Prkdc*	0.186076135	0.016202385	Mpst_Tg_upregulation
NM_080437	*Celsr3*	0.18451127	0.02704741	Mpst_Tg_upregulation
NM_001044740	*Slc7a2*	0.18348095	0.036571032	Mpst_Tg_upregulation
NM_011808	*Ets1*	0.183342952	0.012516299	Mpst_Tg_upregulation
NM_029836	*Tspyl2*	0.182573399	0.013695036	Mpst_Tg_upregulation
NM_177572	*Rimkla*	0.182443449	0.041991422	Mpst_Tg_upregulation
NM_029385	*Nudt16*	0.181806126	0.005539525	Mpst_Tg_upregulation
NM_130879	*Usp48*	0.181206503	0.010491611	Mpst_Tg_upregulation
NM_013780	*Npas3*	0.181011692	0.041824917	Mpst_Tg_upregulation
NM_172779	*Ddx26b*	0.181009955	0.029410504	Mpst_Tg_upregulation
NR_033388	*Gm3002*	0.180241981	0.03394836	Mpst_Tg_upregulation
NM_177047	*Auts2*	0.17948183	0.039242597	Mpst_Tg_upregulation
NM_145940	*Wipi1*	0.179310781	0.025331836	Mpst_Tg_upregulation
NM_009847	*Cd2ap*	0.178634173	0.003807389	Mpst_Tg_upregulation
NM_033602	*Peli2*	0.17818127	0.005004259	Mpst_Tg_upregulation
NM_028234	*Rbm33*	0.17782931	0.016707989	Mpst_Tg_upregulation
NM_134115	*Stk38*	0.177386277	0.000221556	Mpst_Tg_upregulation
NM_153145	*Abca8a*	0.177187178	0.025661758	Mpst_Tg_upregulation
NM_001252530	*Slco2b1*	0.176754154	0.005185519	Mpst_Tg_upregulation
NM_145925	*Pttg1ip*	0.176668712	0.002714629	Mpst_Tg_upregulation
NM_001097644	*Ccnyl1*	0.176436765	0.015466849	Mpst_Tg_upregulation
NM_001011780	*Olfr287*	0.175996741	0.035652358	Mpst_Tg_upregulation
NM_001042707	*Ilf3*	0.175481125	0.021745905	Mpst_Tg_upregulation
NM_138749	*Plxnb2*	0.175307891	0.026380543	Mpst_Tg_upregulation
NM_001244916	*Sall2*	0.175075626	0.031518075	Mpst_Tg_upregulation
NM_019922	*Crtap*	0.174631932	0.020825803	Mpst_Tg_upregulation
NM_175367	*Ston2*	0.173139409	0.010498601	Mpst_Tg_upregulation
NM_023908	*Slco3a1*	0.172576747	0.046211437	Mpst_Tg_upregulation
NM_009071	*Rock1*	0.172517052	0.008004246	Mpst_Tg_upregulation
NM_016721	*Iqgap1*	0.172069659	0.017224768	Mpst_Tg_upregulation
NM_001033286	*Slc30a10*	0.171249739	0.036645368	Mpst_Tg_upregulation
NM_023372	*Rpl38*	0.171085334	0.034463524	Mpst_Tg_upregulation
NM_018732	*Scn3a*	0.17019378	0.022275243	Mpst_Tg_upregulation
NR_033146	*Gm17821*	0.17014145	0.021189558	Mpst_Tg_upregulation
NM_175155	*Sash1*	0.169799119	0.012845396	Mpst_Tg_upregulation
NM_001035123	*Setd6*	0.169383058	0.035418082	Mpst_Tg_upregulation
NM_134003	*Zc3h10*	0.168564368	0.047395604	Mpst_Tg_upregulation
NM_177715	*Kctd12*	0.168470888	0.007746605	Mpst_Tg_upregulation

NM_030147	*Brd8*	0.167491125	0.014942244	Mpst_Tg_upregulation
NM_001013770	*Lipo1*	0.165690629	0.044039974	Mpst_Tg_upregulation
NM_009655	*Alcam*	0.164999456	0.0243589	Mpst_Tg_upregulation
NM_001081391	*Csmd3*	0.164488134	0.015687279	Mpst_Tg_upregulation
NM_010821	*Mpeg1*	0.163539966	0.024272019	Mpst_Tg_upregulation
NM_010918	*Nktr*	0.163311292	0.025610624	Mpst_Tg_upregulation
NM_013659	*Sema4b*	0.162491973	0.042962981	Mpst_Tg_upregulation
NM_026418	*Rgs10*	0.162201256	0.029358903	Mpst_Tg_upregulation
NM_016785	*Tpmt*	0.16095777	0.039253771	Mpst_Tg_upregulation
NM_009980	*Ctbp2*	0.160957574	0.028326775	Mpst_Tg_upregulation
NM_145537	*Edem2*	0.160721566	0.014392219	Mpst_Tg_upregulation
NM_008876	*Pld2*	0.160679528	0.040676071	Mpst_Tg_upregulation
NM_146114	*Dclre1c*	0.160206075	0.021199744	Mpst_Tg_upregulation
NM_028341	*Ttc39c*	0.160153228	0.036845883	Mpst_Tg_upregulation
NM_173788	*Npr2*	0.16003717	0.035904943	Mpst_Tg_upregulation
NM_009615	*Adam17*	0.159849418	0.049439212	Mpst_Tg_upregulation
NM_029781	*Rab36*	0.159824223	0.041271675	Mpst_Tg_upregulation
NM_011261	*Reln*	0.158710069	0.000572378	Mpst_Tg_upregulation
NM_001111268	*Grik2*	0.157815489	0.013338126	Mpst_Tg_upregulation
NM_015828	*Gne*	0.157445513	0.026934243	Mpst_Tg_upregulation
NM_026046	*Zfp329*	0.157367728	0.007751743	Mpst_Tg_upregulation
NR_028101	*Gm12191*	0.156947562	0.047369946	Mpst_Tg_upregulation
NM_016799	*Srrm1*	0.155361372	0.013589006	Mpst_Tg_upregulation
NM_009230	*Soat1*	0.155349974	0.002617993	Mpst_Tg_upregulation
NM_026036	*Cmtm6*	0.155261082	0.024169617	Mpst_Tg_upregulation
NM_025669	*Pnisr*	0.153254145	0.023497513	Mpst_Tg_upregulation
NM_001159516	*Qk*	0.153093947	0.015774522	Mpst_Tg_upregulation
NM_026963	*Lzic*	0.151015617	0.048696748	Mpst_Tg_upregulation
NM_009832	*Ccnk*	0.150560798	0.014336132	Mpst_Tg_upregulation
NM_145955	*Mcmbp*	0.147923587	0.022051144	Mpst_Tg_upregulation
NM_026825	*Lrrc16a*	0.14785945	0.01458372	Mpst_Tg_upregulation
NM_027189	*Gemin7*	0.146544315	0.046101907	Mpst_Tg_upregulation
NM_001162906	*2410089E03Rik*	0.146431432	0.006216197	Mpst_Tg_upregulation
NM_001033472	*A830080D01Rik*	0.146217357	0.049104418	Mpst_Tg_upregulation
NM_028099	*Dusp11*	0.145937029	0.022292086	Mpst_Tg_upregulation
NM_207636	*Fndc3a*	0.14588978	0.003093978	Mpst_Tg_upregulation
NM_011594	*Timp2*	0.145845082	0.02480913	Mpst_Tg_upregulation
NM_172907	*Olfml1*	0.145035243	0.037249563	Mpst_Tg_upregulation
NM_026069	*Rpl37*	0.144095234	0.0494944	Mpst_Tg_upregulation
NM_028493	*Rhobtb3*	0.143869734	0.000881148	Mpst_Tg_upregulation
NM_001166552	*Zbed6*	0.143441724	0.036054423	Mpst_Tg_upregulation
NM_001024928	*Zfp667*	0.142551418	0.0377079	Mpst_Tg_upregulation
NM_145825	*Cetn4*	0.141778497	0.025892985	Mpst_Tg_upregulation
NM_028808	*P2ry13*	0.141749674	0.027645189	Mpst_Tg_upregulation
NM_023644	*Mccc1*	0.141731645	0.003175713	Mpst_Tg_upregulation
NM_001110253	*Fyco1*	0.139981037	0.046218957	Mpst_Tg_upregulation
NM_011247	*Rbbp6*	0.139971296	0.003179695	Mpst_Tg_upregulation
NM_028708	*Jakmip3*	0.139706283	0.015821464	Mpst_Tg_upregulation
NM_001039546	*Myo6*	0.138997494	0.015267282	Mpst_Tg_upregulation
NM_025845	*Prpf38b*	0.138248433	0.008818063	Mpst_Tg_upregulation
NM_013595	*Mbd3*	0.137912753	0.018788883	Mpst_Tg_upregulation
NM_028932	*Eaf1*	0.137596877	0.044372611	Mpst_Tg_upregulation
NM_009163	*Sgpl1*	0.137409086	0.041996253	Mpst_Tg_upregulation
NM_133710	*Ctdspl*	0.13715213	0.00243809	Mpst_Tg_upregulation
NM_008133	*Glud1*	0.13687156	0.013569923	Mpst_Tg_upregulation
NM_133208	*Zfp287*	0.136717267	0.031731829	Mpst_Tg_upregulation
NM_021315	*Noc3l*	0.13621062	0.010128196	Mpst_Tg_upregulation
NM_001005385	*Gprasp1*	0.135821328	0.004424213	Mpst_Tg_upregulation
NM_198100	*Tbkbp1*	0.135609616	0.019305994	Mpst_Tg_upregulation
NM_153319	*Amot*	0.133103636	0.03335832	Mpst_Tg_upregulation
NM_172851	*Cntnap5b*	0.132522126	0.033742894	Mpst_Tg_upregulation
NM_130796	*Snx18*	0.132022302	0.039880828	Mpst_Tg_upregulation

NM_013725	*Rps11*	0.131966441	1.93218E-05	Mpst_Tg_upregulation
NM_011602	*Tln1*	0.131708563	0.041506907	Mpst_Tg_upregulation
NM_025943	*Dzip1*	0.131359023	0.011892704	Mpst_Tg_upregulation
NR_030694	*1110002L01Rik*	0.130443637	0.011546089	Mpst_Tg_upregulation
NM_008745	*Ntrk2*	0.13043048	0.008236113	Mpst_Tg_upregulation
NM_173740	*Maoa*	0.129477379	0.047361082	Mpst_Tg_upregulation
NM_018786	*Prpf40b*	0.129260047	0.029943747	Mpst_Tg_upregulation
NM_001278256	*Prnp*	0.128485964	0.029240668	Mpst_Tg_upregulation
NM_080708	*Bmp2k*	0.127937876	0.039487246	Mpst_Tg_upregulation
NM_001289771	*Exoc1*	0.127272891	0.048387781	Mpst_Tg_upregulation
NM_001276704	*Nxf1*	0.127044228	0.013208744	Mpst_Tg_upregulation
NM_172671	*Lgr4*	0.12389002	0.047616638	Mpst_Tg_upregulation
NM_009538	*Plagl1*	0.123846119	0.017851313	Mpst_Tg_upregulation
NM_001163610	*Nhsl2*	0.121512092	0.048248238	Mpst_Tg_upregulation
NM_172757	*Heatr3*	0.120710308	0.038292655	Mpst_Tg_upregulation
NM_177093	*Lrrc58*	0.116300286	0.016936322	Mpst_Tg_upregulation
NM_011546	*Zeb1*	0.11415181	0.048896434	Mpst_Tg_upregulation
NM_020608	*Cramp1l*	0.113967653	0.014675314	Mpst_Tg_upregulation
NM_172592	*Srek1*	0.113861266	0.030264954	Mpst_Tg_upregulation
NM_029432	*4930402H24Rik*	0.113802423	0.00544976	Mpst_Tg_upregulation
NM_010008	*Cyp2j6*	0.112398207	0.028646235	Mpst_Tg_upregulation
NM_011952	*Mapk3*	0.111093219	0.022246408	Mpst_Tg_upregulation
NM_144925	*Tnrc6a*	0.110894481	0.018615139	Mpst_Tg_upregulation
NM_184088	*Dennd4c*	0.110701863	0.000963883	Mpst_Tg_upregulation
NM_001024952	*Rc3h1*	0.110404908	0.016921983	Mpst_Tg_upregulation
NM_001191004	*Lsm6*	0.109862971	0.007288084	Mpst_Tg_upregulation
NM_001081383	*Kmt2c*	0.109295378	0.029881837	Mpst_Tg_upregulation
NM_183225	*Usp24*	0.108790806	0.010166506	Mpst_Tg_upregulation
NM_172924	*Peak1*	0.107973426	0.02700555	Mpst_Tg_upregulation
NM_001081269	*Whsc1l1*	0.107807458	0.016797396	Mpst_Tg_upregulation
NM_198105	*Fam120c*	0.107283997	0.038932311	Mpst_Tg_upregulation
NM_008304	*Sdc2*	0.106503405	0.044299789	Mpst_Tg_upregulation
NM_011993	*Dpysl4*	0.106187419	0.01413415	Mpst_Tg_upregulation
NM_183186	*Foxn3*	0.105818485	0.030471424	Mpst_Tg_upregulation
NM_001111015	*Syn2*	0.105356485	0.039328635	Mpst_Tg_upregulation
NM_027184	*Ipmk*	0.105187454	0.047927539	Mpst_Tg_upregulation
NM_007839	*Dhx15*	0.104568458	0.020611037	Mpst_Tg_upregulation
NM_011840	*Map2k5*	0.103739092	0.023164972	Mpst_Tg_upregulation
NM_001114399	*Zmym4*	0.102152118	0.037595264	Mpst_Tg_upregulation
NM_001081225	*Fam178a*	0.101906661	0.041923766	Mpst_Tg_upregulation
NM_178686	*Cep120*	0.101671324	0.010994453	Mpst_Tg_upregulation
NM_008891	*Pnn*	0.099712809	0.041446495	Mpst_Tg_upregulation
NM_008514	*Lrp6*	0.098793445	0.002462072	Mpst_Tg_upregulation
NM_020577	*As3mt*	0.098156197	0.018514747	Mpst_Tg_upregulation
NM_001287164	*Nup98*	0.09787947	0.016269305	Mpst_Tg_upregulation
NM_001289895	*Tns1*	0.097496584	0.035554333	Mpst_Tg_upregulation
NM_024200	*Mfn1*	0.097166871	0.028653334	Mpst_Tg_upregulation
NM_001002239	*Rpl17*	0.095957441	0.03106892	Mpst_Tg_upregulation
NM_172861	*Slc7a14*	0.095945615	0.036902682	Mpst_Tg_upregulation
NM_011992	*Rcn2*	0.095103998	0.020871459	Mpst_Tg_upregulation
NM_028643	*Micu2*	0.094989597	0.009070543	Mpst_Tg_upregulation
NM_178880	*Son*	0.094428253	0.003497809	Mpst_Tg_upregulation
NM_010833	*Msn*	0.093799098	0.020072687	Mpst_Tg_upregulation
NM_172409	*Fmnl2*	0.093340523	0.044386524	Mpst_Tg_upregulation
NM_008943	*Psen1*	0.092272985	0.047971857	Mpst_Tg_upregulation
NM_001033463	*Tatdn2*	0.092163264	0.029395861	Mpst_Tg_upregulation
NM_025481	*Smurf2*	0.091721329	0.042637911	Mpst_Tg_upregulation
NM_201609	*Zfp652*	0.09171575	0.020021386	Mpst_Tg_upregulation
NM_001081154	*Marf1*	0.091572363	0.001848743	Mpst_Tg_upregulation
NM_198304	*Nup188*	0.08865017	0.007600754	Mpst_Tg_upregulation
NM_177301	*Hnrnpl*	0.086948237	0.048580908	Mpst_Tg_upregulation
NM_001301643	*Pitpnb*	0.084897997	0.011889065	Mpst_Tg_upregulation

NM_001039184	*Cep350*	0.084049132	0.019462	Mpst_Tg_upregulation
NM_054043	*Msi2*	0.083640112	0.033846741	Mpst_Tg_upregulation
NM_001081149	*Kat6a*	0.081050949	0.024866657	Mpst_Tg_upregulation
NM_011508	*Eif1*	0.080327472	0.0448991	Mpst_Tg_upregulation
NM_148930	*Rbm5*	0.078794425	0.045056276	Mpst_Tg_upregulation
NM_010568	*Insr*	0.078566758	0.029379726	Mpst_Tg_upregulation
NM_146122	*Dennd1a*	0.078498225	0.026498043	Mpst_Tg_upregulation
NM_009128	*Scd2*	0.077051882	0.028704311	Mpst_Tg_upregulation
NM_001164199	*Prkacb*	0.076157807	0.011678876	Mpst_Tg_upregulation
NM_177324	*Sbf2*	0.074778188	0.036554384	Mpst_Tg_upregulation
NM_011032	*P4hb*	0.073640522	0.020279042	Mpst_Tg_upregulation
NM_007896	*Mapre1*	0.072175064	0.006746147	Mpst_Tg_upregulation
NM_008306	*Ndst1*	0.070951794	0.041731728	Mpst_Tg_upregulation
NM_001081221	*Ercc6*	0.069757451	0.032996899	Mpst_Tg_upregulation
NM_030035	*Golgb1*	0.069380169	0.002616463	Mpst_Tg_upregulation
NM_009007	*Rac1*	0.06908682	0.02118356	Mpst_Tg_upregulation
NM_146019	*Chd3*	0.067268571	0.045724469	Mpst_Tg_upregulation
NM_011192	*Psme3*	0.066485857	0.02291172	Mpst_Tg_upregulation
NM_029868	*Gpbp1l1*	0.064729213	0.018338796	Mpst_Tg_upregulation
NM_027558	*Pgrmc2*	0.062068525	0.029438401	Mpst_Tg_upregulation
NM_001081293	*Rprd2*	0.055019673	0.032220024	Mpst_Tg_upregulation
NM_024473	*BC005537*	0.051199764	0.024342112	Mpst_Tg_upregulation
NM_007479	*Arf4*	0.048931637	0.047294104	Mpst_Tg_upregulation
NM_153585	*Cnot10*	0.038768847	0.045137754	Mpst_Tg_upregulation

Appendix Table S14. Canonical pathways enriched for dysregulated genes in the frontal cortex of *Mpst* KO and *Mpst* Tg mice.

Group	Ingenuity Canonical Pathways	-log (P-value)	Molecules
Mpst KO (downregulated)	Acyl-CoA Hydrolysis	2.23	PPT1, ACOT8
	Hypoxia Signaling in the Cardiovascular System	2.19	UBE2W, UBE2J2, UBE2I, ATF2
	L-cysteine Degradation III	1.71	MPST
	Thiosulfate Disproportionation III (Rhodanese)	1.53	MPST
	Role of BRCA1 in DNA Damage Response	1.35	FAAP24, ATR, MLH1
	Lysine Degradation	1.32	AASDH
Mpst KO (upregulated)	Aldosterone Signaling in Epithelial Cells	2.11	CRYAB, ASIC2, PIP5K1C, PIK3R1, DNAJA1, PLCD4
	Ephrin A Signaling	1.63	FYN, PIK3R1, EFNA3
	Role of NFAT in Cardiac Hypertrophy	1.59	HDAC4, CAMK1D, PIK3R1, MEF2D, SLC8A2, PLCD4
	NADH Repair	1.52	NAXD
	D-myo-inositol (1,4,5)-Trisphosphate Biosynthesis	1.51	PIP5K1C, PLCD4
	Rac Signaling	1.49	CYFIP2, PIP5K1C, PIK3R1, ANK1
	PI3K Signaling in B Lymphocytes	1.35	FYN, PIK3R1, FCGR2B, PLCD4
	14-3-3-mediated Signaling	1.34	PIK3R1, PLCD4, SNCA, AKT1S1
	Inhibition of Angiogenesis by TSP1	1.33	FYN, GUCY1B3
	Trehalose Degradation II (Trehalase)	1.3	HK1
	Protein Citrullination	1.3	PADI2
	Galactose Degradation I (Leloir Pathway)	1.3	GALK2

Group	Ingenuity Canonical Pathways	-log (P-value)	Molecules
Mpst Tg (downregulated)	Phagosome Maturation	4.32	ATP6V0E2, ATP6V0C, VTI1A, ATP6V1C1, PRDX5, TUBA4A, ATP6V1A, SNAP25, DYNC1LI2, ATP6V1H, ATP6V0D1, ATP6V1G2, NAPA, ATP6V1B2
	Glycolysis I	4.27	PGK1, ENO1, TPI1, PFKL, PFKP, PFKM
	TCA Cycle II (Eukaryotic)	3.41	SUCLA2, SDHB, IDH3G, MDH1, IDH3B
	Dopamine-DARPP32 Feedback in cAMP Signaling	3.29	ADCY2, Calm1 (includes others), CSNK1G2, KCNJ9, ADCY1, CAMKK1, PRKAR1B, GNAI1, KCNJ3, PPP2R2C, KCNJ6, CAMKK2, CALY
	Cardiac Hypertrophy Signaling	3.15	MAP2K4, ADCY2, Calm1 (includes others), PIK3R1, GNAI1, ATF6, GNG3, ADCY1, PRKAR1B, MAPK10, EIF2B1, MYL4, MEF2C, MAPKAPK2, FNBP1, ADRA1A
	Parkinson's Signaling	3.14	SEPT5, PARK7, CYCS, SNCA
	Mitochondrial Dysfunction	3.12	MAP2K4, SDHB, NDUFA9, PRDX5, PDHA1, PARK7, GPD2, TXN2, NDUFA6, MAPK10, NDUFB6, CYCS, SNCA
	IL-1 Signaling	3.08	MAP2K4, IKBKB, ADCY2, TOLLIP, ADCY1, PRKAR1B, MAPK10, GNAI1, GNG3
	G-Protein Coupled Receptor Signaling	2.86	ADCY2, GRM2, GRM3, PIK3R1, SSTR3, GNAI1, PDE1A, CHRM3, CHRM1, IKBKB, GABBR2, GDE1, ADCY1, PRKAR1B, HTR1F, ADRA1A, HTR2A
	G α i Signaling	2.82	GABBR2, ADCY2, GRM2, GRM3, SSTR3, ADCY1, PRKAR1B, GNAI1, HTR1F, GNG3
	AMPK Signaling	2.65	CHRNA4, PIK3R1, PFKL, PFKP, CHRM3, CHRM1, PFKM, AK5, PRKAR1B, AK4, PPP2R2C, HMGCR, ADRA1A, CAMKK2
	cAMP-mediated signaling	2.45	GABBR2, ADCY2, GRM2, Calm1 (includes others), GRM3, GDE1, ADCY1, SSTR3, PRKAR1B, GNAI1, HTR1F, PDE1A, CHRM1, CHRM3
	G Beta Gamma Signaling	2.43	ADCY2, PAK1, KCNJ9, ADCY1, PRKAR1B, GNAI1, KCNJ3, KCNJ6, GNG3
	Calcium Signaling	2.4	CHRNA4, Calm1 (includes others), TNNC1, HDAC7, CAMKK1, PRKAR1B, Tpm1, MYL4, MEF2C, TRPC6, GRIA4, CAMKK2, GRIA3
	PRPP Biosynthesis I	2.38	PRPS2, PRPS1
	Acetate Conversion to Acetyl-CoA	2.38	ACSS2, ACSL1
	Glutamate Receptor Signaling	2.38	GRM2, Calm1 (includes others), GRM3, SLC17A7, GRIA4, GRIA3
	CREB Signaling in Neurons	2.3	POLR2G, ADCY2, GRM2, Calm1 (includes others), GRM3, PIK3R1, ADCY1, PRKAR1B, POLR2J, GNAI1, GNG3, GRIA4, GRIA3
	Sirtuin Signaling Pathway	2.23	PGK1, SDHB, SLC25A4, NDUFA9, TUBA4A, BECN1, PFKM, PDHA1, ATG4C, ATG9A, NDUFA6, ACSS2, CLOCK, NDUFB6, LDHA, KAT2A
	Huntington's Disease Signaling	2.09	MAP2K4, SDHB, VTI1A, PIK3R1, POLR2J, GNG3, STX1A, SNAP25, POLR2G, DNM1, HDAC7, CYCS, NAPA, SNCA
	α -Adrenergic Signaling	2.04	ADCY2, Calm1 (includes others), ADCY1, PRKAR1B, GNAI1, GNG3, ADRA1A

***Mpst* Tg (upregulated)**	Gap Junction Signaling	3.52	PRKACB, ITPR2, GRIA1, GRIA4, HTR2C, PLCE1, KL, MAPK3, CAV1, GRIK2, NPR2, MAP2K5, EGFR
	Neuropathic Pain Signaling in Dorsal Horn Neurons	3.09	PRKACB, KCNN3, NTRK2, PLCE1, KL, ITPR2, MAPK3, GRIA1, GRIA4
	Axonal Guidance Signaling	2.74	PRKACB, ADAM17, ADAMTS13, RGS3, ADAMTS1, RAC1, DPYSL5, HHIP, ROCK1, VEGFA, NTRK2, PLCE1, GLIS2, SDC2, KL, MAPK3, DCC, SEMA4G, PLXNB2, SEMA4B
	Choline Biosynthesis III	2.63	PLD2, GPLD1, PHKA1
	CMP-N-acetylneuraminate Biosynthesis I (Eukaryotes)	2.36	GNE, NAGK
	STAT3 Pathway	2.3	PIAS3, NTRK2, MAPK3, RAC1, INSR, EGFR
	Semaphorin Signaling in Neurons	2.27	ROCK1, MAPK3, DPYSL4, RAC1, DPYSL5

Appendix Table S15. Results of genomic quantitative PCR for *Mpst*.

Mouse strain	*Mpst* (Exon4) Mm00615042_cn [a]	*Tfrc* (Cat# 4458366) [b]	*Mpst*/*Trfc* [c]
B6 (*n* = 4)	16.281	18.107	0.899
C3H (*n* = 4)	9.617	9.933	0.968

[a] TaqMan assay I.D.

[b] Used as an internal control probe from catalogue No. of Life TechnologiesTM (Carlsbad, CA, USA)

[c] Relative quantity of PCR product

Appendix Table S16. Genetic association analyses of *MPST* and *CBS* with schizophrenia.

Gene			HWE [a]	N	Allele		*P* value (Fisher's exact Test)	Genotype			*P* value (Fisher's exact Test)	MAF [b]
MPST	Tag SNP1				T	C		T/T	T/C	C/C		
	rs5756489	SC [c]	0.1638	2011	1270	2752	0.1404	214	842	955	0.2865	31.58%
		CT [d]	0.5184	2166	1434	2898		244	946	976		33.10%
	Tag SNP2 rs2187723		Not reliable genotyped									
	Tag SNP3				A	G		A/A	A/G	G/G		
	rs5756492	SC [c]	0.0309	2011	2893	1129	0.2654	1060	773	178	0.3416	28.07%
		CT [d]	0.3834	2167	2167	1265		1095	879	193		29.19%
	Tag SNP4				A	G		A/A	A/G	G/G		
	rs5750373	SC [c]	0.0925	2008	2947	1069	0.3883	1096	755	157	0.6081	26.62%
		CT [d]	0.3029	2166	3142	1190		1149	844	173		27.47%
CBS	Tag SNP1				T	C		T/T	T/C	C/C		
	rs8132811	SC [c]	0.7493	2009	533	3485	0.2955	37	459	1513	0.4677	13.27%
		CT [d]	0.5764	2170	542	3798		31	480	1659		12.49%
	Tag SNP2				A	G		A/A	A/G	G/G		
	rs9325622	SC [c]	0.3594	2010	1932	2088	0.3239	454	1024	532	0.4609	48.06%
		CT [d]	0.0451	2167	2036	2298		455	1126	586		46.98%

Tag SNP3				T	C		T/T	T/C	C/C		
rs1789953	SC [c]	0.9039	2009	478	3540	0.5913	29	420	1560	0.8144	11.90%
	CT [d]	0.5186	2168	533	3803		36	461	1671		12.29%
Tag SNP4 rs234705	Could not be genotyped because of the existence of a neighboring SNP, rs1788466										
Tag SNP5				T	C		T/T	T/C	C/C		
rs2851391	SC [c]	0.8752	2009	1402	2616	0.4473	243	916	850	0.6549	34.89%
	CT [d]	0.3832	2169	1479	2859		243	993	933		34.09%
Tag SNP6 rs234712	Could not be genotyped because of the existence of a neighboring SNP, rs2849728										
Tag SNP7				T	G		T/T	T/G	G/G		
rs397589	SC [c]	0.8252	2008	638	3378	0.9523	52	534	1422	0.9956	15.89%
	CT [d]	0.8802	2168	691	3645		56	579	1533		15.94%
Promoter SNP1				T	C		T/T	T/C	C/C		
rs1788484	SC	0.2875	2007	968	3046	0.1791	108	752	1147	0.1473	24.12%
	CT	0.3483	2167	991	3343		121	749	1297		22.87%
Promoter SNP2				G	C		G/G	G/C	C/C		
rs2850144	SC	0.7418	2009	2577	1411	0.4098	823	931	255	0.7076	35.86%
	CT	0.7578	2166	2816	1516		912	992	262		35.00%

[a] HWE: Hardy-Weinberg equilibrium

[b] MAF: minor allele frequency

[c] SC: schizophrenia

[d] CT: control

Appendix Table S17. Primer sequences used for DNA methylation analysis and PCR product length.

Human

MPST CpG153	Primer Name	Primer Sequence	Product Length
#7	MPST_CpG153#7-L	aggaagagagTTTTTAAGGTAGAGGGGGTGG	156bp
	MPST_CpG153#7-R	cagtaatacgactcactatagggagaaggctCCTAATCCACCTAAAACAAAAAACA	
#8	MPST_CpG153#8-L	aggaagagagTTGTTTTAGGTGGATTAGGGGTT	640bp
	MPST_CpG153#8-R	cagtaatacgactcactatagggagaaggctCCCCCTCCAAAAAAACAAAC	
#9	MPST_CpG153#9-L	aggaagagagGTTTGTTTTTTTGGAGGGGG	225bp
	MPST_CpG153#9-R	cagtaatacgactcactatagggagaaggctCTCTCTAATACCACCAAATCACCC	

MPST CpG89	Primer Name	Primer Sequence	Product Length
#2	MPST_CpG89#2-L	aggaagagagTATATGAGGTTTGGTAGAGGGAGGT	322bp
	MPST_CpG89#2-R	cagtaatacgactcactatagggagaaggctTCCAACAACTACAAAAACTACCCAA	
#7	MPST_CpG89#7-L	aggaagagagGTTGGGTAGTTTTTGTAGTTGTTGG	453bp
	MPST_CpG89#7-R	cagtaatacgactcactatagggagaaggctAAATTCCAAATTCTCCTTAATATCCTC	
#8	MPST_CpG89#8-L	aggaagagagGAGGATATTAAGGAGAATTTGGAAT	178bp
	MPST_CpG89#8-R	cagtaatacgactcactatagggagaaggctAAAAAAAATCAAATCCAAAACACTA	
#18	MPST_CpG89#18-L	aggaagagagTAAAAGAAAGGTTAAGTTTAGGGTATTG	178bp
	MPST_CpG89#18-R	cagtaatacgactcactatagggagaaggctAACATCAAAAAAAACCTAAAATCCC	

CBS CpG227	Primer Name	Primer Sequence	Product Length
#17	CBS_CpG227#17-L	aggaagagagGGATTTTTTGGGTTTATTTGTTTTTA	574
	CBS_CpG227#17-R	cagtaatacgactcactatagggagaaggctTCTAACACAAACCCTATCTAACCTAA	
#24	CBS_CpG227#24-L	aggaagagagTTTTTTGGATAGAGGATTTTAATTGG	616
	CBS_CpG227#24-R	cagtaatacgactcactatagggagaaggctAAACAAAACTAAAAACAAATAAACCCA	
#13	CBS_CpG227#13-L	aggaagagagGAGATTTTTTGTTTAGAGAGGGGAG	583
	CBS_CpG227#13-R	cagtaatacgactcactatagggagaaggctTCAAACTCATCAATAAAAATTCCTT	
#26	CBS_CpG227#26-L	aggaagagagGGGGTTTGGGATTTTTTATTTAGTT	484
	CBS_CpG227#26-R	cagtaatacgactcactatagggagaaggctCTATTTTACTTAATTAACTTCTTACCCTTC	
#28	CBS_CpG227#28-L	aggaagagagGTTTTTTTGTTTAGAGTTGATGGGT	124
	CBS_CpG227#28-R	cagtaatacgactcactatagggagaaggctCTTTCAAAAACTAAACCTAATCCCC	

CAT CpG58	Primer Name	Primer Sequence	Product Length
#1	CAT_CpG58#1-L	aggaagagagTGTTGATTTTTTAAGAGTTGAGAAAGT	313
	CAT_CpG58#1-R	cagtaatacgactcactatagggagaaggctCAAACCAAAATTAAAAACCCAATAA	
#3	CAT_CpG58#3-L	aggaagagagTTATTGGGTTTTTAATTTTGGTTTG	151
	CAT_CpG58#3-R	cagtaatacgactcactatagggagaaggctCCCTCAACAAACAAATCTACCTATT	
#5	CAT_CpG58#5-L	aggaagagagAATAGGTAGATTTGTTTGTTGAGGG	472
	CAT_CpG58#5-R	cagtaatacgactcactatagggagaaggctAACTCTCTACCTCTTTCCCCATAAA	

Mouse

Mpst CpG28	Primer Name	Primer Sequence	Product Length
#4	Mpst_CpG28#4-L	aggaagagagGGTGAAAGGGTTTTAGGTATTTTGT	437
	Mpst_CpG28#4-R	cagtaatacgactcactatagggagaaggctTTAAACACAACCCACTATTTAAACCA	

Mpst CpG64	Primer Name	Primer Sequence	Product Length
#1	Mpst_CpG64#1-L	aggaagagagGAATGTAGGTTTGTTTTTTTATTTTGG	497
	Mpst_CpG64#1-R	cagtaatacgactcactatagggagaaggctTTAACAAATTCTAATTCAACCAATAAC	
#7	Mpst_CpG64#7-L	aggaagagagTGGTTGAATTAGAATTTGTTAATTAGTTT	305
	Mpst_CpG64#7-R	cagtaatacgactcactatagggagaaggctCCTTTACTCTATATACATATAAAACCAACA	

Cbs CpG82	Mouse	Primer Sequence	Length
#12	Cbs_CpG82#12-L	aggaagagagGATTTTTTTAGTTTTGGATGGAGAT	327
	Cbs_CpG82#12-R	cagtaatacgactcactatagggagaaggctTAACCCAAAATATCCTACTTCCCTC	
#19	Cbs_CpG82#19-L	aggaagagagGGGATTTGAGTTGGGATTTTGT	498
	Cbs_CpG82#19-R	cagtaatacgactcactatagggagaaggctACCAATACCCCAAAAAACAATTTAC	
#20	Cbs_CpG82#20-L	aggaagagagTGGGTATAGATTTGGGGTTTTTT	305
	Cbs_CpG82#20-R	cagtaatacgactcactatagggagaaggctAATCTAAAACCAACCCAACCACA	

Sod1 CpG87	Primer Name	Primer Sequence	Product Length
#3	Sod1_#3-L	aggaagagagAAAGTTGGAAATGATATTGGGTATTT	700
	Sod1_#3-R	cagtaatacgactcactatagggagaaggctCAACCCCTACCCCAAACCAC	
#4	Sod1_#4-L	aggaagagagGTGGTTTGGGGTAGGGGTTG	304
	Sod1_#4-R	cagtaatacgactcactatagggagaaggctCCTAATACAAAACAACCCAAAACTTTA	

Sod2 CpG88	Primer Name	Primer Sequence	Product Length
#1	Sod2_#1-L	aggaagagagAGGAAAGTTTTTAGGGTTTTTTAGAAG	210
	Sod2_#1-R	cagtaatacgactcactatagggagaaggctTCTCCCCTATACCAAATTAATAAAA	
#3	Sod2_#3-L	aggaagagagGGTTTTTATTAATTTGGTATAGGGGA	392
	Sod2_#3-R	cagtaatacgactcactatagggagaaggctACCACAAAACCCAACCTCCTAC	
#4	Sod2_#4-L	aggaagagagGGTAGGAGGTTGGGTTTTGTG	399
	Sod2_#4-R	cagtaatacgactcactatagggagaaggctAAAACCACCACCAATACTAACTAACA	

Cat CpG30	Primer Name	Primer Sequence	Product Length
#2	Cat_#2-L	aggaagagagGTTGATTGGTGGAGTTTGAAGTTAT	492
	Cat_#2-R	cagtaatacgactcactatagggagaaggctTACAACTCTCTTCCACTTTACCCAA	

Gpx1 CpG30	Primer Name	Primer Sequence	Product Length
#5	Gpx1_#5-L	aggaagagagAGTTAAAAGGAGGTGTAGGGTTTTG	481
	Gpx1_#5-R	cagtaatacgactcactatagggagaaggctTTTTTAACTACAAAACAAAAATTTCCC	

Gpx4 CpG100	Primer Name	Primer Sequence	Product Length
#1	Gpx4_#1-L	aggaagagagTATTTTTAAAGGGGTTTTTTAAGGG	500
	Gpx4_#1-R	cagtaatacgactcactatagggagaaggctCAATAACCCACCATAATACCTACCA	
#8	Gpx4_#8-L	aggaagagagGTTTGGTAGGTATTATGGTGGGTTA	138
	Gpx4_#8-R	cagtaatacgactcactatagggagaaggctCACCTAAATACCCCCTTCTCTAACT	
#11	Gpx4_#11-L	aggaagagagGTTAGTTAGAGAAGGGGGTATTTAGG	462
	Gpx4_#11-R	cagtaatacgactcactatagggagaaggctCTAAAAAAACTCTTACAACAAAAACC	
