# Supplementary material for: Excess hydrogen sulfide and polysulfides production underlies a schizophrenia pathophysiology
Source: EMBO Mol Med. 2019 Oct 28;11(12):e10695. doi: 10.15252/emmm.201910695 (PMC6895609; doi:10.15252/emmm.201910695)
Supplement: Supplementary file 3 — Expanded View Figures PDF [file EMMM-11-e10695-s002.pdf]

Expanded View Figures

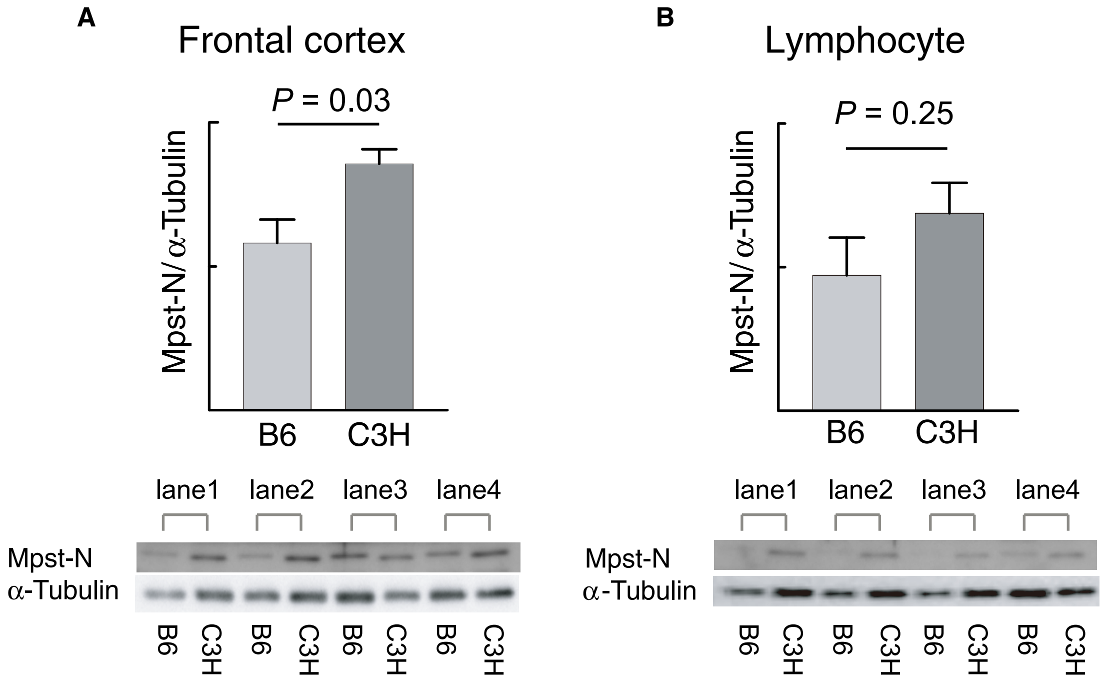

Figure EV1. Expression of Mpst examined using anti-N-terminal Mpst antibodies.

A, B Mpst protein levels in the brain and lymphocytes from B6 ($n = 4$) and C3H ($n = 4$) mice were quantified by standard Western blotting with anti-N-terminal Mpst antibodies. The expression levels of Mpst were normalized using α-tubulin. Bar graphs show the mean expression levels of Mpst in brain (A) and lymphocyte (B) tissues.

Data information: P values were calculated by using unpaired two-tailed t-test. The values represent the mean ± SD.

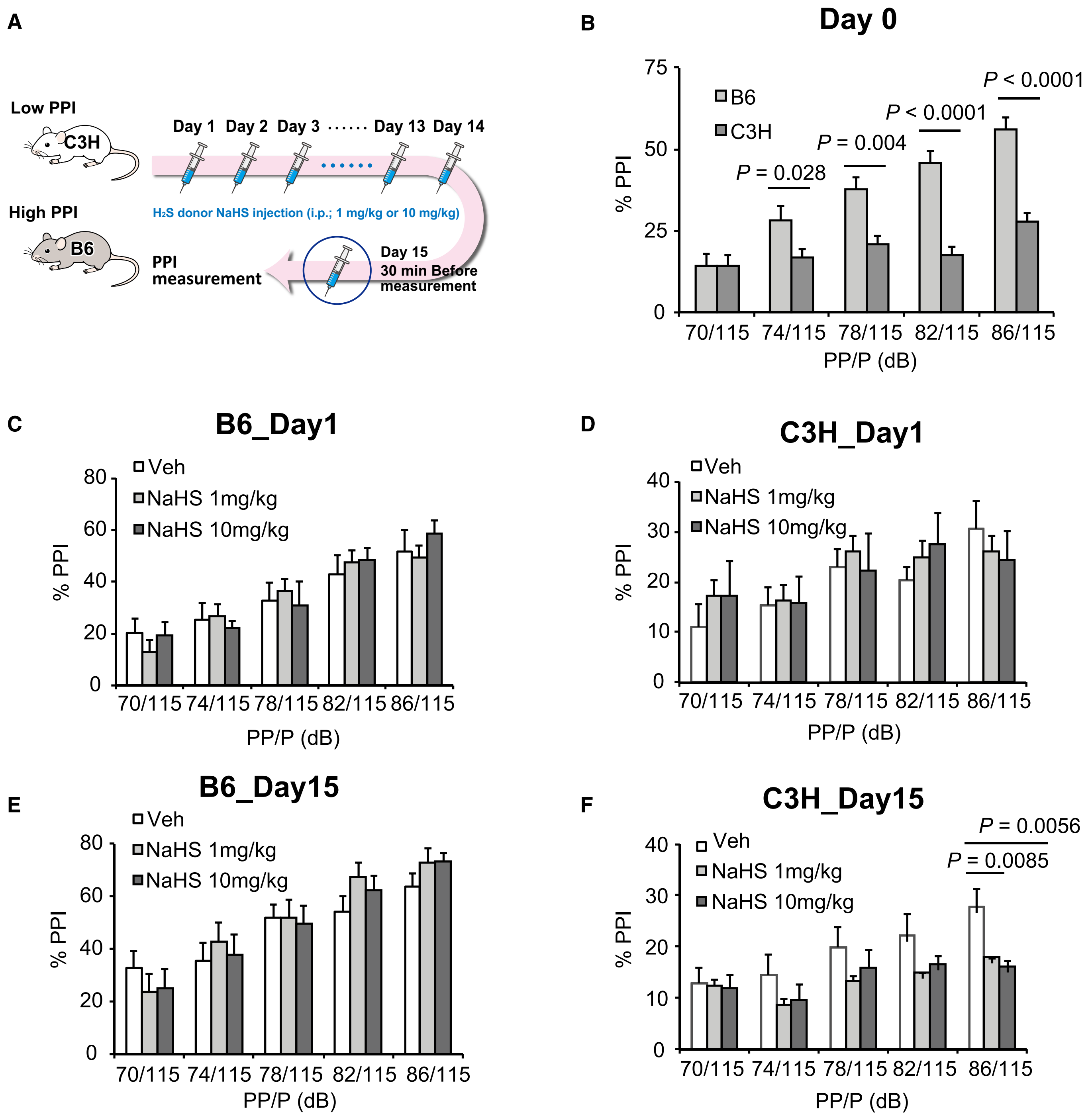

Figure EV2. Effects of chronic NaHS administration on PPI (prepulse inhibition).

A Schematic illustration of the experimental design.

B PPI (%) at 5 different prepulse levels between untreated B6 (n = 12) and C3H (n = 12) mice. *X*-axis shows prepulse (PP) levels. Pulse (P) sound was 115 dB.

C, D PPI levels of B6 (C) and C3H (D) mice after the first administration of vehicle (Veh: PBS) and two different doses of NaHS. NaHS was intraperitoneally injected once a day, and PPI was measured 30 min after the 1st injection. Each dose group contained n = 12 animals.

E, F PPI levels of B6 (E) and C3H (F) mice after the 15th administration of vehicle (Veh: PBS) and two different doses of NaHS. NaHS was intraperitoneally injected once a day, and PPI was measured 30 min after the 15th injection. Each dose group contained n = 12 animals.

Data information: *P* values were calculated using Tukey's *post hoc* test after one-way ANOVA. All data are shown as the mean ± SEM.

© 2019 The Authors

Sample		Target	Copies/microliter*	SEM
BA8	n = 6	*MPST*	259.3	38.1
BA8	n = 6	*CBS*	2261.7	257.5
BA8	n = 6	*CTH*	1171.5	120.1
iPS	n = 6	*MPST*	1338.5	144.7
iPS	n = 6	*CBS*	15214.0	1665.2
iPS	n = 6	*CTH*	7426.1	1969.7
NS	n = 6	*MPST*	2187.3	268.8
NS	n = 6	*CBS*	7043.1	383.0
NS	n = 6	*CTH*	1395.3	73.2

Sample		Target	Copies/microliter*	SEM
HF	n = 6	*MPST*	2130.9	503.1
HF	n = 6	*CBS*	103.7	22.9
HF	n = 6	*CTH*	65.6	5.9
Mouse	n = 1	*Mpst*	1360.8	-
Mouse	n = 1	*Cbs*	1177.5	-
Mouse	n = 1	*Cth*	518.8	-
PBC	n = 4	*MPST*	384.1	36.8
PBC	n = 4	*CBS*	203.2	66.0
PBC	n = 4	*CTH*	165.2	15.1

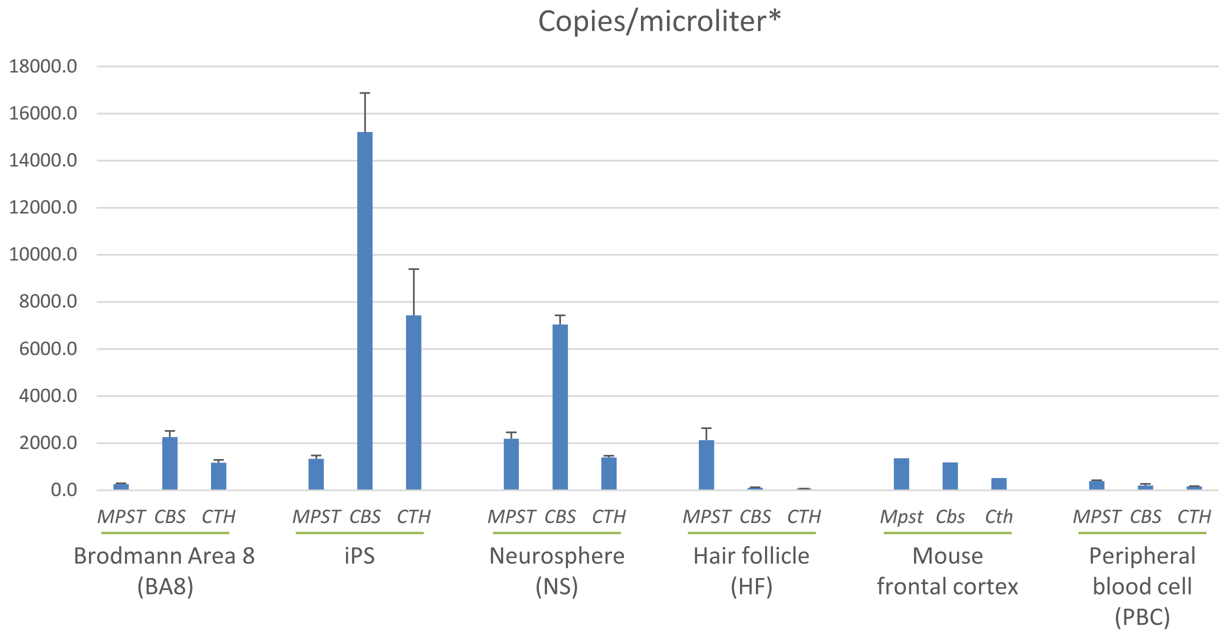

*Copy number per 1 μl of total RNA (10ng)

Figure EV3. Absolute expression levels of genes for H_2S-synthesizing enzymes in human and mouse tissues.
Transcript expression levels were measured by digital PCR. Samples of BA8, iPSC-derived NS, HF, and PBC were from human.

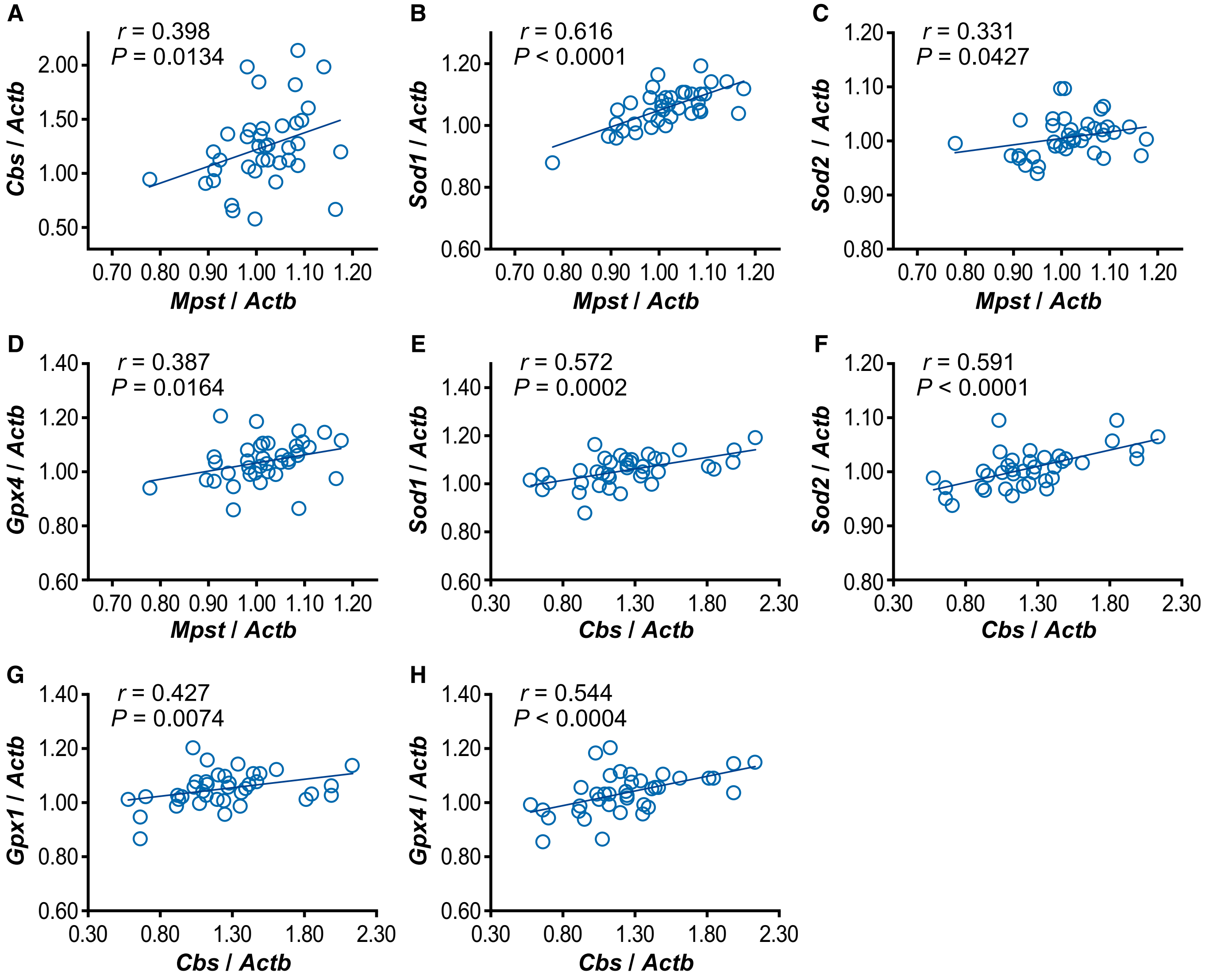

Figure EV4. Correlation between expression levels of *Mpst* and *Cbs* (A) and between *Mpst*/*Cbs* and antioxidant genes (B–H) in poly-I:C administered mice.

A–H Correlation was analyzed using frontal cortex of 38 mice. Each gene expression level was normalized to that of *Actb*.

Data information: Statistical evaluations were performed using Spearman's rank correlation test.

 © 2019 The Authors

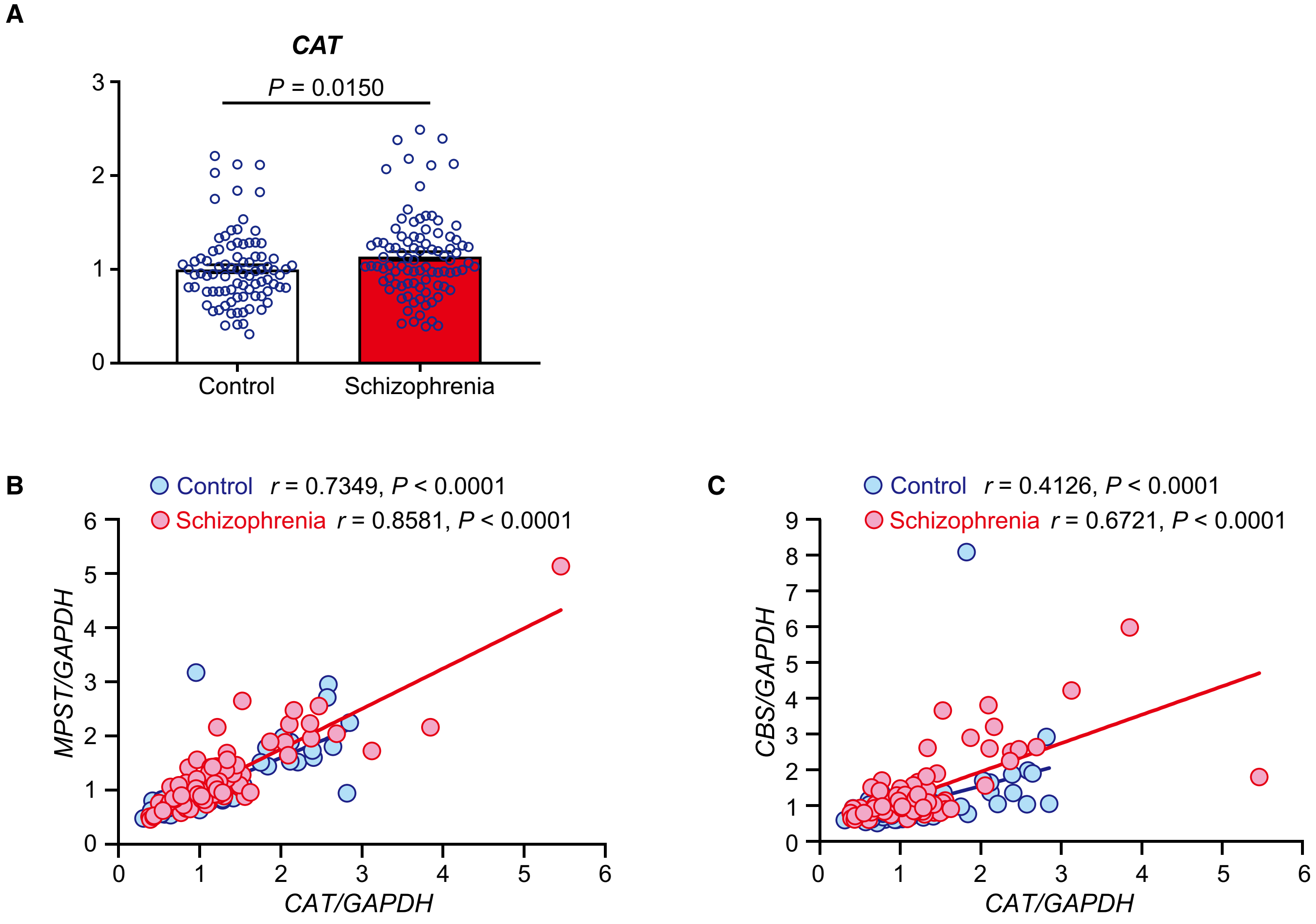

Figure EV5. Analysis of antioxidant gene *CAT* and genes for H_2S-synthesizing enzymes in human postmortem brain.

A Expression levels of *CAT* were measured in the frontal cortex (BA8) from control (*n* = 93) and schizophrenia (*n* = 95) (see Appendix Table S7). The values represent the mean ± SEM.

B, C Correlation between expression levels of *CAT* and *MPST*/*CBS*. The same samples as analyzed in (A) were used. Each gene expression level was normalized to that of *GAPDH*.

Data information: Statistical evaluations were performed using Mann–Whitney *U* test (A) and Spearman's rank correlation test (B, C).

© 2019 The Authors
