# Supplementary material for: Excess hydrogen sulfide and polysulfides production underlies a schizophrenia pathophysiology
Source: EMBO Mol Med. 2019 Oct 28;11(12):e10695. doi: 10.15252/emmm.201910695 (PMC6895609; doi:10.15252/emmm.201910695)
Supplement: Supplementary file 4 — Source Data for Figure 1 [file EMMM-11-e10695-s003.pdf]

Mascot Search Results

```
User         :
Email        :
Search title : H180001_H18
MS data file : C:¥Program Files¥Kompact¥data¥Tsukuba_Univ¥Ide¥060209C3F_Lys_tag¥asc¥H18.txt
Database     : MSDB 20050227 (1942918 sequences; 629040812 residues)
Taxonomy     : Mus musculus (house mouse) (73733 sequences)
Timestamp    : 18 Jan 2007 at 13:43:38 GMT
Top Score    : 64 for THTM_MOUSE, 3-mercaptopyruvate sulfurtransferase (EC 2.8.1.2) (MST).- Mus musculus (Mouse).
```

Probability Based Mowse Score

Ions score is -10*Log(P), where P is the probability that the observed match is a random event.
Protein scores greater than 61 are significant (p<0.05).

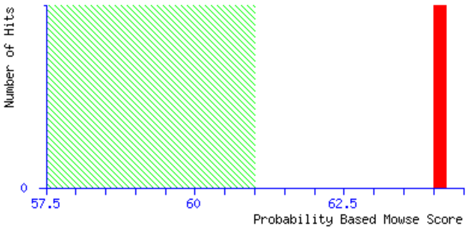

Concise Protein Summary Report

Format As | Concise Protein Summary | Help

Significance threshold p< 0.05 Max. number of hits 200

Re-Search All | Search Unmatched

1. THTM_MOUSE Mass: 33984 Score: 64 Expect: 0.029 Queries matched: 4
3-mercaptopyruvate sulfurtransferase (EC 2.8.1.2) (MST).- Mus musculus (Mouse).
AAH04079 Mass: 34115 Score: 64 Expect: 0.029 Queries matched: 4
BC004079 NID: - Mus musculus
A39962 Mass: 129137 Score: 41 Expect: 5.5 Queries matched: 4
kinase-related transforming protein (abl) (EC 2.7.1.-) type I - mouse
Q8C1X4_MOUSE Mass: 129075 Score: 41 Expect: 5.5 Queries matched: 4
Mus musculus embryo RCB-0545 OHTA cDNA, RIKEN full-length enriched library, clone:G430110C19 produc
Q6PCM5_MOUSE Mass: 131046 Score: 41 Expect: 6 Queries matched: 4
Abl1 protein.- Mus musculus (Mouse).
Q9D5A5_MOUSE Mass: 26825 Score: 46 Expect: 1.9 Queries matched: 3
Mus musculus adult male testis cDNA, RIKEN full-length enriched library, clone:4930481F22 product:H
Q61055_MOUSE Mass: 44447 Score: 37 Expect: 13 Queries matched: 3
Protein tyrosine kinase (Fragment).- Mus musculus (Mouse).
CAC51130 Mass: 9540 Score: 35 Expect: 24 Queries matched: 2
Sequence 21 from Patent WO0149714 (Fragment).- Mus musculus (Mouse).
CAA80012 Mass: 13869 Score: 34 Expect: 33 Queries matched: 2
IMMUNOGLOBULIN VARIABLE REGION (FRAGMENT).- Mus musculus domesticus (western European house mouse).
Q9QXE6_MOUSE Mass: 13455 Score: 33 Expect: 36 Queries matched: 2
Malonyl-CoA decarboxylase.- Mus musculus (Mouse).
Q5R1H0_MOUSE Mass: 12634 Score: 33 Expect: 41 Queries matched: 2
TRAV13D-1 (Fragment).- Mus musculus (Mouse).
Q8BIV0_MOUSE Mass: 66253 Score: 32 Expect: 45 Queries matched: 3
Mus musculus adult male hippocampus cDNA, RIKEN full-length enriched library, clone:C630028L02 prod
Q8C801_MOUSE Mass: 20175 Score: 31 Expect: 66 Queries matched: 2
Mus musculus 0 day neonate cerebellum cDNA, RIKEN full-length enriched library, clone:C230071H18 pr
BAC36903 Mass: 19670 Score: 29 Expect: 85 Queries matched: 2
AK077617 NID: - Mus musculus
Q8BX31_MOUSE Mass: 29986 Score: 27 Expect: 1.5e+002 Queries matched: 2
Mus musculus ES cells cDNA, RIKEN full-length enriched library, clone:C330006M16 product:similar to
Q99KP8_MOUSE Mass: 21354 Score: 27 Expect: 1.5e+002 Queries matched: 2
Tpd52 protein.- Mus musculus (Mouse).
Q8BGK7_MOUSE Mass: 119756 Score: 27 Expect: 1.6e+002 Queries matched: 3
RTN4 (Reticulon 4).- Mus musculus (Mouse).
Q8C1M4_MOUSE Mass: 25235 Score: 26 Expect: 1.9e+002 Queries matched: 2
Mus musculus adult male tongue cDNA, RIKEN full-length enriched library, clone:2310075G14 product:T
S54354 Mass: 110751 Score: 26 Expect: 1.9e+002 Queries matched: 3
inter-alpha-inhibitor H2 chain - mouse
Q9R1Q6_MOUSE Mass: 29599 Score: 26 Expect: 1.9e+002 Queries matched: 2
Clast1 (LR8 protein) (Mus musculus 10 day old male pancreas cDNA, RIKEN full-length enriched librar
Q9WU52_MOUSE Mass: 29575 Score: 26 Expect: 1.9e+002 Queries matched: 2
LR8.- Mus musculus (Mouse).
Q8K016_MOUSE Mass: 110769 Score: 26 Expect: 2e+002 Queries matched: 3
Inter-alpha trypsin inhibitor, heavy chain 2.- Mus musculus (Mouse).
Q9D1B8_MOUSE Mass: 21310 Score: 26 Expect: 2e+002 Queries matched: 2
Mus musculus 18-day embryo whole body cDNA, RIKEN full-length enriched library, clone:1110015M06 pr
BAB24225 Mass: 21490 Score: 26 Expect: 2e+002 Queries matched: 2
AK005762 NID: - Mus musculus
AAD23879 Mass: 21391 Score: 26 Expect: 2e+002 Queries matched: 2
AF130367 NID: - Mus musculus
BAC32928 Mass: 27832 Score: 26 Expect: 2.1e+002 Queries matched: 2

AK046964 NID: - Mus musculus

Q8BGM9_MOUSE Mass: 132219 Score: 25 Expect: 2.1e+002 Queries matched: 3
RTN4 (Reticulon 4).- Mus musculus (Mouse).

Q8K3G8_MOUSE Mass: 132297 Score: 25 Expect: 2.1e+002 Queries matched: 3
Nogo-A.- Mus musculus (Mouse).

Q80WG1_MOUSE Mass: 30111 Score: 25 Expect: 2.3e+002 Queries matched: 2
Kcnh2 protein (Fragment).- Mus musculus (Mouse).

Q6P227_MOUSE Mass: 26226 Score: 25 Expect: 2.4e+002 Queries matched: 2
Transmembrane trafficking protein.- Mus musculus (Mouse).

Q9D1D4_MOUSE Mass: 26223 Score: 25 Expect: 2.4e+002 Queries matched: 2
Mus musculus 18-day embryo whole body cDNA, RIKEN full-length enriched library, clone:1110014C03 pr

A39529 Mass: 105763 Score: 24 Expect: 2.9e+002 Queries matched: 3
cadherin-associated protein, 102K - mouse

Q6NV50_MOUSE Mass: 105766 Score: 24 Expect: 2.9e+002 Queries matched: 3
Catenin alpha 1.- Mus musculus (Mouse).

Q8BMD3_MOUSE Mass: 37085 Score: 24 Expect: 2.9e+002 Queries matched: 2
Mus musculus 12 days embryo male wolffian duct includes surrounding region cDNA, RIKEN full-length

Q8BMU7_MOUSE Mass: 37040 Score: 24 Expect: 2.9e+002 Queries matched: 2
Mus musculus adult male small intestine cDNA, RIKEN full-length enriched library, clone:2010200I21

AAH03432 Mass: 37129 Score: 24 Expect: 2.9e+002 Queries matched: 2
BC003432 NID: - Mus musculus

Q99K20_MOUSE Mass: 36524 Score: 24 Expect: 2.9e+002 Queries matched: 2
Ldh1 protein (Fragment).- Mus musculus (Mouse).

LDHA_MOUSE Mass: 38523 Score: 24 Expect: 3.1e+002 Queries matched: 2
L-lactate dehydrogenase A chain (EC 1.1.1.27) (LDH-A) (LDH muscle subunit) (LDH-M).- Mus musculus (

Q9CX58_MOUSE Mass: 30640 Score: 24 Expect: 3.1e+002 Queries matched: 2
Mus musculus 12 days embryo male wolffian duct includes surrounding region cDNA, RIKEN full-length

DEMSLM Mass: 38653 Score: 24 Expect: 3.2e+002 Queries matched: 2
L-lactate dehydrogenase (EC 1.1.1.27) chain M - mouse

Q5U267_XENLA Mass: 38654 Score: 24 Expect: 3.2e+002 Queries matched: 2
Hypothetical protein.- Xenopus laevis (African clawed frog).

Q9CUL1_MOUSE Mass: 35438 Score: 23 Expect: 3.4e+002 Queries matched: 2
Mus musculus adult male testis cDNA, RIKEN full-length enriched library, clone:4930473A06 product:h

Q922A0_MOUSE Mass: 38701 Score: 23 Expect: 3.4e+002 Queries matched: 2
Eno2 protein (Fragment).- Mus musculus (Mouse).

Q5XF89_MOUSE Mass: 144127 Score: 23 Expect: 3.5e+002 Queries matched: 3
Type V P-type ATPase isoform 3.- Mus musculus (Mouse).

Q8BW60_MOUSE Mass: 45802 Score: 23 Expect: 3.8e+002 Queries matched: 2
Mus musculus 2 days pregnant adult female oviduct cDNA, RIKEN full- length enriched library, clone:

Q99KE9_MOUSE Mass: 51277 Score: 23 Expect: 3.9e+002 Queries matched: 2
Nasp protein (Mus musculus 2 days neonate thymus thymic cells cDNA, RIKEN full-length enriched libr

AAH19812 Mass: 39222 Score: 23 Expect: 3.9e+002 Queries matched: 2
BC019812 NID: - Mus musculus

Q8CBE4_MOUSE Mass: 48620 Score: 23 Expect: 4.1e+002 Queries matched: 2
Mus musculus 16 days neonate cerebellum cDNA, RIKEN full-length enriched library, clone:9630044P12

AAB60876 Mass: 48634 Score: 23 Expect: 4.1e+002 Queries matched: 2
AF001871 NID: - Mus musculus

Q9CYR7_MOUSE Mass: 34740 Score: 22 Expect: 4.3e+002 Queries matched: 2
Mus musculus 10, 11 days embryo whole body cDNA, RIKEN full-length enriched library, clone:2810472M

Search Parameters

```
Type of search         : Peptide Mass Fingerprint
Enzyme                 : Trypsin
Fixed modifications    : Carbamidomethyl (C),Dihydro-Imidazol(K)
Variable modifications : Oxidation (M)
Mass values            : Monoisotopic
Protein Mass           : Unrestricted
Peptide Mass Tolerance : ± 0.5 Da
Peptide Charge State   : 1+
Max Missed Cleavages   : 1
Number of queries      : 4
```

Mascot: http://www.matrixscience.com/
