# Supplementary figures and images for: Excess hydrogen sulfide and polysulfides production underlies a schizophrenia pathophysiology
Source: EMBO Mol Med. 2019 Oct 28;11(12):e10695. doi: 10.15252/emmm.201910695 (PMC6895609; doi:10.15252/emmm.201910695)

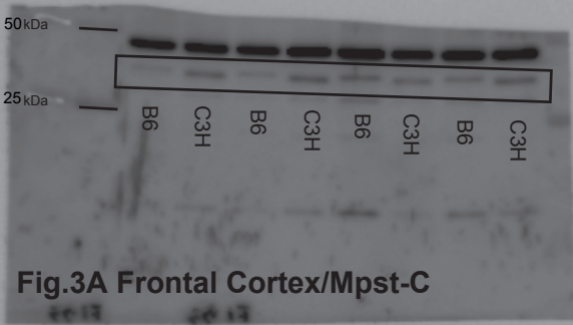

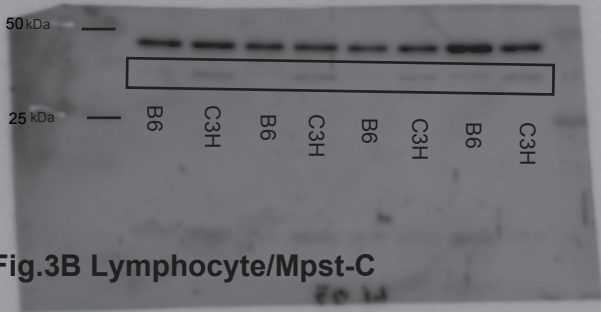

**Fig.3B Lymphocyte/Mpst-C**

Supplement: Supplementary file 5 — Source Data for Figure 3 [file EMMM-11-e10695-s004.pdf]

# WB image for Fig. 7A: MPST+GAPDH

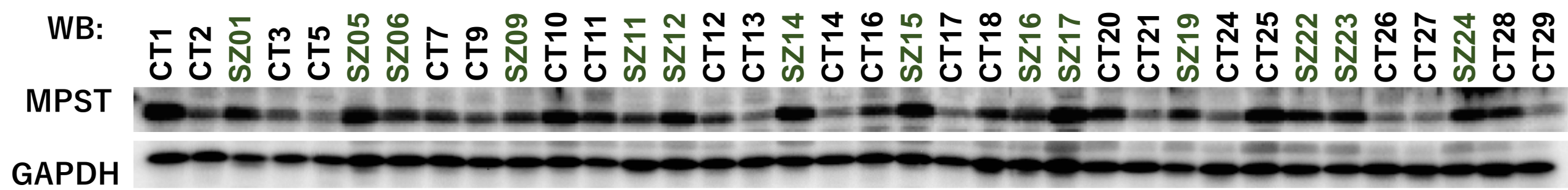

CT: Control  
SZ: Schizophrenia

# WB: anti-MPST

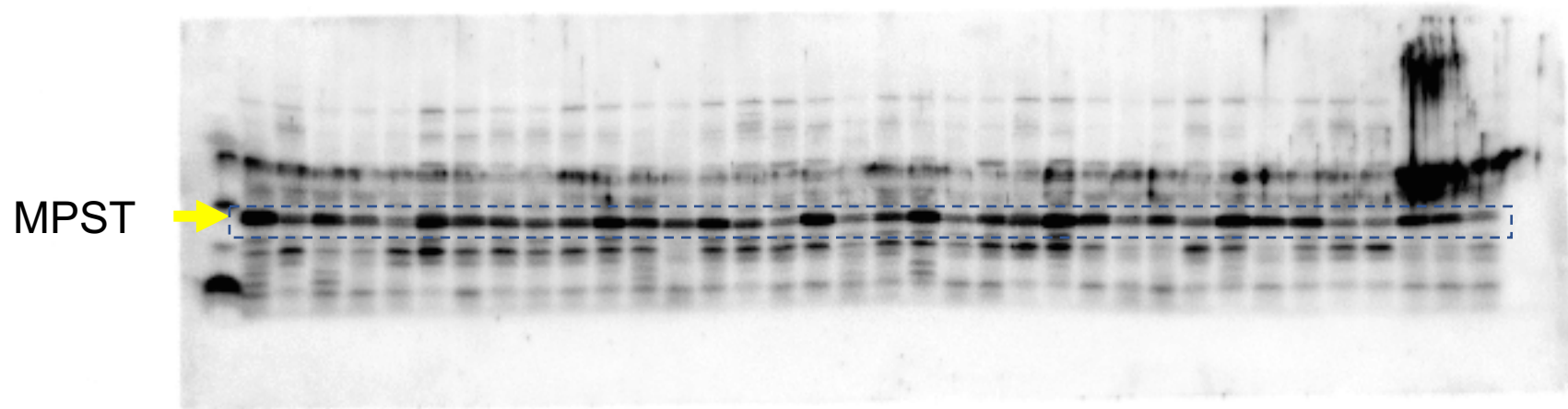

# WB: anti-GAPDH

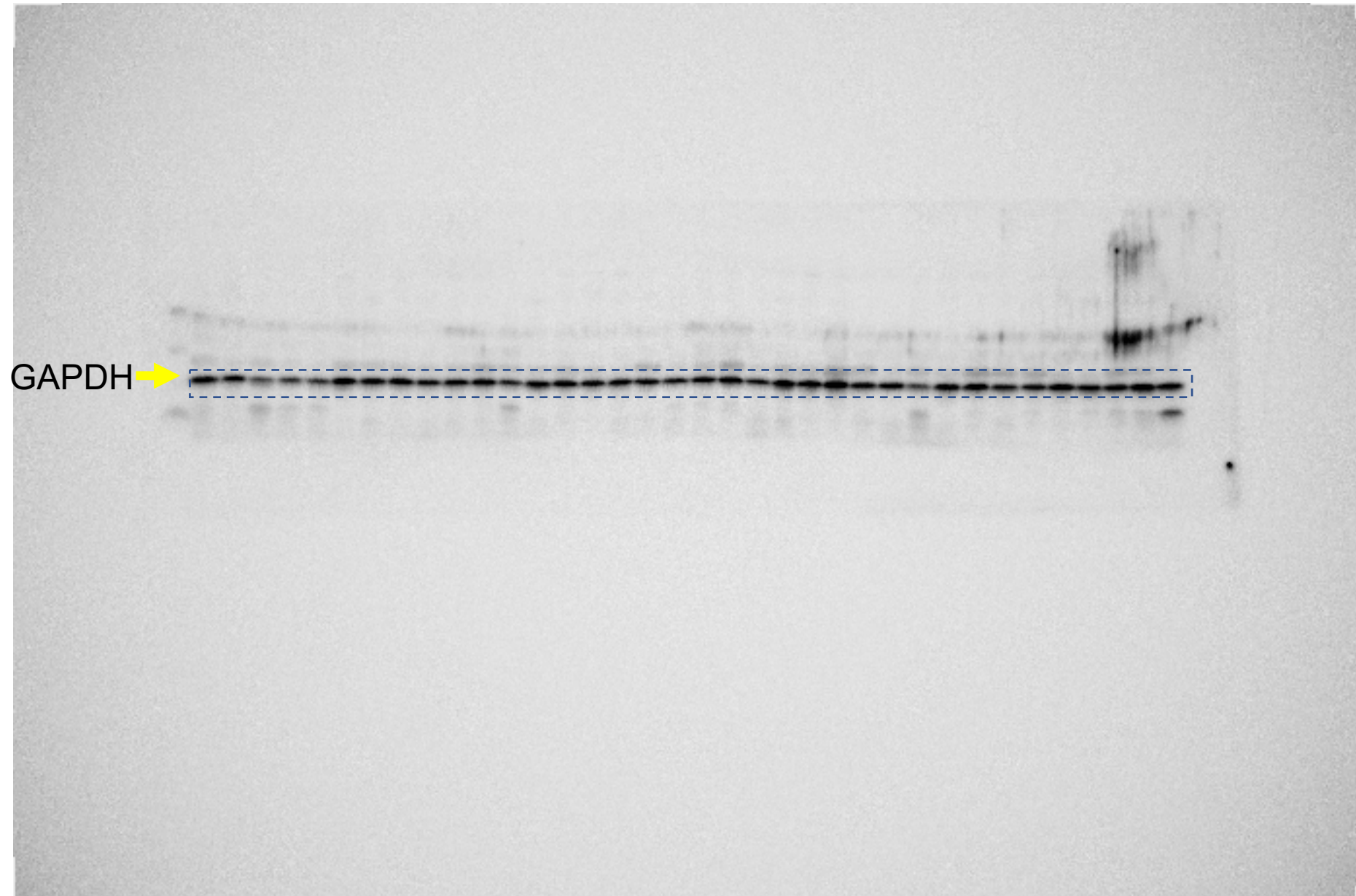

Supplement: Supplementary file 6 — Source Data for Figure 7 [file EMMM-11-e10695-s005.pdf]
